# Gene Expression Profiles Deciphering Rice Phenotypic Variation between Nipponbare (Japonica) and 93-11 (Indica) during Oxidative Stress

**DOI:** 10.1371/journal.pone.0008632

**Published:** 2010-01-08

**Authors:** Fengxia Liu, Wenying Xu, Qiang Wei, Zhenghai Zhang, Zhuo Xing, Lubin Tan, Chao Di, Dongxia Yao, Chunchao Wang, Yuanjun Tan, Hong Yan, Yi Ling, Chuanqing Sun, Yongbiao Xue, Zhen Su

**Affiliations:** 1 State Key Laboratory of Plant Physiology and Biochemistry, College of Biological Sciences, China Agricultural University, Beijing, China; 2 Laboratory of Molecular and Developmental Biology and National Centre for Plant Gene Research, Institute of Genetics and Developmental Biology, Chinese Academy of Sciences, Beijing, China; 3 Department of Plant Genetics and Breeding and State Key Laboratory of Agrobiotechnology, China Agricultural University, Beijing, China; Michigan State University, United States of America

## Abstract

Rice is a very important food staple that feeds more than half the world's population. Two major Asian cultivated rice (*Oryza sativa L.*) subspecies, *japonica* and *indica*, show significant phenotypic variation in their stress responses. However, the molecular mechanisms underlying this phenotypic variation are still largely unknown. A common link among different stresses is that they produce an oxidative burst and result in an increase of reactive oxygen species (ROS). In this study, methyl viologen (MV) as a ROS agent was applied to investigate the rice oxidative stress response. We observed that 93-11 (*indica*) seedlings exhibited leaf senescence with severe lesions under MV treatment compared to Nipponbare (*japonica*). Whole-genome microarray experiments were conducted, and 1,062 probe sets were identified with gene expression level polymorphisms between the two rice cultivars in addition to differential expression under MV treatment, which were assigned as Core Intersectional Probesets (CIPs). These CIPs were analyzed by gene ontology (GO) and highlighted with enrichment GO terms related to toxin and oxidative stress responses as well as other responses. These GO term-enriched genes of the CIPs include glutathine S-transferases (GSTs), P450, plant defense genes, and secondary metabolism related genes such as chalcone synthase (CHS). Further insertion/deletion (InDel) and regulatory element analyses for these identified CIPs suggested that there may be some eQTL hotspots related to oxidative stress in the rice genome, such as GST genes encoded on chromosome 10. In addition, we identified a group of marker genes individuating the *japonica* and *indica* subspecies. In summary, we developed a new strategy combining biological experiments and data mining to study the possible molecular mechanism of phenotypic variation during oxidative stress between Nipponbare and 93-11. This study will aid in the analysis of the molecular basis of quantitative traits.

## Introduction

Rice (*Oryza sativa*) is the major food staple for about half of the world's population, and it also is a model monocot plant for molecular and genetic studies. *Oryza sativa L. ssp indica* (Hsien) and *Oryza sativa L. ssp japonica* (Keng) are two major Asian cultivated rice (*Oryza sativa L.*) subspecies [1], [2], [3]. These two subspecies have been distinguished based on morphological characters and geographical distribution for 2,000 years. Indica and Japonica rice originated from different ancestors and they diverged about 0.2∼0.44 million years ago [4], [5]. From Khush's report, *indica* was probably domesticated in eastern India and *japonica* somewhere in South China [6]. These variations affect genomic structure and may cause intra-specific phenotypic adaptations. For example, there exists variance of seed maturity, seed quality, stress and defense tolerance between the two subspecies. Genome-wide comparative analyses were conducted on DNA sequences derived from *indica* and *japonica* rice [5], [6], [7], [8], [9], [10], [11], [12], [13]. In recent years, sequence variance analysis between the two rice subspecies have become well-established due to the publicly available rice genome, including the genome sequences of the *japonica* variety Nipponbare and *indica* variety 93-11 [14], [15], [16], [17], and a genetic map for 150 rice recombinant inbred lines constructed by the recently introduced next-generation sequencing technology [18]. In order to further elucidate genetic differences between rice subspecies, an approach using Gene Ontology (GO) analysis together with genomic variation analysis was conducted by different research groups [19], [20]. Several GO terms were highlighted with significant enrichment, including production of defense-related compounds, cell wall components, cell signaling proteins, and transcription factors. The GO analysis results indicated that there was positive selection either by natural means or by human interests during *indica*–*japonica* differentiation. However, the underlying regulatory mechanisms of rice phenotypic variation during development or during stress conditions between the two subspecies are largely unknown.

Recently, plant transcriptome mapping studies (such as microarray and high-throughput transcriptome sequencing) have become a popular way to reveal different types of genetic variation and study the possible molecular mechanism related to transcriptional divergence for genes under natural settings or artificial selection that might influence phenotypes [21], [22], [23], [24], [25], [26], [27], [28], [29], [30], [31]. Variation in gene regulation was discovered to be a very important mechanism. For example, gene expression level polymorphisms (ELPs) in *Arabidopsis* were observed between the Eil-0 and Lc-0 accessions [26] and these ELPs may be candidates for quantitative trait loci (QTLs) that influence phenotypic variability. Therefore, the genome-wide analysis of transcripts make it feasible for us to dissect complex traits into component gene expression pathways involved for rice *indica* and *japonica* during development and environmental stress.

Rice stress tolerance is a major factor directly related to yield improvement. The common link among different stresses such as drought, salt, extreme temperature, nutrient deprivation, UV-B radiation and air pollutants [32], [33], [34], [35], [36], [37], [38], [39], is that they all produce an oxidative burst with damaging effects on cellular macromolecules such as lipids, enzymes and DNA. Methyl viologen (MV) is a redox-active constituent of bipyridyl herbicides and is widely used as an oxidant forming the toxic superoxide radical during the study of oxidative stress in plants [40], [41], [42], [43], [44].

Through methyl viologen treatment, we observed significant variation in oxidative stress response and leaf senescence between 93-11 (*indica*) and Nipponbare (*japonica*). We further use microarray data mining and a comparative genomics approach together with gene ontology (GO) analysis to study the molecular mechanisms of resistance to methyl viologen. Our study will assist in the search for important marker genes for rice phenotypic variation between *japonica* and *indica* subspecies, and will also yield new insight into the molecular basis and evolution of transcriptional regulatory networks underlying phenotypic variation. Our findings may improve rice defense responses and seed quality in the future.

## Results

### Effect of Methyl Viologen (MV) Treatment on Rice 93-11 (*Indica*) and Nipponbare (*Japonica*) Seedling Growth

**Figure 1:**
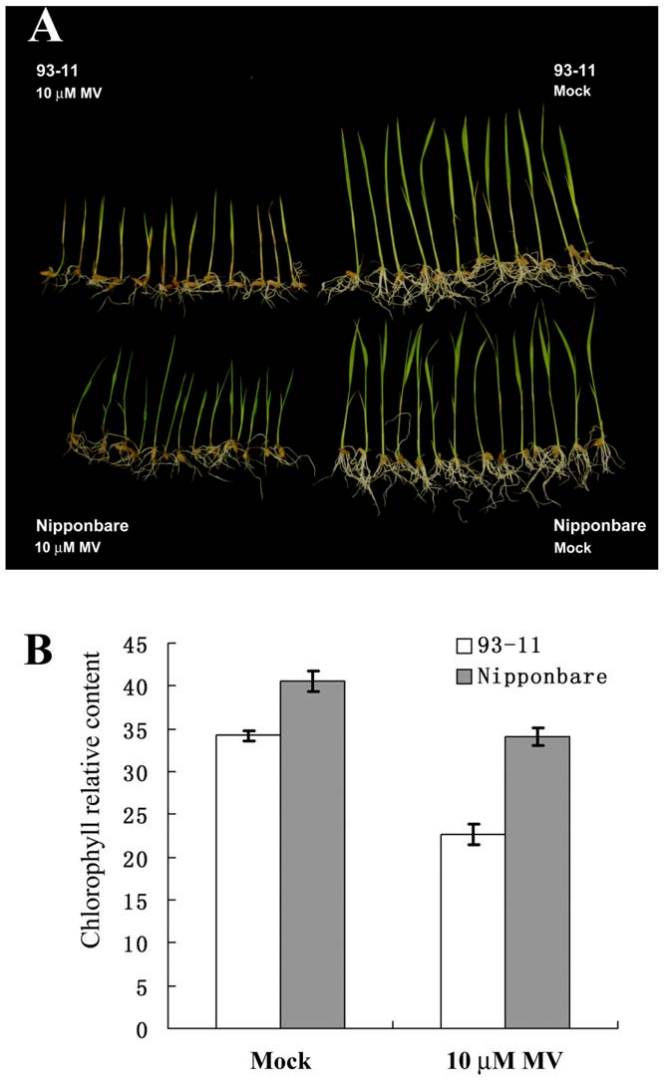
Differential responsiveness of rice *japonica* variety (Nipponbare) and *indica* variety (93-11) to MV treatment. **A.** 93-11 (up) and Nipponbare (down) sprouted seeds were mock-treated (water) or treated with 10 µM MV. **B.** The chlorophyll content of seedling plants after treatment.

An experiment was designed to test Nipponbare (*japonica* variety) and 93-11 (*indica* variety) phenotypic divergence under oxidation stress using methyl viologen (paraquat, a herbicide that induces oxidative stresses in plants). As shown in Figure S1, one-week-old 93-11 and Nipponbare seedlings were incubated for 5 days, 7 days and 10 days in solution containing 10 μM, 15 μM, and 20 μM MV or only water as a mock-treated controls. After 5–10 days' growth in solution with different concentration of MV, differences in leaf senescence between Nipponbare and 93-11 became visible. Nipponbare and 93-11 seedlings under mock treatment both grew normally, and all the leaves were green. Under MV treatment, both Nipponbare and 93-11 seedling plants were dwarfed, but significant phenotype divergence was apparent: the leaves of 93-11 became yellow, presented with severe lesions and died at high MV concentration, while the seedling of Nipponbare were healthier under the same conditions and exhibited relatively lower levels of leaf senescence. To quantify the phenotypic variation, three independent groups (20 rice seedlings in each group) of 93-11 and Nipponbare were treated either with 10μM MV or mock treated, and one replicate is shown in Figure 1A. We measured chlorophyll content to distinguish differences between the two cultivars (shown in Figure 1B). The chlorophyll content in 93-11 was more depleted under MV treatment than that the chlorophyll content of Nipponbare.

Overall, the Nipponbare seedling plants demonstrated strong oxidative stress resistance compared to 93-11. The significant phenotypic variance observed between 93-11 and Nipponbare may be caused by genomic and transcriptional variation between these two cultivars.

### Transcriptome Map of Rice 93-11 (*Indica*) and Nipponbare (*Japonica*) under MV Treatment

In order to investigate transcriptome-level differences between 93-11 and Nipponbare in response to MV treatment, we conducted a microarray analysis using the 57K Affymetrix rice whole genome array. Because the Affymetrix rice whole genome array was designed mainly from the *japonica* genome, we first tested for probe-level polymorphisms between the *japonica* and *indica* genome sequences. Within 628,725 probes, there are 573,313 probes complementary to the rice genomes, including about 93.4% of probes with single or multiple top hits on both *japonica* and *indica* genome, and only 6.6% of probes hitting either *japonica* or *indica*. Therefore, the polymorphic probes likely will not significantly impact on our results.

One week-old Nipponbare and 93-11 seedlings were incubated in a solution containing 10 μM MV or mock treated in water for 24 hours. Three sets of biological samples were collected independently and a total of 12 chips were analyzed (the experimental details are shown in Materials and Methods). The biological reproducibility of the microarray experiment was determined and the pair-wise scatter plots are shown in Figure S2. Among biological replicates of each cultivar under MV or mock treatment, nearly all of the probe sets fell along the diagonal, which indicated that no major variation was observed. The correlation coefficients were greater than 0.98 and the false discovery rates (FDR) were less than 2% (details are listed in Figure S2). For the scatter plots obtained between different treatments and cultivars, some probe sets fell above or below diagonal lines, indicating their differential hybridization intensity which corresponds to variation in gene expression (illustrated by colored boxes in Figure S2).

**Figure 2:**
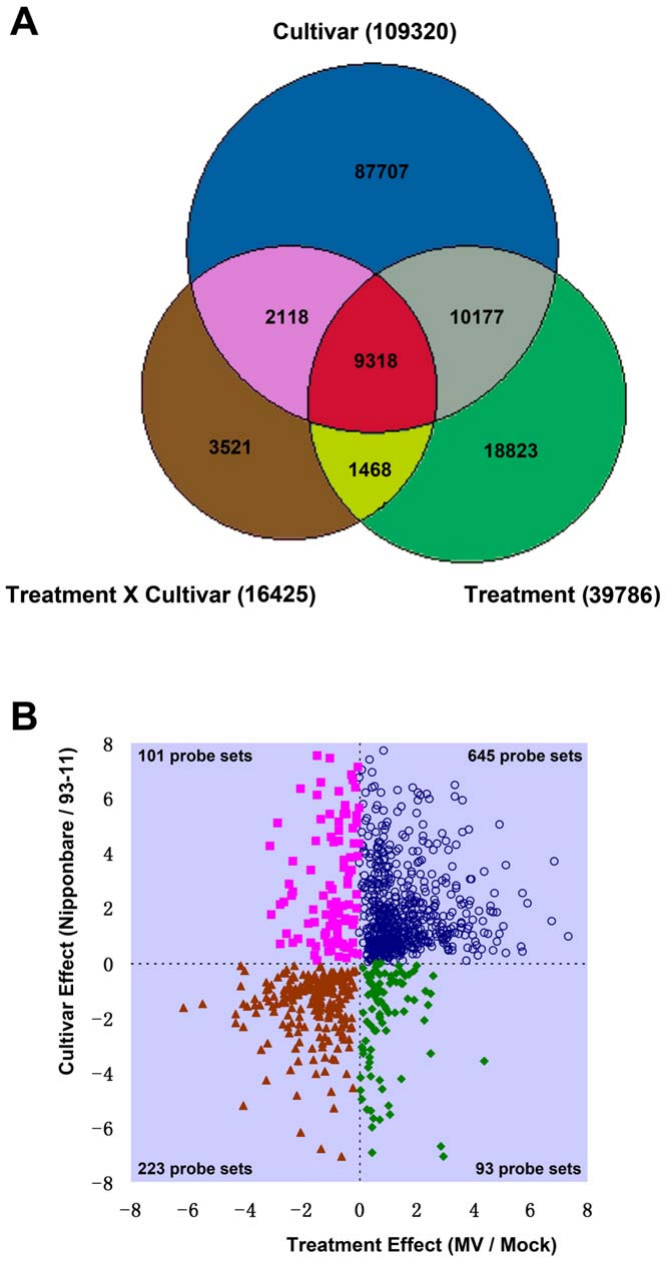
Summary of the transcription profiles of different rice cultivars (Nipponbare vs 93-11) under 10 µM MV treatment. **A.** The Venn diagram illustrates probe level expression affected by two factors -cultivar (Nipponbare vs. 93-11) and treatment (10 µM MV vs. mock). The number of probes showing a significant change at P< = 0.01 is shown. **B.** The cultivar and treatment effects of Core Intersectional Probesets (CIPs). The blue circles at top-right represent the CIPs preferentially expressed in Nipponbare and up-regulated by MV treatment. The brown triangles at bottom-left represent the CIPs preferentially expressed in 93-11 and down-regulated by MV treatment. The yellow squares at upper-left represent the CIPs preferentially expressed in Nipponbare and down-regulated by MV treatment. The green diamonds at bottom-right represents the CIPs preferentially expressed in 93-11 and up-regulated by MV treatment.

To further elucidate the effects of MV and/or rice cultivars on transcriptional variation, two-way ANOVA analysis was applied on the raw hybridization data (CEL file for probe level intensity), and the results are summarized in Figure 2. The two factors were cultivar (Nipponbare vs. 93-11) and treatment (MV vs. Mock), as well as the interaction of the two factors (cultivars×treatment). The Venn diagram given in Figure 2A illustrates the number of probes exhibiting significant changes in hybridization intensity (P< = 0.01) under the effect of rice cultivars (Nipponbare vs. 93-11), MV treatment, and the interaction between the two factors. In the Venn diagram, the intersection of all three groups included 9,318 probes and is highlighted in red. These probes were significantly regulated by cultivar, treatment, and their interaction. We further mapped these probes to probe sets and there were 1,062 probe sets which include at least 3 probes belonging to the intersection. Detailed information is listed in Table S1, including raw intensities, intersection probe number, p-value of ANOVA test, and the FDR q-value for each p-value of each probe set. We called the 1,062 probe sets Core Intersectional Probesets (CIPs). Figure 2B shows that most CIPs preferentially expressed in Nipponbare were also induced by MV treatment (645 up-regulated by MV and 101 down-regulated by MV). In contrast, most CIPs preferentially expressed in 93-11 were also reduced by MV treatment (223 down-regulated by MV and 93 up-regulated by MV). We propose that transcripts represented by these 1,062 probe sets may contribute to the significant phenotypic divergence between 93-11 and Nipponbare under MV treatment.

### Functional Analysis of the CIPs Shown Transcript Variance with Cultivars and MV Treatment

GO (Gene Ontology) terms are widely applied to understand the biological significance of microarray differential gene expression data[45]. We employed GO category enrichment analysis on these 1,062 CIPs using EasyGO [46]. Table 1 includes the GO categories (biological process, molecular function, and cellular component) and enrichment analysis results for the 1,062 probe sets. There were a total of 615 probe sets with GO term annotations in biological processes and the most significant enriched GO term was “response to toxin” (GO: 0009636, FDR 2.89E-26). There were other GO terms enriched, such as “defense response” (GO: 0009814, FDR 1.12E-07), “response to oxidative stress” (GO: 0006979, FDR 1.02E-03), and “chalcone biosynthetic process” (GO: 0009715, FDR 1.34E-07), as well as some GO terms related to hormone stimulus (ABA, JA, and ethylene).

**Table 1:** Gene Ontology Analysis of 1062 CIPs.

GO ID	GO Term	Query item	Background item	FDR p-value
**Biological Process**		**615**	**21427**	
GO:0009636	response to toxin	28	130	2.89E-26
GO:0019748	secondary metabolic process	70	1083	2.09E-09
GO:0009814	defense response, incompatible interaction	22	202	1.12E-07
GO:0009715	chalcone biosynthetic process	6	17	1.34E-07
GO:0010817	regulation of hormone levels	18	155	7.13E-07
GO:0009688	abscisic acid biosynthetic process	6	21	3.82E-06
GO:0009861	jasmonic acid and ethylene-dependent systemic resistance	13	102	1.62E-05
GO:0009723	response to ethylene stimulus	12	93	3.97E-05
GO:0006979	response to oxidative stress	19	236	1.02E-03
**Molecular Function**		**722**	**27148**	
GO:0004364	glutathione transferase activity	31	179	1.08E-24
GO:0004601	peroxidase activity	65	1408	3.38E-03
GO:0019825	oxygen binding	26	417	7.48E-03
**Cellular Component**		**764**	**29111**	
GO:0012505	endomembrane system	201	5277	1.03E-06
GO:0043234	protein complex	64	3996	1.69E-03
GO:0009507	chloroplast	98	5332	6.12E-03

As for molecular function, there were 722 probe sets with GO term annotations and the significant GO terms were “glutathione transferase activity” (GO: 0004364, FDR 1.08E-24), “peroxidase activity” (GO: 0004601), and “oxygen binding” (GO: 0019825, majority P450 proteins). Of particular interest, glutathione S-transferase (GST) was significantly enriched among the 1,062 CIPs. There are 31 GST probe sets (corresponding to 28 GST genes) exhibiting significant expression variance between Napponbare and 93-11 under MV treatment.

764 probe sets with GO term annotations to cellular components demonstrated significant enrichment. 201 probe sets corresponded to “endomembrane system” (GO: 0012505), and the other two terms, “protein complex” and “chloroplast” were also enriched.

**Figure 3:**
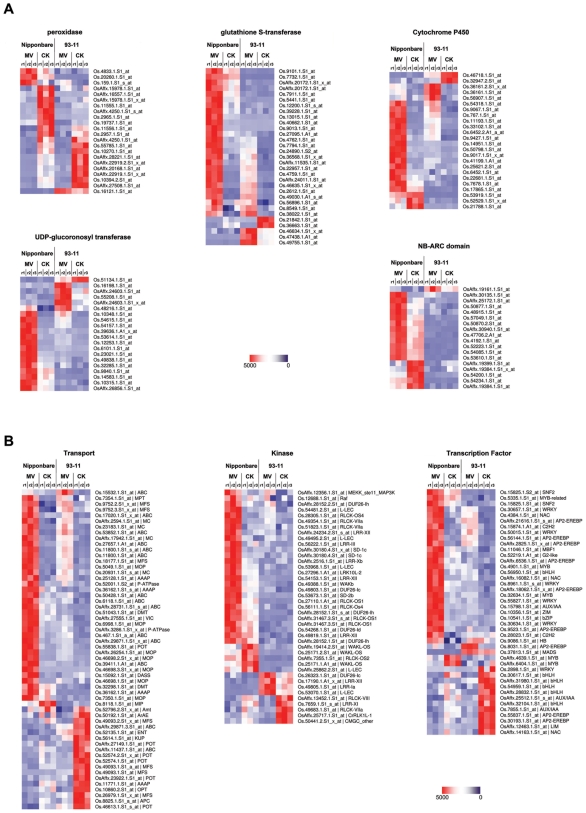
The expression pattern of the Core Intersectional Probesets (CIPs) in highlighted gene superfamilies. **A.** The probe sets were derived from peroxidases, GSTs, UGTs, P450s, UDP-glucoronosyl transferase, and NB-ARC domain-containing proteins. **B.** The probe sets were from transporters, kinases, and TFs. The color scale (representing expression level) is shown at bottom-right. Red indicates a higher expression level, whereas blue indicates a lower expression level. White indicates the average expression level.

We analyzed further the expression levels of these GO enriched genes as well as genes of several important superfamilies. The cluster results were displayed with Treeview as shown in Figure 3. In Figure 3A, we highlight major detoxification enzymes, such as GSTs, P450s, peroxidases, and UDP-glucoronosyl transferase. Other than peroxidases, the majority of genes encoding these enzymes were up-regulated under MV treatment and most of them were preferentially expressed in Nipponbare rather than in 93-11. 18 of 22 probe sets corresponding to peroxidases exhibited down-regulation under MV treatment, of which half of them were highly expressed in Nipponbare and the other half were highly expressed in 93-11. Within 1062 CIPs, a large number of corresponding genes fall into large superfamiles. In Figure 3B, we show the cluster results for transporters, kinases, and transcription factors (TFs) represented by the CIPs. Interestingly, the transporters and TFs preferentially expressed in Nipponbare were up-regulated under MV treatment, whereas those preferentially expressed in 93-11 were mainly down-regulated under MV treatment.

### Real-Time RT-PCR Validation of Microarray Data

Based on enriched GO terms and important superfamilies in 1,062 CIPs, we selected some candidate genes for real-time RT-PCR analysis to confirm our microarray analysis, such as glutathione S-transferase genes, cytochrome P450s, NBS-LRR, and chalcone biosynthetic related genes, etc. In order to do real-time RT-PCR validation, additional biological samples were collected from Nipponbare and 93-11 treated by MV for 24 h or mock treated. The fold-change in expression for selected genes under each condition is listed in Table 2. We also ran a two-way ANOVA test based on replicates. Real-time RT-PCR for the majority of the tested genes confirmed the microarray results.

**Table 2:** Real-time RT-PCR for selected genes in 10 µM MV treated Nipponbare and 93-11 seedlings.

Locus ID	93-11		Nipponbare		P-value		
	Mock 24 h	MV 24 h	Mock 24 h	MV 24 h	Treatment	Cultivar	T x C
LOC_Os10g38340	1.00±0.000	12.06±0.455	36.84±8.311	648.82±195.943	2.10E-03	5.54E-04	6.43E-01
LOC_Os10g38360	1.00±0.000	7.64±0.176	3.02±0.121	88.85±4.087	2.71E-06	6.01E-05	2.00E-02
LOC_Os10g38470(P2)	1.00±0.000	1.00±0.000	87.63±24.417	470.05±138.083	6.79E-02	9.48E-07	6.79E-02
LOC_Os10g38470(P1)	362.46±65.889	546.85±44.836	2.21±0.000	1.00±0.000	2.61E-01	3.61E-10	5.02E-03
LOC_Os10g38189	15.14±2.868	20.82±0.994	1.00±0.000	2.75±0.018	2.78E-02	1.18E-05	2.00E-01
LOC_Os07g23570	4.51±0.021	9.89±0.481	1.00±0.000	53.94±1.409	3.35E-06	6.65E-01	6.37E-05
LOC_Os01g43700	1.64±0.019	1.69±0.019	1.00±0.000	2.51±0.005	1.08E-02	7.36E-01	1.48E-02
LOC_Os03g55240	1.00±0.000	3.71±0.293	3.18±0.038	51.45±1.754	3.69E-02	4.64E-02	3.30E-01
LOC_Os11g10550	2.24±0.093	1.00±0.000	1854.31±237.719	2771.91±561.593	8.21E-01	5.68E-09	1.44E-01
LOC_Os07g33690	1.00±0.000	1.00±0.000	452.99±73.631	582.72±0.000	4.89E-01	5.97E-07	4.89E-01
LOC_Os03g62480	1.09±0.003	1.00±0.000	190.02±9.734	1520.15±75.004	7.05E-02	1.11E-06	5.29E-02
LOC_Os06g18140	2.02±0.000	6.27±0.097	1.00±0.000	29.11±1.136	8.56E-08	6.14E-04	7.62E-05
LOC_Os08g39840	1.00±0.000	3.9±0.008	10.73±0.422	65.8±3.003	1.99E-06	4.32E-08	1.22E-01
LOC_Os01g71860	1.00±0.000	1.01±0.000	2.41±0.034	26.54±2.146	2.44E-04	4.87E-06	2.60E-04
LOC_Os05g12240	134.05±3.677	2.37±0.006	14.69±0.449	1.00±0.000	2.54E-09	1.16E-06	4.56E-04
LOC_Os02g56700	1.00±0.000	8.53±0.258	68.59±6.94	1152.06±83.691	1.95E-04	2.30E-06	4.04E-01

glutathione S-transferase in chromosome 10: LOC_Os10g38360, LOC_Os10g38340, LOC_Os10g38610, LOC_Os10g38189, and LOC_Os10g38470 (two primer pairs designed for LOC_Os10g38470, P1 represented 93-11 3′-UTR region, P2 represented Nipponbare 3′-UTR region); LOC_Os07g23570–cytochrome P450 709C9; LOC_Os01g43700–cytochrome P450 72A17; LOC_Os03g55240–cytochrome P450 81A6; LOC_Os11g10550–NBS-LRR disease resistance protein; LOC_Os07g33690–NBS-LRR type disease resistance protein Hom-F; LOC_Os03g62480–anthocyanidin 5,3-O-glucosyltransferase; LOC_Os06g18140–indole-3-acetate beta-glucosyltransferase; LOC_Os08g39840–lipoxygenase 7, chloroplast precursor; LOC_Os01g71860–beta 1,3-glucanase (BGL); LOC_Os05g12240–Chalcone synthase 8 (CHS8); LOC_Os02g56700–Cinnamoyl-CoA reductase (OsCCR10).

Several putative glutathione S-transferase genes, such as LOC_Os10g38340 and LOC_Os10g38360, exhibited significantly higher expression in Nipponbare and were up-regulated under MV stress. Another GST gene, LOC_Os10g38189, was preferentially expressed in 93-11 and slightly up-regulated by MV treatment. From our original expression analysis, we discovered that one GST gene (LOC_Os10g38470) matched two probe sets in the Affymetrix rice GeneChip and the two probe sets revealed contradictory expression profiles. One set showed increased expression in 93-11 and the other demonstrated higher expression in Nipponbare, both of which were induced by MV treatment. In order to further investigate the regulation of this gene in rice cultivars, two pairs of primers specific for this gene were designed for real-time RT-PCR analysis based on a sequence comparison between *indica* and *japonica* (shown in Appendix S1). We discovered that there is sequence variation in the 3′-UTR region of LOC_Os10g38470 between Nipponbare and 93-11. From our real-time RT-PCR analysis, LOC_Os10g38470-P1 showed significantly higher expression in 93-11 and was not expressed in Nipponbare, whereas LOC_Os10g38470-P2 was not expressed in 93-11 but abundantly expressed in Nipponbare. Thus there do exist gaps in the 3′-UTR regions between two subspecies. Both LOC_Os10g38470-P1 and LOC_Os10g38470-P2 were up-regulated by MV treatment.

Cytochrome P450s are involved in the detoxification of xenobiotic chemicals such as herbicides [47], [48]. CYP709C9 (LOC_Os07g23570) is a possible ortholog for wheat P450 genes CYP709C1 and CYP709C3v2, which were suggested to be involved in wheat defense to pathogens [47], [48]. From our microarray and real-time RT-PCR results, CYP709C9 was up-regulated under MV treatment. Compared to 93-11, CYP709C9 showed significantly higher expression in Nipponbare seedlings after 24 hours of MV treatment. CYP72A17 (LOC_Os01g43700) and CYP81A6 (LOC_Os03g55240) were also up-regulated under MV treatment and more abundantly expressed in Nipponbare seedlings.

NBS-LRR family genes encode plant disease resistance (R) proteins with a nucleotide-binding site (NBS), a series of leucine-rich repeats (LRRs) [49]. Two NBS-LRR family genes (LOC_Os11g10550 and LOC_Os07g33690) were expressed in Nipponbare and also up-regulated under MV treatment, but silent in 93-11. The beta 1,3-glucanase (BGL, LOC_Os01g71860) was significantly up-regulated under MV treatment in Nipponbare but showed very low expression in 93-11. Two glucoronosyl transferase genes (LOC_Os06g18140 and LOC_Os03g62480) showed higher expression in Nipponbare and were up-regulated under MV treatment. The jasmonate biosynthesis related gene lipoxygenase 7 (LOC_Os08g39840) was up-regulated in Nipponbare after MV treatment, but undetectable in the 93-11 variety.

During the GO term analysis, we also found that chalcone biosynthetic process was enriched. Several related genes were selected for real-time RT-PCR. One cinnamoyl CoA reductase (CCR) gene, OsCCR10 (LOC_Os02g56700) was up-regulated under MV treatment. Compared to Nipponbare, OsCCR10 showed significantly lower expression in 93-11 under MV treatment. The chalcone synthase gene (LOC_Os05g12240, CHS8) was significantly down-regulated under MV treatment, exhibiting higher levels of expression in 93-11 than in Nipponbare.

### InDel Analysis of CIPs between *Indica* and *Japanica* Varieties

The genes belonging to CIPs may have sequence-level polymorphisms such as small insertions or deletions (InDels) between *indica* and *japonica* varieties. The sequence variations between 93-11 contigs and TIGR pseudomolecules (Nipponbare) were obtained from TIGR Rice Database. Combining gene expression variations (1,062 CIPs) and InDel result togethers, 565 genes were identified that demonstrated both InDel variations and differential expression between rice *indica* and *japonica* subspecies. The locus ID and annotation of these genes are listed in a supplemental table (Table S1).

**Figure 4:**
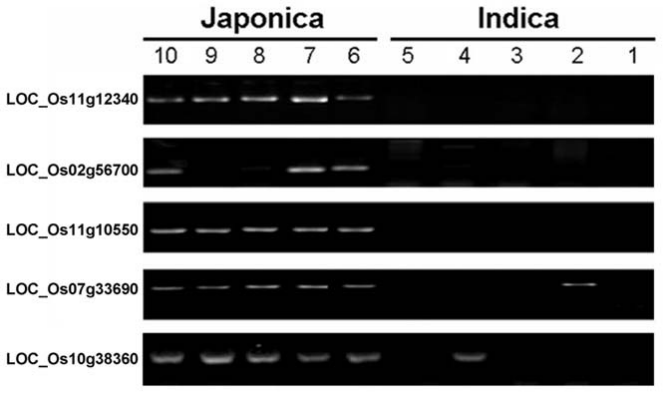
InDel analysis of selected genes between *indica* and *japonica* varieties. Lanes 1–5: PCR products obtained using genomic DNA of *indica* varieties: 93-11, 37760, 03A-11, TP34, and ShuiYuan349, respectively. Lanes 6–10: PCR products obtained using genomic DNA of *japonica* varieties: Nipponbare, IR66746-76-3-2, REIMEL, ShangZhou10, and YunFeng7, respectively. LOC_Os11g12340–disease resistance protein RPM1; LOC_Os02g56700–Cinnamoyl-CoA reductase (OsCCR10); LOC_Os11g10550–NBS-LRR disease resistance protein; LOC_Os07g33690–NBS-LRR type disease resistance protein Hom-F; LOC_Os10g38360–glutathione S-transferase.

We performed a genotyping analysis of *indica* and *japonica* varieties on five selected genes from the CIP set, including one GST gene, two NB-LRR genes, OsCCR10, a disease resistance protein RPM1. Five *indica* cultivars (93-11, 37760, 03A-11, TP34, and ShuiYuan349) and five *japonica* cultivars (Nipponbare, IR66746-76-3-2, REIMEL, ShangZhou10, and YunFeng7) were selected. Gene-specific primers for selected genes were designed based on the 93-11 contigs and TIGR pseudomolecules (Nipponbare) comparison track in the genome browser (http://rice.plantbiology.msu.edu/cgi-bin/gbrowse/rice/) of TIGR Rice Database. PCR primers were designed such that amplicons would not be obtained if InDels were present in the target gene. PCR on genomic DNA was conducted for genotyping the candidate gene variance between *indica* and *japonica* varieties and the results are shown in Figure 4. All 5 genes were amplified from Nipponbare DNA, but not from 93-11, which validated the *in silico* InDels analysis of 93-11 and Nipponbare. As to other cultivars, several minor variants were observed. LOC_Os11g10550 (NBS-LRR), and LOC_Os11g12340 (RPM1) were amplified from all 5 *japonica* cultivars but none of the 5 *indica* cultivars, which perfectly agreed with our predictions. PCR products amplified from LOC_Os02g56700 (OsCCR10) were obtained in 3 of the 5 *japonica* cultivars and from none of the 5 *indica* cultivars. LOC_Os07g33690 (NBS-LRR type disease resistance protein Hom-F) and LOC_Os10g38360 (glutathione S-transferase) were amplified in all 5 *japonica* cultivars and one of the 5 *indica* cultivars. These results indicate that these 5 genes harbor InDel polymorphisms in most *indica* and *japonica* varieties, and our InDel primer pair design may be used for identification of *indica* and *japonica* subspecies.

## Discussion

Genetic variation among different rice varieties may lead to alternative gene expression, which may influence phenotypic variation. Nipponbare seedlings showed strong oxidative stress resistance to methyl viologen (MV) treatment compared to 93-11. In order to elucidate the molecular mechanisms of phenotypic divergence in oxidative stress response observed between Nipponbare (*japonica* variety) and 93-11 (*indica* variety), we conducted a microarray analysis and real-time RT-PCR validation to identify genes differentially expressed in response to MV treatment in the two rice cultivars. We identified Core Intersectional Probeset, and further employed multiple enrichment analysis to understand the genetic basis of oxidative stress resistance.

GO term enrichment analysis provided insight into the CIPs involved in MV treatment. The GO terms “response to toxin”, “defense response” and “response to oxidative stress” were significantly enriched in the 1062 CIPs. Glutathine S-transferase genes (GSTs, EC 2.5.1.18) were significantly enriched in GO molecular function categories. GSTs are soluble proteins with typical molecular masses of around 50 kDa, each composed of two polypeptide subunits. GST proteins have evolved by gene duplication and are encoded by a large and diverse gene family in plants to perform a range of functional roles. They are potential regulators of programmed cell death and involved in detoxification of herbicides, reduction of organic hydroperoxides formed during oxidative stress. GSTs also act as components of ultraviolet-inducible cell signaling pathways and bind flavonoid natural products in the cytosol prior to their deposition in the vacuole [35], [50], [51].

**Figure 5:**
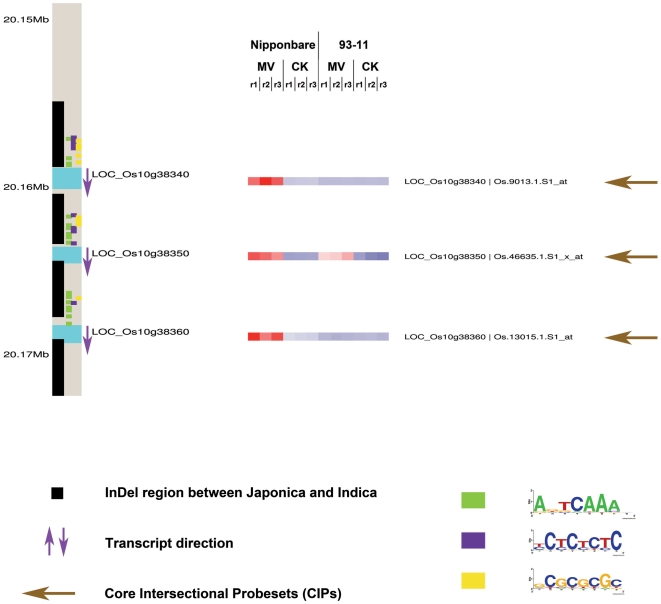
The sketch map for GSTs in the 20.15 to 20.17 Mb region of chromosome 10. This map highlights three glutathione S-transferase genes within the CIP list, LOC_Os10g38340, LOC_Os10g38350, and LOC_Os10g38360, including their locations, transcript direction, the positions of indel regions different between *japonica* and *indica*, motifs in promoter regions, and expression patterns.

There are a total of 28 GST genes among the 1062 CIPs, 21 of which have gaps in genomic regions: 64.29% with at least one InDel in the upstream region, 32.14% with InDel in the coding region, and 10.71% with InDel in the downstream region. Especially, 15 GST genes with CIPs locate in rice chromosome 10, and 12 of them exist gaps between 93-11 and Nipponbare. We made a sketch map for GSTs in chromosome 10 shown in Figure S3. Some GST gene clusters were identified in Chromosome 10, such as LOC_Os10g38340, LOC_Os10g38350 and LOC_Os10g38360 (Figure 5). These genes not only showed variations in expression levels, but also harbor InDels in the promoter regions of one rice cultivar. We applied MDscan [52] to search for common motifs in the promoter regions of these specific GST genes and found that several motifs are significantly enriched, such as GCCGCGGC (CGCGBOXAT, possibly involved in Ca^++^/calmodulin binding) [53] AGTCAAAC (W-box, WRKY binding sites) [54], and an unknown motif (TCTCTCTC). Because a significant percentage of GST genes in the CIP set harbor sequence gaps in their promoter regions, we propose that some of these InDel regions may be referred to as oxidative stress related eQTL hotspots where numerous GST transcript polymorphisms link to the same region.

**Figure 6:**
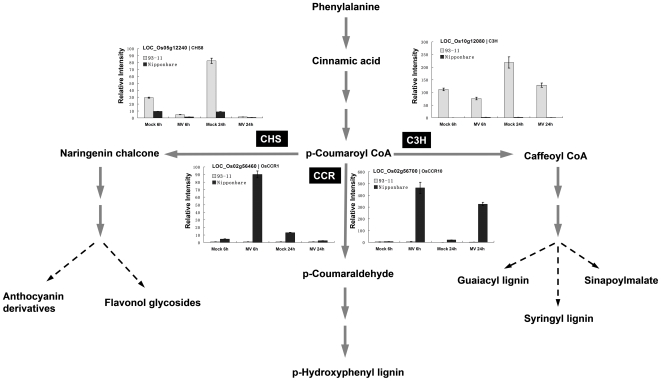
The expression pattern of selected phenylpropanoid pathway related genes in 10 µM MV treated seedling plants from rice *japonica* variety (Nipponbare) and *indica* variety (93-11). The black bars indicate relative expression (fold-change by real-time PCR) of the gene in Nipponbare samples. The grey bars indicate relative expression (fold-change by real-time PCR) of the gene in 93-11 samples. Error bars represent the standard error of three replicates. LOC_Os05g12240–Chalcone synthase 8 (CHS8); LOC_Os10g12080–Cytochrome P450 98A15p (C3H); LOC_Os02g56460–Cinnamoyl-CoA reductase (OsCCR1); LOC_Os02g56700–Cinnamoyl-CoA reductase (OsCCR10).

The GO term “chalcone biosynthetic process” was also significantly enriched. We propose that secondary metabolism related genes may also contribute to the different leaf senescence levels observed between the two subspecies 93-11 and Nipponbare. Among the identified CIPs, there are a large number of lignin biosynthesis pathway and flavonoid biosynthesis pathway related genes, which were reported to be involved in programmed cell death, including the cinnamoyl CoA reductase gene CCR1. CCR is a key enzyme in lignin biosynthesis via the phenylpropanoid pathway, and lignin and lignin-related compounds are induced by infection with pathogens [55], [56], [57]. Figure 6 showed a simple phenylpropanoid pathway together with the real-time RT-PCR results of some key genes. The p-Coumaroyl CoA is produced though a series of reactions beginning with phenylalanine, and it is the common substrate for different lignins, anthosyanin derivatives, and flavonol glycosides. One CCR gene, OsCCR10 (LOC_Os02g56700, AP005303, AK119257) assigned as a CIP, was up-regulated under MV treatment. Compared to Nipponbare, OsCCR10 was significantly less expressed in 93-11 under MV treatment. We also determined the relative expression levels of OsCCR1 genes, which all exhibited similar expression patterns as OsCCR10 after 6 h MV treatment. Furthermore, another lignin biosynthesis pathway related gene, CYP98A15p, was down-regulated under MV treatment in 93-11, and was only weakly expressed in Nipponbare. CYP98A15p is a P450 gene and possible ortholog of *Arabidopsis* C3H (p-Coumarate 3-hydroxylase) in the rice lignin biosynthesis pathway [56]. During MV oxidative stress, the lignin synthesis repression in 93-11 may drive metabolic flux into flavonoids through chalcone synthase activity [58]. CHS8 gene (LOC_Os05g12240) was selected for comparative gene expression analysis between the two cultivars. From the real-time RT-PCR shown in the Figure 6, CHS8 was significantly down-regulated under MV treatment, and exhibited higher expression in 93-11 than in Nipponbare. Therefore, the differential expression of lignin biosynthesis genes may lead to different metabolite flux between Nipponbare and 93-11.

In summary, we developed a new strategy to study the molecular mechanisms underlying the significant divergence of oxidative stress response between *japonica* and *indica* subspecies. In so doing, we successfully identified a group of potential important marker genes of *japonica* and *indica* subspecies. The new strategy will benefit the further study of the molecular basis and evolution of transcriptional regulatory networks underlying the phenotypic variation between rice subspecies *japonica* and *indica*. However, we considered that our methodology will yield some false positive and false negative results due to limited GO information of the rice genome. Furthermore, the microarray platform has limitations due to cross-hybridization and relatively lower dynamic range. Future analysis using next generation deep-sequencing analysis of expression variation and further individual gene analysis using reverse genetics approaches will be necessary to dissect gene functions during rice oxidative stress.

## Materials and Methods

### Plant Materials

#### For DNA isolation

Fresh leaves from different cultivars (*indica* cultivars 93-11, 37760, 03A-11, TP34, and ShuiYuan349; *japonica* cultivars Nipponbare, IR66746-76-3-2, REIMEL, ShangZhou10, and YunFeng7) were harvested from rice plants grown under natural conditions.

#### For RNA isolation

Seeds of two rice cultivars (93-11 and Nipponbare) were surface-sterilized in 5% (w/v) sodium hypochlorite for 20 min and then washed in distilled water three or four times, then germinated in water for 2 days at room temperature and 1 day at 37°C. The seedlings were transferred to water-saturated Whatman paper and grown in a greenhouse (28°C day/25°C night, 12 h light/12 h dark, and 83% relative humidity). After about 4 days, phenotypically normal seedlings of Nipponbare and 93-11 were chosen and placed into water containing 10 μM methyl viologen (MV) to be incubated, whereas water was used as a mock treatment. Seedlings were harvested after 6 h and 24 h MV treatment. Control plants receiving only mock treatment were also harvested at the same time.

#### For phenotype evaluation

To identify the phenotypic divergence between 93-11 and Nipponbare under methyl viologen (MV) treatment, we transferred the one-week-old seedlings to water (mock treatment) or solution containing different concentration of MV. After 5 days, 7 days, and 10 days incubation in solution with 10 μM, 15 μM, and 20 μM MV, the phenotype of 93-11 and Nipponbare was investigated and recorded.

### RNA Isolation and Real-Time RT-PCR

All seedling samples from varieties 93-11 and Nipponbare were homogenized in liquid nitrogen before isolation of RNA. Total RNA was isolated using TRIZOL® reagent (Invitrogen, CA, USA) and purified using Qiagen RNeasy columns (Qiagen, Hilden, Germany). Reverse transcription was performed using Moloney murine leukemia virus (M-MLV; Invitrogen). We heated 10 µl samples containing 2 µg of total RNA, and 20 pmol of random hexamers (Invitrogen) at 70°C for 2 min to denature the RNA, and then chilled the samples on ice for 2 min. We added reaction buffer and M-MLV to a total volume of 20 µl containing 500 µM dNTPs, 50 mM Tris–HCl (pH 8.3), 75 mM KCl, 3 mM MgCl_2_, 5 mM dithiothreitol, 200 units of M-MLV, and 20 pmol random hexamers. The samples were then heated at 42°C for 1.5 h. The cDNA samples were diluted to 8 ng/µl for real-time RT-PCR analysis.

For real-time RT-PCR, triplicate assays were performed on 1 µl of each cDNA dilution using the SYBR Green Master Mix (Applied Biosystems, PN 4309155) with an ABI 7900 sequence detection system according to the manufacture's protocol (Applied Biosystems). The gene-specific primers were designed by using PRIMER3 (http://frodo.wi.mit.edu/primer3/input.htm). The amplification of 18S rRNA was used as an internal control to normalize all data (forward primer, 5′-CGGCTACCACATCCAAGGAA-3′; reverse primer, 5′- TGTCACTACCTCCCCGTGTCA-3′). Gene-specific primers are listed in Table S2. The relative quantification method (DDCT) was used to evaluate quantitative variation between replicates examined.

### Affymetrix GeneChip Analysis

For each sample, 8 µg of total RNA was used for making biotin-labeled cRNA targets, cDNA and cRNA synthesis, cRNA fragmentation, hybridization, washing and staining, and scanning, followed the GeneChip Standard Protocol (Eukaryotic Target Preparation). In this experiment, Poly-A RNA Control Kit and the One-Cycle cDNA Synthesis kit were applied. Affymetrix GCOS software was used to do data normalization and comparative analysis.

The probe level polymorphisms were identified by bowtie (version 0.10.1 from http://bowtie-bio.sourceforge.net/index.shtml) with the rice *japonica* genome sequence (TIGR 5) and rice *indica* genome sequence (RISe, http://rice.genomics.org.cn/rice/index2.jsp). Probe-level two-way ANOVA analysis was calculated by Partek Genomics Suite (Version 6.3) with quantile normalization on all CEL files. The FDR q-value of each p-value was also calculated by Partek. The signal intensity for each probe set on the GeneChip microarray was extracted by Affymetrix GCOS software and the TGT (target mean value) was scaled as 500 for each chip.

In order to map the probe set ID to the locus ID in the rice genome, the consensus sequence of each probe set was compared by BLAST (Basic Local Alignment and Search Tool) against the TIGR Rice Genome version 5. The cut-off e-value was set as 1e-20. Within the 57,195 designed probe sets in the Affymetrix rice genome array, there are 52,697 probe sets mapped to rice genes in TIGR rice pseudomolecules.

The promoter sequences were extracted from MSU Rice Genome Annotation Website (http://rice.plantbiology.msu.edu/) and ELEMENT (http://element.cgrb.oregonstate.edu/). MDscan (http://ai.stanford.edu/~xsliu/MDscan/) [52] and Place (http://www.dna.affrc.go.jp/PLACE/) [60] were used for motif search and cis-element identification.

### DNA Extraction and PCR Analysis

Fresh leaves were collected and ground in liquid nitrogen. DNA was extracted from the ground tissues by the CTAB method [59]. Primer sets (listed in Table S3) were designed according to the genome sequence of Nipponbare. A 25 μl reaction mixture was composed of 30 ng of genomic DNA, 10 mM Tris–HCl (pH 9.0), 50 mM MgCl_2_, 0.1% (v/v) Triton X-100, 200 µM dNTPs, 2 µM each primer, and one unit of Taq DNA polymerase (Promega). An initial denaturing step was for 5 min at 94°C, followed by 35 cycles of 30 sec at 94°C, 45 sec at 58°C, 1 min 30 sec at 72°C, with a final extension for 10 min at 72°C. PCR products were separated by electrophoresis in a 1.2% (w/v) agarose gel.

## Supporting Information

Appendix S1 The location of two primer pairs designed for detection of LOC_Os10g38470 expression by real time RT-PCR.
(0.04 MB DOC)

Figure S1 MV concentrations and time effects on rice japonica variety (Nipponbare) and indica variety (93-11) seedlings. Nipponbare (left) and 93-11 (right) sprouted seeds were mock-treated (water) or treated with a gradient concentration of MV (10 μM, 15 μM, and 20 μM) for 5 days, 7 days, and 10 days.
(0.52 MB TIF)

Figure S2 Pair-wise scatter plots for the raw probe set intensity data across all arrays.
(5.84 MB JPG)

Figure S3 Sketch map of GSTs encoded in an interval from 20.10 Mb to 20.32 Mb on chromosome 10.
(4.35 MB JPG)

Table S1 1062 CIPs with differential expression between indica (93-11) and japonica (Nipponbare) under MV treatment.
(0.24 MB PDF)

Table S2 The primer sequences of selected genes for real-time RT-PCR analysis.
(0.05 MB DOC)

Table S3 The primer sequence for InDel detection.
(0.03 MB DOC)
