# Supplementary material for: Gene Expression Profiles Deciphering Rice Phenotypic Variation between Nipponbare (Japonica) and 93-11 (Indica) during Oxidative Stress
Source: PLoS One. 2010 Jan 8;5(1):e8632. doi: 10.1371/journal.pone.0008632 (PMC2799674; doi:10.1371/journal.pone.0008632)
Supplement: Appendix S1 — The location of two primer pairs designed for detection of LOC_Os10g38470 expression by real time RT-PCR. (0.04 MB DOC) [file pone.0008632.s001.doc]

Appendix 1. The location of two primer pairs designed for detection of LOC_Os10g38470 expression by real time RT-PCR

LOC_Os10g38470-P1, sequences labeled in blue, located in 3’-UTR region, only perfect matched 9311 genome sequence; LOC_Os10g38470-P2, sequences labeled in red, located in 3’-UTR region, only perfect matched Nipponbare genome sequence. The yellow background sequences showed the intron region of LOC_Os10g38470.

LOC_Os10g38470: the genome sequence of LOC_Os10g38470

Contig005622: 9311 contig, including the genome region of LOC_Os10g38470 in 9311

LOC_Os10g38470 1 AACACCAACCAGACAGAACAGAGGACACCGCTCACAAGCAAATACAGAGT 50

||||||||||||||||||||||||||||||||||||||||||||||||||

Contig005622 15049 AACACCAACCAGACAGAACAGAGGACACCGCTCACAAGCAAATACAGAGT 15098

LOC_Os10g38470 51 CGAGAGTGAGACTTGAGGCAGAGGCCATGGCCGGAGGAGGAGACGAGCTG 100

||||||||||||||||||||||||||||||||||||||||||||||||||

Contig005622 15099 CGAGAGTGAGACTTGAGGCAGAGGCCATGGCCGGAGGAGGAGACGAGCTG 15148

LOC_Os10g38470 101 AAGCTGCTGGGCATGTGGGCGAGCCCGTACGTTCTGCGAGTGAAGTTCGC 150

|||||||||||||||||||||||||||||||||||||||||||||.||||

Contig005622 15149 AAGCTGCTGGGCATGTGGGCGAGCCCGTACGTTCTGCGAGTGAAGCTCGC 15198

LOC_Os10g38470 151 GCTCAGTCTCAAGGGCCTCAGCTACGAGTACGTCGAGGAGGATCTCATGA 200

|||||||||||||||||||||||||||.||||||||||||||||||||||

Contig005622 15199 GCTCAGTCTCAAGGGCCTCAGCTACGACTACGTCGAGGAGGATCTCATGA 15248

LOC_Os10g38470 201 ACAAGAGCGACCTCCTCCTCAGCTCCAACCCGGTGAACAAGAAGGTGCCC 250

||||||||||||||||||||||||||||||||||||||||||||||||||

Contig005622 15249 ACAAGAGCGACCTCCTCCTCAGCTCCAACCCGGTGAACAAGAAGGTGCCC 15298

LOC_Os10g38470 251 GTGCTCATCCACAACGGCAAGCCCGTCTGCGAGTCGCAGGTCATCCTCCA 300

||||||||||||||||||||||||:||||||||||||||:|||||||.||

Contig005622 15299 GTGCTCATCCACAACGGCAAGCCCATCTGCGAGTCGCAGATCATCCTGCA 15348

LOC_Os10g38470 301 GTACCTCGACGAGGCGTTCCCCGGCGCCGGCGCCACCCTCCTCCCCGCCG 350

||||||||||||||||||||||||||||||||||||||||||||||||||

Contig005622 15349 GTACCTCGACGAGGCGTTCCCCGGCGCCGGCGCCACCCTCCTCCCCGCCG 15398

LOC_Os10g38470 351 ACCCCCACGAACGCGCCGTCGCTCGCTTCTGGGCCGCCTTCAACGACGAC 400

||||||||||||||||||||||||||||||||||||||||||||||||||

Contig005622 15399 ACCCCCACGAACGCGCCGTCGCTCGCTTCTGGGCCGCCTTCAACGACGAC 15448

LOC_Os10g38470 401 ACGGTACGTCGGTACCATGTCACCTCACATCGCGATCATCCGCATCGTCT 450

||||||.|..:| |||||.|||||||||||||||||

Contig005622 15449 ACGGTATGGTAG---------------CATCGAGATCATCCGCATCGTCT 15483

LOC_Os10g38470 451 CCGACTCTTCCGTTTTTGATTCAATTTGATTTGTTCGCAGCTGGTGGAGG 500

|||||||||||| ||||||||||||||||||||||||||||||.|||.||

Contig005622 15484 CCGACTCTTCCG-TTTTGATTCAATTTGATTTGTTCGCAGCTGTTGGCGG 15532

LOC_Os10g38470 501 CGTCGCAGGCGGCGTCGTGGGGGAAGACGGAGGAGGAGAGGGCGGAGGGG 550

||||||||||||||||||||||||||||||||||||||||||||||||||

Contig005622 15533 CGTCGCAGGCGGCGTCGTGGGGGAAGACGGAGGAGGAGAGGGCGGAGGGG 15582

LOC_Os10g38470 551 GAGAAGAAGGTGGTGGAGGCGCTGGAGAAGATGGAGGTCGGGCTGAGGGA 600

||||||||||||||||||||||||||||:|||||||||||||||||||||

Contig005622 15583 GAGAAGAAGGTGGTGGAGGCGCTGGAGAGGATGGAGGTCGGGCTGAGGGA 15632

LOC_Os10g38470 601 GTGCTCCAAGGGGAAGCCCTTCTTCGGCGGCGACACCGTCGGGTACCTCG 650

||||||||||||||||||||||||||||||||||||||||||.|||||||

Contig005622 15633 GTGCTCCAAGGGGAAGCCCTTCTTCGGCGGCGACACCGTCGGCTACCTCG 15682

LOC_Os10g38470 651 ACGTCGTGCTCGGCGGCTTCCTCGCGTGGGTGCGCGCCACCGACGTGATG 700

||||||||||||||||||||||||||||||||||||||||||||||||||

Contig005622 15683 ACGTCGTGCTCGGCGGCTTCCTCGCGTGGGTGCGCGCCACCGACGTGATG 15732

LOC_Os10g38470 701 CGCGGCGTCAAGAGGTTCGACCCGGCCACCACCCCGCTGCTGGCGGCGTG 750

||.|||||||||.|||||||||||||||||||||||||||||||||||||

Contig005622 15733 CGTGGCGTCAAGCGGTTCGACCCGGCCACCACCCCGCTGCTGGCGGCGTG 15782

LOC_Os10g38470 751 GGCCGAGCGCTTCGTGGAGCTGGACGCCGCCAAGGCCGTCATGCCGGACA 800

|||||||||||||||.||||||||||||||||||||||||||||||||||

Contig005622 15783 GGCCGAGCGCTTCGTCGAGCTGGACGCCGCCAAGGCCGTCATGCCGGACA 15832

LOC_Os10g38470 801 TGGACAAGATGATCGAGTTCGGCAAGGTGCTGCAGGCTCGCGCCGCCGCC 850

|||||||||||||||||||||||||||||||||||||||| |||||||

Contig005622 15833 TGGACAAGATGATCGAGTTCGGCAAGGTGCTGCAGGCTCG---CGCCGCC 15879

LOC_Os10g38470 851 GCCACCAACTGAAGTGACAG------------------------------ 870

||||||||||||||||||||

Contig005622 15880 GCCACCAACTGAAGTGACAGATGACACTGGAATCTTTGCGGTGCAGCGCA 15929

LOC_Os10g38470 871 -------TTGACTTGTCGTGTCATGCCGAAGACGAACGGATGTTGGGATT 913

|||||||||||||||||||||||||||||||.|:||| |||||

Contig005622 15930 CAAGTGATTGACTTGTCGTGTCATGCCGAAGACGAACGCAAGTT-GGATT 15978

LOC_Os10g38470 914 GTTTC----------C-ATCTGTTTCACAGTCGAGTGTCGTGTACGTTAC 952

||||| | |||||||||||||||||||||||||||||||||

Contig005622 15979 GTTTCTTGGTCTGTTCGATCTGTTTCACAGTCGAGTGTCGTGTACGTTAC 16028

LOC_Os10g38470 953 GAGTTCAGATGTGTTACTACAG----TAGTAGTAACTTGGGTTGAAAAAA 998

|:||.|||||||||||||||:. .||||||||||||.||||||:|||

Contig005622 16029 GTGTCCAGATGTGTTACTACGTCTACCAGTAGTAACTTGTGTTGAATAAA 16078

LOC_Os10g38470 999 TATCTAGGGAACAGTTTGTCCAAGGTAAAATGTTATAAACCGAAATGAAT 1048

|||||||||||||||||||||||||||||||||||||:||.|| ::.

Contig005622 16079 TATCTAGGGAACAGTTTGTCCAAGGTAAAATGTTATAGACTGA----TTC 16124

LOC_Os10g38470 1049 AGTGTTAATTAGAGAAAAGAAACTAGTACAAGTGGCGTCTGGGTGAGAAA 1098

|.|||||||||||:||||||||.|||||||:|||||:|||||:|||||||

Contig005622 16125 ACTGTTAATTAGAAAAAAGAAATTAGTACAGGTGGCATCTGGATGAGAAA 16174

LOC_Os10g38470 1099 GGTCTTTACAACTTAATAACATGGCCCAATGAAAATAATTACAGATTTGG 1148

|:||:|||||||||||:|||||||||||||||||||||||||||||||||

Contig005622 16175 GATCATTACAACTTAAAAACATGGCCCAATGAAAATAATTACAGATTTGG 16224

LOC_Os10g38470 1149 CTGTGAAAAAAAAAGAGAGAAGACTATACTGTGCGGGACAATGAAAAGGT 1198

||||| |||||||||||||||||||||||||.|||||||||||||||||

Contig005622 16225 CTGTG--AAAAAAAGAGAGAAGACTATACTGTCCGGGACAATGAAAAGGT 16272

LOC_Os10g38470 1199 ACACCAATAATATAT 1213

|.|||||||||||||

Contig005622 16273 ATACCAATAATATAT 16287
