# Supplementary figures and images for: Gene Expression Profiles Deciphering Rice Phenotypic Variation between Nipponbare (Japonica) and 93-11 (Indica) during Oxidative Stress
Source: PLoS One. 2010 Jan 8;5(1):e8632. doi: 10.1371/journal.pone.0008632 (PMC2799674; doi:10.1371/journal.pone.0008632)

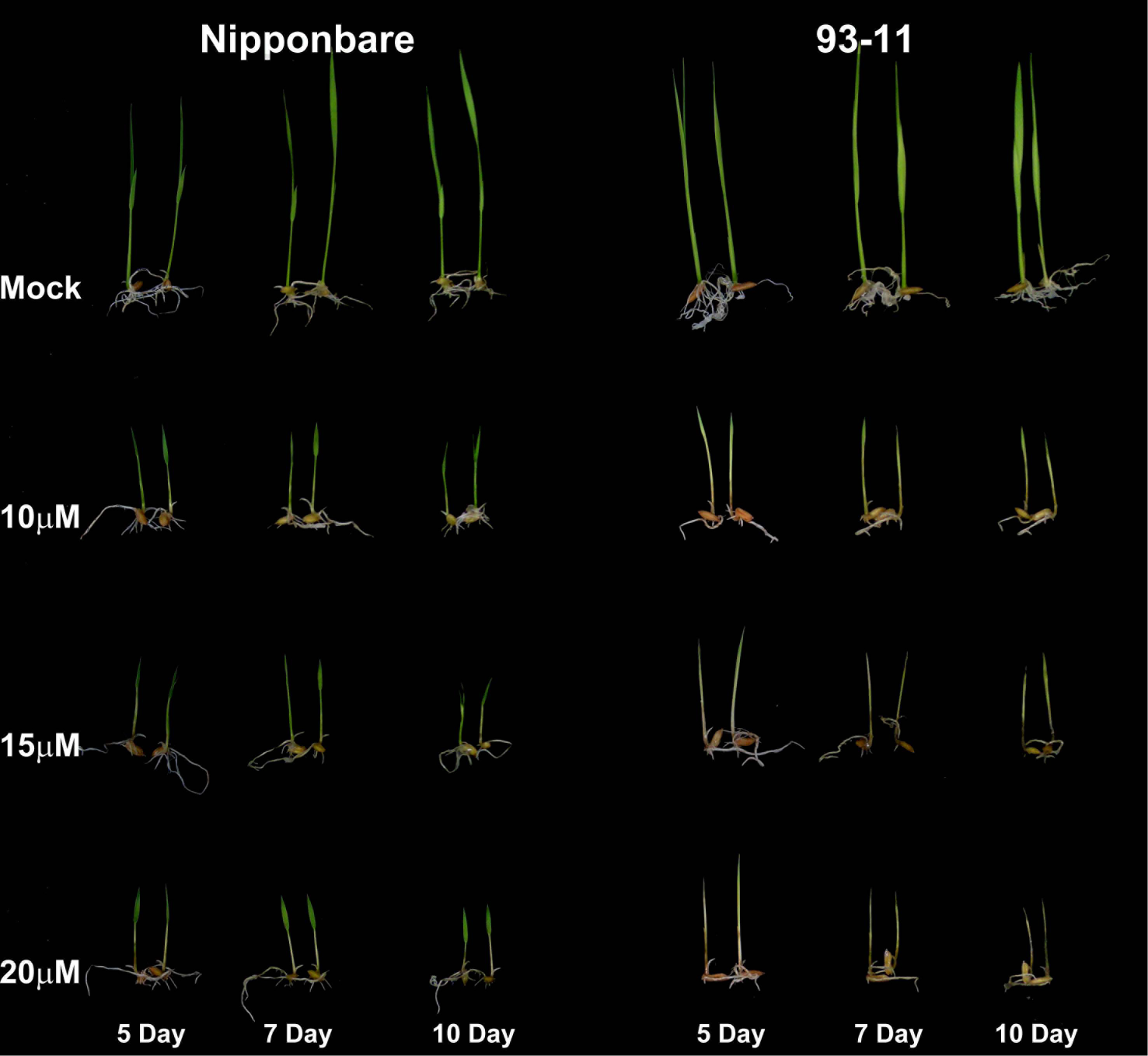

Supplement: Figure S1 — MV concentrations and time effects on rice japonica variety (Nipponbare) and indica variety (93-11) seedlings. Nipponbare (left) and 93-11 (right) sprouted seeds were mock-treated (water) or treated with a gradient concentration of MV (10 μM, 15 μM, and 20 μM) for 5 days, 7 days, and 10 days. (0.52 MB TIF) [file pone.0008632.s002.tif]

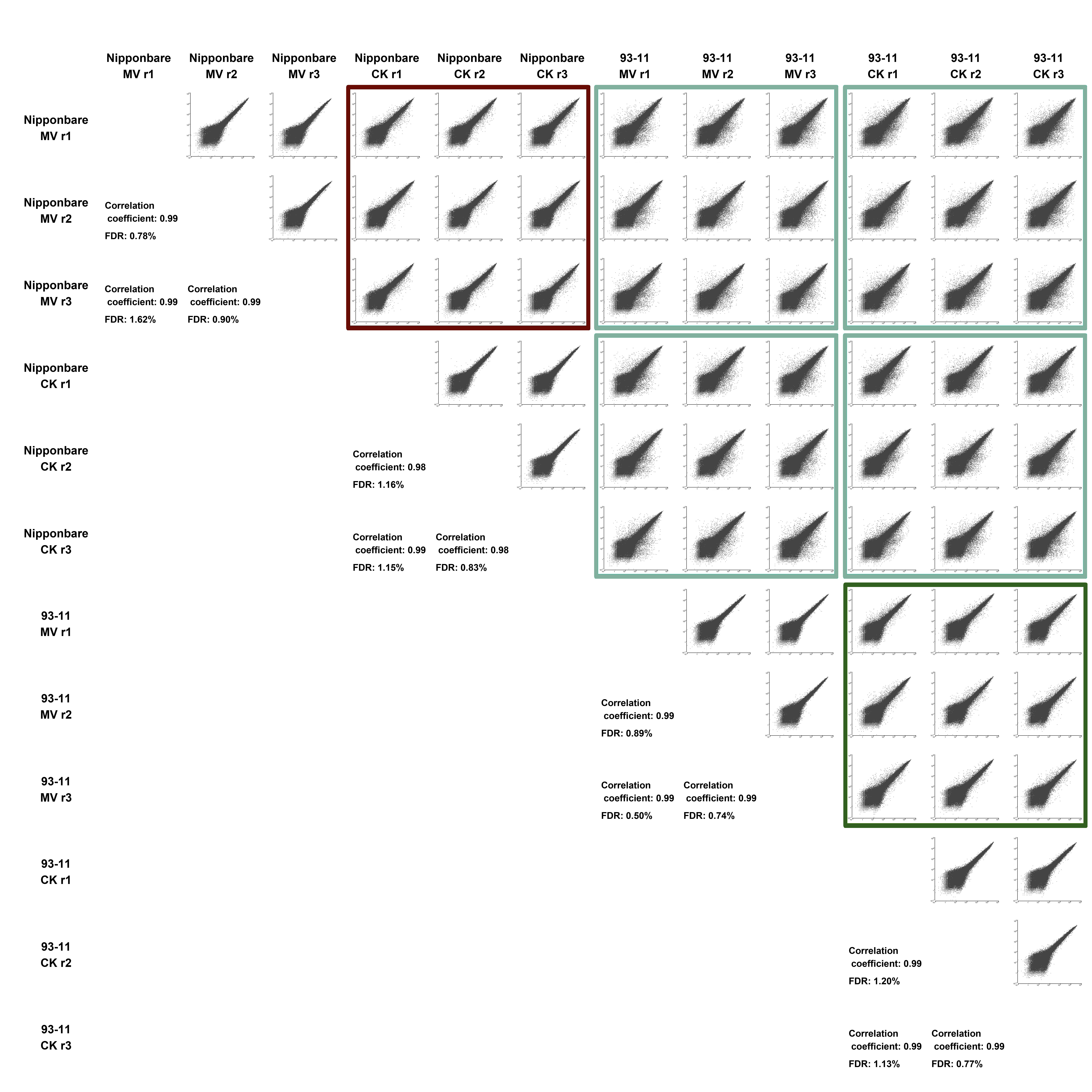

Supplement: Figure S2 — Pair-wise scatter plots for the raw probe set intensity data across all arrays. (5.84 MB JPG) [file pone.0008632.s003.jpg]

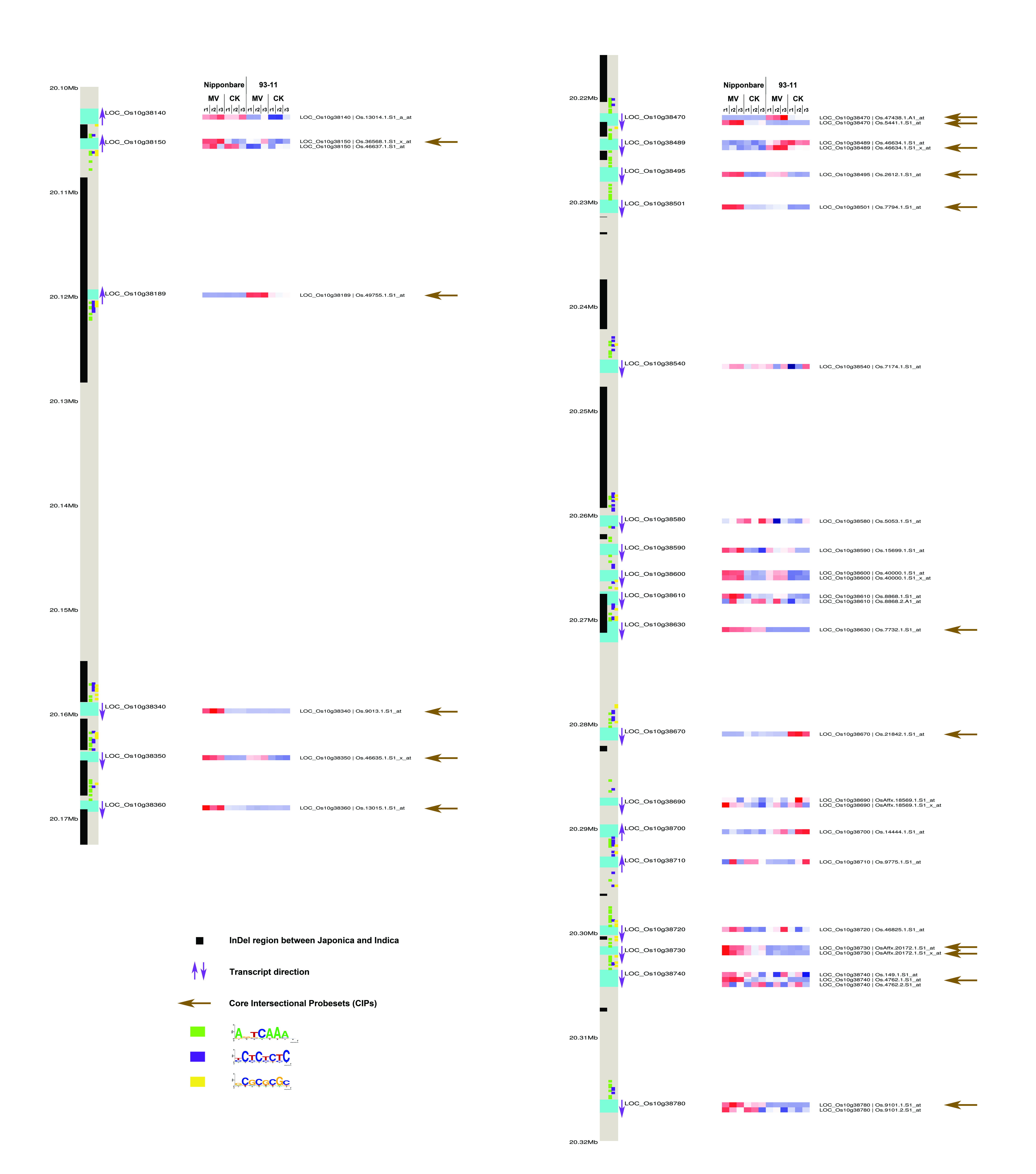

Supplement: Figure S3 — Sketch map of GSTs encoded in an interval from 20.10 Mb to 20.32 Mb on chromosome 10. (4.35 MB JPG) [file pone.0008632.s004.jpg]
