# Supplementary material for: Gene Expression Profiles Deciphering Rice Phenotypic Variation between Nipponbare (Japonica) and 93-11 (Indica) during Oxidative Stress
Source: PLoS One. 2010 Jan 8;5(1):e8632. doi: 10.1371/journal.pone.0008632 (PMC2799674; doi:10.1371/journal.pone.0008632)
Supplement: Table S1 — 1062 CIPs with differential expression between indica (93-11) and japonica (Nipponbare) under MV treatment. (0.24 MB PDF) [file pone.0008632.s005.pdf]

Table S1. 1062 CIPs with differential expression between indica (93-11) and japonica (Nipponbare) under MV treatment

Probe Set ID	Locus ID	probe #	ANOVA p-value [a]			FDR q-value [b]			Nipponbare MV			Nipponbare CK			93-11 MV			93-11 CK			Indel (Indica vs Japonica)			Annotation
			C [c]	T [d]	C×T [e]	C	T	C×T	Repeat 1	Repeat 2	Repeat 3	Repeat 1	Repeat 2	Repeat 3	Repeat 1	Repeat 2	Repeat 3	Repeat 1	Repeat 2	Repeat 3	upstream	gene	downstream	
Os.30617.1.S1_at	LOC_Os01g02110	4	1.8E-04	5.2E-06	3.6E-04	1.6E-03	7.9E-04	5.6E-02	44.1	47.3	31.8	255.7	154.4	167.6	17.4	45.8	34.5	289.1	252.8	194.2	Yes [f]	No [g]	No	helix-loop-helix DNA-binding domain containing protein, expressed
Os.30376.1.S1_at	LOC_Os01g02130	5	2.6E-08	2.9E-04	1.3E-04	2.7E-06	1.3E-02	3.0E-02	538.3	538.9	628.1	343.2	338.8	424.2	34.6	47.5	13.3	4.9	40	40.2	No	Yes	Yes	expressed protein
Os.32123.1.S1_at	LOC_Os01g02930	6	5.6E-06	1.9E-08	1.7E-05	1.1E-04	2.1E-05	7.9E-03	341.9	424.7	373.2	1155.4	855.5	946.5	317.3	325.6	279.7	2185.2	1812	1905.6	No	No	No	glycosyltransferase, putative, expressed
Os.38065.1.S1_at	LOC_Os01g03030	11	2.1E-06	1.1E-06	5.2E-05	5.6E-05	2.8E-04	1.6E-02	3000.3	2760.4	2859.7	1378.1	1226.7	1449.5	1420.4	1369.4	1260.4	1320.7	1130.8	1032.2	Yes	No	No	alanyl-tRNA synthetase, putative, expressed
Os.22594.1.S1_at	LOC_Os01g03390	5	7.6E-09	1.8E-09	3.2E-06	1.3E-06	5.2E-06	2.6E-03	3851	3434.2	4111.3	1563.8	1574	1836.9	2056.5	2145.3	2000.2	1004.3	1268.9	1098	No	No	No	Bowman-Birk type bran trypsin inhibitor precursor, putative, expressed
Os.29768.1.S1_at	LOC_Os01g03620	5	6.1E-08	6.9E-09	1.5E-07	4.8E-06	1.1E-05	3.9E-04	53.8	16.6	11.6	330.1	263	209.8	10.1	9	12	133.2	27.5	69.3	No	No	Yes	copper ion binding protein, putative, expressed
Os.21801.1.S1_at	LOC_Os01g04620	6	1.1E-04	1.1E-07	2.4E-05	1.1E-03	6.4E-05	9.9E-03	429.5	393.1	349.1	75.3	17.5	38.8	127	243.1	199.3	19.4	17.1	13.6	No	No	No	expressed protein
Os.16097.1.S1_a_at	LOC_Os01g04660	3	1.4E-04	7.4E-05	1.7E-04	1.3E-03	4.9E-03	3.5E-02	180.8	169.6	124	4.9	48	9	68.9	7.6	31.1	2.6	7.8	21.6	No	No	No	lipid phosphate phosphatase 3, chloroplast precursor, putative, expressed
Os.38086.1.S1_at	LOC_Os01g05530	3	7.4E-10	1.5E-04	4.2E-04	3.2E-07	8.1E-03	6.2E-02	1882.9	1793.7	2080.3	2436.7	2115.4	2702	259.6	174.7	258.4	306.5	267.9	381.2	Yes	No	No	expressed protein
Os.33885.1.S1_at	LOC_Os01g06220	8	3.2E-09	1.0E-10	2.2E-08	7.4E-07	1.3E-06	1.2E-04	9131.7	8241.4	8249.3	1252.9	1375.6	1146.3	3616.3	4946.3	3575.5	707.5	609.2	645.5	No	No	No	gibberellin receptor GID1L2, putative, expressed
Os.6389.1.S1_at	LOC_Os01g06310	3	2.4E-05	9.1E-07	3.1E-04	3.4E-04	2.5E-04	5.1E-02	334.6	227.3	208.1	28.9	150.7	82.4	213.9	168.1	192.7	77.6	119.8	48.6	Yes	Yes	Yes	glycine-rich cell wall structural protein precursor, putative, expressed
Os.46028.1.S1_at	LOC_Os01g06510	6	3.9E-09	2.1E-06	2.8E-06	8.3E-07	4.4E-04	2.4E-03	341.8	336.7	261.3	180.7	149.7	147.7	3.6	30.8	27.8	5.4	45.1	8.9	Yes	Yes	Yes	arginyl-tRNA synthetase, putative, expressed
Os.11371.1.S1_at	LOC_Os01g06740	9	1.2E-08	3.4E-08	3.3E-08	1.7E-06	3.0E-05	1.5E-04	558	607	558	67.9	88.9	65.1	18.9	34.9	2.3	5	3.1	27.3	Yes	Yes	Yes	protein synthesis inhibitor I, putative, expressed
OsAffx.23143.5.S1_at	LOC_Os01g06940	3	2.5E-06	2.8E-06	8.2E-06	6.4E-05	5.3E-04	4.9E-03	22.3	8.7	4.2	13.7	67.6	19.9	11.6	78.5	21.3	130.8	95.2	100.4	Yes	No	Yes	expressed protein
OsAffx.23143.5.S1_x_at	LOC_Os01g06940	4	3.4E-07	8.9E-07	1.2E-06	1.6E-05	2.4E-04	1.4E-03	24.5	4.8	9.3	77.7	17	48.4	48.3	20.2	18.1	302.2	186.5	258.5	Yes	No	Yes	expressed protein
OsAffx.23143.3.S1_at	LOC_Os01g06980	5	7.6E-05	7.3E-05	9.5E-05	8.3E-04	4.9E-03	2.4E-02	16.1	6.1	30.7	76.8	55.4	51.4	39.6	7.4	5.2	270.8	254.6	317.3	Yes	Yes	Yes	conserved hypothetical protein
OsAffx.23143.3.S1_x_at	LOC_Os01g06980	9	2.1E-08	2.3E-08	2.5E-08	2.4E-06	2.3E-05	1.3E-04	65	41.6	39	102	90.5	69.4	88.8	74.8	92	1220.5	1133.6	944.8	Yes	Yes	Yes	conserved hypothetical protein
OsAffx.23143.4.S1_s_at	LOC_Os01g07000	9	5.8E-07	8.2E-07	1.0E-06	2.2E-05	2.3E-04	1.3E-03	9.7	7.2	7.5	142.6	83.3	12.9	51.6	80.8	45.7	3807.5	2872	2836.2	Yes	Yes	Yes	conserved hypothetical protein
OsAffx.23143.1.S1_x_at	LOC_Os01g07010	11	1.0E-05	1.3E-05	1.6E-05	1.8E-04	1.5E-03	7.7E-03	10.2	45.5	26.4	225.6	167.9	114.8	159.8	232.1	116.8	4558.7	3341.7	3196.6	No	No	Yes	proline-rich protein 1, putative
OsAffx.10948.1.S1_at	LOC_Os01g07030	7	3.9E-06	2.9E-07	5.1E-06	8.9E-05	1.2E-04	3.5E-03	105.2	56.5	139.4	815.7	309.6	416.2	58.7	60.3	16.8	1613.8	1784.3	1425.3	No	No	Yes	proline-rich protein, putative, expressed
Os.46013.1.S1_at	LOC_Os01g07040	11	8.3E-07	4.0E-07	1.3E-06	2.9E-05	1.4E-04	1.5E-03	79.4	61.6	45.2	209.5	147.5	122	171.3	225.9	96.8	2870.4	2412.4	2164.4	No	No	Yes	proline-rich protein, putative
OsAffx.10949.1.S1_x_at	LOC_Os01g07060	6	5.5E-05	2.8E-06	8.8E-05	6.5E-04	5.4E-04	2.3E-02	14.7	10.8	6	138.6	22	145.5	8.7	9	9.8	516.7	483.5	704.3	Yes	No	No	tyrosine-rich hydroxyproline-rich glycoprotein, putative
Os.1665.1.S1_a_at	LOC_Os01g07300	3	2.0E-09	1.4E-07	1.3E-07	5.4E-07	7.2E-05	3.4E-04	631.7	512	361.8	227.2	213.8	220.3	155.2	152.4	242.5	79.5	153.7	129.2	No	No	No	expressed protein
Os.1665.2.S1_x_at	LOC_Os01g07300	4	6.3E-07	1.5E-06	3.9E-06	2.4E-05	3.4E-04	3.0E-03	381.7	373.9	292.6	216.3	182.4	213	131.4	201.8	227.7	141.7	198.3	155.1	No	No	No	expressed protein
Os.16494.1.S1_at	LOC_Os01g07376	3	3.7E-07	2.5E-06	7.2E-04	1.6E-05	4.9E-04	8.7E-02	512.6	632.5	608.4	716.2	1050.5	869.3	692.9	989.8	724.4	1263.5	1398.2	1325.7	No	Yes	No	thioredoxin H-type, putative, expressed
Os.51043.1.S1_at	LOC_Os01g07730	6	8.8E-08	1.2E-05	2.1E-05	6.1E-06	1.5E-03	9.1E-03	2122.6	2318.8	2172.6	825.8	637.6	1040.1	443.7	588.5	493	357.7	430.3	388.1	No	No	No	triose phosphate/phosphate translocator, non-green plastid,chloroplast precursor, putative, expressed
Os.15532.1.S1_at	LOC_Os01g07870	6	2.5E-05	6.4E-05	5.4E-05	3.5E-04	4.4E-03	1.7E-02	459.5	526	393.9	419.2	366.3	356.9	631	1013.1	690.1	333.7	290.3	310.7	Yes	Yes	Yes	multidrug resistance-associated protein 3, putative, expressed
Os.9695.2.S1_at	LOC_Os01g08830	5	3.7E-07	5.5E-08	8.9E-06	1.6E-05	4.0E-05	5.2E-03	704.7	517.3	619.4	332.7	271.9	346.6	431.2	311.4	375.3	311.4	197.9	218.2	No	No	No	F-box domain containing protein, expressed
Os.26887.2.A1_a_at	LOC_Os01g10340	3	1.3E-08	3.2E-06	3.9E-06	1.7E-06	5.8E-04	3.0E-03	10.6	2.2	2.7	1.8	27.3	5.6	209.7	221.3	238.1	119.9	192	224.2	No	Yes	No	electron transporter, putative, expressed
Os.55837.1.S1_at	LOC_Os01g10370	4	5.8E-06	6.6E-08	1.5E-05	1.2E-04	4.5E-05	7.5E-03	29.1	13.9	32.6	91.4	68.6	71.9	28.4	44.2	32.1	180.2	149.2	168.4	No	No	Yes	transcriptional factor TINY, putative, expressed
Os.18296.1.S1_x_at	LOC_Os01g10440	4	5.3E-07	1.2E-06	2.8E-06	2.1E-05	3.0E-04	2.4E-03	310.5	346.4	295.2	173.5	189.6	111.1	59.1	110.3	74	62.6	69.6	50.8	No	No	No	xylosyltransferase oxt, putative, expressed
Os.45991.1.S1_x_at	LOC_Os01g10440	6	7.0E-07	3.5E-05	3.4E-05	2.5E-05	3.0E-03	1.2E-02	358.8	381.1	331	242.2	208.7	183.4	130.9	123.6	70.9	96.5	84.9	103	No	No	No	xylosyltransferase oxt, putative, expressed
Os.15557.1.S1_at	LOC_Os01g11600	11	1.7E-08	1.1E-10	4.9E-08	2.1E-06	1.3E-06	1.9E-04	3782.6	4571	4247.4	395	449.5	381.1	1595.4	1918.2	1674.1	338.2	313	340.6	No	No	No	conserved hypothetical protein
Os.12340.1.S1_at	LOC_Os01g11730	4	3.7E-08	1.1E-08	1.9E-05	3.5E-06	1.5E-05	8.6E-03	4935.4	4849.8	5259.8	10285.8	9121.9	11649.4	2868.3	2537.3	2936.9	5971	5931.6	7681	No	No	No	esterase precursor, putative, expressed
Os.49073.1.S1_at	LOC_Os01g12570	11	1.0E-09	1.7E-08	3.2E-08	3.7E-07	2.0E-05	1.5E-04	223.4	234	194.5	726.5	357.7	512.5	1841.4	1660.6	1603.5	7663.2	6499.3	6634.6	No	No	No	3-methyl-2-oxobutanoate hydroxymethyltransferase, putative, expressed
OsAffx.23221.1.S1_s_at	LOC_Os01g12940	9	6.5E-09	2.1E-08	7.4E-08	1.1E-06	2.2E-05	2.5E-04	29.5	42.4	11.3	202.7	69.8	98.6	230.3	229.2	208	1050.6	888.2	1345.4	No	No	No	vegetative storage protein PNI288, putative
Os.22846.1.A1_at	LOC_Os01g13560	3	7.1E-05	3.1E-08	1.1E-03	7.9E-04	2.8E-05	1.1E-01	409.3	475.5	463.4	139	143.4	136.7	294.4	398.3	323.6	113.9	168.3	180.4	No	No	No	plant integral membrane protein TIGR01569 containing protein, expressed
Os.31185.1.S1_at	LOC_Os01g13950	7	1.6E-06	1.2E-07	3.3E-06	4.6E-05	6.5E-05	2.7E-03	423.1	445.2	430.4	86.3	54.3	95.3	117.3	118.4	151	30.8	82	86.8	No	No	Yes	OsGrx_A1 - glutaredoxin subgroup III, expressed
Os.39087.1.S1_at	LOC_Os01g14550	8	2.6E-08	1.3E-08	1.0E-06	2.7E-06	1.6E-05	1.3E-03	764.1	899	742.9	253.8	262.7	268.2	2000.1	2023.7	2624.5	544.2	468.6	491.9	No	No	Yes	pathogen-related protein, putative, expressed
Os.872.2.S1_at	LOC_Os01g14590	9	1.2E-06	5.1E-10	2.1E-06	3.8E-05	2.6E-06	2.0E-03	1754.3	1787.4	1694.7	362.5	355.2	292.7	2250.8	2772.9	2626.4	380.5	340.5	387.8	No	Yes	No	pathogen-related protein, putative, expressed
OsAffx.23248.1.S1_at	LOC_Os01g14710	3	5.1E-06	7.0E-05	8.1E-05	1.1E-04	4.7E-03	2.2E-02	102.1	152.9	108.5	467	241.6	338.4	88.5	65.3	91.2	123.4	74.3	263.3	No	No	Yes	conserved hypothetical protein
Os.22571.1.S1_at	LOC_Os01g15270	8	8.2E-06	2.2E-06	1.8E-05	1.5E-04	4.5E-04	8.4E-03	497.6	529.8	499	179	247.1	149.2	182.5	194.3	123.5	96.8	59.4	90.5	Yes	No	Yes	expressed protein
Os.37869.2.S1_a_at	LOC_Os01g15270	5	3.6E-07	1.0E-06	7.3E-05	1.6E-05	2.7E-04	2.0E-02	15882.2	13956.6	14206.3	12160.8	9638.2	11301.2	12424.5	8043.2	8614.4	6975.8	5467.3	5481.3	Yes	No	Yes	expressed protein
Os.40139.1.A1_x_at	LOC_Os01g15270	6	1.9E-07	2.8E-06	6.6E-05	1.0E-05	5.3E-04	1.9E-02	13462.2	12131.4	10467.3	5281.9	5010.4	4221.5	3632.1	4250.8	3531	1901.4	2054.4	2041.4	Yes	No	Yes	expressed protein
Os.10270.1.S1_at	LOC_Os01g15810	4	1.7E-04	5.9E-09	2.7E-04	1.6E-03	1.0E-05	4.7E-02	279.9	190.9	227	1104.7	730.8	1080.5	237.5	291.5	247.2	1567.8	1458	1401.2	No	No	No	peroxidase 9 precursor, putative, expressed
Os.207.1.S1_at	LOC_Os01g16900	3	8.0E-04	1.1E-06	1.3E-04	5.1E-03	2.8E-04	2.9E-02	4689.5	4497.9	4562.6	3230.8	2593.8	2938.4	3835.1	3955.5	3780.7	3174.4	2804.8	2741.7	No	Yes	No	3-isopropylmalate dehydrogenase, chloroplast precursor, putative, expressed
Os.43666.1.S1_at	LOC_Os01g17160	11	4.9E-05	6.9E-07	5.2E-05	6.0E-04	2.0E-04	1.6E-02	163.9	162.6	123.1	473.9	323.6	557.1	104.9	83.1	121.2	1149.4	998.9	1039.5	No	Yes	No	palmitoyltransferase ZDHHC9, putative, expressed
Os.43666.2.S1_x_at	LOC_Os01g17160	3	1.5E-04	8.6E-07	2.7E-04	1.4E-03	2.4E-04	4.7E-02	79.9	61.8	36.8	169.3	167	131.2	38.4	8.4	53.2	248.6	249	269.6	No	Yes	No	palmitoyltransferase ZDHHC9, putative, expressed
Os.446.1.S1_at	LOC_Os01g17330	3	1.4E-03	3.2E-07	1.1E-03	7.7E-03	1.3E-04	1.1E-01	329.2	531.1	409.7	47.7	15.5	55.5	176.8	266.9	219.9	6	49.6	58.6	No	No	No	eukaryotic translation initiation factor 6, putative, expressed
OsAffx.22919.2.S1_x_at	LOC_Os01g18950	7	5.0E-06	1.8E-08	1.7E-05	1.1E-04	2.0E-05	7.9E-03	84.9	62.1	33	1186.8	624.3	1014.5	161.9	111.2	164.7	3083	1772.2	3256.2	No	No	Yes	cationic peroxidase 1 precursor, putative
OsAffx.22919.1.S1_x_at	LOC_Os01g18970	10	2.0E-05	2.1E-08	3.6E-05	3.0E-04	2.2E-05	1.3E-02	80.7	66.8	77.4	1655.3	757.4	1322.7	204.5	137.1	195.9	5220.9	3769.4	5160	Yes	No	No	peroxidase 28 precursor, putative
Os.8031.1.S1_at	LOC_Os01g21120	4	3.0E-04	1.8E-05	2.5E-03	2.4E-03	1.9E-03	1.8E-01	285.3	475.2	339.2	192.4	203.4	294.3	723.4	753.1	712.7	300.2	272.6	263.3	No	No	No	ethylene-responsive factor-like protein 1, putative, expressed
Os.19003.1.S1_a_at	LOC_Os01g21160	7	2.1E-04	3.4E-04	1.5E-05	1.8E-03	1.4E-02	7.3E-03	2771.5	1983.2	2141.6	1403.9	1052	1383.4	1113.9	1187.9	1212	1287.4	1170.1	1330	Yes	No	No	dihydrolipoyllysine-residue acetyltransferase component of pyruvatedehydrogenase complex, putative, expressed
Os.2915.1.S1_at	LOC_Os01g21250	9	5.7E-08	2.3E-09	1.7E-06	4.6E-06	6.2E-06	1.7E-03	11525.4	10833.8	10863.8	3381	2669.5	2907.3	4499	4878.6	5394.3	1804.6	1528	1702.1	No	No	No	protein induced upon tuberization, putative, expressed
OsAffx.23327.2.S1_at	LOC_Os01g21620	5	2.0E-04	9.0E-09	1.2E-04	1.7E-03	1.3E-05	2.7E-02	463.4	335.8	244.8	1.5	1.2	1.7	112.6	209	213.1	1.5	2	2.6	Yes	No	Yes	conserved hypothetical protein
Os.21817.1.S1_at	LOC_Os01g21650	4	4.0E-04	1.3E-03	2.4E-03	3.0E-03	3.6E-02	1.8E-01	3	51.1	7.9	63.3	67.3	43.1	69.4	153.5	108.9	326.8	264.4	271.2	No	Yes	Yes	expressed protein
Os.20260.1.S1_at	LOC_Os01g22352	5	1.4E-08	3.0E-05	9.0E-05	1.8E-06	2.6E-03	2.3E-02	1665.8	1604.3	2017.5	810.9	900.9	1114.8	109.4	137	100.7	98.1	77	94.6	Yes	Yes	No	peroxidase 2 precursor, putative, expressed
Os.16121.1.S1_at	LOC_Os01g22370	4	1.3E-07	8.5E-05	8.6E-05	8.2E-06	5.4E-03	2.2E-02	81	86.6	55.9	77.6	213	74.7	3980.5	3787.6	3311	2349	2135	2926.1	Yes	Yes	No	peroxidase 1 precursor, putative, expressed
Os.56235.1.S1_at	LOC_Os01g22910	3	5.4E-08	1.2E-04	1.4E-04	4.4E-06	6.9E-03	3.1E-02	341.3	429.3	335.4	221.3	246.6	277.7	21.4	72.1	29.9	31.8	30.8	37.8	No	Yes	No	oxidoreductase, 2OG-Fe oxygenase family protein, expressed
OsAffx.23382.1.S1_at	LOC_Os01g25030	9	6.3E-12	1.9E-11	1.9E-11	2.4E-08	5.8E-07	1.5E-06	158	124.5	126	1266.1	769	1246.8	5.9	4.8	3.6	53.3	35.6	4.4	Yes	No	No	dirigent, putative, expressed
Os.7911.1.S1_at	LOC_Os01g27210	10	2.9E-13	1.0E-09	9.1E-10	5.9E-09	3.6E-06	1.5E-05	12161.4	12225.5	13544.4	5905.7	4838.7	6373.9	1246.3	1937.1	1616.7	917.2	820.3	1047.3	Yes	No	Yes	glutathione S-transferase IV, putative, expressed
Os.40662.1.S1_at	LOC_Os01g27340	10	1.4E-10	1.7E-10	1.6E-10	1.1E-07	1.6E-06	6.0E-06	2253	1864.3	2034.5	116.4	87.7	109.8	55	70.4	49.4	36.6	43	71.7	Yes	Yes	Yes	glutathione S-transferase GSTF1, putative, expressed
Os.36663.1.S1_at	LOC_Os01g27390	10	1.6E-08	2.5E-11	4.8E-08	2.0E-06	6.4E-07	1.8E-04	1140.7	1066.2	1578.4	4621.6	3123.9	4420.9	1348	1478	1378	11671.9	9344.5	10494.6	No	No	No	glutathione S-transferase GSTF2, putative, expressed
Os.7136.1.S1_at	LOC_Os01g27750	3	5.4E-07	9.1E-04	1.1E-03	2.1E-05	2.8E-02	1.1E-01	2522.7	2230.1	2609.5	1628.7	1435.3	1790.5	1469.2	1921.5	2332.6	1588.1	1421.6	2177.6	Yes	Yes	No	shikimate biosynthesis protein aroDE, putative, expressed
Os.9116.1.S1_at	LOC_Os01g28680	3	1.8E-07	1.6E-06	3.1E-04	1.0E-05	3.6E-04	5.2E-02	3116.4	2954.6	2290.1	1556.9	1105.3	1176.8	1256.1	1472.8	2012.4	1002.6	1035.2	1467	Yes	Yes	Yes	LEC14B, putative, expressed
OsAffx.23470.1.S1_at	LOC_Os01g32160	8	3.7E-07	8.3E-07	1.1E-06	1.6E-05	2.3E-04	1.3E-03	177.6	237.9	159.2	33.4	58.8	17.2	14.5	24.5	10.4	16.1	9.3	10.6	Yes	No	Yes	hypothetical protein
Os.4618.1.S1_at	LOC_Os01g32670	10	3.5E-08	4.3E-10	1.2E-07	3.3E-06	2.4E-06	3.2E-04	9882.7	11548.5	9705.3	903.8	903.9	867.1	4381.4	5573.8	5109	515.2	454.5	466.2	Yes	No	No	expressed protein
Os.7535.1.S1_at	LOC_Os01g32780	7	1.7E-06	1.0E-07	6.6E-05	4.8E-05	6.1E-05	1.9E-02	5628.6	5687.2	6039.7	2549.2	2531.3	2688.3	3612.3	3680.1	3129.1	1620.7	2470.3	2025.4	No	No	No	ethylene-responsive protein, putative, expressed
Os.7812.2.S1_x_at	LOC_Os01g35040	4	3.5E-06	7.5E-04	2.0E-03	8.2E-05	2.4E-02	1.6E-01	3000.5	2671.9	2692	2112.3	1976.2	2236.1	2102.3	1770.1	1661.3	1758.1	1638.3	1514.8	No	Yes	No	PRLI-interacting factor K, putative, expressed
Os.9827.1.S1_a_at	LOC_Os01g37000	3	1.4E-04	2.5E-10	7.6E-05	1.3E-03	1.8E-06	2.1E-02	1260.1	1339.9	1595.8	3253	3063.6	3181.5	1132.6	1286.8	1186.3	4107.3	3274.3	4109.4	No	No	No	carboxyl-terminal peptidase, putative, expressed
Os.9827.1.S1_at	LOC_Os01g37000	6	2.7E-06	2.1E-07	3.7E-04	6.8E-05	9.6E-05	5.8E-02	1056.3	1396.8	1660.5	2716	3361.6	2585.7	1856.1	1770.7	1422.9	5697.3	4915.1	6401.3	No	No	No	carboxyl-terminal peptidase, putative, expressed
Os.10255.1.S1_s_at	LOC_Os01g37350	8	4.7E-08	1.9E-05	8.6E-05	4.1E-06	2.0E-03	2.2E-02	507	427.5	509	220.7	213.8	255.3	40.5	36.9	36.9	28	49	28.5	Yes	Yes	Yes	retrotransposon protein, putative, Ty3-gypsy subclass, expressed
Os.6157.1.S1_at	LOC_Os01g37630	6	1.0E-04	2.3E-08	3.7E-05	1.0E-03	2.4E-05	1.3E-02	1630.1	1189.8	952.7	168.8	102.1	107.4	499.5	606.5	552	182.8	229.5	111	No	No	No	esterase PIR7B, putative, expressed
Os.38249.2.S1_at	LOC_Os01g38530	4	5.9E-06	4.2E-06	1.2E-03	1.2E-04	6.9E-04	1.2E-01	1903.5	1610.5	2136.2	1127.4	1218.3	1327.2	1222.6	1332.9	1258.7	927.1	1157.3	1028.8	Yes	No	Yes	early flowering 3, putative, expressed
Os.37454.1.S1_at	LOC_Os01g38580	6	1.5E-06	5.7E-07	4.0E-06	4.3E-05	1.8E-04	3.1E-03	268.4	112.9	267	170.3	92.6	85	256	297	285.9	44.1	100.1	104	Yes	Yes	Yes	beta,beta-carotene 9,10-dioxygenase, putative, expressed
Os.30657.1.S1_at	LOC_Os01g40260	7	5.9E-06	1.2E-06	8.0E-06	1.2E-04	2.9E-04	4.8E-03	178	200.3	221.6	19.6	72.4	17.3	81.7	90.4	71.7	3.6	2.5	1	Yes	No	No	OsWRKY77 - Superfamily of rice TFs having WRKY and zinc finger domains, expressed
Os.8965.1.S1_at	LOC_Os01g40280	4	5.3E-07	1.3E-05	5.1E-04	2.1E-05	1.5E-03	7.0E-02	1279.4	1081.9	1091.9	923.1	709.6	791.2	705.6	827.3	813.9	911.9	697.6	775.5	No	No	No	integral membrane protein, putative, expressed
OsAffx.21410.1.S1_x_at	LOC_Os01g40560	3	1.5E-04	1.4E-04	8.4E-05	1.4E-03	7.5E-03	2.2E-02	186.4	114.7	154	907	495.1	697.9	194.9	122.1	174.5	19.8	149.4	238	No	Yes	No	hypersensitive-induced response protein, putative
Os.2010.1.S1_at	LOC_Os01g40870	3	2.2E-10	3.9E-05	1.0E-04	1.5E-07	3.2E-03	2.5E-02	2981	2701.1	2740.1	5420.1	3791.1	4899.2	706.4	1048.1	716.3	1107.3	1066.2	1850.5	No	No	Yes	aldehyde dehydrogenase, mitochondrial precursor, putative, expressed
Os.20265.1.S1_x_at	LOC_Os01g41120	4	4.0E-05	3.5E-08	3.1E-05	5.1E-04	3.1E-05	1.2E-02	82.3	60.2	106.3	412.6	309.3	317.9	68.7	109.8	63.9	697.7	572.4	484.8	No	No	No	protein binding protein, putative, expressed
Os.20265.2.S1_at	LOC_Os01g41120	8	2.8E-04	4.7E-07	1.8E-04	2.3E-03	1.6E-04	3.6E-02	27.3	78.6	85.7	130.6	327.1	227.6	40.6	86.9	46.3	580.6	784.2	596.8	No	No	No	protein binding protein, putative, expressed
Os.10348.1.S1_at	LOC_Os01g41430	8	4.2E-06	4.8E-10	4.1E-06	9.4E-05	2.5E-06	3.1E-03	1669.3	2251.4	1935.6	146.3	193.5	172	1069.2	1446.4	1083.7	274.9	186.9	191.8	Yes	No	No	flavonol-3-O-glycoside-7-O-glucosyltransferase 1, putative, expressed
Os.22681.1.S1_at	LOC_Os01g41810	5	1.9E-08	1.2E-08	2.3E-05	2.2E-06	1.6E-05	9.6E-03	702.1	584.7	453.9	169.7	226.9	159.8	119.8	193.4	89.9	28.5	45.6	16.9	No	No	Yes	cytochrome P450 72A1, putative, expressed
Os.8825.1.S1_a_at	LOC_Os01g42234	4	6.3E-05	1.2E-05	7.0E-04	7.2E-04	1.4E-03	8.6E-02	242.3	307.8	257.9	512.9	476.3	328.1	273.1	440.7	370.5	1075.2	1021.2	820.7	No	No	No	amino-acid permease C584.13, putative, expressed
OsAffx.11437.1.S1_at	LOC_Os01g42350	4	2.6E-03	1.6E-04	2.5E-03	1.2E-02	8.4E-03	1.8E-01	7.8	5.8	23	107.7	62.5	65	4	11.8	3.1	380.5	248.3	160.5	No	No	No	PDR5-like ABC transporter, putative
Os.467.1.S1_a_at	LOC_Os01g42380	9	2.5E-08	2.5E-09	4.1E-07	2.7E-06	6.4E-06	7.2E-04	1634.4	1610.6	1476	520.3	348.4	415.2	525.4	620.1	736.4	267.1	203.4	204.3	No	No	No	PDR5-like ABC transporter, putative, expressed

Probe Set ID	Locus ID	probe #	ANOVA p-value			FDR q-value			Nipponbare MV			Nipponbare CK			93-11 MV			93-11 CK			Indel (Indica vs Japonica)			Annotation
			C	T	C×T	C	T	C×T	Repeat 1	Repeat 2	Repeat 3	Repeat 1	Repeat 2	Repeat 3	Repeat 1	Repeat 2	Repeat 3	Repeat 1	Repeat 2	Repeat 3	upstream	gene	downstream	
Os.1043.1.S1_at	LOC_Os01g42860	4	9.9E-06	6.6E-06	5.2E-05	1.8E-04	9.4E-04	1.6E-02	289.8	180.9	233.3	97.2	131.8	103.6	104.2	153	92.4	34.2	91.4	88.2	No	No	Yes	subtilisin-chymotrypsin inhibitor 2, putative, expressed
Os.21830.1.S1_at	LOC_Os01g43110	6	2.6E-08	7.1E-05	7.7E-05	2.8E-06	4.8E-03	2.1E-02	55	50.2	43.4	76.3	56.7	63	190.9	228.5	225.9	381.2	509.8	466.9	No	No	No	expressed protein
Os.9195.1.S1_at	LOC_Os01g43370	5	1.4E-05	2.9E-07	6.1E-05	2.3E-04	1.2E-04	1.8E-02	6525.7	6236.1	7149.7	2575.1	2434.5	2964.4	5511.8	4927.2	5363	3561.8	3553.6	3849.4	Yes	No	No	expressed protein
Os.767.1.S1_at	LOC_Os01g43700	7	8.7E-05	6.2E-08	9.7E-05	9.2E-04	4.3E-05	2.4E-02	2084.8	1849.4	2018.5	809.1	820.1	827.4	1262.2	1417.6	1430	837.2	794.1	810.1	No	No	No	cytochrome P450 72A1, putative, expressed
Os.6452.1.S1_at	LOC_Os01g43710	4	2.1E-04	1.8E-04	2.1E-04	1.8E-03	9.0E-03	4.0E-02	220.2	167.7	169.1	50.9	49.7	51.3	49.4	1.8	36.1	64.8	29.8	39.6	No	No	No	cytochrome P450 72A1, putative, expressed
Os.6452.2.A1_a_at	LOC_Os01g43710	8	1.4E-05	9.5E-08	4.6E-05	2.3E-04	5.7E-05	1.5E-02	8454.6	8694.3	8098.8	3083.3	2537.4	2802.3	4415.9	5188	4566.9	2270.7	2406.2	2243.2	No	No	No	cytochrome P450 72A1, putative, expressed
Os.33102.1.S1_at	LOC_Os01g43740	9	3.4E-09	4.5E-11	3.7E-09	7.7E-07	8.5E-07	3.6E-05	2391.6	3194	2673.3	23.4	20.6	8.8	672.9	1283.4	927.4	36.2	7.4	8	No	No	No	cytochrome P450 72A1, putative, expressed
Os.41199.1.A1_at	LOC_Os01g43774	10	3.2E-07	3.1E-10	2.8E-07	1.5E-05	2.0E-06	5.7E-04	5275.2	3768.2	4838.8	51.8	99.4	19.1	1030.4	1049.6	969.9	52.6	22.5	3.6	No	No	No	cytochrome P450 72A1, putative, expressed
Os.34501.1.S1_x_at	LOC_Os01g43890	3	1.1E-06	5.7E-07	7.6E-06	3.4E-05	1.8E-04	4.6E-03	533.7	418.4	317.4	104.2	91.9	132.9	151.1	211	226.9	74.6	107.6	92.6	Yes	No	No	SCPL50, putative, expressed
Os.12253.1.S1_at	LOC_Os01g45110	11	9.1E-13	1.7E-14	1.1E-12	1.1E-08	9.2E-09	2.5E-07	10995.3	11227.6	10582.4	246.4	238.8	225.5	1712	1534.9	1860.8	312.6	281.7	200	No	No	No	cytokinin-O-glucosyltransferase 1, putative, expressed
OsAffx.11519.1.S1_at	LOC_Os01g48530	3	2.9E-04	8.3E-06	2.7E-03	2.3E-03	1.1E-03	1.9E-01	328.8	444.6	385.8	569.1	434.1	550.2	393.8	458.8	404.1	953.7	952.4	706.2	No	No	No	expressed protein
Os.4901.1.S1_at	LOC_Os01g49160	4	3.3E-07	3.3E-06	6.5E-04	1.5E-05	5.9E-04	8.2E-02	983.8	992	946.9	510.2	562.5	539.8	556.5	497.9	402.3	339.1	278.9	296.6	No	No	No	myb-like DNA-binding domain containing protein, expressed
Os.26819.1.A1_at	LOC_Os01g49614	3	4.3E-08	7.4E-04	1.1E-03	3.8E-06	2.4E-02	1.1E-01	20.8	7.1	11.1	1.4	1.3	1.2	68.2	85.2	101.6	59.5	65.1	52.9	Yes	Yes	Yes	Ser/Thr protein kinase, putative, expressed
Os.38022.1.S1_at	LOC_Os01g49710	3	3.6E-06	1.2E-10	7.4E-04	8.4E-05	1.4E-06	8.9E-02	475.8	440.4	365.3	92	129.5	76.2	427.1	544.4	535	167.3	155.5	132.9	No	No	Yes	glutathione S-transferase GSTU6, putative, expressed
OsAffx.11535.1.S1_at	LOC_Os01g49720	4	1.2E-04	2.3E-07	1.5E-03	1.2E-03	1.0E-04	1.3E-01	802.9	590.6	632.8	165.2	130.2	145.4	353.8	298.3	372.7	87.4	57.4	71.8	Yes	No	No	glutathione S-transferase GSTU6, putative, expressed
Os.11800.1.S1_at	LOC_Os01g50100	7	3.7E-08	2.9E-11	3.2E-08	3.5E-06	7.2E-07	1.5E-04	2729.1	3053.4	2479.4	200.8	280.3	253.2	1141.5	1574.5	1424.9	184	322.9	226.2	No	Yes	No	multidrug resistance protein 4, putative, expressed
Os.11800.1.S1_s_at	LOC_Os01g50100	8	6.6E-06	5.6E-09	9.8E-06	1.3E-04	1.0E-05	5.6E-03	3993.8	4623.8	4188.2	376.8	462.8	458.2	1715.3	2686.3	2290.9	331.7	511.8	395.8	No	Yes	No	multidrug resistance protein 4, putative, expressed
Os.39411.1.A1_at	LOC_Os01g50100	7	2.2E-10	1.8E-10	8.3E-10	1.5E-07	1.6E-06	1.5E-05	1244	1481	1448.8	216.8	260.1	256.4	148.8	208.8	208.4	97.6	191.6	114.2	No	Yes	No	multidrug resistance protein 4, putative, expressed
Os.21788.1.S1_at	LOC_Os01g50530	3	6.3E-04	1.2E-03	1.4E-03	4.3E-03	3.4E-02	1.3E-01	15.7	45.9	33	175.6	131.7	166.6	5.4	3.5	4.5	2.1	8.8	2.1	Yes	Yes	Yes	cytochrome P450 monooxygenase CYP711A, putative, expressed
Os.52529.1.S1_x_at	LOC_Os01g50530	6	2.0E-05	6.9E-05	6.8E-05	3.0E-04	4.7E-03	1.9E-02	132.7	194.5	74.1	378.3	228.7	287.7	9.2	45.3	14.4	25.1	25.9	10.5	Yes	Yes	Yes	cytochrome P450 monooxygenase CYP711A, putative, expressed
Os.18272.1.S1_x_at	LOC_Os01g51060	3	8.6E-05	1.5E-04	6.2E-05	9.1E-04	8.0E-03	1.8E-02	94	109.2	85.5	135	88.3	142.3	117.7	81.8	79.9	224.1	270	262.4	Yes	No	No	IAA-amino acid hydrolase ILR1-like 4 precursor, putative, expressed
Os.4124.1.S1_a_at	LOC_Os01g51390	5	3.9E-05	9.5E-05	1.5E-03	5.0E-04	5.8E-03	1.3E-01	2183.9	2328.3	1973.2	1154.4	1189	1066	1051.1	1225.9	919.6	968.3	885	664.6	Yes	No	Yes	mitochondrial-processing peptidase beta subunit, mitochondrial precursor, putative, expressed
Os.37243.1.S1_at	LOC_Os01g51670	10	2.8E-09	4.6E-06	4.5E-06	6.9E-07	7.3E-04	3.3E-03	2072.2	1809.9	2156.7	641.3	851.8	911.9	46.9	62	47.4	53.8	31.5	50.4	Yes	Yes	Yes	expressed protein
Os.37243.2.S1_x_at	LOC_Os01g51670	9	3.9E-09	5.5E-06	4.6E-06	8.3E-07	8.2E-04	3.3E-03	1697.8	1548.6	1766.9	656.7	604.5	884.9	7.6	19	32	38.9	13.7	24.7	Yes	Yes	Yes	expressed protein
Os.30634.1.S1_at	LOC_Os01g51690	9	2.8E-07	1.1E-06	8.8E-07	1.4E-05	2.9E-04	1.2E-03	693.3	502.2	506.1	122.6	92.7	94.3	51.5	47.5	102.4	7.8	45.4	60.3	Yes	Yes	Yes	OsWRKY26 - Superfamily of rice TFs having WRKY and zinc finger domains, expressed
Os.57351.1.S1_at	LOC_Os01g52260	8	4.4E-07	1.5E-05	1.3E-05	1.8E-05	1.7E-03	6.9E-03	324.1	298.6	387.4	105.9	128.6	104.8	4	22.2	39.5	5	4	5.7	No	No	No	serine acetyltransferase 3, mitochondrial precursor, putative, expressed
Os.32865.1.S1_at	LOC_Os01g52470	4	1.2E-05	5.9E-06	8.6E-06	2.0E-04	8.7E-04	5.1E-03	237.6	270.3	179.6	4	4	8.8	115.7	60.5	108.6	3.5	40.7	12.8	Yes	No	No	elongation factor 2, putative, expressed
Os.29866.1.S1_at	LOC_Os01g52530	6	2.4E-05	1.3E-06	3.0E-05	3.5E-04	3.1E-04	1.1E-02	964	1026.4	822	428.7	489.2	396.9	515	528.9	494.7	522.4	520.9	424.3	No	No	Yes	ribose-phosphate pyrophosphokinase 3, putative, expressed
Os.26924.1.A1_at	LOC_Os01g54240	3	4.3E-07	1.6E-04	1.0E-04	1.8E-05	8.3E-03	2.6E-02	58.9	79.8	65.7	87.9	51.7	77.1	320.6	462.5	330.1	218.6	241.2	304.9	No	Yes	No	nup85 Nucleoporin family protein, expressed
Os.42124.1.S1_at	LOC_Os01g54450	3	1.3E-06	3.7E-03	1.9E-03	4.0E-05	7.3E-02	1.5E-01	146.2	77.4	157.7	106.7	16.3	122.7	566.6	463.9	540.8	366.2	253.5	307.5	No	No	No	nuclear protein, putative, expressed
OsAffx.21616.1.S1_s_at	LOC_Os01g54890	4	3.0E-06	1.2E-08	2.1E-04	7.3E-05	1.5E-05	4.0E-02	12442.6	13973.9	14264.9	5210.1	5220	7589.7	10846.2	10085.2	10166.4	5001	4715.2	5484.2	No	No	No	ethylene-responsive transcription factor 2, putative, expressed
Os.37620.1.S1_at	LOC_Os01g55820	3	5.2E-06	2.9E-05	7.7E-04	1.1E-04	2.6E-03	9.1E-02	131.2	87.4	105.4	320.8	294.9	355.9	66.5	71.2	92.8	239.5	188	196.9	No	No	No	glucan endo-1,3-beta-glucosidase 1 precursor, putative, expressed
Os.29865.1.S1_at	LOC_Os01g56570	4	1.8E-06	5.5E-04	5.0E-06	5.0E-05	2.0E-02	3.5E-03	1107.9	1397.8	1419.4	464	310.3	408.6	199.7	241.4	264.9	670.4	541.4	553.3	Yes	No	No	xylosyltransferase 2, putative, expressed
OsAffx.11629.1.S1_at	LOC_Os01g56890	5	3.1E-09	4.0E-04	1.1E-04	7.4E-07	1.6E-02	2.6E-02	385.1	337.3	308.2	173.5	207.7	219.8	30.2	45	59.1	85.6	62.3	31.5	No	No	No	expressed protein
OsAffx.11631.1.S1_x_at	LOC_Os01g57066	6	3.8E-07	2.2E-06	4.8E-04	1.7E-05	4.5E-04	6.8E-02	3324	3077.7	2942.2	1712.6	1538.3	1723.3	1096.7	1052.6	1178.3	842.7	800.9	872.6	No	No	No	transmembrane protein 49, putative, expressed
Os.26729.1.A1_at	LOC_Os01g57073	5	1.1E-06	3.2E-06	6.2E-04	3.4E-05	5.8E-04	8.0E-02	3513.6	4497.2	3560.7	1790	2516.5	2150.1	2167	2591.4	2358.8	1547.5	1828.6	1795.3	No	Yes	Yes	insulin-degrading enzyme, putative, expressed
Os.9073.1.S1_at	LOC_Os01g57470	4	4.0E-06	1.6E-05	3.9E-05	9.0E-05	1.8E-03	1.3E-02	554.4	479.3	690.7	162.6	205.7	208.4	260.6	240.3	146.3	173.2	59	149.9	Yes	No	Yes	caltractin, putative, expressed
Os.30258.1.S1_at	LOC_Os01g58240	9	4.7E-09	5.0E-06	1.2E-05	9.3E-07	7.7E-04	6.3E-03	1171.2	1733.2	1462.2	564.8	736.3	600.2	20.3	33	6.8	11.8	4.2	5.4	No	Yes	Yes	peptidase/ subtilase, putative, expressed
Os.30258.2.S1_x_at	LOC_Os01g58240	3	1.4E-08	3.1E-05	1.6E-05	1.8E-06	2.7E-03	7.7E-03	468.2	369	321.6	199	331.4	228.4	11.1	11.9	18.9	11.3	9.8	7	No	Yes	Yes	peptidase/ subtilase, putative, expressed
Os.55436.1.S1_at	LOC_Os01g58670	4	9.4E-09	1.9E-04	2.1E-04	1.4E-06	9.4E-03	4.0E-02	40.3	51.1	39.6	37	70.5	68.8	575.7	733.5	777.8	910.6	1046.3	1010.7	Yes	No	No	expressed protein
Os.36161.1.S1_at	LOC_Os01g58960	9	1.8E-05	3.2E-07	5.4E-05	2.8E-04	1.3E-04	1.7E-02	237.3	307.8	332.5	47.3	104.7	59.8	556.5	797.9	753.3	110.4	79.3	102.5	No	No	No	cytochrome P450 94A1, putative, expressed
Os.36161.2.S1_x_at	LOC_Os01g58960	4	9.7E-05	5.1E-06	1.1E-03	1.0E-03	7.9E-04	1.1E-01	194.7	180.6	156.4	98.8	86.9	75	272.4	389.5	335.1	102.2	80.6	107.6	No	No	No	cytochrome P450 94A1, putative, expressed
Os.54928.1.S1_at	LOC_Os01g59680	6	5.3E-06	3.4E-07	7.2E-06	1.1E-04	1.3E-04	4.5E-03	53.3	69.2	67	278.6	204.5	237.2	73.2	68.3	97.1	624.9	619.4	618.3	No	No	No	NHL25, putative, expressed
Os.4384.1.S1_at	LOC_Os01g60020	8	8.4E-09	3.9E-10	4.3E-08	1.3E-06	2.2E-06	1.7E-04	887.6	1061.5	1085.1	222.7	375.5	309.7	539.8	528.9	637.6	139.4	201.6	208.7	No	No	No	NAC domain-containing protein 68, putative, expressed
Os.35704.2.S1_at	LOC_Os01g60930	7	9.4E-06	1.4E-05	4.2E-05	1.7E-04	1.6E-03	1.4E-02	196.9	157.3	212.6	301.3	175.5	285.3	228.5	185.6	256.6	429.3	371.7	561.5	Yes	No	No	expressed protein
Os.36162.1.S1_at	LOC_Os01g61044	5	5.5E-06	9.3E-04	1.1E-03	1.1E-04	2.8E-02	1.1E-01	297	447.2	374.3	143.4	254.4	126.4	1.1	6.6	0.7	6	1.7	3.1	No	Yes	Yes	amino acid-polyamine transporter, putative, expressed
Os.36162.1.S1_s_at	LOC_Os01g61044	4	2.9E-06	2.6E-04	4.8E-04	7.1E-05	1.2E-02	6.8E-02	1109.3	1136.9	1131.5	870.4	580.5	702.7	542.4	494.2	533.3	478.5	568	564.1	No	Yes	Yes	amino acid-polyamine transporter, putative, expressed
Os.31778.1.S1_x_at	LOC_Os01g61610	3	4.0E-04	1.8E-04	6.0E-04	3.0E-03	9.1E-03	7.8E-02	2.7	12.5	5.1	3.7	3.8	26	3.5	2.3	2.4	53.2	6.7	15.3	No	No	No	gibberellin 20 oxidase 2, putative, expressed
Os.36919.1.S1_at	LOC_Os01g62010	5	1.6E-05	1.3E-03	2.0E-03	2.6E-04	3.6E-02	1.6E-01	1281.3	1371.9	1811	575.4	707.1	846	260.6	300.6	315.9	267	215.5	223.2	No	No	No	monoglyceride lipase, putative, expressed
Os.36919.1.S1_x_at	LOC_Os01g62010	3	2.2E-06	1.0E-04	2.8E-04	5.8E-05	6.1E-03	4.8E-02	861.6	1149.8	1293.1	441.9	547.1	642.5	289.5	343	297.2	221.3	247.7	247.8	No	No	No	monoglyceride lipase, putative, expressed
Os.36919.2.S1_s_at	LOC_Os01g62010	6	2.2E-06	8.7E-05	2.8E-04	5.7E-05	5.5E-03	4.8E-02	823.3	881.8	1007.4	324.4	391.5	476.3	198.7	210.1	155	151.9	101.4	150.4	No	No	No	monoglyceride lipase, putative, expressed
Os.28023.1.S1_at	LOC_Os01g62130	3	2.2E-04	1.3E-05	1.1E-05	1.9E-03	1.5E-03	5.8E-03	499.7	312.4	367	91.1	38.9	122.9	164.5	192.1	181.8	191.8	165	237.5	No	No	No	zinc finger, C2H2 type family protein, expressed
Os.11253.1.S1_at	LOC_Os01g63210	7	1.0E-04	2.5E-09	5.3E-05	1.1E-03	6.4E-06	1.6E-02	10583.2	12285.7	12663.6	2968.8	4105.1	3319.5	9838.6	10462.6	10388.7	4257.6	4196.8	4725.7	No	No	No	expressed protein
Os.2623.1.S1_at	LOC_Os01g63270	3	1.5E-06	8.4E-05	5.1E-03	4.4E-05	5.4E-03	2.7E-01	3331.7	2895.9	3980.3	3097.9	2599.5	2689.4	7916	5206.7	6042.5	4551.8	4129.8	4376.8	No	No	No	alpha-glucan phosphorylase, H isozyme, putative, expressed
Os.6321.1.S1_at	LOC_Os01g63690	3	1.1E-03	6.4E-07	5.0E-03	6.7E-03	1.9E-04	2.7E-01	2994.4	2996.5	2910.4	783.1	951.6	804.2	1953.8	2511.4	1590.3	777.8	751.1	936.9	No	No	No	nematode-resistance protein, putative, expressed
Os.10541.1.S1_at	LOC_Os01g64000	11	1.0E-07	7.0E-08	1.2E-07	6.8E-06	4.6E-05	3.2E-04	436.6	402.9	513.3	70.9	33	60.7	76.9	46.6	34.9	7.8	33.9	51.4	No	No	No	ABA response element binding factor, putative, expressed
OsAffx.2403.1.S1_at	LOC_Os01g64440	4	8.4E-06	2.1E-05	1.0E-04	1.6E-04	2.1E-03	2.5E-02	95.4	183.6	125.6	27.6	34.3	43.4	36	32.1	78.5	30	3.5	48.8	No	No	No	expressed protein
Os.12119.1.S1_at	LOC_Os01g64470	8	4.8E-04	1.4E-06	3.6E-04	3.5E-03	3.3E-04	5.6E-02	948.6	961.8	1013.2	190.3	305.1	314.8	478.2	544.4	522.1	337.7	304.2	313.8	No	No	No	YLS9, putative, expressed
OsAffx.23922.1.S1_at	LOC_Os01g65210	3	2.1E-05	9.5E-07	6.4E-04	3.1E-04	2.5E-04	8.1E-02	12.6	37.6	12.2	38	42.5	30.1	37.2	53.2	11.6	162.7	136.4	179.6	No	Yes	No	peptide transporter PTR2-B, putative, expressed
Os.9534.1.S1_at	LOC_Os01g65830	6	8.7E-05	3.0E-05	3.2E-05	9.3E-04	2.6E-03	1.2E-02	692.2	993.6	1372.3	3148.4	3937.4	4777.8	1360.5	1227.1	1413.6	1807.4	1374.6	1548.8	No	No	No	acyl-desaturase, chloroplast precursor, putative, expressed
Os.27932.1.S1_at	LOC_Os01g68580	5	6.5E-06	1.9E-06	1.8E-04	1.3E-04	4.1E-04	3.7E-02	123.9	145.5	162.9	931.7	694	942.3	327.9	484.9	385	3471	1911.7	1798.3	Yes	No	Yes	protease inhibitor/seed storage/LTP family protein, expressed
Os.7281.1.S1_at	LOC_Os01g68730	4	9.2E-09	3.4E-07	8.5E-05	1.4E-06	1.3E-04	2.2E-02	7570.2	9726.1	10021.1	3369.1	5041.4	5234.6	3855.2	4835.2	3710.6	1568.5	1914.3	2167.1	No	No	No	RNA-binding protein FUS, putative, expressed
Os.33625.3.S1_at	LOC_Os01g70840	6	1.2E-07	3.1E-06	3.2E-05	7.4E-06	5.7E-04	1.2E-02	86.7	91.1	90	140.2	111.3	114.6	216.7	272.6	249.6	498.9	487.1	603.3	No	No	No	esterase PIR7B, putative, expressed
Os.33625.5.S1_x_at	LOC_Os01g70840	6	9.7E-09	8.6E-07	5.5E-06	1.5E-06	2.4E-04	3.7E-03	347.9	263.6	222	429.9	299.5	351	445.7	453.6	444.3	735.3	778.7	847.8	No	No	No	esterase PIR7B, putative, expressed
Os.33625.4.S1_at	LOC_Os01g70850	11	4.2E-10	3.6E-12	1.2E-09	2.3E-07	2.4E-07	1.9E-05	19202.5	20146.7	19876.1	2017.3	1918.1	2062.1	8425.1	8584	8068.2	918.2	1079.7	930.6	No	No	No	esterase PIR7B, putative, expressed
Os.32596.3.S1_x_at	LOC_Os01g71624	10	7.9E-13	1.1E-12	1.1E-12	1.0E-08	1.1E-07	2.5E-07	1588.4	1665.5	1706.5	86.8	61.5	51.6	2.1	6.6	1.4	13.2	18.4	44.3	Yes	Yes	Yes	expressed protein
Os.18664.2.S1_x_at	LOC_Os01g71810	3	2.7E-07	1.4E-04	1.8E-05	1.3E-05	7.4E-03	8.3E-03	2111.3	1215.9	1557.5	1057.2	779	829	466.6	490.9	435.5	645.7	442.7	537.6	Yes	Yes	No	glucan endo-1,3-beta-glucosidase GV, putative, expressed
Os.12952.1.S1_at	LOC_Os01g71860	11	7.6E-11	7.0E-11	1.7E-10	8.6E-08	1.1E-06	6.0E-06	6363.3	9594.7	7260.8	763.4	1011.4	824.9	53.7	95.8	65.5	37.7	104.9	4.4	No	Yes	Yes	glycosyl hydrolases family 17 protein, expressed
Os.12279.1.S1_a_at	LOC_Os01g72009	3	2.8E-10	7.9E-13	1.5E-07	1.7E-07	1.1E-07	3.8E-04	17465.6	18745.3	17321.5	11560.1	9286.2	13187.7	17869	15441	17365.8	10035.3	9632	10700	No	No	No	expressed protein
Os.12279.2.S1_a_at	LOC_Os01g72009	5	4.9E-08	1.1E-10	1.7E-05	4.2E-06	1.4E-06	7.9E-03	10376	11733.8	10406.9	3770.5	4816.5	3896.1	7281.9	8751	8104.3	3577.2	3474.1	3726.3	No	No	No	expressed protein
Os.24890.1.S2_at	LOC_Os01g72120	9	3.2E-07	3.0E-08	2.5E-05	1.5E-05	2.8E-05	1.0E-02	8699.4	9634.7	9852.2	2677.9	2920.8	3277.5	4446	5144.3	5037.4	1358.3	1409.9	1713.1	No	No	No	glutathione S-transferase, putative, expressed
Os.22957.1.S1_at	LOC_Os01g72140	11	2.1E-10	3.8E-10	9.1E-10	1.5E-07	2.2E-06	1.5E-05	6439.6	6202.8	6740	594.5	636.3	654.5	3122.3	3663.3	3509.2	162.2	182.9	133.2	No	No	No	glutathione S-transferase, putative, expressed
OsAffx.24011.1.S1_at	LOC_Os01g72150	8	2.5E-07	1.7E-10	1.4E-07	1.2E-05	1.6E-06	3.7E-04	6886.3	5842.9	6716.9	290.3	400.1	290.3	2160.8	3322.1	3207.3	592.3	360.1	453.4	No	No	No	glutathione S-transferase, putative, expressed
Os.24906.1.S1_at	LOC_Os01g72460	7	2.3E-05	8.7E-08	4.7E-05	3.3E-04	5.4E-05	1.5E-02	1173.6	1107.4	1160.8	293	311.6	298.9	517.9	618.4	637.3	234.3	277.5	257	Yes	No	No	NADPH quinone oxidoreductase 2, putative, expressed
Os.18922.2.S1_at	LOC_Os01g72530	4	2.5E-04	7.9E-07	3.1E-04	2.1E-03	2.2E-04	5.1E-02	1889.8	3353.9	3063.1	313.5	1027.9	731.8	1220.4	2171.7	1263.4	800.5	426.2	609.8	No	Yes	Yes	calmodulin-like protein 41, putative, expressed
Os.28573.1.S1_at	LOC_Os01g74490	4	3.3E-07	1.3E-04	1.0E-04	1.5E-05	7.2E-03	2.5E-02	388.1	439.4	281.8	281.8	228.6	186.8	72.1	61.1	30	45.2	93.1	75.6	Yes	Yes	Yes	metal ion binding protein, putative, expressed
Os.49093.1.S1_a_at	LOC_Os02g02170	6	7.1E-06	7.2E-07	3.0E-05	1.4E-04	2.1E-04	1.2E-02	361.3	236.6	243.6	527.1	525.9	537.1	386.7	379.9	385	1214.2	1169.3	1606.1	No	Yes	Yes	high affinity nitrate transporter, putative, expressed
Os.49093.1.S1_at	LOC_Os02g02170	9	7.5E-06	2.4E-05	2.8E-05	1.4E-04	2.3E-03	1.1E-02	1.8	7.9	6.4	2.1	41.5	2.6	158.1	179.8	212.6	1279.8	997.6	1709.5	No	Yes	Yes	high affinity nitrate transporter, putative, expressed
Os.49093.2.S1_x_at	LOC_Os02g02190	3	3.5E-05	6.4E-06	2.1E-04	4.6E-04	9.1E-04	4.0E-02	101.9	124	104.8	152.9	317.5	236.9	115.8	88.5	98.7	398.2	363.8	524.2	No	No	No	high affinity nitrate transporter, putative, expressed
Os.2370.1.S1_at	LOC_Os02g02560	3	7.4E-05	7.6E-06	7.7E-04	8.1E-04	1.0E-03	9.1E-02	142.8	169.3	127.2	228.5	208	203.5	91.1	98.5	140.2	409	301.8	311.7	No	No	No	UTP--glucose-1-phosphate uridylyltransferase, putative, expressed
Os.6263.1.S1_at	LOC_Os02g03280	9	1.4E-08	2.7E-08	5.1E-07	1.9E-06	2.6E-05	8.3E-04	15651.5	16914.5	16510.1	9517.3	9374.3	10797.4	11002.2	10382.5	11271.7	9268.6	8133.1	9843.8	Yes	No	No	bax inhibitor 1, putative, expressed
Os.12228.1.S1_at	LOC_Os02g04160	3	2.7E-08	8.0E-09	6.8E-06	2.8E-06	1.2E-05	4.3E-03	2158.5	1597.4	1862.5	773	840.9	997.6	958.4	822.5	992	474.5	554.6	575.1	No	No	No	transcription elongation factor 1, putative, expressed
Os.52219.1.A1_at	LOC_Os02g04640	6	2.0E-07	3.3E-06	1.1E-05	1.1E-05	5.9E-04	6.2E-03	1385.7	1594.1	1437.7	584.7	784.2	761.2	346.6	468.3	537.8	354.7	382.5	456.2	No	No	No	transfactor-like protein, putative, expressed
Os.37946.1.S1_at	LOC_Os02g05830	4	3.0E-05	3.4E-05	1.2E-03	4.1E-04	2.9E-03	1.2E-01	3668.6	2902.7	3623.6	2514.4	2856.2	2652.9	5767.2	4964.3	6142.5	2572	2763	2843.4	No	No	No	ribulose bisphosphate carboxylase small chain SSU40B, chloroplast precursor, putative, expressed
OsAffx.2516.1.S1_at	LOC_Os02g05970	5	1.7E-05	5.6E-05	5.3E-05	2.7E-04	4.1E-03	1.6E-02	158.9	170.7	153.8	52.8	39.2	25.3	0.8	4.9	1.6	31.2	39.6	3	Yes	Yes	Yes	phytosulfokine receptor precursor, putative, expressed
Os.5009.1.S1_at	LOC_Os02g06390	6	1.2E-04	2.2E-05	6.6E-04	1.2E-03	2.2E-03	8.3E-02	1060	1038.4	894.9	585.7	470.9	577.5	655.5	584.1	562.3	465.5	474.9	483.8	No	No	No	expressed protein
OsAffx.24135.1.S1_at	LOC_Os02g06660	4	2.7E-06	4.1E-08	1.9E-06	6.7E-05	3.3E-05	1.9E-03	34.3	30.9	2.9	71.4	46.8	61.7	2.3	2.7	2	125.1	116.3	103.5	No	No	No	desiccation-associated protein, putative
Os.22940.1.S1_at	LOC_Os02g07010	4	2.8E-05	7.9E-06	3.2E-04	3.9E-04	1.1E-03	5.2E-02	1925.7	1886.2	1543.4	1054.1	971.7	871.7	937.8	1180.4	1057.7	787.9	835	931.7	No	No	No	expressed protein

Probe Set ID	Locus ID	probe #	ANOVA p-value			FDR q-value			Nipponbare MV			Nipponbare CK			93-11 MV			93-11 CK			Indel (Indica vs Japonica)			Annotation
			C	T	C×T	C	T	C×T	Repeat 1	Repeat 2	Repeat 3	Repeat 1	Repeat 2	Repeat 3	Repeat 1	Repeat 2	Repeat 3	Repeat 1	Repeat 2	Repeat 3	upstream	gene	downstream	
Os.11995.1.S1_at	LOC_Os02g07160	3	1.1E-03	4.4E-07	3.8E-04	6.4E-03	1.5E-04	5.9E-02	2821.3	2868.9	1679.2	1402.8	1590.1	1176.9	1442.7	1798.4	1270.8	985.1	1414.7	894.5	No	Yes	No	4-hydroxyphenylpyruvate dioxygenase, putative, expressed
Os.4769.1.S1_at	LOC_Os02g08230	3	6.4E-05	1.7E-03	1.0E-03	7.3E-04	4.4E-02	1.1E-01	870.1	523.9	662.4	882.3	612.3	560.6	1204.1	1209.6	1217.3	665.4	850.2	883.6	No	No	No	gl1 protein, putative, expressed
Os.53919.1.S1_at	LOC_Os02g09220	4	3.3E-10	7.9E-04	9.6E-04	2.0E-07	2.5E-02	1.0E-01	9181.8	10935.2	11652.6	13900.9	15182.5	15038.8	6	43.4	43.2	234.9	76.5	27.2	Yes	Yes	Yes	cytochrome P450 71D8, putative, expressed
Os.25128.1.S1_at	LOC_Os02g09810	8	4.0E-05	2.0E-08	2.1E-05	5.1E-04	2.2E-05	9.2E-03	3345.3	2895.1	3765.5	1369.7	1310.4	1639.6	2343.2	2339.8	2266.3	1421.4	1301.4	1431.7	No	No	No	amino acid transporter-like protein, putative, expressed
OsAffx.11997.1.S1_at	LOC_Os02g12070	8	2.0E-07	1.4E-05	1.6E-05	1.1E-05	1.6E-03	7.6E-03	426.1	372.8	401.9	176.7	201.3	172.5	62.5	70.9	63.1	91	63	78.2	Yes	No	No	expressed protein
Os.20204.2.S1_a_at	LOC_Os02g12350	4	1.0E-07	9.5E-07	1.4E-06	6.7E-06	2.5E-04	1.6E-03	330.3	412.2	375.6	107.8	147.9	84.3	90	125.1	170.8	42.8	46.6	30.2	Yes	Yes	Yes	histone deacetylase, putative, expressed
Os.20204.1.S1_s_at	LOC_Os02g12380	10	6.3E-09	9.4E-09	2.8E-07	1.1E-06	1.3E-05	5.7E-04	703.2	648.1	588.7	126.9	166.4	139.2	128.8	149.1	174	12.6	4.7	7.2	No	Yes	Yes	histone deacetylase, putative, expressed
Os.20204.1.S1_x_at	LOC_Os02g12380	5	3.7E-06	5.3E-06	7.6E-05	8.4E-05	8.1E-04	2.1E-02	52.6	49.6	52	38.5	56.4	26.2	16.5	47.8	13.7	20.7	12.1	33.3	No	Yes	Yes	histone deacetylase, putative, expressed
Os.20204.3.S1_a_at	LOC_Os02g12380	11	1.0E-09	3.4E-08	3.6E-08	3.8E-07	3.0E-05	1.6E-04	1015.9	1018	918.5	175.4	285.8	305.9	3.1	16.5	15	1.9	4.4	3.7	No	Yes	Yes	histone deacetylase, putative, expressed
Os.57132.1.S1_at	LOC_Os02g12900	5	5.4E-11	6.6E-09	6.6E-09	7.0E-08	1.1E-05	5.4E-05	1604.6	1970.6	1666.6	608.8	640.8	424.4	660.5	832.5	869	219	218.8	335.8	No	No	Yes	cysteine synthase, putative, expressed
Os.20931.1.S1_s_at	LOC_Os02g13300	3	1.2E-03	4.4E-07	4.4E-05	7.0E-03	1.5E-04	1.4E-02	1562.8	1782.3	1316.5	660	716.3	565.1	1001.4	1160.6	966.6	694.3	843.1	765.3	Yes	Yes	No	Grave disease carrier protein, putative, expressed
OsAffx.2594.1.S1_at	LOC_Os02g13300	3	1.9E-03	5.3E-07	1.3E-04	9.9E-03	1.7E-04	2.9E-02	1263.2	1092.9	1165.2	523.3	471.3	384.6	749.2	875.2	848.7	525.1	626.3	632.2	Yes	Yes	No	Grave disease carrier protein, putative, expressed
Os.4170.1.S1_at	LOC_Os02g13380	10	1.1E-08	1.1E-08	4.1E-07	1.6E-06	1.5E-05	7.2E-04	10617.5	8658.1	9774.7	4514.6	3724.3	4251	4439.2	3849.2	4568.9	3178.6	2685.4	2977.2	Yes	No	No	early nodulin 93, putative, expressed
OsAffx.24234.2.S1_s_at	LOC_Os02g13780	5	1.6E-06	2.5E-06	1.4E-05	4.6E-05	4.9E-04	6.9E-03	122.9	156.7	94.4	21.4	28.7	43.5	48.3	34.9	40.1	9.8	31.8	24.7	No	No	No	receptor-like protein kinase precursor, putative
Os.11467.1.S1_at	LOC_Os02g13840	8	2.7E-06	1.8E-07	4.5E-05	6.7E-05	8.8E-05	1.5E-02	9592	10538.6	10360.7	6947.7	5728.2	6410.1	7917.9	7548.2	7656	6041.6	6173.3	6375.9	No	No	Yes	citrate synthase, glyoxysomal precursor, putative, expressed
Os.54037.1.S1_s_at	LOC_Os02g15700	4	1.7E-09	2.9E-04	1.1E-05	5.0E-07	1.3E-02	6.0E-03	9040	9576.9	8815.7	5643.6	6237.2	5986.3	2884.5	4165.1	3628.5	5437.9	5129.2	5928.3	Yes	No	Yes	expressed protein
Os.53610.1.S1_at	LOC_Os02g16270	4	1.0E-09	1.3E-05	2.3E-05	3.7E-07	1.5E-03	9.6E-03	592.7	652.1	577.2	354.7	349.1	417.9	8.9	8.2	16.9	2.7	5.4	34.6	Yes	Yes	Yes	xa1-like protein, putative, expressed
OsAffx.24284.4.S1_x_at	LOC_Os02g16780	4	4.3E-04	5.8E-05	6.3E-04	3.2E-03	4.2E-03	8.0E-02	18.6	30.4	24.9	101.6	80.8	81.8	7.2	32.6	57.3	322.5	245.5	212.4	No	No	No	alpha-expansin 1 precursor, putative, expressed
OsAffx.24284.5.S1_at	LOC_Os02g16839	5	8.3E-05	1.4E-04	2.2E-05	8.9E-04	7.6E-03	9.4E-03	37.5	15.5	14.2	22.6	14.2	7.8	16.4	14.6	6.8	383.7	196	153.9	No	No	No	alpha-expansin 3 precursor, putative
Os.8089.1.S1_at	LOC_Os02g17240	4	2.4E-06	2.5E-08	1.1E-06	6.2E-05	2.4E-05	1.4E-03	51	29.4	24.8	67.8	103.7	110.1	45.4	21.6	26.8	152	158.2	161.1	No	No	No	pollen-specific kinase partner protein, putative, expressed
Os.9729.1.S1_a_at	LOC_Os02g17390	8	1.1E-05	8.5E-07	1.1E-04	1.9E-04	2.4E-04	2.7E-02	13927.2	14467	13743.5	9036.4	8101.8	8814.8	10753.2	10052.1	10699.3	9226.6	8237	9255.9	No	No	No	peroxisomal fatty acid beta-oxidation multifunctional protein, putative, expressed
OsAffx.12089.1.S1_at	LOC_Os02g17620	3	5.6E-07	5.8E-04	7.1E-04	2.2E-05	2.0E-02	8.7E-02	702.4	591.2	795.2	1835.3	1061.6	1799.7	7.1	10	4.5	41.4	29.6	7.5	No	Yes	Yes	expressed protein
OsAffx.24928.1.S1_at	LOC_Os02g17880	3	3.8E-05	6.8E-07	4.0E-05	4.9E-04	2.0E-04	1.4E-02	65.5	32.5	34.8	547.8	310.7	366	44.8	48.7	57.2	1288.6	676	1241.5	No	Yes	No	xyloglucan endotransglucosylase/hydrolase protein 26 precursor, putative
OsAffx.24297.1.S1_at	LOC_Os02g17900	8	1.1E-08	3.9E-09	1.1E-08	1.6E-06	8.2E-06	7.4E-05	16.8	6.9	9.2	141.1	63.9	99.9	46	3.5	12.3	399.7	329.1	399.7	No	No	No	xyloglucan endotransglucosylase/hydrolase protein 26 precursor, putative, expressed
Os.5314.1.S1_at	LOC_Os02g19770	7	2.7E-05	7.6E-06	1.2E-04	3.8E-04	1.0E-03	2.7E-02	3581.7	3399.3	3752.3	1878.4	1674.1	2256	2151.6	1958.1	2468.6	1648.3	1285.8	1722.7	No	No	No	eukaryotic translation initiation factor 1A, putative, expressed
Os.57017.1.S1_at	LOC_Os02g19990	4	1.3E-08	5.0E-04	3.2E-04	1.8E-06	1.8E-02	5.2E-02	526.5	446.9	468.9	646.6	759.2	611.9	55.2	42.9	69.8	65	49.9	57.9	No	Yes	Yes	reticulon family protein, expressed
OsAffx.2707.1.S1_at	LOC_Os02g21300	11	3.8E-06	6.0E-09	1.9E-06	8.7E-05	1.0E-05	1.9E-03	6383.5	4218.7	5018.8	108.6	96.9	70.4	1896.5	1665.8	2174.7	258.7	137.2	231.3	Yes	No	No	transposon protein, putative, unclassified, expressed
Os.27095.1.A1_at	LOC_Os02g21460	9	2.1E-07	8.9E-07	2.4E-05	1.1E-05	2.4E-04	1.0E-02	7433.3	9038.1	7109.7	3964.7	4221.8	3568.9	3171.6	3595.5	3055.7	2463.1	2655.8	2250.5	Yes	No	No	glutathione S-transferase, C-terminal-like, putative, expressed
Os.49731.1.S1_at	LOC_Os02g21920	4	7.6E-05	1.3E-06	2.9E-04	8.3E-04	3.1E-04	5.0E-02	4475.7	4000	3711.9	1768.4	2087.6	1681	2940.8	2784.5	2565	1795.3	2075	1697.9	No	No	Yes	senescence-associated protein, putative, expressed
Os.49731.1.S1_s_at	LOC_Os02g21920	4	1.5E-05	1.2E-06	1.8E-04	2.4E-04	2.9E-04	3.6E-02	5468	5626.2	6189.8	1686.4	1679.8	1869	2915.6	3223.1	3076.1	2065.7	1883.4	2523.8	No	No	Yes	senescence-associated protein, putative, expressed
Os.8901.1.S1_at	LOC_Os02g22130	3	3.3E-06	1.2E-05	1.0E-03	7.8E-05	1.5E-03	1.1E-01	3083.7	2829.6	3033.3	1712.6	1540.7	1735	1577.7	1366.3	1636	1458.3	831.3	1182.7	No	No	Yes	GTPase activating protein, putative, expressed
Os.8901.1.S2_at	LOC_Os02g22130	6	1.5E-08	2.3E-07	1.0E-04	1.9E-06	1.0E-04	2.5E-02	8216.5	9129.4	8962.8	5417.3	5978.1	6410.4	5813.5	5504.1	5211	5191.9	4489.1	4250.7	No	No	Yes	GTPase activating protein, putative, expressed
Os.53176.1.S1_at	LOC_Os02g26720	5	3.4E-06	1.7E-06	5.1E-06	8.0E-05	3.7E-04	3.5E-03	159.2	114.9	139.1	49.2	54.6	30.7	56.8	53.1	47.1	34.7	57.5	22.3	No	No	No	inositol-tetrakisphosphate 1-kinase 1, putative, expressed
Os.8902.1.S1_at	LOC_Os02g28220	8	7.4E-09	9.2E-07	8.0E-07	1.2E-06	2.5E-04	1.1E-03	451.9	409	437	234.5	234.5	156.9	18.7	57.9	6.4	68.4	44.7	40.9	No	Yes	Yes	transferase, putative, expressed
Os.14042.1.S1_at	LOC_Os02g30470	7	1.3E-09	1.0E-04	1.1E-04	4.4E-07	6.1E-03	2.7E-02	1.8	17.2	2.2	21.8	50.1	58.8	2425.5	1922.8	2093.9	3968	3147.8	3112.3	Yes	Yes	Yes	expressed protein
Os.9326.1.S1_at	LOC_Os02g30470	4	4.7E-10	7.7E-05	1.5E-04	2.4E-07	5.1E-03	3.3E-02	2706.2	2990	3066.6	3837.4	3492.9	3865.4	51.1	75.8	47.8	111.6	108	82	Yes	Yes	Yes	expressed protein
Os.49976.1.S1_at	LOC_Os02g30714	7	7.5E-08	2.6E-07	2.3E-07	5.5E-06	1.1E-04	5.1E-04	58.7	47.5	64	79.5	74.5	68.5	2.3	50.3	55.3	218.6	222.6	299.9	No	Yes	No	dehydrogenase/reductase SDR family member 7 precursor, putative, expressed
Os.52903.1.S1_at	LOC_Os02g30790	9	4.9E-09	1.0E-06	7.1E-07	9.6E-07	2.7E-04	1.0E-03	320.7	299.4	291.2	216.5	168.4	181.2	30.6	23.4	37.5	42.7	22.7	58.4	No	No	No	expressed protein
Os.56036.1.S1_at	LOC_Os02g31080	4	4.0E-07	7.3E-05	1.0E-04	1.7E-05	4.9E-03	2.5E-02	176.6	144.5	119.4	90.4	113.7	115.5	2.8	5.9	3.3	3	5.6	7.8	No	Yes	Yes	expressed protein
Os.49297.1.S1_at	LOC_Os02g31120	8	1.7E-07	3.2E-06	2.5E-06	9.7E-06	5.8E-04	2.3E-03	1181.7	739.4	726.5	266	294.9	291	13	22.8	62.4	51.3	38.3	14.3	Yes	No	No	retrotransposon protein, putative, unclassified
OsAffx.2825.1.S1_x_at	LOC_Os02g32140	4	2.0E-04	4.4E-07	1.6E-04	1.8E-03	1.5E-04	3.3E-02	1094.5	745.8	866.5	281.6	291.9	220.7	631.4	590.3	487.7	260.3	230.9	272.9	No	No	No	AP2 domain transcription factor, putative, expressed
OsAffx.30613.1.S1_x_at	LOC_Os02g32170	5	4.7E-07	6.1E-05	3.0E-04	1.9E-05	4.3E-03	5.0E-02	48.4	25.2	7.7	4.7	51.8	21.2	61.5	135.2	104.6	177.6	145.5	202.7	Yes	Yes	Yes	transposon protein, putative, unclassified
Os.50966.1.S1_at	LOC_Os02g32980	3	1.2E-10	3.7E-04	4.4E-04	1.1E-07	1.5E-02	6.4E-02	4795.9	4760.2	4745.5	3670.5	3503.2	3813.1	1592.6	2004.2	2088.6	1335.5	1706.8	2207.5	Yes	No	Yes	germin-like protein subfamily 3 member 2 precursor, putative, expressed
Os.9283.1.S1_at	LOC_Os02g33070	7	7.0E-10	7.4E-09	1.9E-08	3.1E-07	1.2E-05	1.0E-04	12221.6	12975.3	12247.8	1805.1	2070	2188.4	5206	5301.5	6287.1	2028.8	2256.1	2331.3	No	No	Yes	ATPase inhibitor, putative, expressed
Os.9416.1.S1_at	LOC_Os02g33110	6	6.9E-08	1.3E-09	1.8E-07	5.3E-06	4.1E-06	4.2E-04	1988.3	1636.3	1692.7	2904.1	2123.3	2589.5	2157.9	1847.8	1945.6	4159.2	4120.1	4199.2	No	Yes	No	beta-fructofuranosidase, insoluble isoenzyme 1 precursor, putative, expressed
Os.56326.2.S1_at	LOC_Os02g33780	5	2.0E-09	1.1E-09	7.4E-08	5.5E-07	3.6E-06	2.5E-04	222.1	187.6	166.7	489.6	497	419.8	73.2	69.3	86.9	163.9	152.6	23.3	No	Yes	Yes	NF-180, putative, expressed
OsAffx.12356.1.S1_at	LOC_Os02g35010	4	1.4E-07	7.5E-05	1.0E-04	8.5E-06	5.0E-03	2.5E-02	493.3	376.6	333.1	264	226	218.8	256.9	333.3	316.4	180.5	179.9	250.3	Yes	Yes	Yes	MAP3KA, putative, expressed
Os.50441.2.S1_x_at	LOC_Os02g35300	3	4.8E-05	2.1E-05	1.3E-04	5.9E-04	2.1E-03	2.9E-02	12.5	26.8	14.6	42	51.1	19.1	4.7	2.1	3.3	109	131.6	90	Yes	Yes	No	transposon protein, putative, unclassified, expressed
Os.3710.1.S1_at	LOC_Os02g35401	9	4.0E-07	1.8E-08	2.0E-06	1.7E-05	2.0E-05	1.9E-03	1189.9	1378.4	1151.5	201.9	129.5	196.9	225	195.1	266.3	110.1	88.8	77.7	Yes	Yes	Yes	zinc finger, C3HC4 type family protein, expressed
Os.54566.1.S1_at	LOC_Os02g35940	4	8.9E-08	7.9E-06	5.0E-04	6.2E-06	1.1E-03	7.0E-02	1369.6	1528.9	1567.2	784.3	758.8	626.2	787.2	874.3	748	437.8	539.8	524.5	No	No	No	gibberellin receptor GID1L2, putative, expressed
Os.7335.1.S1_at	LOC_Os02g36340	9	2.2E-08	4.7E-09	3.6E-07	2.5E-06	9.2E-06	6.7E-04	4697.9	5115.1	4754.7	1682.2	1769	2023	2705.1	3051.8	2743.7	1772.2	1236.2	1631.4	No	No	Yes	riboflavin biosynthesis protein ribAB, chloroplast precursor, putative, expressed
OsAffx.24603.1.S1_at	LOC_Os02g36810	7	2.2E-06	1.2E-05	9.1E-06	5.8E-05	1.4E-03	5.3E-03	30.3	12.3	27.3	47.2	24	44.5	206.1	266.9	253.2	76	76.2	164.2	Yes	No	Yes	cytokinin-O-glucosyltransferase 2, putative
OsAffx.24603.1.S1_x_at	LOC_Os02g36810	7	3.0E-07	9.8E-07	2.7E-06	1.4E-05	2.6E-04	2.4E-03	95.2	66.9	96.1	63.2	62.4	49.9	402.3	396.1	383.8	93.7	103.4	162	Yes	No	Yes	cytokinin-O-glucosyltransferase 2, putative
Os.55640.1.S1_at	LOC_Os02g37000	5	1.3E-05	2.1E-07	2.9E-04	2.2E-04	9.5E-05	4.9E-02	1695.9	2577.3	1698.5	483.9	652.2	495.9	886.4	971.5	803.1	566.7	481.5	405.5	No	No	Yes	mitochondrial prohibitin complex protein 1, putative, expressed
Os.11315.1.S1_at	LOC_Os02g37250	11	8.3E-09	1.3E-07	3.7E-07	1.3E-06	6.9E-05	6.7E-04	1023.4	924.1	1048.7	151.5	234.6	217.8	59.6	67.9	64.1	2.1	7.3	6	Yes	No	No	expressed protein
Os.20238.1.S1_s_at	LOC_Os02g37260	8	1.5E-08	4.3E-06	4.4E-06	2.0E-06	7.0E-04	3.3E-03	243.8	315.8	280.5	92.1	115.5	155.4	10.3	4.4	13.6	14.6	19.9	2.3	Yes	Yes	Yes	expressed protein
Os.54116.1.S1_at	LOC_Os02g37309	7	1.9E-07	1.8E-05	2.4E-05	1.0E-05	1.9E-03	1.0E-02	510.9	440.7	568.2	179.6	200.5	254.6	73.3	25.8	12.7	18.1	12.8	46.3	Yes	Yes	Yes	expressed protein
OsAffx.24613.1.S1_at	LOC_Os02g37320	10	2.6E-08	7.2E-06	5.9E-06	2.7E-06	1.0E-03	3.9E-03	672.5	820.1	757.4	235.4	324.2	298.7	0.6	1.2	0.7	13.9	1.1	8.3	No	Yes	Yes	ATFP4, putative, expressed
OsAffx.2891.1.S1_s_at	LOC_Os02g37330	5	5.4E-07	1.3E-03	3.5E-03	2.1E-05	3.5E-02	2.2E-01	852.7	944.8	864.3	313.1	622.3	538.4	51.7	106.6	82.2	54.5	3.3	63.8	No	No	No	ATFP4, putative, expressed
OsAffx.24629.1.S1_at	LOC_Os02g38230	5	7.3E-06	5.9E-07	1.0E-04	1.4E-04	1.8E-04	2.5E-02	439.5	267.1	290.1	1077.7	791	901.3	703.7	558.3	668.4	1488.2	1819.2	1824.3	No	Yes	No	component of high affinity nitrate transporter, putative, expressed
Os.51991.1.S1_at	LOC_Os02g38392	6	5.8E-09	2.2E-04	2.4E-04	1.1E-06	1.0E-02	4.4E-02	464.2	515.3	392.1	249.9	310.8	289.3	19.4	23.4	2.2	12.1	5.4	1.7	Yes	Yes	Yes	NBS-LRR disease resistance protein, putative, expressed
Os.1433.2.S1_at	LOC_Os02g40330	4	1.6E-04	1.0E-03	1.0E-03	1.5E-03	3.1E-02	1.1E-01	210.8	141.8	140.1	49.8	39.8	40.6	7.9	17.2	30.9	20.7	63.2	24.3	Yes	Yes	Yes	expressed protein
OsAffx.2928.1.S1_at	LOC_Os02g40500	9	4.9E-07	1.3E-07	1.2E-06	2.0E-05	7.1E-05	1.4E-03	1284.1	790.6	1082.9	259.7	197.2	169	242.9	219.8	354.6	157	80.7	133.9	Yes	No	No	OsGrx_C2.1 - glutaredoxin subgroup I, expressed
OsAffx.12463.1.S1_at	LOC_Os02g42820	11	3.3E-08	1.3E-08	3.9E-08	3.2E-06	1.6E-05	1.6E-04	8.7	1.6	37	68.9	47.7	42	19.3	27.9	22.9	424.7	338.6	389.9	No	No	No	pollen-specific protein SF3, putative, expressed
Os.51823.1.S1_at	LOC_Os02g43430	4	2.3E-05	3.3E-06	8.3E-05	3.4E-04	5.9E-04	2.2E-02	684.3	756.6	681	288.6	389.9	382.4	393.7	410.1	428.7	248.3	432.3	352	No	No	No	protein kinase APK1B, chloroplast precursor, putative, expressed
Os.22624.1.A1_at	LOC_Os02g43924	4	1.1E-08	1.7E-05	1.9E-04	1.6E-06	1.8E-03	3.8E-02	205	266.4	214.5	297.8	276.8	366.6	747.2	828.8	927.9	1222.5	1463.5	1718.1	No	No	No	expressed protein
OsAffx.24719.1.S1_at	LOC_Os02g44200	10	3.6E-09	4.8E-09	6.1E-09	7.9E-07	9.2E-06	5.3E-05	76.3	62	56.6	144.6	96.5	65.5	85.4	69.6	58.5	566.2	642.5	542.3	No	No	Yes	pollen thioesterase, putative
Os.52855.1.S1_at	LOC_Os02g44470	6	1.3E-06	8.0E-08	2.6E-06	3.9E-05	5.1E-05	2.3E-03	135.2	158.9	158.4	1068.4	568.7	903.2	150.1	160.3	160.5	2679.7	1785.4	2165.5	No	No	Yes	actin-depolymerizing factor, putative, expressed
Os.4833.1.S1_at	LOC_Os02g44500	3	9.6E-08	5.2E-04	8.5E-04	6.5E-06	1.9E-02	9.7E-02	4457.3	5544.8	4620.3	3126.6	4097.3	2837.7	1818.1	2522.5	2349.3	1846.6	2333.4	2507.4	Yes	No	No	phospholipid hydroperoxide glutathione peroxidase, putative, expressed
Os.26517.1.S1_at	LOC_Os02g44990	11	2.4E-06	3.1E-07	7.5E-06	6.2E-05	1.2E-04	4.6E-03	2188.3	1488.9	1876.5	139.6	301.5	177.3	363.4	495.8	311.2	123.7	97	100.9	No	No	Yes	F-box domain containing protein, expressed
Os.7396.2.S1_a_at	LOC_Os02g47580	3	1.6E-03	1.1E-07	1.1E-04	8.7E-03	6.3E-05	2.6E-02	2171.8	2190.5	2237.5	958.7	734.1	1029.4	1611.1	1587	1546	1019.1	1018.7	800.1	No	No	No	expressed protein
Os.16068.3.S1_at	LOC_Os02g47830	11	5.1E-07	1.2E-08	1.5E-06	2.1E-05	1.5E-05	1.6E-03	2163.4	2015.7	2088.6	684.6	618.5	675.3	1024.1	935.7	1045.1	589	464.7	588	Yes	No	No	expressed protein
Os.19315.1.S1_s_at	LOC_Os02g49650	4	6.9E-07	4.4E-07	8.1E-06	2.5E-05	1.5E-04	4.9E-03	42.1	68.4	38.4	83	175.1	97.2	125.4	127.7	109.2	351.4	296	351.6	No	No	No	expressed protein
Os.10379.1.S1_at	LOC_Os02g49860	5	2.0E-08	5.3E-07	5.1E-07	2.3E-06	1.7E-04	8.3E-04	280.2	169.1	191.9	75.1	119.3	82.4	46.8	45.6	44	27.9	21.5	51.9	No	No	No	ABA induced plasma membrane protein PM 19, putative, expressed
OsAffx.30013.3.S1_at	LOC_Os02g49930	4	5.9E-06	6.4E-06	1.3E-05	1.2E-04	9.2E-04	6.9E-03	102.4	77.7	57.5	10.4	15.2	18.3	25.4	26	23	0.8	0.8	0.8	No	No	No	retrotransposon protein, putative, Ty1-copia subclass
OsAffx.30013.3.S1_x_at	LOC_Os02g49930	4	1.6E-04	3.1E-05	1.6E-04	1.4E-03	2.7E-03	3.4E-02	159.5	106.7	122.6	32.2	23.3	21.2	31	10.4	29.1	10.2	1.4	14.3	No	No	No	retrotransposon protein, putative, Ty1-copia subclass
Os.54038.1.S1_at	LOC_Os02g50330	7	7.3E-06	4.9E-05	9.7E-05	1.4E-04	3.7E-03	2.4E-02	318.5	131.3	132.2	185.7	177.8	197.4	477.1	566.9	608.5	262.9	206	240.8	No	No	No	RNA-directed RNA polymerase 2, putative, expressed
OsAffx.12575.1.S1_at	LOC_Os02g50330	7	1.2E-05	3.5E-05	5.9E-05	2.0E-04	3.0E-03	1.8E-02	197.7	164.1	113.3	140.2	178.5	128.3	328.3	572.5	508.5	158.2	215.2	190.4	No	No	No	RNA-directed RNA polymerase 2, putative, expressed
OsAffx.24811.1.S1_at	LOC_Os02g50450	3	2.2E-06	2.5E-07	8.9E-06	5.9E-05	1.1E-04	5.2E-03	114.6	73.6	82.9	83	70.2	70.7	36.7	122.4	70.7	44.6	67.2	77.9	Yes	Yes	Yes	hypothetical protein
Os.8195.1.S1_at	LOC_Os02g50700	11	3.9E-07	5.1E-06	4.3E-05	1.7E-05	7.9E-04	1.4E-02	2783.9	2693.9	3211	1447.6	1365.6	1637.4	1058.2	1228.3	1059.9	1028.2	869.6	1032.1	No	No	Yes	expressed protein
Os.55360.1.S1_at	LOC_Os02g50710	9	2.1E-07	1.3E-06	2.4E-05	1.1E-05	3.2E-04	1.0E-02	1780	1554.2	1845.2	491	518.1	663.9	280.8	438.8	461.5	208.8	150.2	188	Yes	No	No	expressed protein
Os.52263.1.S1_at	LOC_Os02g50730	9	4.8E-06	8.4E-06	2.9E-04	1.0E-04	1.1E-03	4.9E-02	2855.8	2876.6	3512.5	770.8	1048.5	1169.2	997.2	1178.4	1131.9	849	489.3	719.4	No	No	No	metalloendoproteinase 1 precursor, putative, expressed
Os.53614.1.S1_at	LOC_Os02g51930	11	1.6E-10	2.1E-11	8.1E-10	1.2E-07	5.8E-07	1.5E-05	3968.2	3513.7	3674.3	664.9	483.4	496	841.9	742.5	903.4	472	379.1	331	Yes	No	Yes	cytokinin-O-glucosyltransferase 2, putative, expressed
Os.12240.1.S1_at	LOC_Os02g52010	6	2.1E-06	8.0E-05	2.6E-04	5.7E-05	5.2E-03	4.6E-02	776.7	621.8	573.7	312.7	367.8	300.9	264.9	367.8	287.8	324.8	215	272.8	Yes	No	Yes	phi-1-like phosphate-induced protein, putative, expressed
Os.55764.1.S1_at	LOC_Os02g52590	3	1.7E-03	2.5E-05	2.5E-03	8.9E-03	2.4E-03	1.8E-01	56.7	63.8	62.7	194	122.4	132	77.8	112.1	70.9	339.2	272	276	No	No	No	fucosyltransferase 8, putative, expressed
Os.54146.1.S1_at	LOC_Os02g53130	3	3.3E-04	4.8E-08	1.5E-04	2.6E-03	3.6E-05	3.3E-02	1511.8	1572	1855.3	47.5	62.8	5.5	1023.3	1076.8	682.2	90	72.8	44.7	No	No	No	nitrate reductase, putative, expressed
OsAffx.24845.1.S1_at	LOC_Os02g53250	6	7.9E-08	3.5E-04	1.1E-04	5.7E-06	1.4E-02	2.6E-02	875.7	844.5	913.1	514.1	457.8	458.2	71.2	122.6	107.6	100.8	115.8	111.7	Yes	No	Yes	expressed protein
Os.16435.1.S1_at	LOC_Os02g53410	3	4.4E-05	1.2E-06	6.2E-04	5.5E-04	3.0E-04	8.0E-02	2730.2	2786.9	3164.3	1244.9	1210.1	1306.3	1666.2	1882.1	1851.5	1045.4	998.2	1195.3	Yes	No	No	expressed protein

Probe Set ID	Locus ID	probe #	ANOVA p-value			FDR q-value			Nipponbare MV			Nipponbare CK			93-11 MV			93-11 CK			Indel (Indica vs Japonica)			Annotation
			C	T	C×T	C	T	C×T	Repeat 1	Repeat 2	Repeat 3	Repeat 1	Repeat 2	Repeat 3	Repeat 1	Repeat 2	Repeat 3	Repeat 1	Repeat 2	Repeat 3	upstream	gene	downstream	
Os.12391.1.S1_a_at	LOC_Os02g54820	3	1.1E-07	9.6E-06	1.8E-04	7.2E-06	1.2E-03	3.7E-02	4778.3	5577.7	5519.6	2339.5	3501.6	2957	3263.9	3451.3	3275.9	2617.3	2556.1	2041.6	Yes	No	No	trehalose-6-phosphate synthase, putative, expressed
OsAffx.12645.1.S1_at	LOC_Os02g54870	4	7.0E-08	2.4E-04	1.0E-04	5.3E-06	1.1E-02	2.5E-02	15.1	9.4	8	7.6	29	4.6	252.5	251.6	338.7	106.2	200.4	155.5	No	No	No	expressed protein
OsAffx.12645.1.S1_s_at	LOC_Os02g54870	9	2.5E-08	1.7E-04	8.4E-05	2.7E-06	8.6E-03	2.2E-02	265.8	335.5	324.9	416	541.5	417.3	3331.5	3364.7	3682.1	1527.6	2619.3	1978.9	No	No	No	expressed protein
Os.51432.1.S1_at	LOC_Os02g54880	5	2.7E-05	1.4E-03	4.2E-04	3.7E-04	3.7E-02	6.2E-02	363.2	394.4	591.6	408.3	403.9	623	1085.5	1210.4	1249.5	407.9	600.6	732.4	No	No	No	cytochrome c oxidase subunit, putative
Os.53968.1.S1_at	LOC_Os02g56310	10	9.2E-10	6.7E-07	1.4E-06	3.5E-07	2.0E-04	1.6E-03	3737.5	4732.1	3692	1883.3	1731.9	1818.5	832.4	1532.3	1171.4	807.7	908.3	1128.7	Yes	No	Yes	calcium-dependent protein kinase, isoform AK1, putative, expressed
Os.29925.1.S1_at	LOC_Os02g56440	3	8.5E-10	2.9E-03	3.0E-03	3.4E-07	6.2E-02	2.0E-01	775	790.6	677.7	604	598.8	603.9	28.8	51.9	23	33.3	52.8	24.9	Yes	Yes	Yes	expressed protein
Os.20154.1.S1_at	LOC_Os02g56700	3	1.1E-09	1.7E-09	2.1E-09	4.0E-07	4.9E-06	2.4E-05	567.8	1146.8	911.1	4.9	145.8	137.3	257.5	242.4	308.1	5.5	6.9	4.6	Yes	Yes	Yes	dihydroflavonol-4-reductase, putative, expressed
Os.4154.1.S1_at	LOC_Os02g56850	10	2.3E-06	6.5E-07	1.0E-05	6.0E-05	2.0E-04	5.6E-03	9175.1	8560.5	9245.8	4793.8	3852.3	4767	5019.6	4350.6	5668.3	4340.3	3969.3	4704.6	No	No	No	glutathione reductase, cytosolic, putative, expressed
Os.8733.1.S1_at	LOC_Os02g57260	9	5.5E-07	3.1E-07	5.5E-04	2.1E-05	1.2E-04	7.4E-02	10875.6	10216.4	11052.1	7031.4	6073	7019.9	7782.4	7368.8	8132.9	6564.8	5305.2	6370.5	No	Yes	No	3-ketoacyl-CoA thiolase 2, peroxisomal precursor, putative, expressed
Os.55392.1.S1_at	LOC_Os02g57350	3	9.0E-07	3.0E-03	2.5E-03	3.0E-05	6.3E-02	1.8E-01	510.3	511	622	293.3	385.6	348.9	6	6.8	10.9	3.9	3	6.3	Yes	Yes	Yes	expressed protein
Os.9312.1.S1_at	LOC_Os03g01270	3	4.9E-05	3.6E-05	4.1E-03	5.9E-04	3.0E-03	2.4E-01	6569.1	7196	7724.8	11869.6	10135.6	11662	12213.8	11210.6	11305	13532.6	12860.9	12512	No	No	No	beta-expansin 1a precursor, putative, expressed
Os.9346.1.S1_at	LOC_Os03g01310	4	8.5E-11	1.1E-07	1.2E-04	9.0E-08	6.4E-05	2.8E-02	1880.6	2730.3	2419.6	5037.4	5425.1	5600.6	7141.8	8839.8	8090	13832.5	14156.5	14782.9	No	No	No	cortical cell-delineating protein precursor, putative, expressed
Os.40007.1.S1_x_at	LOC_Os03g02874	10	4.6E-07	1.3E-06	1.3E-06	1.9E-05	3.2E-04	1.5E-03	588.4	712.2	647.4	85.1	145.6	84.9	31.7	47.9	34.9	27.3	54.4	31.2	No	No	Yes	retrotransposon protein, putative, Ty3-gypsy subclass, expressed
Os.26406.1.S1_at	LOC_Os03g03450	5	8.7E-09	1.1E-06	5.0E-06	1.4E-06	2.8E-04	3.5E-03	3617.6	3573.5	2865.5	1276.6	1552	1481.7	833.9	1378	1107.5	693	804.5	967.9	Yes	No	No	anthranilate phosphoribosyltransferase, chloroplast precursor, putative, expressed
Os.26406.2.S1_x_at	LOC_Os03g03450	9	5.4E-08	5.0E-07	3.7E-05	4.4E-06	1.7E-04	1.3E-02	2337.2	2142.2	1962.3	1058	1086.8	920.3	840.1	945.2	692.8	490.4	603	354.9	Yes	No	No	anthranilate phosphoribosyltransferase, chloroplast precursor, putative, expressed
Os.49527.1.S1_at	LOC_Os03g03790	4	3.8E-07	1.9E-08	2.1E-05	1.7E-05	2.1E-05	9.1E-03	2154.3	1686.3	2338.1	735.1	729.3	930.8	1069.3	1080.6	967.5	449.3	599.5	452.1	No	No	No	acyl-activating enzyme 11, putative, expressed
Os.7192.1.S1_at	LOC_Os03g04410	8	6.6E-07	4.7E-08	2.2E-04	2.4E-05	3.6E-05	4.1E-02	11211.5	11364	10793.7	6510	5474.1	6376.1	9158.1	8964.7	8249.2	6750.5	6231.3	6349.6	Yes	No	Yes	aconitate hydratase, cytoplasmic, putative, expressed
Os.56054.1.S1_at	LOC_Os03g05700	11	3.1E-09	1.7E-09	3.6E-09	7.3E-07	4.9E-06	3.5E-05	4242	4050.6	4238.2	139.6	146	178.4	421.4	422	392.8	104.7	125.1	114.5	No	No	No	expressed protein
Os.8947.1.S1_at	LOC_Os03g05910	11	4.6E-09	1.6E-10	7.6E-09	9.2E-07	1.6E-06	5.9E-05	10256	11076	10310.8	554.5	603.7	486.7	3897	3889.4	4817.3	550.9	499.1	360.2	No	No	No	monooxygenase, putative, expressed
Os.4891.1.S1_at	LOC_Os03g06200	4	7.5E-06	6.1E-06	1.2E-03	1.4E-04	8.9E-04	1.2E-01	8786.2	9824.8	9310.4	5031.7	4790.5	5241.5	5680.5	5854.4	5568.7	4462.3	4075.5	3744.9	No	No	No	phosphoserine aminotransferase, chloroplast precursor, putative, expressed
Os.51791.1.S1_at	LOC_Os03g06970	6	1.1E-06	5.0E-06	4.6E-06	3.6E-05	7.8E-04	3.3E-03	327.5	323.8	283.5	167.4	140.2	86.6	67.2	98.1	72	55.4	48.9	48.6	No	No	No	pollenless3, putative, expressed
Os.10356.1.S1_at	LOC_Os03g08320	10	2.3E-06	1.0E-06	7.8E-06	5.9E-05	2.7E-04	4.8E-03	5050.6	5052.4	5224.8	1669	1260.8	1475.5	1511.8	1865	1807.1	1564.5	1029.2	1277.1	No	No	No	pnFL-2, putative, expressed
OsAffx.12780.1.S1_at	LOC_Os03g08410	3	2.8E-07	4.5E-06	3.7E-06	1.3E-05	7.3E-04	2.9E-03	144.4	115.1	125.1	48.1	55.4	70.9	24.5	11.8	7	19.8	74.1	41.4	Yes	No	No	monooxygenase/ oxidoreductase, putative, expressed
OsAffx.12783.1.S1_at	LOC_Os03g08530	7	9.2E-06	1.2E-06	3.4E-05	1.7E-04	3.0E-04	1.2E-02	475.8	468.9	462.2	121.9	101.5	91.4	160.4	300.7	250.4	106.9	151.4	151.6	Yes	No	Yes	alanine aminotransferase 2, putative, expressed
Os.32298.1.S1_at	LOC_Os03g08880	4	1.2E-06	4.7E-05	6.3E-04	3.8E-05	3.6E-03	8.1E-02	516.4	887.1	649.5	378	424.9	360.4	147.5	117.1	175.1	130.8	104	70.1	No	No	No	ATPUP3, putative, expressed
Os.43892.1.S1_at	LOC_Os03g09070	3	1.4E-05	1.6E-07	1.9E-05	2.2E-04	8.1E-05	8.5E-03	237.8	244.9	321.1	49.6	89.8	19.9	87.9	141.6	93.3	38.2	86.2	6.5	No	No	No	protein binding protein, putative, expressed
Os.20151.1.S1_at	LOC_Os03g09900	5	9.7E-08	1.7E-04	2.4E-03	6.5E-06	8.6E-03	1.8E-01	6493.2	6507.5	7144.8	4688.1	4682.2	5461.3	3750.8	3733.3	3446.7	3301.3	2952	3099.3	Yes	No	Yes	membrane protein, putative, expressed
Os.49776.1.S1_at	LOC_Os03g09910	10	1.0E-06	5.1E-06	1.6E-05	3.3E-05	7.9E-04	7.7E-03	1444.5	1459.3	1409.3	333	350.3	362.4	120.8	143.6	138.2	70.3	132.3	85.9	No	No	No	transaminase/ transferase, transferring nitrogenous groups, putative, expressed
Os.9086.1.S1_at	LOC_Os03g10210	3	2.2E-04	4.5E-04	1.6E-03	1.9E-03	1.7E-02	1.4E-01	527.4	500.9	464.3	187.3	362.1	218.7	331.2	462.7	501	495.3	418	346	Yes	No	No	DNA binding protein, putative, expressed
Os.15799.1.S1_at	LOC_Os03g11420	8	2.4E-06	1.2E-08	1.2E-05	6.1E-05	1.5E-05	6.4E-03	1382.6	1272.8	1465.7	3550.3	2877.9	3575.7	1337	1215.7	1431.3	2246	2009.3	2271.7	No	No	No	non-cyanogenic beta-glucosidase precursor, putative, expressed
Os.7678.1.S1_at	LOC_Os03g12500	5	3.6E-10	1.2E-07	3.7E-07	2.1E-07	6.6E-05	6.7E-04	614.9	739.7	713.6	353	309.8	333.4	119.5	148.7	203.9	131.5	106.1	88.4	Yes	No	Yes	cytochrome P450 74A2, putative, expressed
Os.8741.1.S1_at	LOC_Os03g12820	11	1.6E-06	2.6E-09	1.9E-06	4.5E-05	6.4E-06	1.9E-03	2135.8	1719.6	1840	270.1	353.7	299.7	988.2	1148.3	918.9	277.5	305.3	223.4	No	No	No	ATP8, putative, expressed
Os.50391.1.S1_at	LOC_Os03g13030	4	7.5E-08	4.3E-08	2.6E-04	5.5E-06	3.4E-05	4.6E-02	1677.5	1924.7	1951.2	659.2	962.9	662.2	834.2	1051.2	1173.9	484.5	528.6	588	No	No	No	1-O-acylceramide synthase precursor, putative, expressed
Os.12191.1.S1_at	LOC_Os03g13140	5	3.7E-05	1.6E-04	3.2E-03	4.8E-04	8.4E-03	2.1E-01	979.3	1388.9	1239.4	403.8	614.2	614.5	335.6	428.9	407.2	351.3	159.5	230.8	No	No	No	non-symbiotic hemoglobin 2, putative, expressed
Os.12191.1.S1_x_at	LOC_Os03g13140	3	6.2E-05	3.1E-04	1.1E-03	7.1E-04	1.3E-02	1.1E-01	734.8	927.8	1110.6	390.3	573.4	609.6	353.5	321.4	404.7	348.1	198.3	254.9	No	No	No	non-symbiotic hemoglobin 2, putative, expressed
Os.159.1.S1_s_at	LOC_Os03g13210	3	2.5E-04	1.1E-04	7.3E-03	2.1E-03	6.5E-03	3.2E-01	1307	1245.7	1102.5	722.3	882.6	607.6	788	885.6	916.9	677.4	643.1	609.5	No	No	No	peroxidase N precursor, putative, expressed
Os.49217.1.S1_at	LOC_Os03g14300	5	1.3E-05	8.5E-05	5.1E-05	2.2E-04	5.4E-03	1.6E-02	2636.6	1513.5	1689	438.9	434.5	547	458.5	339.4	371.5	561	304.3	386.3	No	No	No	expressed protein
Os.49495.2.S1_at	LOC_Os03g15250	3	3.3E-06	9.4E-06	5.7E-06	7.7E-05	1.2E-03	3.8E-03	198.7	172.4	145.5	79.6	76.3	36.6	41.5	81.1	84.3	61.1	72.7	53.1	No	No	No	lectin-like receptor kinase 7, putative
Os.53936.1.S1_at	LOC_Os03g15270	9	1.3E-05	7.7E-07	2.4E-05	2.2E-04	2.2E-04	9.8E-03	1388.2	1488.6	1306.3	567.9	618.8	437.2	636.5	666.4	607.2	440.8	488.5	464.6	No	No	No	prMC3, putative, expressed
OsAffx.30103.1.S1_s_at	LOC_Os03g15370	6	4.7E-05	1.9E-05	3.3E-04	5.8E-04	1.9E-03	5.3E-02	325.9	303.5	250.5	81.6	84.9	49.8	122.6	113.2	110.9	100.2	69	111.2	Yes	No	No	ubiquitin, putative, expressed
Os.7114.1.S1_s_at	LOC_Os03g15780	10	3.6E-09	8.6E-09	8.4E-07	8.0E-07	1.3E-05	1.1E-03	1846.7	1629.6	1754.8	816.7	754.1	723.8	637.6	708.2	618.4	408.6	484	267.9	No	No	No	anthranilate synthase component I-1, chloroplast precursor, putative, expressed
OsAffx.25105.1.S1_at	LOC_Os03g16160	4	1.9E-03	2.4E-04	9.3E-04	9.9E-03	1.1E-02	1.0E-01	32.5	14.4	2.6	68.2	60.9	53.7	10.7	9.1	2.9	150.3	102.9	193.1	No	No	No	hypothetical protein
Os.56930.1.S1_at	LOC_Os03g17220	4	3.4E-06	8.3E-09	2.3E-05	8.0E-05	1.2E-05	9.7E-03	154.4	143.6	145.5	409.4	465.2	387.8	113.9	199.3	103.6	653	676.9	565	No	No	No	dirigent-like protein, expressed
Os.27671.1.S1_at	LOC_Os03g17470	5	5.3E-09	1.4E-05	2.7E-05	1.0E-06	1.6E-03	1.1E-02	5664.3	5929.9	5953.6	3063.1	3585.3	2935.6	2656.4	2579.9	2578.5	1812.2	1733	1769.8	No	No	No	IN2-1 protein, putative, expressed
Os.2376.1.S1_at	LOC_Os03g17480	4	4.7E-05	9.5E-07	1.9E-04	5.8E-04	2.5E-04	3.8E-02	3174.2	4550.3	3228.8	697.8	1046.6	633.5	3666.3	3946.3	4580.4	2289.5	1852.6	1731	No	No	No	IN2-1 protein, putative, expressed
Os.11313.1.S1_at	LOC_Os03g18850	3	8.6E-11	2.9E-05	9.8E-04	9.0E-08	2.6E-03	1.1E-01	11370.4	12576.7	11678.8	9477	11468.5	10332.9	2916.1	3707.7	4356.7	2958.9	2635.9	3881.5	No	No	No	pathogenesis-related protein 1, putative, expressed
Os.8742.1.S1_at	LOC_Os03g19250	4	3.6E-06	3.4E-06	7.2E-04	8.3E-05	6.1E-04	8.8E-02	9912.2	8462.8	10954.4	5592.5	3893	5141.2	4710.2	4914	5969	3090	3159.2	3292.6	No	Yes	Yes	AMP-binding protein, putative, expressed
Os.56274.1.S1_at	LOC_Os03g19700	5	5.8E-07	3.1E-06	4.3E-06	2.2E-05	5.7E-04	3.2E-03	76.9	56.4	64.5	64.6	70.9	62.9	79.1	76.2	142	256.8	221.4	220	No	No	No	expressed protein
Os.32659.1.S1_at	LOC_Os03g20020	3	8.0E-11	1.9E-04	1.6E-04	8.8E-08	9.3E-03	3.4E-02	1711	1900	1699.9	1276.7	1124.8	1184.3	952.3	932.8	979.1	616.8	1014.5	731.6	No	No	No	beta-fructofuranosidase, putative, expressed
Os.53074.1.S1_at	LOC_Os03g20330	9	4.0E-07	4.8E-09	9.9E-07	1.7E-05	9.2E-06	1.3E-03	1749.1	1991.4	1757.9	323	464.1	303	900.4	1032.8	891.4	193.7	271.6	358	No	No	No	VQ motif family protein, expressed
Os.51506.1.S1_at	LOC_Os03g20600	8	2.9E-08	4.0E-06	1.3E-04	2.9E-06	6.7E-04	3.0E-02	3909	3075.2	4284.8	2155.9	1252.2	1860.6	445.9	569.4	333.6	383.1	231.4	271.2	No	No	Yes	expressed protein
OsAffx.12954.1.S1_at	LOC_Os03g20680	5	4.9E-09	4.0E-05	2.5E-04	9.7E-07	3.2E-03	4.5E-02	623.9	1064.3	941.4	1114.3	930.6	1127.4	3112.3	3224.3	3911.5	5358.3	5937.6	5749.5	Yes	No	No	embryonic protein DC-8, putative, expressed
OsAffx.25172.1.S1_at	LOC_Os03g20840	6	3.8E-06	1.1E-04	1.0E-03	8.7E-05	6.6E-03	1.1E-01	593.9	579.8	625	211.7	295.1	330.7	199.8	164.6	198.7	189.3	168.6	170.8	No	No	Yes	disease resistance RPP13-like protein 1, putative, expressed
OsAffx.3286.1.S1_x_at	LOC_Os03g20949	7	1.6E-07	2.3E-05	1.0E-03	9.0E-06	2.2E-03	1.1E-01	1640.6	1804.2	1622.4	954.2	899.2	934.3	935.5	1095.1	926	805	743.5	836.1	Yes	No	No	phospholipid-transporting ATPase 1, putative, expressed
Os.12186.1.S1_at	LOC_Os03g21040	3	2.1E-09	4.4E-09	2.8E-07	5.8E-07	8.8E-06	5.7E-04	13219.7	12244.7	13180.6	14300.4	11397.7	14297.3	15490.7	14221.6	15838.6	18052.8	17926.7	16607	No	No	No	expressed protein
Os.56849.1.S1_at	LOC_Os03g22600	3	7.0E-07	6.3E-09	2.8E-06	2.6E-05	1.1E-05	2.4E-03	177.5	218.7	194.1	253.9	384.9	302	179.6	256.9	159.4	406.6	502.3	416.4	No	No	No	transposon protein, putative, Mutator sub-class, expressed
OsAffx.3313.1.S1_at	LOC_Os03g24690	3	6.2E-05	4.8E-06	4.3E-05	7.1E-04	7.5E-04	1.4E-02	72.2	71.3	43.7	32.5	30.2	7.9	52.5	69.7	114.2	23.8	4.2	15.5	Yes	No	Yes	terpene synthase 7, putative, expressed
Os.17424.1.S1_at	LOC_Os03g24730	3	8.3E-12	8.1E-04	8.3E-04	2.7E-08	2.6E-02	9.6E-02	16.3	3.5	0.9	9.3	33.1	1.2	1171.8	1319	1383	1512	1504.7	1746.7	Yes	No	Yes	expressed protein
Os.18713.1.S1_at	LOC_Os03g25030	10	1.5E-08	6.5E-06	6.1E-06	1.9E-06	9.3E-04	4.0E-03	2779.6	3076	3618.2	1019.7	1481.8	1384.4	35.5	28	37.2	54.6	75.5	49.5	Yes	Yes	Yes	esterase precursor, putative, expressed
Os.2965.1.S1_at	LOC_Os03g25280	10	7.0E-07	3.4E-07	1.1E-06	2.5E-05	1.3E-04	1.4E-03	138.3	259.5	330.9	4144.1	5525.1	6590.1	80.3	176.9	80.2	302.1	331.9	326.9	Yes	No	No	peroxidase 1 precursor, putative, expressed
Os.19737.1.S1_at	LOC_Os03g25300	6	6.9E-09	4.8E-09	5.3E-07	1.2E-06	9.2E-06	8.6E-04	499.6	594.8	580.2	1511.2	1705.7	2237.5	22.5	17	55	455.9	337.6	421.6	Yes	No	Yes	peroxidase 1 precursor, putative, expressed
Os.2957.1.S1_at	LOC_Os03g25330	6	6.6E-05	3.9E-07	4.6E-04	7.5E-04	1.4E-04	6.6E-02	5107.3	6124.5	6102.9	14580.1	12850.9	16139.8	5160.4	6885.2	4699.3	8591.5	9467.4	11959.8	No	No	No	peroxidase 66 precursor, putative, expressed
Os.57293.1.S1_at	LOC_Os03g25690	10	5.5E-09	8.6E-09	8.5E-09	1.0E-06	1.3E-05	6.2E-05	385.2	316.7	280.3	90.5	51.9	65.1	6.5	2.6	6	14.3	32.7	35	Yes	Yes	Yes	conserved hypothetical protein
Os.52886.1.S1_at	LOC_Os03g25750	5	2.9E-05	1.5E-04	4.6E-04	4.0E-04	7.8E-03	6.6E-02	1646.6	1605.9	1973.2	978.2	516.6	1170	822.5	770.5	1005	851.8	728.9	836.4	Yes	No	No	expressed protein
Os.45229.1.S1_at	LOC_Os03g28389	3	2.2E-08	7.6E-04	8.4E-04	2.5E-06	2.5E-02	9.6E-02	679.4	567.9	662.4	466.5	472.1	484.7	48.2	60.8	56.2	29.4	8.3	16.6	Yes	No	No	expressed protein
Os.52108.1.S1_at	LOC_Os03g30130	9	2.0E-10	5.8E-09	2.3E-08	1.4E-07	1.0E-05	1.2E-04	1146	1051.1	1174.2	389.8	498.3	457.6	403.8	419.2	403.2	308.1	259.6	388.5	No	No	No	phospholipase C, putative, expressed
Os.7354.1.S1_at	LOC_Os03g30200	3	6.8E-07	3.5E-07	9.6E-07	2.5E-05	1.3E-04	1.2E-03	155.5	198.1	120.7	40.6	43.8	40.5	114.4	103.6	72.7	16.3	88.1	54.6	Yes	No	No	mitochondrial import inner membrane translocase subunit Tim17, putative, expressed
Os.6998.1.S1_at	LOC_Os03g37490	8	7.0E-08	2.3E-07	9.7E-06	5.3E-06	1.0E-04	5.5E-03	5053.6	5582.6	5607.2	2286.6	1845.8	2060.6	2048.1	2431.4	2302.9	1251	1222.3	1187.7	Yes	No	No	transparent testa 12 protein, putative, expressed
Os.57170.1.S1_at	LOC_Os03g41130	10	4.8E-08	9.2E-10	4.7E-08	4.2E-06	3.5E-06	1.8E-04	57	31.6	31	416.7	299.2	368.2	79.9	33.2	43.5	1094.4	879.4	1428.1	No	No	Yes	expressed protein
Os.49538.1.S1_at	LOC_Os03g43010	3	2.5E-05	1.4E-07	1.9E-04	3.6E-04	7.4E-05	3.7E-02	268.2	302.4	254.5	638.9	414.4	542.1	284.8	381	330.2	764	1093.1	1290.5	No	No	No	expressed protein
OsAffx.25512.1.S1_s_at	LOC_Os03g43400	3	4.0E-06	4.2E-09	1.1E-04	9.0E-05	8.6E-06	2.7E-02	182.3	129.3	168.8	808.5	472.3	763.1	329.6	332.3	459.9	1148.6	1010.4	1117.9	Yes	Yes	No	OsIAA11 - Auxin-responsive Aux/IAA gene family member, expressed
OsAffx.13338.1.S1_at	LOC_Os03g44100	11	7.6E-09	8.1E-09	1.2E-08	1.3E-06	1.2E-05	7.8E-05	864.6	813.4	919.9	101.1	160.6	129.7	160.9	80.9	107.1	85.3	70.5	75.3	No	Yes	Yes	hydrolase-like protein, putative, expressed
OsAffx.25532.1.S1_at	LOC_Os03g44110	4	2.8E-05	5.8E-06	3.0E-05	3.8E-04	8.5E-04	1.1E-02	56.6	63.1	27.9	44.9	35.7	43.9	40.3	26	8.7	30.5	89	19.4	Yes	No	Yes	hydrolase-like protein, putative
Os.56896.1.S1_at	LOC_Os03g44170	3	6.9E-06	3.9E-05	1.7E-03	1.3E-04	3.2E-03	1.5E-01	263.2	416.2	358.1	172.8	171.6	268.5	261.9	249.6	303	105.2	76.6	134.1	Yes	Yes	Yes	glutathione S-transferase parA, putative, expressed
Os.459.1.S1_at	LOC_Os03g46070	3	6.2E-08	3.3E-06	8.2E-05	4.9E-06	5.9E-04	2.2E-02	517.1	669	578.9	213.6	266.3	352.2	144.4	243.5	175.5	58.2	114.5	109	No	No	No	protein P21, putative, expressed
Os.54959.1.S1_at	LOC_Os03g46860	3	4.0E-07	2.3E-07	5.9E-06	1.7E-05	1.0E-04	3.9E-03	93.7	104.8	128.9	161.3	141	98.6	79	122.4	99.1	188.3	222	221.1	No	No	No	DNA binding protein, putative, expressed
Os.32285.1.S1_at	LOC_Os03g49524	3	7.3E-06	3.7E-04	8.7E-04	1.4E-04	1.5E-02	9.8E-02	181.8	236.6	152.3	91.1	74.7	76.1	30.1	83.6	63.8	33.7	50	25.6	No	No	No	anthocyanidin 3-O-glucosyltransferase, putative, expressed
Os.57422.1.S1_x_at	LOC_Os03g49770	8	2.5E-06	4.6E-06	3.5E-05	6.4E-05	7.3E-04	1.2E-02	1427.6	1349.9	1455.4	764	621	624	607.8	671.9	587.4	525.1	552.2	505.5	No	No	No	oxysterol-binding protein 1, putative, expressed
Os.6670.1.S1_at	LOC_Os03g49770	11	1.6E-07	4.6E-07	2.4E-06	9.4E-06	1.6E-04	2.2E-03	2579.9	2855	2664	1389.9	1327.9	1399.1	1192.2	1294.5	1213.2	989.3	1052.3	1140.8	No	No	No	oxysterol-binding protein 1, putative, expressed
Os.8549.1.S1_at	LOC_Os03g50130	4	5.4E-05	1.2E-08	6.5E-04	6.4E-04	1.6E-05	8.2E-02	18764.6	21838.6	19994.4	9753.6	11768.2	16446.9	18592.9	20423.7	19943.3	13924.4	13036.4	15502.9	No	No	No	microsomal glutathione S-transferase 3, putative, expressed
OsAffx.25660.1.S1_at	LOC_Os03g50590	8	4.8E-07	2.4E-07	5.8E-07	2.0E-05	1.1E-04	9.1E-04	377.6	278	379.2	61.2	29.1	49.2	67.1	82.2	73.8	39.6	48.2	54.9	No	No	No	expressed protein
OsAffx.13452.1.S1_at	LOC_Os03g51040	4	9.7E-06	9.8E-07	2.1E-05	1.7E-04	2.6E-04	9.0E-03	7.2	2.3	29.6	104.5	96.4	51.9	3.8	30.2	3.3	166.3	112	189.8	No	No	Yes	ATP binding protein, putative
Os.10468.1.S1_at	LOC_Os03g51470	9	2.0E-08	1.8E-11	6.3E-08	2.4E-06	5.8E-07	2.2E-04	3560.3	3685	3769.7	437.1	429.2	415.2	2155.7	1710	1976.3	305.3	378.4	234.1	Yes	No	No	expressed protein
Os.31771.2.S1_x_at	LOC_Os03g51479	8	2.3E-08	4.7E-11	1.6E-07	2.5E-06	8.5E-07	3.9E-04	4418.6	3818.4	5721.8	591.8	766.8	768.4	2324.1	2129.5	1984.7	413.4	418.3	588.1	Yes	No	No	maf-like protein, expressed
Os.11802.1.S1_at	LOC_Os03g51650	4	3.8E-10	1.9E-05	1.7E-05	2.1E-07	1.9E-03	7.9E-03	4424	4019.5	4526.8	2771.3	2672.2	3024.8	5975.4	5607.3	6224.4	5625.3	5793.4	6172.3	No	No	Yes	expressed protein
Os.16532.1.S1_at	LOC_Os03g52370	4	1.1E-05	7.8E-07	3.8E-03	1.9E-04	2.2E-04	2.3E-01	4665.1	4759	5063	1713.3	1954.9	1705.8	2390.6	3374.1	2316.1	1132	888.3	833.2	No	No	No	expressed protein
Os.6620.1.S1_at	LOC_Os03g52390	7	2.5E-08	1.2E-06	1.3E-05	2.7E-06	3.0E-04	6.7E-03	1587.6	1396.4	1910.3	882.8	683	972.2	625.1	678.2	492.3	592.8	423.8	389.1	No	No	No	type II proteinase inhibitor family protein, expressed
Os.12747.1.S1_at	LOC_Os03g53230	4	9.9E-05	1.4E-05	4.6E-04	1.0E-03	1.6E-03	6.6E-02	18477.1	22168.3	19504.1	13682.9	13827.8	14762.1	16231.8	18028.7	17284.1	16581.4	16313.2	15170	No	No	No	bifunctional 3-phosphoadenosine 5-phosphosulfate synthetase, putative, expressed

Probe Set ID	Locus ID	probe #	ANOVA p-value			FDR q-value			Nipponbare MV			Nipponbare CK			93-11 MV			93-11 CK			Indel (Indica vs Japonica)			Annotation
			C	T	C×T	C	T	C×T	Repeat 1	Repeat 2	Repeat 3	Repeat 1	Repeat 2	Repeat 3	Repeat 1	Repeat 2	Repeat 3	Repeat 1	Repeat 2	Repeat 3	upstream	gene	downstream	
Os.46743.3.S1_a_at	LOC_Os03g53660	5	2.1E-04	2.1E-05	5.8E-04	1.8E-03	2.1E-03	7.7E-02	300.1	269.2	220	367.3	427.1	371.7	281.7	286.6	352.5	1023.6	722.4	822.2	No	No	Yes	XIF, putative, expressed
Os.54157.1.S1_at	LOC_Os03g55050	11	1.4E-09	4.9E-11	1.5E-09	4.5E-07	8.5E-07	2.0E-05	2066.2	2478.5	2078.8	91.8	64.4	69	558.4	678.5	552.5	43.9	38.1	9.4	No	No	No	cytokinin-O-glucosyltransferase 2, putative, expressed
Os.11216.1.S1_at	LOC_Os03g55090	6	1.8E-05	1.7E-04	4.8E-04	2.8E-04	8.7E-03	6.8E-02	524.1	466	386.6	470.4	453.3	309.8	706.2	754.5	838.7	543.7	482.2	467.5	No	No	No	alpha-1,4 glucan phosphorylase, L isozyme, chloroplast precursor, putative, expressed
Os.2898.1.S1_at	LOC_Os03g55164	4	1.2E-05	5.3E-07	7.2E-06	2.1E-04	1.7E-04	4.5E-03	9.3	9.6	8.4	11.5	8.1	2.7	2.8	4.3	9.6	94	14.8	128.2	No	Yes	No	OsWRKY4 - Superfamily of rice TFs having WRKY and zinc finger domains, expressed
OsAffx.13491.1.S1_at	LOC_Os03g55180	4	1.4E-04	4.2E-05	1.3E-04	1.4E-03	3.3E-03	2.9E-02	352.3	371.9	339.7	414.7	316.1	372.4	298.2	347.6	349.9	598.8	610.4	708.8	Yes	No	No	expressed protein
OsAffx.25717.1.S1_at	LOC_Os03g55210	8	1.2E-06	3.2E-07	1.0E-06	3.7E-05	1.3E-04	1.3E-03	62.1	43.2	48.1	122.9	77.5	67.5	55.7	32.7	41.9	258.7	280.5	201.7	No	Yes	No	protein kinase, putative, expressed
Os.11193.1.S1_at	LOC_Os03g55240	11	1.4E-07	1.5E-09	8.9E-08	8.6E-06	4.5E-06	2.8E-04	10559.1	11622.2	11637.2	519.2	556.1	630.7	3789.7	3759.4	4245.3	1104.8	1138.7	743	No	Yes	Yes	cytochrome P450 81E1, putative, expressed
Os.17985.1.S1_s_at	LOC_Os03g56500	6	1.8E-07	7.7E-08	6.5E-05	1.0E-05	4.9E-05	1.9E-02	794.9	852.7	863.1	259.4	304.8	292.3	185.5	306.2	226.2	65.5	69.8	74.8	No	No	No	expressed protein
Os.5581.1.S1_at	LOC_Os03g56540	9	6.4E-07	2.6E-10	8.2E-07	2.4E-05	1.8E-06	1.1E-03	5998.3	4742.9	5610.7	1007.4	713.9	1048.7	3038	3043.9	2658.2	689.7	698.8	815.5	No	No	No	mitochondrial import inner membrane translocase subunit TIM14, putative, expressed
Os.23297.1.S1_at	LOC_Os03g56860	3	1.1E-03	3.7E-06	5.0E-04	6.5E-03	6.4E-04	6.9E-02	1212.7	1025.9	1243.8	403.2	339.1	438.8	640.7	653.7	575.8	458.5	308.5	445.7	No	No	No	expressed protein
Os.2820.1.A1_at	LOC_Os03g56900	4	2.4E-06	4.2E-07	3.1E-04	6.2E-05	1.5E-04	5.1E-02	1276.6	1167.2	1277.5	1998.2	1522.3	1872.6	1714.7	1536.4	1526.2	3067.9	2456.2	2618.7	No	No	No	seed maturation protein, putative, expressed
Os.12200.1.S1_s_at	LOC_Os03g57200	7	4.1E-07	4.2E-05	1.4E-05	1.8E-05	3.3E-03	7.2E-03	6975.2	7675.7	8603	3424.1	2242.4	3943.1	1582.5	1823.1	2132.1	2524.4	2042.6	1925.5	Yes	No	No	glutathione S-transferase parA, putative, expressed
Os.46765.1.S1_x_at	LOC_Os03g57580	3	7.5E-08	1.6E-04	2.5E-03	5.5E-06	8.5E-03	1.8E-01	807	581.2	604.1	520.5	390.7	411.7	229.1	232.7	308.9	241.7	193.5	220.4	No	No	No	expressed protein
Os.54936.1.S1_at	LOC_Os03g57640	9	1.0E-06	2.9E-09	2.4E-06	3.3E-05	6.8E-06	2.2E-03	2315	2258.6	2142.9	243.6	276.4	246.1	1086.4	1063.9	927.8	136	225.7	188.5	No	No	No	gibberellin receptor GID1L2, putative, expressed
Os.26768.1.S1_at	LOC_Os03g58010	8	4.2E-06	9.2E-08	1.3E-05	9.4E-05	5.7E-05	6.9E-03	2996.1	3448.6	3063.8	589.8	577.5	434.4	1165.6	1858.6	1299.5	416.3	437.8	385.5	No	No	No	N-acetyltransferase, putative, expressed
Os.10884.1.S1_at	LOC_Os03g58790	3	4.6E-08	2.2E-08	7.1E-04	4.0E-06	2.3E-05	8.7E-02	5175.8	6048.8	6371.3	2360.7	2893	2712.5	3447.9	4802.2	4120.8	1342.1	1587.1	1613.3	No	Yes	Yes	ATPase 3, putative, expressed
OsAffx.25764.1.S1_at	LOC_Os03g59110	3	1.0E-05	7.7E-07	1.4E-05	1.8E-04	2.2E-04	7.2E-03	65.7	33.1	32	156.2	97	89.3	72.9	34.6	23.7	255.6	315.6	209.6	No	No	No	pheophorbide a oxygenase, chloroplast precursor, putative, expressed
Os.10146.1.S1_at	LOC_Os03g59320	4	7.1E-05	1.8E-06	6.3E-05	7.9E-04	4.0E-04	1.8E-02	4163.2	4061	3829.5	1982.9	2127	1931.6	4163.4	3914.1	4797.9	3371.6	3776.6	3448.4	No	No	No	expressed protein
Os.52136.1.S1_at	LOC_Os03g59430	3	4.4E-07	2.6E-05	9.3E-04	1.9E-05	2.4E-03	1.0E-01	140.3	175.5	118.9	291.7	194.6	291.9	218.6	295.4	202.9	444	314.7	470	Yes	No	No	stachyose synthase precursor, putative, expressed
OsAffx.25777.1.S1_at	LOC_Os03g59880	3	3.8E-07	5.6E-04	1.3E-03	1.7E-05	2.0E-02	1.2E-01	282	325.9	267.1	160.2	197.6	169.1	8.2	10.9	2.2	42.4	30.2	1.3	No	No	No	expressed protein
Os.10845.1.S1_s_at	LOC_Os03g60370	10	9.1E-08	1.1E-05	9.0E-06	6.3E-06	1.3E-03	5.2E-03	2648	2688.5	2984.2	1592.8	1365.1	1599.4	1371.3	1282.7	1416.5	1400.8	1276.3	1222.9	Yes	No	No	acid phosphatase, putative, expressed
Os.15874.1.A1_at	LOC_Os03g60570	5	8.6E-04	5.1E-09	3.1E-04	5.4E-03	9.6E-06	5.1E-02	740.7	618	952.6	69.6	119.6	147	304.8	465.7	469.6	194.2	47.3	85.8	No	No	No	zinc finger DNA-binding protein, putative, expressed
Os.39973.1.S1_s_at	LOC_Os03g60580	4	4.2E-05	2.8E-06	1.5E-05	5.3E-04	5.4E-04	7.3E-03	3304.5	3715.3	3748	1104.4	831.2	876.4	1635.2	2038.9	1763.2	1123.2	1711.6	1495.2	No	No	No	actin-depolymerizing factor 4, putative, expressed
Os.30886.1.S1_at	LOC_Os03g61360	5	1.9E-08	4.3E-04	1.4E-04	2.3E-06	1.7E-02	3.1E-02	3190.3	3733.1	3587.3	2327.1	2111.4	2248.9	1072.5	2658.9	2248.2	1687.9	2051.7	2511.9	Yes	Yes	Yes	epoxide hydrolase 2, putative, expressed
OsAffx.22650.2.S1_at	LOC_Os03g61470	4	4.1E-04	2.3E-04	3.9E-04	3.0E-03	1.1E-02	5.9E-02	130.9	97.2	100.8	477.2	253.3	338.4	76.4	55.1	72.5	279.9	182.7	188.2	No	No	No	PGPS/D12, putative, expressed
Os.34488.1.S1_at	LOC_Os03g61590	8	7.4E-07	3.0E-08	1.7E-06	2.6E-05	2.8E-05	1.7E-03	143.1	121.5	198.1	808.5	598.4	675.2	195.5	161.9	183.2	2313.1	2166.9	1979.6	No	No	No	expressed protein
Os.10435.1.S1_at	LOC_Os03g62060	5	8.7E-04	3.3E-06	4.5E-04	5.5E-03	5.9E-04	6.5E-02	957.4	1010.2	1063.8	3175.8	1839.5	2034	648.5	800.8	705	4764.8	5799.8	4350.8	Yes	No	No	IAA-amino acid hydrolase ILR1 precursor, putative, expressed
Os.52796.2.S1_x_at	LOC_Os03g62200	3	2.3E-04	1.4E-06	2.9E-04	1.9E-03	3.2E-04	4.9E-02	451.9	761.8	596.6	737.8	1000.6	737.7	329.3	459.6	446.1	748.6	942.9	792.5	Yes	Yes	Yes	ammonium transporter 2, putative, expressed
Os.49388.1.S1_at	LOC_Os03g62430	8	4.4E-09	6.4E-07	8.0E-07	8.9E-07	2.0E-04	1.1E-03	318	393.6	332.4	120.9	149.3	150.8	77	63.8	53.5	33	39.3	12.4	Yes	No	Yes	OsWAK28 - OsWAK receptor-like protein kinase, expressed
Os.23021.1.S1_at	LOC_Os03g62480	11	4.7E-12	4.2E-11	4.8E-11	2.2E-08	8.5E-07	2.7E-06	3549.4	3883.2	3694.2	450.4	408.4	373.8	109.7	286.1	140.2	69.7	67.6	48.4	No	No	Yes	anthocyanidin 5,3-O-glucosyltransferase, putative, expressed
Os.10849.1.S1_at	LOC_Os03g62490	5	1.2E-09	2.5E-07	2.5E-07	4.2E-07	1.1E-04	5.4E-04	1094.3	1101.8	1116.9	360.9	409.7	481.2	399.8	419.4	374.5	152.4	149	161.9	Yes	No	No	mitochondrial prohibitin complex protein 2, putative, expressed
OsAffx.10400.1.S1_x_at	LOC_Os03g62974	6	6.7E-07	1.1E-08	1.6E-06	2.5E-05	1.5E-05	1.7E-03	587.9	474.8	469	188	192.5	109.8	320.9	344.6	335.5	153	132.9	164.4	Yes	Yes	No	hypothetical protein
Os.56016.1.S1_at	LOC_Os03g63620	11	1.0E-07	8.9E-07	2.8E-06	6.8E-06	2.4E-04	2.4E-03	1970.5	1496.6	1741	285.9	310.2	409.4	112.6	104.5	67.4	18.2	43.8	32.2	Yes	No	No	senescence-associated protein DH, putative, expressed
Os.56144.1.S1_at	LOC_Os03g64260	4	4.0E-07	1.4E-07	4.5E-07	1.7E-05	7.2E-05	7.7E-04	286	230.2	179.2	38.9	21.7	27.3	130.1	106.3	113.1	13.3	10.3	10	No	No	No	ethylene responsive element binding protein, putative, expressed
Os.53673.1.S1_at	LOC_Os04g01310	7	4.2E-06	8.6E-06	4.9E-06	9.4E-05	1.1E-03	3.4E-03	449.3	426.8	498.3	116.5	174.7	132.5	62.1	67.4	55.3	68.4	71.2	61.6	Yes	No	No	serine/threonine-protein kinase receptor precursor, putative, expressed
Os.52423.1.S1_at	LOC_Os04g01674	3	1.7E-09	3.5E-03	5.2E-03	5.0E-07	6.9E-02	2.7E-01	1340.8	1489.3	1529.4	1667.8	1759.7	1781.8	307.7	334.4	350.3	204.6	246.7	265.5	Yes	No	No	glyoxylate reductase, putative, expressed
Os.18856.1.S1_at	LOC_Os04g02050	7	1.7E-05	5.1E-06	7.4E-06	2.7E-04	7.9E-04	4.6E-03	3819.6	4298.3	2921.9	1573.1	1384.1	1150.1	1551.8	1930.7	1343.7	1356.1	1599.9	1238.6	Yes	Yes	Yes	bifunctional 3-phosphoadenosine 5-phosphosulfate synthetase 2, putative, expressed
Os.27102.1.S1_x_at	LOC_Os04g02530	3	9.0E-10	3.8E-05	3.0E-05	3.5E-07	3.1E-03	1.1E-02	38.1	28.1	27.8	28	56	45.5	401.4	450.8	367.8	218.1	312.3	288	Yes	No	No	expressed protein
Os.54925.1.S1_at	LOC_Os04g02550	3	2.0E-07	1.8E-05	1.4E-04	1.1E-05	1.9E-03	3.1E-02	160.8	148.4	149.6	178.2	196.1	198.4	178.1	197.3	168.7	205.1	289.9	277.7	Yes	No	No	expressed protein
Os.37386.1.S1_at	LOC_Os04g03164	11	2.6E-09	8.4E-07	4.5E-07	6.6E-07	2.4E-04	7.6E-04	2002.7	2090.2	2087.3	745.7	657.3	967.8	54.7	29.5	59	44.3	104.9	116.8	Yes	Yes	Yes	expressed protein
OsAffx.25862.2.S1_at	LOC_Os04g03370	3	5.0E-05	4.1E-04	1.2E-03	6.0E-04	1.6E-02	1.2E-01	104.4	66.7	69	144.1	98.8	176.5	33.5	35.6	32.4	26.1	44.7	46.4	Yes	Yes	Yes	protein kinase, putative
Os.10027.1.S1_at	LOC_Os04g03579	10	7.8E-09	1.5E-05	1.4E-05	1.3E-06	1.6E-03	6.9E-03	1152.6	964.9	1458.3	2482.3	2605.8	3293	79.3	45.8	25.3	55.7	76.6	61.5	Yes	Yes	Yes	ATP binding protein, putative, expressed
Os.51175.1.S1_at	LOC_Os04g03884	4	1.8E-07	3.3E-04	3.5E-04	1.0E-05	1.4E-02	5.5E-02	84.8	129.3	85.1	152.9	192.9	97.9	8.8	3	1.8	1.1	0.8	1.2	Yes	Yes	Yes	transposon protein, putative, CACTA, En/Spm sub-class, expressed
Os.26930.1.S1_x_at	LOC_Os04g06590	4	3.4E-07	3.4E-06	7.7E-07	1.5E-05	6.0E-04	1.1E-03	1196.1	475.6	370.5	130.9	151.8	137.3	186.9	63.6	110.5	84	112.2	187.4	Yes	Yes	Yes	expressed protein
OsAffx.13699.1.S1_at	LOC_Os04g06900	3	5.2E-05	1.1E-04	1.3E-04	6.2E-04	6.5E-03	3.0E-02	13.1	7.6	24.8	6.3	26.2	42.8	86.8	72.8	45.1	141.6	149	126.9	No	Yes	Yes	expressed protein
Os.353.1.S1_at	LOC_Os04g08350	11	4.4E-07	4.6E-08	1.6E-07	1.9E-05	3.5E-05	4.0E-04	11696.3	12521.9	11710.4	3774	3229	3543	5522.3	6083.5	5916.9	4573.1	5217.2	5168.8	No	No	No	cysteine synthase, chloroplast precursor, putative, expressed
Os.55600.1.S1_at	LOC_Os04g08550	9	7.6E-09	1.3E-06	3.7E-05	1.3E-06	3.1E-04	1.3E-02	1902	2313.8	1900.3	827	908.8	890.7	113.3	211.9	164.7	129.6	95.5	78.7	No	Yes	Yes	NAD(P)H-dependent oxidoreductase, putative, expressed
Os.16233.1.S1_a_at	LOC_Os04g09900	3	9.6E-07	4.5E-04	3.3E-03	3.2E-05	1.7E-02	2.1E-01	896.2	1088.7	726.7	619.9	607.1	355.4	106	224.3	163	133.6	59.3	70	Yes	Yes	Yes	ent-kaurene synthase A, chloroplast precursor, putative, expressed
Os.7434.1.S1_at	LOC_Os04g10010	7	2.7E-08	1.2E-05	2.1E-05	2.8E-06	1.4E-03	9.1E-03	757.7	1058.7	979.6	771.9	489.4	334.1	141.7	160.2	110.1	113.1	89.4	110.7	Yes	Yes	Yes	sex determination protein tasselseed-2, putative, expressed
Os.24822.1.A1_at	LOC_Os04g10060	4	8.8E-07	7.7E-06	1.5E-05	3.0E-05	1.0E-03	7.4E-03	177.9	311.7	146.8	108.2	200.6	59.5	16.2	40.7	12.6	10.1	20.4	4.4	Yes	No	Yes	ent-kaurene synthase B, chloroplast precursor, putative, expressed
Os.53788.1.S1_at	LOC_Os04g10434	6	3.5E-07	1.0E-04	1.8E-04	1.6E-05	6.1E-03	3.7E-02	114.2	85.8	67.2	95.6	86	91.7	235.7	217.8	198.8	645	451.2	496	No	No	Yes	glutamyl-tRNA, putative, expressed
Os.5271.1.S1_at	LOC_Os04g10530	8	4.8E-07	5.1E-06	6.7E-06	2.0E-05	7.9E-04	4.3E-03	241.4	254.7	186.1	54.2	40.2	73.1	1.1	22.5	1.1	14.8	5.2	3	Yes	Yes	No	amidase, putative, expressed
Os.50428.1.S1_at	LOC_Os04g11820	3	2.4E-04	8.4E-06	6.6E-04	2.0E-03	1.1E-03	8.3E-02	496.8	468.6	562.4	219.5	254.8	206.6	244.9	248.8	310.1	181.6	162.9	148.1	No	No	No	ABC transporter-like protein, putative, expressed
Os.39636.1.A1_x_at	LOC_Os04g12720	8	9.3E-08	9.6E-08	9.5E-08	6.4E-06	5.8E-05	2.9E-04	437.4	483.7	332.1	23.9	3	50	32	50.3	5.2	24.7	4.3	2.8	No	Yes	Yes	indole-3-acetate beta-glucosyltransferase, putative, expressed
Os.54954.1.S1_x_at	LOC_Os04g12744	3	2.6E-08	4.3E-04	4.7E-04	2.8E-06	1.7E-02	6.6E-02	260.3	263.4	189.7	199.4	210.9	106.6	28	37.7	7.4	7.3	4.2	80	No	Yes	No	expressed protein
Os.28875.2.A1_x_at	LOC_Os04g12950	3	1.7E-06	9.4E-08	1.4E-06	4.7E-05	5.7E-05	1.6E-03	303.2	244	207.6	63.6	67.5	69.1	120.1	154.2	181	68	94.3	94.2	Yes	Yes	No	indole-3-acetate beta-glucosyltransferase, putative
Os.16198.1.S1_at	LOC_Os04g12960	10	6.0E-07	5.2E-07	6.1E-07	2.3E-05	1.7E-04	9.3E-04	16.7	66.1	2.7	6.4	32.6	3.2	493.8	621.5	396.7	3.9	27.5	6.4	No	No	Yes	indole-3-acetate beta-glucosyltransferase, putative, expressed
Os.6101.1.S1_at	LOC_Os04g12970	10	9.0E-08	9.4E-08	9.1E-08	6.2E-06	5.7E-05	2.8E-04	1382.3	1923.9	1616.1	1.2	20	0.8	112.7	134.1	86.1	1.1	18.6	36.5	No	No	No	indole-3-acetate beta-glucosyltransferase, putative, expressed
Os.14583.1.S1_at	LOC_Os04g12980	11	1.0E-09	3.0E-07	1.2E-06	3.7E-07	1.2E-04	1.4E-03	2938	3338.8	3687.8	1329.2	1946.3	1382.2	175.4	185.6	159	119	145.5	108.1	No	Yes	No	indole-3-acetate beta-glucosyltransferase, putative, expressed
Os.11286.1.A1_at	LOC_Os04g13140	7	3.6E-07	2.1E-05	7.2E-05	1.6E-05	2.1E-03	2.0E-02	831.6	927.3	678.6	202.3	432.4	308.5	122.2	145.9	218.8	132.6	156.8	107	No	No	No	vignain precursor, putative, expressed
Os.53852.1.S1_at	LOC_Os04g13210	3	1.9E-03	4.1E-08	7.6E-04	1.0E-02	3.3E-05	9.1E-02	978.9	1194.1	935.9	204.6	226	194.9	664.2	911.9	729.8	247.1	285.6	230.3	Yes	No	No	multidrug resistance-associated protein 4, putative, expressed
Os.8531.1.S1_at	LOC_Os04g15690	10	1.8E-09	3.1E-08	3.9E-07	5.2E-07	2.8E-05	7.0E-04	11734.8	14000	13944	5416.8	6280.8	7798.3	5115.3	5251.2	5196.1	3607.2	4458.6	3979	Yes	No	No	DSBA-like thioredoxin domain containing protein, expressed
Os.6089.1.S1_at	LOC_Os04g15920	11	7.4E-07	1.2E-05	3.3E-05	2.6E-05	1.4E-03	1.2E-02	2220.2	2222	2380.2	1220.8	828.4	1275.4	786	640.9	753.1	601.9	698.4	698.9	No	No	No	mannitol dehydrogenase, putative, expressed
Os.40398.1.S1_s_at	LOC_Os04g21130	6	7.7E-08	1.7E-06	3.4E-06	5.6E-06	3.8E-04	2.7E-03	30	47	21.2	46.8	54.7	46.7	64.4	99.8	87.5	376.1	363.9	301.1	Yes	No	Yes	expressed protein
Os.27688.1.A1_at	LOC_Os04g21350	4	2.1E-09	2.9E-05	2.4E-05	5.7E-07	2.6E-03	9.8E-03	92.9	109.3	90.9	89.5	104.3	118.4	616.5	509.9	830	875.6	879.4	1354.8	No	No	No	flowering promoting factor-like 1, putative, expressed
Os.11837.1.S1_at	LOC_Os04g26920	4	1.5E-07	3.0E-04	3.9E-04	9.0E-06	1.3E-02	5.9E-02	1022.4	923.2	715.7	670.9	552.1	495.7	27.1	40.6	19.8	42.2	34.4	15.2	No	No	No	IN2-2 protein, putative, expressed
OsAffx.26176.1.S1_at	LOC_Os04g26960	4	1.8E-03	1.3E-04	9.0E-04	9.3E-03	7.1E-03	1.0E-01	6.1	71.6	47.4	307.3	178.9	314.1	17.4	43.9	19.6	40.1	73	50.9	No	No	No	terpene synthase 7, putative, expressed
Os.11266.1.S1_at	LOC_Os04g27060	8	2.0E-05	1.7E-10	1.6E-05	3.0E-04	1.6E-06	7.7E-03	14827.6	15929.2	13037.9	105.5	326.3	177.1	8258.2	11102.9	8980.7	371.9	351.9	187.1	No	No	No	auxin-induced protein PCNT115, putative, expressed
Os.4819.1.A1_s_at	LOC_Os04g27980	7	4.2E-05	1.6E-04	3.7E-04	5.3E-04	8.3E-03	5.7E-02	98.2	195.6	184.2	234.1	206.1	211.3	274.3	297.2	143.3	1033.6	867.2	810.1	No	No	No	acidic endochitinase precursor, putative, expressed
Os.25531.1.S1_at	LOC_Os04g28990	4	6.0E-05	1.5E-06	4.3E-03	7.0E-04	3.5E-04	2.5E-01	1503.2	1371.4	1575.9	3353.3	2008.9	2857.6	2078.7	1924.4	2151.8	5049.4	3987.1	5187.9	No	No	No	chloroplastic quinone-oxidoreductase, putative, expressed
OsAffx.26254.1.S1_at	LOC_Os04g30490	11	1.2E-08	9.5E-10	2.3E-08	1.7E-06	3.5E-06	1.2E-04	2256.6	2050.7	2164.8	275.5	234.8	222.3	496.2	583.1	533.4	174.7	174.9	162.5	Yes	No	No	transparent testa 12 protein, putative, expressed
Os.9523.1.S1_at	LOC_Os04g32620	3	2.3E-06	3.4E-05	6.7E-04	6.0E-05	2.9E-03	8.4E-02	403.3	425.1	471.7	187.7	198	142.1	135.6	277.8	158.4	166.9	174.2	111.1	Yes	No	No	DNA binding protein, putative, expressed
Os.24051.2.S1_at	LOC_Os04g33640	4	7.8E-05	1.0E-04	6.6E-05	8.5E-04	6.2E-03	1.9E-02	129.5	94.5	100.5	52.5	87.4	24.3	57.2	25.8	38.9	64.9	85.2	15.2	Yes	Yes	No	glucan endo-1,3-beta-glucosidase 7 precursor, putative, expressed
OsAffx.26320.1.S1_at	LOC_Os04g34240	4	4.8E-09	9.2E-05	1.2E-04	9.4E-07	5.7E-03	2.7E-02	1206.5	807.7	1542.1	1863.2	1333.6	1787.7	70.5	53.3	90.9	91.4	58.2	51.8	Yes	Yes	Yes	histone H3, putative, expressed
OsAffx.26320.1.S1_x_at	LOC_Os04g34240	4	1.7E-08	6.2E-04	7.4E-04	2.1E-06	2.1E-02	8.9E-02	1491.4	1014.1	1494.4	2352.7	2219.5	2081.2	112.3	143.9	94.5	187.5	143	123	Yes	Yes	Yes	histone H3, putative, expressed
Os.15942.1.S1_s_at	LOC_Os04g34600	5	2.7E-12	7.0E-04	5.0E-04	1.6E-08	2.3E-02	7.0E-02	3472.8	3710.2	3302.9	2316.7	2458.5	2657.8	17.2	222.7	137.4	181.6	190.8	169.2	No	Yes	Yes	ABA/WDS induced protein, expressed
Os.15942.5.S1_x_at	LOC_Os04g34600	3	1.6E-09	1.2E-03	1.0E-03	4.8E-07	3.5E-02	1.1E-01	2257.4	2547.9	2504.1	1744.7	1992.8	1615.2	110.8	170	80.4	195.9	98.1	95	No	Yes	Yes	ABA/WDS induced protein, expressed
OsAffx.14163.1.S1_at	LOC_Os04g35660	5	1.3E-04	2.1E-05	1.7E-04	1.2E-03	2.1E-03	3.5E-02	83.8	71.8	90.1	162.4	102.6	89.3	81.1	95.4	60.6	254.6	186.8	217	No	No	No	NAC domain-containing protein 18, putative, expressed
OsAffx.26357.1.S1_at	LOC_Os04g36830	9	2.8E-05	1.0E-06	2.8E-05	3.9E-04	2.7E-04	1.1E-02	479.5	389.3	298.1	47.9	60.2	2.6	116.8	131.7	98.9	35.9	48.8	37.2	No	No	No	expressed protein
Os.11816.1.S1_at	LOC_Os04g37490	11	3.5E-08	4.6E-10	7.8E-07	3.3E-06	2.4E-06	1.1E-03	8379.8	8660.2	8170.2	1303.5	1409.9	1260.7	3650.4	3837.5	2843.2	612.5	687.5	589.5	No	No	No	NAD(P)H-dependent oxidoreductase, putative, expressed
Os.53639.1.S1_at	LOC_Os04g37650	3	1.8E-04	4.1E-06	3.1E-04	1.6E-03	6.7E-04	5.1E-02	43	60	36.7	309.2	380.8	337.4	42.3	54.5	2.5	207.1	130	136.7	No	Yes	No	fucosyltransferase 7, putative, expressed
Os.14998.1.S1_at	LOC_Os04g37670	5	2.9E-04	8.3E-05	5.8E-05	2.4E-03	5.3E-03	1.7E-02	1062.4	1283.6	1156	658.8	562.5	629.3	652.9	738.5	637.6	656	720.8	679	No	No	No	steroid nuclear receptor, ligand-binding, putative, expressed
Os.5846.1.S1_at	LOC_Os04g38530	3	2.2E-13	4.4E-04	8.3E-04	5.8E-09	1.7E-02	9.6E-02	1743.8	1823.6	1909	2041.6	2007.3	2081.9	27.7	36.4	34.1	58.9	80.7	32.9	No	No	No	aldose 1-epimerase precursor, putative, expressed
Os.6876.1.S1_at	LOC_Os04g38950	8	1.6E-09	6.6E-09	6.9E-07	4.9E-07	1.1E-05	1.0E-03	3461.8	3466.7	3103.9	1578.8	1525.1	1291.2	1416.5	1707.3	1280.9	892.6	972.5	858.4	No	No	Yes	anthranilate synthase component II, putative, expressed
Os.23802.1.A1_at	LOC_Os04g39540	10	1.2E-07	6.1E-07	2.2E-06	7.7E-06	1.9E-04	2.1E-03	3845	3714.9	4010.2	1076.8	1002.4	1466.7	961.8	1067.8	1025.2	601	604.8	752.5	Yes	No	Yes	expressed protein
Os.5963.1.S1_at	LOC_Os04g39600	4	1.9E-06	4.8E-08	2.8E-06	5.2E-05	3.6E-05	2.4E-03	97.3	133.4	105	637	317.6	387.6	81.9	100.5	70.9	612.2	462.9	510.8	Yes	No	No	fasciclin-like arabinogalactan protein 7 precursor, putative, expressed
Os.22910.1.S1_at	LOC_Os04g39880	5	4.7E-09	3.5E-08	8.9E-05	9.4E-07	3.1E-05	2.3E-02	2031.1	2065.2	2397.5	5230.3	2922.6	5094.5	6583	5482.1	5708.3	11910.2	11945.7	12600.6	No	No	No	non-cyanogenic beta-glucosidase precursor, putative, expressed
Os.22910.2.S1_at	LOC_Os04g39880	3	2.6E-08	1.4E-07	3.8E-07	2.8E-06	7.3E-05	6.9E-04	36.8	36.8	8.6	62.4	62.8	52	95.2	89.3	91.9	155.5	186.7	222.8	No	No	No	non-cyanogenic beta-glucosidase precursor, putative, expressed

Probe Set ID	Locus ID	probe #	ANOVA p-value			FDR q-value			Nipponbare MV			Nipponbare CK			93-11 MV			93-11 CK			Indel (Indica vs Japonica)			Annotation
			C	T	C×T	C	T	C×T	Repeat 1	Repeat 2	Repeat 3	Repeat 1	Repeat 2	Repeat 3	Repeat 1	Repeat 2	Repeat 3	Repeat 1	Repeat 2	Repeat 3	upstream	gene	downstream	
Os.7806.1.S1_at	LOC_Os04g39980	3	5.7E-06	9.2E-07	3.5E-04	1.2E-04	2.5E-04	5.6E-02	2861.9	2829	3464.8	1457.1	671.3	1242.7	890.6	1640.1	2018.2	773.5	922.2	915.4	Yes	No	No	gibberellin 20 oxidase 2, putative, expressed
Os.9293.1.S1_at	LOC_Os04g40040	4	2.9E-08	3.4E-07	2.6E-05	2.9E-06	1.3E-04	1.1E-02	5220.9	4429.9	5356.6	8102.2	6626.9	9084.3	3542	2811.8	3546.6	4629.3	4381.1	5202.3	Yes	Yes	Yes	copper methylamine oxidase precursor, putative, expressed
Os.23932.1.A1_at	LOC_Os04g40140	5	6.6E-07	2.5E-05	4.4E-04	2.4E-05	2.3E-03	6.4E-02	250.3	149.9	146.6	149.1	116.9	155.9	390.9	667.4	537.7	287.4	297.6	299.6	No	No	No	expressed protein
Os.16333.1.S1_at	LOC_Os04g40310	4	3.9E-04	1.4E-06	3.0E-04	3.0E-03	3.2E-04	5.0E-02	5527.8	6236.9	5131.4	2796.5	2753.9	2819.4	3900.4	4242.1	3876.5	3129.6	3241.2	3180.2	No	No	No	isocitrate dehydrogenase subunit 1, mitochondrial precursor, putative, expressed
Os.52574.1.S1_at	LOC_Os04g41450	10	4.1E-08	6.5E-09	2.9E-07	3.7E-06	1.1E-05	5.8E-04	168.4	131.4	163.9	744	315.1	527	323.4	349.2	385.2	1752.5	1983.9	1788.8	No	No	No	TGF-beta receptor, type I/II extracellular region, putative, expressed
Os.52574.2.S1_x_at	LOC_Os04g41450	9	7.3E-06	1.6E-06	3.1E-05	1.4E-04	3.6E-04	1.2E-02	79.3	107.8	101.5	339.5	116.9	216	152.3	113.2	175	825.1	643.7	781.2	No	No	No	TGF-beta receptor, type I/II extracellular region, putative, expressed
Os.53077.1.S1_at	LOC_Os04g42760	4	4.2E-05	2.9E-05	1.5E-04	5.3E-04	2.6E-03	3.3E-02	61.5	128.7	85.9	184.2	96.1	94.5	63.8	99.1	100.2	431.2	374.4	246.2	No	No	No	sialyltransferase-like protein, putative, expressed
Os.52536.1.S1_at	LOC_Os04g43800	10	4.3E-07	8.5E-08	2.3E-06	1.8E-05	5.3E-05	2.1E-03	4587	7234.9	6395.2	995.9	1823.7	1529.1	2013.9	2492	1522.2	780	799.2	753	No	No	No	phenylalanine ammonia-lyase, putative, expressed
Os.10860.2.S1_at	LOC_Os04g44320	4	2.9E-03	1.1E-06	9.8E-05	1.4E-02	2.8E-04	2.5E-02	1912.2	2019.9	2093.7	2433.8	2194.9	2908.1	1717.8	1959.7	1941	3720.6	3679.4	3706.4	No	No	No	transposon protein, putative, unclassified, expressed
Os.12986.1.S1_at	LOC_Os04g44500	3	1.7E-03	5.2E-03	1.9E-05	9.0E-03	9.0E-02	8.6E-03	2283.4	2359.7	2224.8	2032.9	1954	1874	1763	1859.3	1802.7	3484.4	3011.7	3630.9	Yes	No	No	FIP1, putative, expressed
Os.19361.1.S2_at	LOC_Os04g45290	3	6.9E-04	6.1E-05	2.9E-03	4.6E-03	4.3E-03	2.0E-01	2145.8	2602.8	2967.7	2943	2907.5	3005.9	3032.6	3298	2388.1	5201.5	5139.9	4786	No	No	Yes	beta-fructofuranosidase 1 precursor, putative, expressed
Os.30193.1.S1_at	LOC_Os04g46250	4	3.4E-07	3.8E-07	8.5E-07	1.5E-05	1.4E-04	1.1E-03	30.3	6.9	30.1	69.5	40.8	11.2	40.5	35.4	30.2	163.8	126.7	144.5	No	No	No	AP2 domain containing protein, expressed
Os.47380.1.S1_at	LOC_Os04g46810	3	6.7E-04	3.6E-10	1.2E-03	4.4E-03	2.2E-06	1.2E-01	246.2	193	745.9	6908.6	5485.9	8799.5	495	693.2	501.1	11181.2	7554	6835.3	No	No	No	cortical cell-delineating protein precursor, putative, expressed
OsAffx.26492.1.S1_at	LOC_Os04g46830	3	3.6E-03	7.0E-03	6.3E-03	1.6E-02	1.1E-01	3.0E-01	3346.6	2159.9	3745.1	3075.5	2437.6	3187.4	9373.6	5245.9	6544.7	3345.4	2899.8	3496.5	Yes	No	No	cortical cell-delineating protein precursor, putative, expressed
OsAffx.26492.1.S1_s_at	LOC_Os04g46830	5	3.1E-04	9.2E-04	8.5E-04	2.4E-03	2.8E-02	9.7E-02	6622.5	5271.9	8519.7	6475.4	6508.4	8192.9	16432.6	12511.6	14070.9	8187.5	7306	10460.7	Yes	No	No	cortical cell-delineating protein precursor, putative, expressed
Os.23440.1.A1_at	LOC_Os04g46910	8	2.7E-08	9.1E-10	3.6E-08	2.8E-06	3.5E-06	1.6E-04	4.3	8.6	43.5	192.2	159.7	139.3	41.4	76.8	69.7	587.5	425.4	455.1	No	No	No	actin-depolymerizing factor, putative, expressed
Os.49838.1.S1_at	LOC_Os04g47720	9	4.7E-09	3.4E-08	9.5E-08	9.3E-07	3.0E-05	2.9E-04	533.5	616.9	564.6	168.7	140.1	143.7	98	135	100.4	25.3	37.6	83	No	No	Yes	cis-zeatin O-glucosyltransferase, putative, expressed
Os.49507.1.S1_at	LOC_Os04g48850	4	9.5E-05	1.2E-06	1.2E-05	9.9E-04	2.9E-04	6.2E-03	535.8	482.2	485.9	143.9	137.5	153	286.8	295.8	330.8	262.7	217.2	200.4	No	No	No	1-aminocyclopropane-1-carboxylate synthase, putative, expressed
Os.7095.1.S1_at	LOC_Os04g49210	6	8.0E-08	1.0E-06	2.2E-05	5.8E-06	2.7E-04	9.3E-03	1500	1802	1755.5	1157.6	1278.7	1194.7	5362.8	4835.6	5104.6	2131.5	2754.7	2671.4	Yes	Yes	Yes	naringenin,2-oxoglutarate 3-dioxygenase, putative, expressed
Os.5335.1.S1_at	LOC_Os04g49450	4	4.7E-09	2.4E-07	2.9E-05	9.4E-07	1.1E-04	1.1E-02	595.3	665.7	589.9	452.8	517.2	445.1	266.7	272.6	248.9	165.1	287.1	247.1	No	No	No	expressed protein
Os.54481.2.S1_at	LOC_Os04g49460	6	3.2E-05	2.6E-06	5.0E-04	4.3E-04	5.1E-04	7.0E-02	438.3	504.5	539.8	164.6	207.7	194.7	213.5	243.8	284.4	127.4	124.9	203.9	No	No	No	ATP binding protein, putative, expressed
Os.27657.1.A1_at	LOC_Os04g49890	8	3.1E-06	2.1E-08	2.6E-05	7.4E-05	2.2E-05	1.1E-02	5106.9	5707.3	3894.7	917.4	1095.9	1084.8	2030.2	2734.1	2512.9	586.8	714.3	581.5	No	No	No	ABC transporter family protein, expressed
Os.5562.1.S1_at	LOC_Os04g51150	9	5.6E-06	1.8E-10	6.3E-06	1.1E-04	1.6E-06	4.1E-03	9871.4	10367.2	9541.8	1046.7	941.6	883.2	6519.6	7777.8	5996.5	1171.1	930.8	804.9	No	No	Yes	transposon protein, putative, unclassified, expressed
Os.3406.1.S1_at	LOC_Os04g51160	11	2.9E-07	2.2E-10	5.7E-07	1.4E-05	1.7E-06	9.0E-04	11153.7	11557.9	12694.4	925.5	810.5	1124.3	4194	4984.6	4349.8	839.4	438.8	573.2	No	No	No	transposon protein, putative, unclassified, expressed
OsAffx.13041.1.S1_x_at	LOC_Os04g51420	4	6.3E-10	1.8E-07	3.2E-07	2.8E-07	8.5E-05	6.2E-04	287.6	372.2	169.3	218.9	142.9	78.7	82.3	93.4	137.8	83.8	52.3	77.3	No	No	No	transposon protein, putative, unclassified
Os.9554.1.S1_at	LOC_Os04g52504	7	3.0E-05	9.6E-06	4.4E-05	4.1E-04	1.2E-03	1.4E-02	6247.5	6416.8	6712.9	3084.4	2517.6	3379.5	3416.5	3550	3501	3214.6	2921.9	3704.7	No	No	No	adhesive/proline-rich protein, putative, expressed
Os.55628.1.S1_at	LOC_Os04g53606	8	2.1E-06	3.7E-09	1.9E-06	5.5E-05	7.9E-06	1.9E-03	1129.3	1166.5	1316.1	25.8	35.4	52	433.5	689.6	496.5	39.4	64.2	53.7	No	No	Yes	expressed protein
OsAffx.26671.1.S1_x_at	LOC_Os04g53612	4	3.8E-04	2.6E-08	2.1E-04	2.9E-03	2.5E-05	4.0E-02	90.6	76.5	106	3.7	5.9	2.4	5.2	66.9	14.5	2.7	3.2	3.6	No	No	No	expressed protein
Os.15825.1.S1_at	LOC_Os04g53720	4	2.8E-07	3.2E-05	1.7E-05	1.3E-05	2.8E-03	7.9E-03	307.3	251	192.9	139.2	117.1	88	54	9.8	39	37.7	46	64.7	Yes	No	No	ATP binding protein, putative, expressed
Os.15825.1.S2_at	LOC_Os04g53720	6	4.1E-07	6.9E-05	8.6E-05	1.8E-05	4.7E-03	2.2E-02	755.7	615.8	531.8	261	388	408.8	54	44.7	52.6	24.9	56.1	32.1	Yes	No	No	ATP binding protein, putative, expressed
Os.11471.1.S1_at	LOC_Os04g54610	7	4.4E-03	1.0E-03	1.7E-03	1.9E-02	3.0E-02	1.5E-01	395.3	460.3	411.8	641.1	676.3	503.1	301.6	285.7	304.8	3857.6	2977.7	1896.7	No	No	No	hypothetical protein
Os.18254.1.A1_at	LOC_Os04g55159	3	2.0E-05	6.2E-09	3.2E-05	2.9E-04	1.0E-05	1.2E-02	3070.8	4485	3486.4	1151.7	1646.1	1302.4	2007.4	2878.3	1812.1	1005.8	1100.6	1064.6	No	No	No	expressed protein
Os.28110.1.S1_x_at	LOC_Os04g55710	10	5.9E-09	3.2E-07	8.2E-07	1.1E-06	1.3E-04	1.1E-03	5284.9	4616.2	4211.3	2044.2	1446.2	1507.8	689.7	965.8	756.1	571.7	514.2	683.4	Yes	No	No	transposon protein, putative, unclassified, expressed
Os.28110.3.S1_at	LOC_Os04g55710	3	1.0E-05	2.0E-05	2.1E-04	1.8E-04	2.0E-03	4.0E-02	54	70.1	27.5	71.1	48.7	23.3	71.5	32.1	27.2	3.7	70.6	7.8	Yes	No	No	transposon protein, putative, unclassified, expressed
Os.5551.1.S1_at	LOC_Os04g55720	5	5.7E-07	1.4E-08	3.9E-06	2.2E-05	1.7E-05	3.0E-03	9261.9	9883.4	8982.4	4369.9	4285	4067.6	6469.6	6837.8	6419.3	4596	3430.9	3441.2	No	No	No	D-3-phosphoglycerate dehydrogenase, chloroplast precursor, putative, expressed
Os.55343.1.S1_at	LOC_Os04g56030	6	1.4E-07	9.3E-09	1.1E-06	8.3E-06	1.3E-05	1.4E-03	925.5	802.9	937.9	157.6	152.5	222.9	364.2	412.1	264.3	68.6	108.1	140.1	Yes	Yes	Yes	expressed protein
OsAffx.26624.1.S1_at	LOC_Os04g56040	4	3.8E-07	8.9E-06	1.1E-05	1.7E-05	1.2E-03	6.2E-03	176.9	157.9	194.6	55.8	4.2	71.5	10.2	8.8	17.6	4.3	24.3	4.9	No	Yes	Yes	glycine rich protein family protein, expressed
Os.28305.1.S1_at	LOC_Os04g56110	6	1.2E-07	1.9E-09	2.3E-07	7.4E-06	5.4E-06	5.0E-04	1808.5	1539	1801.4	416.6	385.9	356.5	602.6	741.4	764.1	467.8	426.5	471.7	Yes	No	No	ATP binding protein, putative, expressed
Os.56111.1.S1_at	LOC_Os04g56120	4	1.0E-07	2.8E-06	1.4E-05	6.7E-06	5.3E-04	6.9E-03	278.8	235.7	193.6	121.7	107.8	93.2	38	79.5	35.8	25.6	24.8	53.5	No	No	No	receptor-like kinase, putative, expressed
Os.1606.1.S1_at	LOC_Os04g56430	4	6.6E-04	4.8E-06	2.9E-06	4.4E-03	7.5E-04	2.5E-03	3460.2	2716.5	2855.6	370.8	465.8	357.6	894.2	1059.9	525.5	862.2	847.8	997.4	No	No	Yes	CRK5, putative, expressed
Os.55838.1.S1_at	LOC_Os04g56560	7	2.0E-08	1.0E-08	4.2E-08	2.3E-06	1.4E-05	1.7E-04	456.6	499.8	374	117.8	61.1	55	92.5	131.7	120.4	59.9	44.7	33	No	No	No	peptide transporter PTR2, putative, expressed
Os.5514.1.S1_at	LOC_Os04g56740	3	4.8E-05	2.2E-05	1.5E-03	5.9E-04	2.1E-03	1.3E-01	5201	6193.6	5546.4	6730.7	7236.1	7616.6	8388	7405.8	7634	8227	8016.9	8708.2	No	No	No	IQ calmodulin-binding motif family protein, expressed
OsAffx.14448.1.S1_at	LOC_Os04g56930	3	3.9E-07	1.9E-05	1.0E-03	1.7E-05	2.0E-03	1.1E-01	1497.1	1602.2	1440.6	963.9	831.7	908.2	482.2	647	427.4	298.4	355.3	311.4	No	No	No	beta-fructofuranosidase, insoluble isoenzyme 5, putative, expressed
Os.50125.1.S1_at	LOC_Os04g57860	8	2.5E-07	2.9E-09	3.6E-07	1.2E-05	6.8E-06	6.6E-04	105.3	109.3	121.4	1290.4	836.9	1386.7	179.9	122	122.8	4012.5	3805.7	3573.1	No	No	No	endoglucanase precursor, putative, expressed
Os.49198.1.S1_at	LOC_Os04g58090	10	8.8E-07	7.0E-09	1.4E-06	3.0E-05	1.1E-05	1.5E-03	2059.4	1990.2	2246.1	194.9	274.5	202.7	823.5	953.8	915.2	237.3	108.5	214.8	No	No	No	NHL25, putative, expressed
Os.22374.1.S2_a_at	LOC_Os04g58710	3	5.8E-06	4.2E-04	4.9E-05	1.2E-04	1.6E-02	1.6E-02	14591.6	17494.7	16279.1	10418.2	11500.2	12073.6	17656	17958.8	18881.1	19855.2	20428	16465.5	No	No	No	peroxisomal-coenzyme A synthetase, putative, expressed
Os.15573.1.A1_s_at	LOC_Os04g58734	3	1.9E-06	1.8E-04	6.0E-03	5.1E-05	9.0E-03	2.9E-01	783	604.2	471.5	485.3	572.2	472.8	1542.2	1211.6	1543.8	844.8	747	1242.2	No	No	No	expressed protein
Os.32360.1.A1_at	LOC_Os04g58760	5	1.7E-05	9.3E-09	2.5E-05	2.7E-04	1.3E-05	1.0E-02	165.6	169.5	201.7	962.2	653.6	894.4	107.5	146.3	154	1502.8	1031	1054.7	No	No	Yes	plant integral membrane protein TIGR01569 containing protein, expressed
Os.52001.1.S2_at	LOC_Os05g01030	3	1.7E-04	2.9E-05	1.0E-03	1.6E-03	2.6E-03	1.1E-01	1196.8	1938.2	1494.3	580.4	723.9	678.9	666	1019.5	762.7	603.4	676.5	553	No	No	No	phospholipid-transporting ATPase 10, putative, expressed
Os.7381.1.S1_at	LOC_Os05g02770	6	1.3E-05	5.5E-06	3.2E-04	2.2E-04	8.3E-04	5.2E-02	925	953.3	846.7	494.8	543.6	454.2	305.9	362.4	501	327.3	295.5	297.5	No	Yes	No	glycine-rich protein A3, putative, expressed
OsAffx.14512.1.S1_at	LOC_Os05g02790	4	1.4E-04	7.9E-05	8.7E-05	1.4E-03	5.1E-03	2.3E-02	40	4.5	32	58.1	54.7	59.3	62.3	32	14.5	127.9	146.4	127.9	No	No	Yes	protein kinase interacting protein 1, putative, expressed
Os.12568.1.S1_at	LOC_Os05g03820	6	8.5E-06	1.9E-06	7.6E-05	1.6E-04	4.0E-04	2.1E-02	3973.4	4665.1	4296.4	1875.2	2122.7	2008.5	2479.8	2817.9	2545.2	1957.3	2229.1	1803	Yes	No	Yes	glutamate--cysteine ligase, chloroplast precursor, putative, expressed
Os.12568.1.S1_x_at	LOC_Os05g03820	4	4.6E-07	4.8E-09	1.5E-05	1.9E-05	9.2E-06	7.5E-03	5745.2	6849.1	6167.8	3704.5	3814	3779.2	4461.6	4818.1	4674.3	3339.5	4282	3702.7	Yes	No	Yes	glutamate--cysteine ligase, chloroplast precursor, putative, expressed
Os.50587.1.S1_at	LOC_Os05g03960	7	5.3E-07	2.6E-05	4.0E-05	2.1E-05	2.4E-03	1.4E-02	200.7	289.2	219.8	141.3	227.2	115.5	811	907.6	983.7	422.6	377.2	364	Yes	Yes	Yes	hypothetical protein
OsAffx.4250.1.S1_at	LOC_Os05g04440	3	2.4E-03	2.4E-03	4.2E-03	1.2E-02	5.4E-02	2.4E-01	153.9	63.5	111.8	2465.5	1012.5	1638.3	58.5	76.1	127.2	2793.7	1418.7	1442.5	Yes	No	No	peroxidase 24 precursor, putative, expressed
OsAffx.4250.1.S1_s_at	LOC_Os05g04440	7	1.5E-06	1.4E-10	3.3E-06	4.3E-05	1.5E-06	2.7E-03	120	221.4	164	1943.6	2798	2343.2	85.7	92.4	65.2	303.5	272.1	246	Yes	No	No	peroxidase 24 precursor, putative, expressed
Os.11555.1.S1_at	LOC_Os05g04450	4	5.9E-08	2.4E-09	1.8E-07	4.7E-06	6.2E-06	4.3E-04	210.8	248.6	261.9	2122.7	1625.8	2007.2	75.2	94.8	47.3	435	488	376.3	No	No	Yes	peroxidase 12 precursor, putative, expressed
Os.13968.2.S1_a_at	LOC_Os05g04700	3	4.7E-05	2.1E-05	1.2E-04	5.8E-04	2.1E-03	2.7E-02	508.5	485	486.8	212.1	235.6	230.5	229.2	218.5	280.7	167.8	244.9	257.2	No	No	No	hydrophobic protein LTI6B, putative, expressed
OsAffx.26739.1.S1_s_at	LOC_Os05g05080	9	7.6E-10	1.3E-10	1.5E-09	3.2E-07	1.4E-06	2.0E-05	1428.7	1935.1	1659.3	78.8	115.7	112.7	346	362.6	343.9	10.2	37.7	31.2	Yes	No	No	expressed protein
Os.50180.1.S1_at	LOC_Os05g05100	4	1.5E-06	6.8E-05	8.6E-05	4.4E-05	4.6E-03	2.2E-02	154	284.6	185.8	58	79.4	67.8	67.4	72.4	80.8	43.5	27.7	36	No	No	Yes	expressed protein
Os.51864.1.S1_at	LOC_Os05g05110	4	3.2E-06	1.8E-06	3.6E-05	7.7E-05	4.0E-04	1.3E-02	113.3	117.3	146.5	36.5	33.1	36.4	19.5	52.5	33.6	1.9	4.6	11.1	No	No	No	expressed protein
Os.12993.1.S1_at	LOC_Os05g07090	4	1.8E-06	6.2E-07	4.1E-05	5.0E-05	1.9E-04	1.4E-02	4067.7	4093.8	3892.2	2571.9	2444.9	2611.9	2204.6	3020.4	2690	2252.4	2089.2	2488.6	No	No	Yes	glutaryl-CoA dehydrogenase, mitochondrial precursor, putative, expressed
Os.56950.1.S1_at	LOC_Os05g07120	3	5.8E-09	7.2E-09	8.8E-09	1.1E-06	1.1E-05	6.3E-05	223.9	184.3	223.5	33.4	75.8	47.6	80.9	60	76.5	2.2	29.7	3.4	No	No	No	DNA binding protein, putative, expressed
OsAffx.26787.1.S1_s_at	LOC_Os05g08390	3	4.5E-09	3.1E-04	2.6E-03	9.1E-07	1.3E-02	1.9E-01	274.2	295.5	298.6	470.8	469	486.2	117.6	171.8	84.6	114.7	151.5	176.7	No	No	No	conserved hypothetical protein
Os.6152.1.S1_at	LOC_Os05g08900	8	2.1E-09	3.2E-06	3.0E-06	5.6E-07	5.8E-04	2.5E-03	1367.8	1411.6	1446.4	474.7	681.5	682.9	124.6	208	130.2	118.1	74.8	164.4	Yes	Yes	Yes	expressed protein
Os.51184.1.S1_at	LOC_Os05g10370	9	3.1E-09	2.9E-08	4.5E-08	7.3E-07	2.7E-05	1.8E-04	1784	1382	1508.3	1873.3	1700.9	1764.6	2818.8	3207.8	2582.8	8629.2	8904.8	8957.7	Yes	No	No	acid phosphatase, putative, expressed
OsAffx.14625.1.S1_at	LOC_Os05g10780	3	1.4E-07	1.4E-07	9.5E-07	8.4E-06	7.3E-05	1.2E-03	19.3	15.3	2.4	57.3	31	43	31.6	4.4	4.5	118.9	98.8	80.4	Yes	Yes	No	1-aminocyclopropane-1-carboxylate synthase 7, putative, expressed
OsAffx.26850.2.S1_at	LOC_Os05g12190	7	9.8E-10	9.4E-10	1.6E-09	3.7E-07	3.5E-06	2.0E-05	5.2	50.7	44.8	81.6	43.9	4.3	8.3	53.3	6.6	547.4	660.8	503.4	No	No	No	chalcone and stilbene synthases, N-terminal domain containing protein
OsAffx.26850.1.S1_x_at	LOC_Os05g12240	10	1.5E-11	1.2E-11	1.8E-11	3.6E-08	4.5E-07	1.5E-06	25.7	39.1	33.1	387.2	168.1	141.1	106	219.8	112.1	2230	1690.6	1763.8	No	No	No	chalcone synthase 8, putative
OsAffx.26856.1.S1_at	LOC_Os05g12450	9	2.4E-09	1.1E-07	1.7E-07	6.2E-07	6.3E-05	4.1E-04	459.2	475.3	341.2	166.1	189	122.4	2.4	26.4	20.1	19.1	48.4	18.6	No	No	No	hydroquinone glucosyltransferase, putative, expressed
Os.50261.1.S1_at	LOC_Os05g15150	3	1.5E-05	1.2E-06	1.2E-05	2.4E-04	3.0E-04	6.4E-03	188.5	190.8	131.7	52.2	35.5	36	88.4	87.2	52.5	78.8	57.4	61.6	No	No	No	leucyl-tRNA synthetase, cytoplasmic, putative, expressed
Os.4892.1.S1_at	LOC_Os05g15530	4	2.8E-05	2.8E-09	3.6E-05	3.9E-04	6.7E-06	1.3E-02	546.9	589.3	615.2	50.6	109.3	106.7	281.3	326.2	377.4	68.1	100.6	80.6	Yes	No	No	4-amino-4-deoxychorismate lyase, putative, expressed
Os.53842.2.S1_x_at	LOC_Os05g20620	3	9.3E-09	2.9E-07	1.8E-07	1.4E-06	1.2E-04	4.3E-04	91.6	36.7	55.7	8.4	9.3	50.8	8.1	13.3	53.1	12.1	55.9	13.2	No	No	No	retrotransposon protein, putative, unclassified, expressed
Os.53753.1.S1_at	LOC_Os05g25430	7	1.9E-07	3.9E-05	3.9E-05	1.0E-05	3.2E-03	1.3E-02	37.6	54.9	36.3	38.6	41.3	36.9	271.5	389.3	329.9	93.3	148.4	127.3	No	No	No	protein kinase, putative, expressed
Os.21300.2.S1_at	LOC_Os05g25974	4	1.6E-03	8.8E-07	6.1E-04	8.8E-03	2.4E-04	7.9E-02	248.4	353.8	275.2	142.5	182.5	163.4	372.4	497.8	470.1	140.5	159.1	127.9	No	No	No	expressed protein
Os.49558.1.S1_at	LOC_Os05g29170	4	7.8E-09	5.9E-04	5.0E-04	1.3E-06	2.1E-02	7.0E-02	920.9	718.7	861.7	622.3	669.5	579.3	66.4	73	184.3	108.1	134.2	106.7	No	No	No	expressed protein
Os.9300.1.S1_at	LOC_Os05g29710	4	3.4E-08	1.1E-06	1.1E-04	3.3E-06	2.8E-04	2.7E-02	171.2	181.2	167.4	125.1	118.2	122.4	494.3	467.6	576.5	305	252.6	309	No	No	No	RING-H2 finger protein ATL5F, putative, expressed
Os.23542.1.A1_at	LOC_Os05g29880	3	1.2E-08	1.6E-03	1.7E-03	1.7E-06	4.2E-02	1.4E-01	569.2	631.2	659.9	1045.4	716.5	992.5	1421.1	1227.7	1243.1	2219.8	1844.3	1641.8	Yes	No	No	glyoxysomal fatty acid beta-oxidation multifunctional protein MFP-a, putative, expressed
Os.49685.1.S1_at	LOC_Os05g30940	4	5.6E-04	5.3E-07	2.3E-04	3.9E-03	1.7E-04	4.3E-02	23.9	57.4	24.2	96.6	61.6	54.1	2.5	39.9	13	82.1	106.4	61.6	No	Yes	Yes	vacuolar calcium binding protein, putative, expressed
OsAffx.14900.1.S1_at	LOC_Os05g31470	4	9.1E-06	2.2E-06	7.7E-05	1.7E-04	4.4E-04	2.1E-02	54.4	5.6	2.7	77.7	137.4	95	1.4	7.2	1.9	12.1	0.9	12.9	No	Yes	Yes	expressed protein
OsAffx.14900.1.S1_s_at	LOC_Os05g31470	3	1.0E-08	4.0E-07	3.6E-07	1.5E-06	1.5E-04	6.7E-04	165.8	176.6	150.6	381.7	473.4	442.9	7.2	45.2	58.4	187.8	207.3	161	No	Yes	Yes	expressed protein
Os.6223.1.S1_at	LOC_Os05g33110	5	6.8E-08	6.7E-04	1.5E-03	5.2E-06	2.3E-02	1.4E-01	3921.5	3934.7	3913.3	2746.6	2398.8	2986	1454.3	1336.9	1480.3	1447	1040.4	1376.7	Yes	Yes	Yes	endo-1,3;1,4-beta-D-glucanase precursor, putative, expressed
OsAffx.27135.1.S1_at	LOC_Os05g33200	8	8.7E-04	7.9E-04	8.9E-04	5.5E-03	2.5E-02	1.0E-01	13.4	1.7	12.6	114.4	25.9	55.8	22.5	47.5	15.9	833.4	650.5	451.1	Yes	Yes	Yes	retrotransposon protein, putative, Ty1-copia subclass
Os.47979.1.A1_at	LOC_Os05g33220	5	4.8E-09	4.8E-06	4.9E-06	9.5E-07	7.5E-04	3.4E-03	333	321.2	310.2	553.7	478.8	484.3	3.7	3.7	5.5	2.6	4.6	4.7	Yes	Yes	Yes	plasma membrane associated protein, putative, expressed
OsAffx.27149.1.S1_at	LOC_Os05g34030	3	3.6E-04	2.9E-04	2.7E-04	2.7E-03	1.3E-02	4.7E-02	16	35.5	5.8	79.4	5.3	9.6	33.6	13.3	13.9	221.6	134.3	122.6	No	No	No	peptide transporter PTR2, putative, expressed
OsAffx.27188.2.S1_at	LOC_Os05g35830	5	2.7E-05	3.0E-05	3.6E-05	3.8E-04	2.7E-03	1.3E-02	9.7	38.7	45.9	468.9	371.2	534.4	25.6	2.4	36.6	84.8	65.3	106.7	No	No	Yes	expressed protein
Os.5469.1.S1_at	LOC_Os05g38264	7	2.4E-05	8.2E-09	2.7E-05	3.5E-04	1.2E-05	1.1E-02	1924	2121.6	2316.5	503.4	406	586.1	1182.9	1469.3	1268.2	512.3	368.5	471.9	No	No	Yes	expressed protein
Os.12348.5.S1_at	LOC_Os05g38740	3	7.0E-09	1.6E-04	1.7E-04	1.2E-06	8.4E-03	3.5E-02	1983.2	1567.7	2466.5	3289.2	2837.1	3157.9	198.9	128.2	199.7	162.9	197.6	185.9	Yes	No	No	histone H4, putative, expressed

Probe Set ID	Locus ID	probe #	ANOVA p-value			FDR q-value			Nipponbare MV			Nipponbare CK			93-11 MV			93-11 CK			Indel (Indica vs Japonica)			Annotation
			C	T	C×T	C	T	C×T	Repeat 1	Repeat 2	Repeat 3	Repeat 1	Repeat 2	Repeat 3	Repeat 1	Repeat 2	Repeat 3	Repeat 1	Repeat 2	Repeat 3	upstream	gene	downstream	
Os.49683.1.S1_at	LOC_Os05g38770	4	5.8E-08	9.0E-09	6.3E-08	4.7E-06	1.3E-05	2.2E-04	5.5	42.7	16.4	99.7	45.1	54.2	28.4	33.1	36.9	115.5	145	126	Yes	Yes	Yes	protein kinase APK1B, chloroplast precursor, putative, expressed
OsAffx.15031.1.S1_at	LOC_Os05g39860	3	2.8E-05	1.2E-05	3.6E-04	3.9E-04	1.4E-03	5.6E-02	369.9	252	298	152.2	122.2	115.4	140.1	95.2	150.4	99.8	95.1	141.7	No	No	No	expressed protein
Os.8150.1.S1_a_at	LOC_Os05g42350	9	1.6E-06	2.0E-07	1.1E-05	4.5E-05	9.2E-05	6.1E-03	3574.3	3623.5	3211.8	1581.9	1276.4	1158.2	1881.7	1944.6	2183.3	1363.7	1248.8	1320.9	No	No	No	sulfite reductase, putative, expressed
Os.8150.1.S1_at	LOC_Os05g42350	8	2.0E-06	8.0E-09	6.0E-06	5.4E-05	1.2E-05	3.9E-03	4076.2	4291.9	3967.2	1512.6	1429.1	1643.8	2431.5	2364.2	2550.9	1576.5	1418.2	1446.7	No	No	No	sulfite reductase, putative, expressed
Os.8150.2.S1_x_at	LOC_Os05g42350	9	3.7E-06	3.8E-07	2.3E-05	8.6E-05	1.4E-04	9.7E-03	3067.5	3155.1	2909.5	1342.4	1279.6	1222.4	1734.1	1771.2	1701.9	1359.2	1225.9	1161.8	No	No	No	sulfite reductase, putative, expressed
Os.55637.1.S1_at	LOC_Os05g45070	7	2.6E-09	9.4E-10	7.4E-09	6.6E-07	3.5E-06	5.8E-05	116.4	158.9	134.7	386.5	169.3	171.2	88.4	128.1	113.8	871	973	1027	Yes	No	No	harpin-induced protein, putative, expressed
Os.9327.1.S1_at	LOC_Os05g45460	3	2.1E-06	2.9E-05	5.6E-05	5.6E-05	2.6E-03	1.7E-02	574.7	564.6	595.4	1638.2	1898.5	2578.4	247.6	163	203.9	1448.1	840.3	1372.6	No	Yes	Yes	pistil-specific extensin-like protein precursor, putative, expressed
Os.7325.1.S1_at	LOC_Os05g45840	5	3.6E-05	2.2E-04	1.1E-03	4.7E-04	1.0E-02	1.2E-01	1494.5	1597.4	1568.8	1000	879.8	1005.3	769.2	786.1	969.4	715.1	655.6	893.2	No	No	No	GTP-ase activating protein for Arf containing protein, putative, expressed
Os.8961.1.S1_s_at	LOC_Os05g46020	4	3.1E-07	3.6E-06	7.5E-04	1.4E-05	6.3E-04	9.0E-02	714.6	574	704.2	342.2	303.3	316.7	241.4	245.4	245.1	184	149.3	139	No	No	No	OsWRKY7 - Superfamily of rice TFs having WRKY and zinc finger domains, expressed
OsAffx.4639.1.S1_at	LOC_Os05g46610	3	2.2E-08	6.4E-05	4.3E-04	2.5E-06	4.5E-03	6.3E-02	520.2	384.1	459.9	840.9	625.1	681.6	372.1	337.6	409.3	447.2	370.3	406.2	No	No	No	myb-related protein Hv33, putative, expressed
OsAffx.27357.2.S1_at	LOC_Os05g47750	6	1.3E-09	2.4E-06	9.5E-06	4.4E-07	4.8E-04	5.4E-03	688.2	672.3	588.8	370.4	387.7	362.1	45.4	94.1	74.1	38	112.4	65.8	No	Yes	Yes	Ser/Thr protein kinase, putative, expressed
Os.53178.1.S1_s_at	LOC_Os05g47840	4	2.6E-07	1.7E-06	2.7E-06	1.3E-05	3.8E-04	2.4E-03	45.6	32.3	35.9	76.4	80.3	59.6	112.5	163.9	118.7	260.3	413.1	284.6	No	No	No	ATIPT1, putative, expressed
OsAffx.27370.1.S1_at	LOC_Os05g48570	6	1.3E-06	4.2E-05	2.1E-05	3.9E-05	3.3E-03	9.2E-03	187	213.4	246.4	213.7	208.7	227.3	230.3	314.7	347.8	1040.1	873.6	911.2	No	No	Yes	conserved hypothetical protein
OsAffx.15154.1.S1_at	LOC_Os05g48650	6	1.6E-09	3.0E-06	2.7E-06	4.8E-07	5.5E-04	2.4E-03	416.5	355.3	387.2	235.9	170.8	114.6	9.7	13	10.3	3.5	3	14.3	No	No	No	tumor-related protein-like, putative, expressed
Os.13704.1.S1_x_at	LOC_Os05g48910	8	3.3E-10	3.2E-10	2.4E-10	2.0E-07	2.0E-06	8.4E-06	185.9	293.6	200.4	9.5	25.6	11.6	11.7	10.8	31.4	2.3	23.4	5.2	No	No	No	retrotransposon protein, putative, unclassified
Os.5124.1.S1_at	LOC_Os05g49770	11	2.7E-07	4.8E-10	1.7E-07	1.3E-05	2.5E-06	4.1E-04	2134.9	1939	1868.4	719.2	601.5	710	1170.3	1150	1141.4	590.1	643	739.8	No	No	No	CTP synthase, putative, expressed
Os.5124.1.S1_s_at	LOC_Os05g49770	5	2.6E-04	3.4E-07	1.2E-04	2.2E-03	1.3E-04	2.7E-02	3734.5	3951.2	4101.4	1356.6	1471.4	1763.2	2927	2960.3	3005.2	1564.6	1698.1	2103.4	No	No	No	CTP synthase, putative, expressed
Os.56284.1.S1_at	LOC_Os05g50580	3	2.4E-06	2.3E-04	2.8E-04	6.2E-05	1.1E-02	4.8E-02	86.6	70	61.3	51.3	70.2	90	10.7	37.9	26.1	3.6	16.7	4.9	No	Yes	Yes	SCPL50, putative, expressed
Os.10131.1.S1_a_at	LOC_Os05g51670	7	2.0E-05	5.1E-07	5.3E-06	3.0E-04	1.7E-04	3.6E-03	2583.6	2825.4	2678.1	1320	1158.1	1166.6	1400.4	2066.9	1586.3	1479.3	1256.6	1414.6	No	No	No	UDP-glucose 4-epimerase GEPI48, putative, expressed
Os.8578.1.S1_at	LOC_Os06g01350	7	7.2E-08	2.6E-08	2.7E-06	5.4E-06	2.5E-05	2.4E-03	1786.6	2135.6	2392.3	737.6	939.3	892.3	897.9	1087.3	945.2	665.3	506.6	726	No	No	No	transferase, putative, expressed
Os.11622.1.S1_at	LOC_Os06g01360	6	9.1E-07	1.5E-06	2.2E-05	3.1E-05	3.5E-04	9.4E-03	1449.7	2137.2	1753.1	552.4	580.6	525.5	818.1	723.5	614.9	432.6	569	552.4	No	No	No	homogentisate 1,2-dioxygenase, putative, expressed
OsAffx.22609.1.S1_x_at	LOC_Os06g01920	6	1.8E-05	3.7E-07	3.3E-06	2.8E-04	1.4E-04	2.7E-03	185.5	161.1	167.1	882.2	395.1	587.4	199.8	170.2	203.4	1569	1335	1763.3	Yes	No	No	alpha-expansin 7 precursor, putative, expressed
Os.39933.1.S1_x_at	LOC_Os06g02440	3	1.5E-04	1.5E-05	1.3E-03	1.4E-03	1.7E-03	1.2E-01	275.9	197.1	264.6	97.6	111.7	87.4	98.1	124.6	137.8	57.2	64.6	68.8	No	No	No	expressed protein
Os.4518.1.S1_a_at	LOC_Os06g02490	9	4.7E-06	2.4E-06	2.6E-05	1.0E-04	4.7E-04	1.0E-02	961.5	927.1	1017.6	1248.4	1040.3	1133.4	1015	1387.6	1183.8	2359	2624.4	2544	No	No	Yes	acyl-CoA-binding protein, putative, expressed
Os.4518.2.S1_x_at	LOC_Os06g02490	7	1.4E-06	9.6E-07	1.3E-05	4.1E-05	2.6E-04	6.8E-03	1549.1	1313.9	1420.6	2013.6	1313.1	1577.2	1556.4	1688.4	1863.6	2962.7	3237.7	3513.4	No	No	Yes	acyl-CoA-binding protein, putative, expressed
Os.17677.1.S1_at	LOC_Os06g03810	11	1.6E-06	8.1E-06	8.3E-06	4.5E-05	1.1E-03	4.9E-03	2276.2	2740.3	2528.5	1028.9	1164.9	1022.7	596.5	1038.6	587.7	767.8	394.5	488.6	No	No	No	expressed protein
Os.606.1.S1_at	LOC_Os06g04220	5	5.1E-06	6.8E-10	2.4E-06	1.1E-04	3.1E-06	2.2E-03	671.7	573.8	826.7	87	154.2	77.4	376.6	403.3	427	171	144.8	170.9	No	No	No	expressed protein
Os.4773.1.S1_at	LOC_Os06g04240	6	1.5E-04	1.2E-07	5.6E-05	1.4E-03	6.5E-05	1.7E-02	2258.3	1883.6	2286.4	390	383.1	437.9	1105.4	1229.3	993.2	592.3	354.8	443.4	No	No	No	Q-rich domain protein, putative, expressed
Os.7031.1.S1_at	LOC_Os06g04450	4	4.5E-06	4.1E-08	5.3E-06	9.8E-05	3.3E-05	3.6E-03	57	38.3	42.9	170.5	168.8	136.7	73.5	38.6	94.6	318.1	332.8	321.3	No	No	No	SNARE-interacting protein KEULE, putative, expressed
Os.21318.1.S1_at	LOC_Os06g04510	5	1.7E-06	1.4E-10	5.8E-07	4.7E-05	1.5E-06	9.1E-04	10337.5	8961.2	9648.2	797.1	702.2	704.5	6030	7084.5	6430.4	969.4	1152.8	1138.4	No	No	No	enolase 1, putative, expressed
Os.38638.1.S1_at	LOC_Os06g04990	3	1.2E-05	1.6E-03	4.2E-04	2.0E-04	4.2E-02	6.2E-02	867.8	1640.9	1372	2205.8	2979.5	3627.9	814.7	416.4	703.9	520	353.3	461	Yes	No	No	early nodulin 93, putative, expressed
Os.10862.1.S1_at	LOC_Os06g05410	8	1.2E-08	1.6E-08	1.6E-08	1.7E-06	1.9E-05	9.1E-05	928.2	744.2	761.9	73.3	62.2	48.7	11.3	29.8	24.3	1.3	21	14	Yes	Yes	Yes	expressed protein
Os.50399.1.S1_at	LOC_Os06g05420	10	1.0E-08	4.0E-08	4.0E-08	1.5E-06	3.3E-05	1.6E-04	1573.6	1143.2	1503	161.5	216.4	205.8	25.8	6.6	2.2	20.9	4.4	6	Yes	No	Yes	expressed protein
Os.53206.1.S1_at	LOC_Os06g05424	9	3.1E-08	1.7E-07	1.6E-07	3.0E-06	8.4E-05	4.0E-04	597.4	657.7	604.1	76.2	55.3	86.1	13.3	4.6	5.8	3.8	1.8	3.9	Yes	Yes	Yes	expressed protein
OsAffx.4731.1.S1_s_at	LOC_Os06g05430	4	8.9E-09	3.5E-04	1.3E-03	1.4E-06	1.4E-02	1.2E-01	407.8	405.8	333.8	664.6	525	639.6	162.6	151	228.4	165.3	144.2	175.7	Yes	Yes	Yes	expressed protein
Os.10736.1.S1_at	LOC_Os06g05440	8	1.8E-10	3.8E-05	3.5E-05	1.4E-07	3.1E-03	1.3E-02	2827.3	2427.8	2719.4	1382.9	1846.1	1723.9	42.2	86.9	26.2	70.9	61.7	73	Yes	Yes	Yes	expressed protein
Os.51845.1.S1_x_at	LOC_Os06g05450	5	5.0E-07	2.6E-05	2.3E-05	2.0E-05	2.4E-03	9.7E-03	325.2	281.6	518.5	119.8	132.2	134.4	27.6	15.6	2.4	2.1	37.4	28.3	Yes	Yes	No	expressed protein
Os.2260.1.S1_at	LOC_Os06g05690	6	6.4E-09	9.7E-07	2.9E-05	1.1E-06	2.6E-04	1.1E-02	295.9	342.4	307.1	641.1	510.4	605.1	317.6	525.8	468.3	1442.1	940.2	1194.3	Yes	No	No	cysteine synthase, chloroplast precursor, putative, expressed
Os.15798.1.S1_at	LOC_Os06g07040	7	1.0E-07	3.7E-06	3.4E-06	6.8E-06	6.4E-04	2.7E-03	1004.6	1181.7	994.7	277	249.7	294	249.7	301.5	306.2	175.4	150.7	115.5	Yes	Yes	Yes	OsIAA20 - Auxin-responsive Aux/IAA gene family member, expressed
Os.18952.1.S1_at	LOC_Os06g07200	5	6.2E-07	1.1E-07	5.5E-05	2.3E-05	6.4E-05	1.7E-02	1987.8	2620.9	2471.7	780.8	1133.6	1053.3	985.8	1529.8	1316.2	520.8	608.8	812.7	No	No	No	syntaxin 132, putative, expressed
OsAffx.15295.1.S1_x_at	LOC_Os06g07210	5	3.5E-07	9.3E-08	7.8E-04	1.6E-05	5.7E-05	9.2E-02	1679.9	1577.6	1814.1	773.2	354.1	656.8	501.4	1064.8	510.4	273.8	220.1	227.9	Yes	No	No	ribonucleoside-diphosphate reductase large subunit, putative, expressed
Os.56006.1.S1_at	LOC_Os06g08610	3	1.8E-08	1.3E-04	2.4E-04	2.2E-06	7.4E-03	4.4E-02	595	681.2	563.6	753.1	940.6	725.3	19.1	16.1	11.7	43.3	22.9	39.7	Yes	Yes	Yes	anthranilate N-benzoyltransferase protein 1, putative, expressed
Os.55786.1.S1_at	LOC_Os06g09270	9	5.9E-09	9.8E-11	2.8E-08	1.1E-06	1.3E-06	1.4E-04	44.9	55.7	40.7	719.6	432.1	643.8	205.2	141.1	180	1266.1	1311.2	1510.5	No	No	No	hyprol, putative, expressed
OsAffx.27536.1.S1_x_at	LOC_Os06g09600	3	9.7E-05	1.8E-06	3.2E-05	1.0E-03	4.0E-04	1.2E-02	592.6	489.6	501.8	432.4	347.2	256.7	331.3	553	377.3	275.9	323.9	448	No	No	No	expressed protein
Os.54864.2.S1_at	LOC_Os06g09980	4	6.9E-05	1.1E-05	3.1E-03	7.7E-04	1.3E-03	2.0E-01	871.7	786.8	733.8	419.3	380.6	381.7	413.9	437.5	326.2	213	248.8	171	No	No	No	expressed protein
Os.39031.1.S1_at	LOC_Os06g09990	5	1.9E-07	5.4E-07	8.1E-04	1.0E-05	1.8E-04	9.5E-02	426.7	374.9	420	181.3	190.7	147.6	164.6	179.7	147.7	48	44.2	58.3	No	No	No	hypothetical protein
Os.53177.1.S1_at	LOC_Os06g10510	4	1.4E-06	8.2E-07	8.6E-04	4.1E-05	2.3E-04	9.7E-02	169.5	253.8	261.2	386.6	517.9	452.3	362.6	407.7	395.7	892	1166.4	990.7	No	No	Yes	expressed protein
Os.53177.1.S1_s_at	LOC_Os06g10510	3	2.1E-06	1.5E-06	1.1E-04	5.7E-05	3.4E-04	2.7E-02	274.3	318.7	202.2	464	569.6	488.2	276.8	561	473.9	966.6	992.2	1143.3	No	No	Yes	expressed protein
OsAffx.27555.1.S1_at	LOC_Os06g10580	8	2.0E-06	2.3E-06	3.9E-04	5.4E-05	4.6E-04	6.0E-02	1550.7	1670.8	1646.2	733	807.7	682.8	520.6	755.5	601.7	343.1	355	435.7	Yes	No	No	cyclic nucleotide-gated ion channel 19, putative, expressed
Os.11914.1.S1_at	LOC_Os06g11240	11	2.5E-11	1.9E-09	5.3E-09	4.8E-08	5.3E-06	4.8E-05	6884.2	7289.4	6554.2	3215.7	2776.6	3011.4	2083.8	2102.4	2173.1	1636.7	1548.6	1354.1	Yes	No	No	12-oxophytodienoate reductase 2, putative, expressed
Os.8778.1.S1_a_at	LOC_Os06g11290	9	6.2E-09	5.3E-10	3.5E-08	1.1E-06	2.6E-06	1.6E-04	5388.9	5337.6	5550.3	868.8	1007.9	858.1	1763.5	2199.2	2164.7	824	736	551.8	No	No	No	12-oxophytodienoate reductase 2, putative, expressed
Os.50807.1.S1_at	LOC_Os06g11650	5	4.8E-05	3.4E-07	5.2E-05	5.9E-04	1.3E-04	1.6E-02	117.5	143.5	177.5	255.8	296.3	410	131.9	132.9	133.2	319.5	431.6	429.8	Yes	No	No	erg-1, putative, expressed
OsAffx.15374.1.S1_s_at	LOC_Os06g11700	5	4.1E-07	2.6E-06	4.2E-06	1.7E-05	5.1E-04	3.2E-03	91.6	106.5	126.5	411.6	478.5	398.6	34.8	56.2	20.4	48	59.9	78.1	Yes	Yes	Yes	phi-1-like phosphate-induced protein, putative, expressed
Os.11771.1.S1_at	LOC_Os06g12320	5	1.5E-06	3.2E-09	1.4E-05	4.3E-05	7.2E-06	7.0E-03	38.3	44.6	10.7	206.1	149.1	203	77.9	92	40.5	332.3	369.3	376.8	No	No	No	amino acid/polyamine transporter II, putative, expressed
OsAffx.15391.1.S1_at	LOC_Os06g12500	3	7.3E-06	1.3E-07	2.3E-05	1.4E-04	7.1E-05	9.7E-03	38.3	17	32.9	158.1	99.4	157.4	49.1	33.6	34.6	258.8	221.2	286.3	No	No	No	plant integral membrane protein TIGR01569 containing protein, expressed
OsAffx.27606.1.S1_at	LOC_Os06g13220	4	6.9E-06	3.0E-05	6.2E-05	1.3E-04	2.7E-03	1.8E-02	1575	1195.1	879.5	291.4	271.8	99.2	204.3	238.7	192.9	133.2	87.3	101.8	Yes	Yes	Yes	growth regulator, putative, expressed
Os.57211.1.S1_at	LOC_Os06g13880	3	1.1E-04	1.4E-05	2.0E-03	1.1E-03	1.6E-03	1.6E-01	247	349.6	264.3	88.8	108.7	140.3	121.2	96.1	181	86.7	57.7	46	No	No	No	expressed protein
Os.17665.1.S1_x_at	LOC_Os06g14190	7	6.2E-06	8.3E-07	7.7E-05	1.2E-04	2.3E-04	2.1E-02	1627	1800.7	2026.4	953.9	747.5	910.2	995.1	1079.7	832.4	713.4	735.8	599.7	Yes	Yes	No	transcriptional repressor NF-X1, putative, expressed
Os.53612.1.S1_at	LOC_Os06g14450	4	4.1E-09	3.8E-06	5.5E-05	8.5E-07	6.4E-04	1.7E-02	167.8	185.3	101	107.8	99.4	82.9	569.4	986.1	678.7	412.2	339.7	400.5	No	No	No	protein binding protein, putative, expressed
Os.54493.1.S1_at	LOC_Os06g14490	8	1.2E-05	8.1E-05	1.4E-04	2.0E-04	5.2E-03	3.2E-02	1476.6	1431.7	1594.3	860.3	724	865.1	829.2	833.7	708.2	959.7	637.7	582	No	No	No	calmodulin-binding heat-shock protein, putative, expressed
Os.54615.1.S1_at	LOC_Os06g18140	10	4.3E-10	8.7E-11	4.9E-10	2.3E-07	1.2E-06	1.2E-05	1500.9	1288.4	1319.9	85.5	86.8	65.9	396.9	552.5	435.3	54.9	74.2	72.6	No	No	No	indole-3-acetate beta-glucosyltransferase, putative, expressed
Os.9889.1.S1_at	LOC_Os06g19730	11	7.1E-08	3.1E-06	3.8E-05	5.3E-06	5.7E-04	1.3E-02	8697.3	8887.2	8159.8	3946.4	3709	4618.7	3901.4	4088.1	4123.5	3069.9	2764.1	3437.9	No	Yes	No	HEAT repeat family protein, expressed
Os.54595.1.S1_at	LOC_Os06g19800	3	1.7E-03	7.0E-05	1.7E-03	9.3E-03	4.8E-03	1.5E-01	456.1	482.6	402.2	1004.5	384.9	511.6	678.3	369.2	263	1350.7	1029.7	1358.2	No	Yes	No	BURP domain containing protein, expressed
Os.12376.1.S1_at	LOC_Os06g19990	4	4.1E-08	1.8E-07	9.1E-04	3.7E-06	8.7E-05	1.0E-01	3005.1	2620.4	3122.3	1825.7	1656	1895	2404.8	1814.6	2612.4	1457.5	1371.1	1596	No	No	No	GPI-anchored protein, putative, expressed
Os.16879.1.S1_at	LOC_Os06g20820	6	1.2E-07	2.0E-06	1.2E-05	7.7E-06	4.3E-04	6.4E-03	816.9	2093.5	1693.1	298.4	1084	281.6	251.4	527.7	264.2	214.2	346.9	131.6	Yes	No	No	expressed protein
OsAffx.27741.1.S1_s_at	LOC_Os06g20820	10	1.1E-07	8.0E-07	8.4E-06	7.0E-06	2.3E-04	5.0E-03	2070.9	1537.4	2085.2	803.7	521.6	692.2	359.4	468.1	375.9	244.9	253	211.6	Yes	No	No	expressed protein
Os.51974.1.S1_at	LOC_Os06g20860	7	6.0E-07	2.8E-07	6.1E-07	2.3E-05	1.2E-04	9.4E-04	72.4	74.7	35.7	99	110.8	100.5	34.5	52.3	50.6	227.5	417.1	455	No	No	No	paramyosin, putative, expressed
Os.50192.1.S1_at	LOC_Os06g22600	3	2.2E-05	2.3E-06	2.2E-05	3.2E-04	4.7E-04	9.4E-03	25	38.1	4.8	72.2	5.5	62.2	3.1	16.1	21.2	68.8	109.4	102.9	No	No	No	ALMT1, putative, expressed
OsAffx.4907.1.S1_x_at	LOC_Os06g22919	7	1.1E-06	4.7E-08	1.4E-06	3.6E-05	3.6E-05	1.5E-03	155.5	71.8	89.9	465.3	231.8	485	127.5	13.6	64.5	1270.8	868.3	1324.2	Yes	Yes	No	xyloglucan endotransglucosylase/hydrolase protein 21 precursor, putative, expressed
OsAffx.27815.1.S1_s_at	LOC_Os06g24990	8	4.6E-10	1.4E-05	1.5E-05	2.4E-07	1.6E-03	7.3E-03	3.2	65.9	5.7	4.8	60.8	4.2	3875.1	3963.2	3240.6	2281	2231	2249.2	Yes	Yes	Yes	xylanase inhibitor protein 1 precursor, putative, expressed
OsAffx.27816.1.S1_at	LOC_Os06g25010	5	7.8E-10	1.4E-06	6.5E-06	3.2E-07	3.3E-04	4.2E-03	336.3	287.1	278.3	231.7	129.3	169.6	1247.7	1047.6	1152.3	737	569.3	886.5	Yes	Yes	Yes	xylanase inhibitor protein 1 precursor, putative, expressed
Os.28593.1.S1_at	LOC_Os06g31090	3	7.4E-04	1.1E-07	1.8E-04	4.8E-03	6.2E-05	3.7E-02	318.2	200.2	355.8	2.9	3	2.8	179.9	180.7	213.7	43.3	29.8	32.7	No	No	Yes	GAMYB-binding protein, putative, expressed
OsAffx.27942.1.S1_at	LOC_Os06g34560	3	9.1E-08	1.6E-05	1.2E-03	6.2E-06	1.8E-03	1.2E-01	359.7	290.4	428.7	36.9	68.3	54.6	116.8	121.9	122.4	28.6	20.6	28.2	Yes	No	No	RING-H2 finger protein ATL2G precursor, putative
Os.8956.1.S1_at	LOC_Os06g36210	4	4.5E-05	3.7E-05	3.4E-05	5.6E-04	3.1E-03	1.2E-02	58.4	57.1	101.3	74.6	155.2	71.9	317.3	444.5	193.4	49.3	10.8	42.8	No	No	No	amino acid permease 1, putative, expressed
Os.40406.1.A1_at	LOC_Os06g38110	6	2.7E-07	3.9E-06	2.5E-06	1.3E-05	6.6E-04	2.3E-03	105.6	143.9	102.9	26.4	15.9	49.2	24.8	4.6	17	3.6	11.8	6.6	Yes	Yes	Yes	hypothetical protein
OsAffx.5041.1.S1_at	LOC_Os06g38110	4	2.3E-06	4.1E-06	8.2E-06	6.0E-05	6.8E-04	4.9E-03	145.4	91.4	90.3	18	37.8	26.9	24.8	16.2	20.1	2.6	19	1.2	Yes	Yes	Yes	hypothetical protein
Os.12027.1.S1_a_at	LOC_Os06g38210	3	6.2E-14	1.4E-04	8.6E-05	2.3E-09	7.7E-03	2.2E-02	17730.5	18563.9	17651.6	18148.3	13668	19122.2	72.6	264.1	15.6	341.1	204.3	17	Yes	Yes	Yes	expressed protein
Os.53438.1.S1_at	LOC_Os06g38580	6	1.4E-09	2.9E-05	1.4E-04	4.5E-07	2.6E-03	3.0E-02	1913.4	1958.2	1914	2915.8	2854.5	3221.1	434.7	534.6	487.2	671.5	515.7	881.5	Yes	Yes	No	hypothetical protein
Os.8503.1.S1_at	LOC_Os06g39110	6	1.1E-07	3.2E-05	9.4E-04	7.1E-06	2.8E-03	1.0E-01	6098.4	6432.4	5576.4	3361.1	3195.2	3364.6	795.8	684.1	775.1	263.6	386.9	351.5	No	No	Yes	expressed protein
Os.11046.1.S1_at	LOC_Os06g39240	11	1.3E-09	2.3E-08	3.1E-07	4.3E-07	2.4E-05	6.0E-04	7467.4	8641.8	9284.3	4534	5555.7	4479.9	4135.1	4793.1	4391.4	2810.5	2741.6	3980.3	Yes	No	No	endothelial differentiation-related factor 1, putative, expressed
OsAffx.28012.1.S1_at	LOC_Os06g39800	5	9.6E-06	8.1E-04	7.8E-04	1.7E-04	2.6E-02	9.2E-02	887.1	668.4	802.8	258	275.6	294.3	82.6	107.8	126.6	55.4	66.1	146.3	Yes	Yes	Yes	hypothetical protein
Os.5614.1.S1_at	LOC_Os06g42030	5	5.3E-06	2.5E-04	2.7E-04	1.1E-04	1.1E-02	4.7E-02	1267.1	1413.9	1416.8	1284	1258	1501	1447.9	1866.3	1930.6	2817.2	2452.3	2631	Yes	No	No	potassium transporter 10, putative, expressed
Os.49526.1.S1_a_at	LOC_Os06g42560	4	1.8E-06	7.7E-06	3.5E-04	5.1E-05	1.0E-03	5.5E-02	4355.1	3417.9	3580.1	1949.4	1294.3	1644.1	1874.4	2123	2084.1	1006.7	958.7	1045.9	Yes	No	Yes	tryptophan synthase beta chain 2, putative, expressed
Os.56222.1.S1_at	LOC_Os06g43170	3	9.3E-05	1.6E-04	2.1E-04	9.7E-04	8.3E-03	4.0E-02	94.5	141.3	120.9	8.9	31.7	60.3	11.6	28.8	8.5	5	3.7	8.4	Yes	Yes	No	transposon protein, putative, unclassified, expressed
Os.51521.1.S2_at	LOC_Os06g43980	3	6.9E-07	1.8E-04	2.4E-03	2.5E-05	9.1E-03	1.8E-01	65.8	117.6	104.4	58.3	56	97.3	223.4	242.2	231.5	142.4	148.8	117.1	No	Yes	Yes	expressed protein
Os.50015.1.S1_at	LOC_Os06g44010	6	8.4E-05	1.9E-07	2.1E-04	9.0E-04	9.1E-05	4.0E-02	1754.7	1618.8	1401.2	278.3	412.3	303.3	817.8	1250.5	927.2	429.9	193.8	233.1	No	No	No	OsWRKY28 - Superfamily of rice TFs having WRKY and zinc finger domains, expressed
Os.8559.1.S1_at	LOC_Os06g44190	5	6.3E-06	6.0E-05	1.7E-04	1.3E-04	4.2E-03	3.5E-02	288.3	402.5	305.3	170.4	144.2	147.3	100.6	160.9	126.1	124.9	175.5	113.4	No	No	Yes	expressed protein
Os.25409.1.S1_at	LOC_Os06g45184	7	9.0E-07	4.9E-07	7.4E-07	3.0E-05	1.6E-04	1.0E-03	441.9	456.6	319.7	1.7	3.9	12.5	19.2	18.1	36.4	9.1	12.7	10.1	No	No	No	retrotransposon protein, putative, Ty3-gypsy subclass, expressed

Probe Set ID	Locus ID	probe #	ANOVA p-value			FDR q-value			Nipponbare MV			Nipponbare CK			93-11 MV			93-11 CK			Indel (Indica vs Japonica)			Annotation
			C	T	C×T	C	T	C×T	Repeat 1	Repeat 2	Repeat 3	Repeat 1	Repeat 2	Repeat 3	Repeat 1	Repeat 2	Repeat 3	Repeat 1	Repeat 2	Repeat 3	upstream	gene	downstream	
Os.25409.1.S1_x_at	LOC_Os06g45184	9	1.8E-08	1.8E-08	1.8E-08	2.2E-06	2.0E-05	1.0E-04	466.5	453.5	394.4	0.9	31.6	17.6	37.7	42.7	48.9	8.2	19.9	9.2	No	No	No	retrotransposon protein, putative, Ty3-gypsy subclass, expressed
Os.24196.1.A1_x_at	LOC_Os06g45200	3	3.1E-05	5.5E-06	2.5E-05	4.2E-04	8.2E-04	1.0E-02	31.5	13	26.3	1.5	7.3	11.7	4.9	31.4	12	6.1	10.5	25.7	No	No	No	pollen allergen Phl p 2 precursor, putative
Os.55395.1.S1_s_at	LOC_Os06g45570	4	3.9E-06	3.5E-07	5.1E-04	8.8E-05	1.3E-04	7.1E-02	1030.6	907.7	783.3	585.1	516.8	359.8	449.9	535.9	463.4	418.5	323.1	388	No	No	No	VQ motif family protein, expressed
Os.52280.1.S1_at	LOC_Os06g46149	5	9.4E-08	5.1E-08	2.9E-07	6.4E-06	3.8E-05	5.8E-04	318.9	240.3	239.1	60.6	71.2	56.2	80.4	100.3	95	48.6	53.6	87.5	No	Yes	Yes	expressed protein
Os.55380.1.S1_at	LOC_Os06g46740	6	9.4E-06	8.0E-08	9.8E-07	1.7E-04	5.1E-05	1.3E-03	736.5	536.9	612.7	70.6	79.6	80.9	202.3	204.5	231.3	122.8	166.1	176.7	No	No	No	early nodulin 20 precursor, putative, expressed
OsAffx.28152.2.S1_at	LOC_Os06g47470	3	2.4E-06	3.6E-06	8.6E-05	6.2E-05	6.3E-04	2.2E-02	200	248	179.7	115.6	130	95.8	90.3	134.3	158.8	70.6	80.6	55.3	No	Yes	Yes	protein kinase domain containing protein, expressed
OsAffx.28152.1.S1_at	LOC_Os06g47530	6	4.6E-08	3.0E-05	3.8E-05	4.0E-06	2.7E-03	1.3E-02	271.5	293.3	208.8	246.2	148	141.4	10.7	48.6	80.9	36	37.1	21.7	No	No	Yes	serine/threonine-protein kinase receptor precursor, putative, expressed
OsAffx.28152.1.S1_s_at	LOC_Os06g47530	4	1.7E-09	5.1E-07	3.2E-06	5.0E-07	1.7E-04	2.7E-03	1025.8	862.8	899.5	576.1	505.6	460.9	196.5	212	212.9	212	209.2	139.8	No	No	Yes	serine/threonine-protein kinase receptor precursor, putative, expressed
OsAffx.15978.1.S1_at	LOC_Os06g48010	4	2.0E-06	5.6E-07	1.4E-06	5.3E-05	1.8E-04	1.6E-03	112.6	87.6	176.6	512.7	420.3	540.3	65	223.7	256.9	152.3	105.9	311.5	No	No	Yes	peroxidase 16 precursor, putative, expressed
OsAffx.15978.1.S1_x_at	LOC_Os06g48010	3	9.5E-05	2.9E-05	7.5E-05	9.9E-04	2.6E-03	2.1E-02	386.7	310.4	315.9	841.1	710.4	767.5	239.6	358.3	361.7	363.1	308.5	485.9	No	No	Yes	peroxidase 16 precursor, putative, expressed
Os.9637.1.S1_at	LOC_Os06g48050	9	1.1E-07	3.8E-08	2.1E-07	6.9E-06	3.2E-05	4.7E-04	46	94.1	116.8	186.1	150.4	168.9	65.4	90.3	88.5	517.5	528.2	575.8	No	No	No	expressed protein
OsAffx.28169.2.S1_at	LOC_Os06g48240	3	9.7E-06	3.9E-04	4.8E-04	1.7E-04	1.6E-02	6.8E-02	413	282.3	385.2	258.5	177	168.8	85.2	77.3	122	69.9	52.7	70	Yes	Yes	No	ATPase protein, putative, expressed
Os.56682.1.S1_at	LOC_Os06g50940	6	1.2E-05	2.0E-07	1.4E-03	2.0E-04	9.3E-05	1.3E-01	1673.8	1596.7	1965.7	484.4	713	427.9	779.4	1140.8	752.1	314.4	406.6	338.4	Yes	No	No	anther-specific proline-rich protein APG precursor, putative, expressed
Os.51172.1.S1_x_at	LOC_Os06g51050	3	2.4E-08	3.2E-04	3.5E-04	2.6E-06	1.3E-02	5.5E-02	1359.8	1614.6	1647.9	1057.5	963.3	1222	111.1	69.8	65.2	73.8	107.3	81.5	No	No	No	basic endochitinase 1 precursor, putative, expressed
Os.30269.1.S1_at	LOC_Os07g01340	5	1.8E-07	6.0E-07	9.7E-07	9.9E-06	1.9E-04	1.2E-03	222	166.5	205.9	68.6	99.7	43.2	76.9	48.6	55.2	43.9	63.1	35.4	Yes	Yes	Yes	gibberellin 2-beta-dioxygenase 7, putative, expressed
OsAffx.28221.1.S1_at	LOC_Os07g01420	9	1.5E-04	7.2E-06	2.1E-04	1.4E-03	1.0E-03	4.0E-02	3.8	3.8	5	317.4	157.7	210.9	4.2	5.8	10.7	811.6	506.3	863.8	No	No	Yes	peroxidase 1 precursor, putative
Os.30866.1.S1_at	LOC_Os07g02460	6	1.1E-06	1.2E-04	2.8E-05	3.4E-05	6.7E-03	1.1E-02	625.1	710	616.2	573.8	497.4	526.4	698.6	694	937.7	1540.6	1288.7	1521.8	No	No	Yes	expressed protein
OsAffx.5189.1.S1_at	LOC_Os07g02870	11	3.2E-08	6.6E-08	8.6E-08	3.2E-06	4.5E-05	2.7E-04	3.3	57.1	6	122.1	57.7	26.4	67.5	101.5	46.2	1275.3	1517.3	1129.1	No	No	No	conserved hypothetical protein
Os.40003.1.S1_x_at	LOC_Os07g02920	3	3.0E-05	1.2E-05	2.7E-05	4.1E-04	1.4E-03	1.1E-02	34.7	66	36.6	49.3	46.1	42	23.9	34.6	58.4	85.9	144.9	102.8	No	No	No	expressed protein
Os.32682.1.S1_at	LOC_Os07g02960	10	4.6E-09	9.5E-10	1.4E-08	9.2E-07	3.5E-06	8.4E-05	1374.3	1424.8	1689.6	259.7	273.8	337.6	426	613	468.1	126.6	195.6	171.7	No	No	No	expressed protein
Os.32682.2.S1_x_at	LOC_Os07g02960	11	1.3E-06	9.9E-08	5.1E-06	3.9E-05	5.9E-05	3.5E-03	1330.5	1124.9	1263.1	266.2	231	324.7	336.5	393.8	510.8	171.3	153.9	204.7	No	No	No	expressed protein
OsAffx.16056.1.S1_at	LOC_Os07g03020	3	2.2E-04	2.4E-05	1.8E-04	1.9E-03	2.3E-03	3.6E-02	133.6	147.2	99.3	2.4	7.3	29.7	4.7	16.7	8.5	5.8	6.8	2.7	Yes	No	No	hypothetical protein
OsAffx.16082.1.S1_at	LOC_Os07g04560	10	3.9E-06	5.0E-07	1.1E-05	8.8E-05	1.7E-04	6.2E-03	390.5	281.6	376.3	50.5	53.7	52	102.4	80.9	121.7	10.5	33.9	23.4	Yes	No	No	NAC domain-containing protein 94, putative
OsAffx.28276.1.S1_x_at	LOC_Os07g04970	7	1.8E-07	1.5E-08	7.8E-07	1.0E-05	1.8E-05	1.1E-03	1650.6	1027.5	1101.9	335.7	128.7	113.8	412.2	402.5	365.4	93.2	65.2	27.1	No	No	No	transferase, putative, expressed
OsAffx.16129.1.S1_at	LOC_Os07g06800	3	7.6E-08	1.6E-04	4.9E-04	5.6E-06	8.2E-03	6.9E-02	80.5	69.8	119.4	150.8	62.1	63.6	161.6	152.1	184.4	275.2	265.6	220.3	No	No	No	3-oxo-5-alpha-steroid 4-dehydrogenase 2, putative, expressed
Os.11982.1.S1_at	LOC_Os07g07470	3	1.3E-05	9.7E-08	2.2E-03	2.2E-04	5.8E-05	1.7E-01	2475	2432.9	2290.3	1798.5	1359.2	1671.1	2001.4	1756.6	2055.1	1638.4	1698.3	1900.7	No	Yes	Yes	3-methyl-2-oxobutanoate dehydrogenase, putative, expressed
Os.32503.1.S1_at	LOC_Os07g08430	7	4.3E-06	3.0E-08	4.3E-06	9.6E-05	2.8E-05	3.2E-03	3249.9	3434.8	3695.5	590.6	706	473.9	1570.7	2022.5	1727.9	724.5	796.6	730.8	Yes	Yes	Yes	indole-3-glycerol phosphate lyase, chloroplast precursor, putative, expressed
Os.32503.1.S1_x_at	LOC_Os07g08430	11	4.5E-07	2.1E-09	3.3E-07	1.9E-05	5.8E-06	6.3E-04	4566.8	4490.7	4084.7	941.1	968.8	800.3	1952	2402.3	1762.5	912.9	911.8	828.1	Yes	Yes	Yes	indole-3-glycerol phosphate lyase, chloroplast precursor, putative, expressed
Os.10872.1.S1_at	LOC_Os07g09420	5	1.0E-06	2.2E-09	1.1E-06	3.3E-05	5.9E-06	1.4E-03	2992.4	4013.2	3602.5	722.9	859.7	753.3	1967.5	3523.3	2376.3	1066.2	944.3	784.5	No	No	Yes	ATPase 2, putative, expressed
Os.48723.1.A1_at	LOC_Os07g09710	3	2.6E-06	2.0E-03	5.0E-03	6.6E-05	4.8E-02	2.7E-01	47	38.2	21.5	30.9	12.4	36.4	192.7	237.9	209.1	125.9	112.7	152.7	Yes	No	No	F-box domain containing protein, expressed
OsAffx.5270.1.S1_at	LOC_Os07g09710	4	2.4E-05	3.6E-04	8.1E-04	3.5E-04	1.5E-02	9.5E-02	68.6	48.8	44.4	56.6	67.8	59.6	250.8	228.1	217.9	153.8	202.6	164.7	Yes	No	No	F-box domain containing protein, expressed
Os.51134.1.S1_at	LOC_Os07g10190	10	4.1E-10	3.8E-06	7.7E-06	2.2E-07	6.5E-04	4.7E-03	223.4	160.2	171.7	314	201.4	187.6	856.4	1139.8	944.3	1706.2	1867.4	1851.3	Yes	No	No	anthocyanidin 3-O-glucosyltransferase, putative, expressed
Os.51134.2.S1_x_at	LOC_Os07g10220	3	5.5E-07	2.6E-05	1.0E-04	2.2E-05	2.4E-03	2.5E-02	122.8	106.8	65.4	175.8	119.4	114	227.7	224.2	216.3	454.2	447.7	421	Yes	No	No	anthocyanidin 3-O-glucosyltransferase, putative
OsAffx.28379.1.S1_at	LOC_Os07g10230	9	1.9E-09	4.5E-09	3.2E-08	5.4E-07	8.9E-06	1.5E-04	48	35.8	43.5	81.2	77	75	126.8	127.4	153.1	566.8	681.5	684.2	No	No	No	hydroquinone glucosyltransferase, putative, expressed
Os.18981.1.S1_at	LOC_Os07g18120	9	5.5E-07	3.1E-09	1.5E-07	2.2E-05	7.1E-06	3.9E-04	372.2	550.9	383.7	692	737.8	519.5	313.3	435.8	401.3	1638.3	1774.8	2052.5	Yes	Yes	Yes	abscisic-aldehyde oxidase, putative, expressed
Os.18981.1.S1_x_at	LOC_Os07g18120	11	1.2E-05	1.0E-07	2.2E-06	2.0E-04	6.0E-05	2.1E-03	610.1	877.5	612.8	910.6	956.6	833.8	491.4	746.5	671.1	1953.2	2621.7	2444.5	Yes	Yes	Yes	abscisic-aldehyde oxidase, putative, expressed
OsAffx.28501.1.S1_x_at	LOC_Os07g18120	8	1.7E-06	3.0E-09	9.7E-08	4.7E-05	6.9E-06	2.9E-04	607.7	599.4	538.2	882.4	688.8	888.5	358.4	489.9	427	1633	1766.1	1754.5	Yes	Yes	Yes	abscisic-aldehyde oxidase, putative, expressed
Os.9017.1.S1_x_at	LOC_Os07g23570	11	3.9E-09	3.0E-09	3.7E-09	8.3E-07	6.9E-06	3.6E-05	594.4	549.9	510.9	37.4	59.2	23.3	52	49.8	68.9	18.6	8.4	40.5	No	No	No	cytochrome P450 72A1, putative, expressed
OsAffx.28574.2.S1_at	LOC_Os07g25050	3	4.2E-07	5.2E-04	3.5E-04	1.8E-05	1.9E-02	5.6E-02	72.3	82.7	69.3	96.3	195.7	89	4.3	38.5	3.7	34.8	44	5.3	Yes	Yes	Yes	thionin-like peptide, putative, expressed
Os.51412.1.S1_at	LOC_Os07g25220	3	1.5E-05	3.3E-06	8.1E-04	2.4E-04	5.9E-04	9.5E-02	33.5	59.8	24.7	25.4	9.5	4.3	78.1	43.8	44.4	18.6	26.1	10.4	Yes	Yes	Yes	retrotransposon protein, putative, unclassified
OsAffx.5450.1.S1_at	LOC_Os07g26460	7	9.6E-05	1.3E-06	1.3E-04	1.0E-03	3.2E-04	2.9E-02	4.2	3.1	49.4	220.6	180.8	246.4	10.4	14.6	5.6	476.6	389.8	397	No	No	No	pleckstrin homology domain containing, family A, putative
Os.8118.1.S1_at	LOC_Os07g26630	9	1.4E-10	1.4E-06	1.1E-05	1.1E-07	3.3E-04	5.9E-03	4809.2	3995	5193.7	9567.4	8239.6	10215.1	1883.4	2255.1	2238.6	3741.2	2796	2981.7	Yes	Yes	Yes	aquaporin PIP2.4, putative, expressed
Os.32835.1.S1_x_at	LOC_Os07g27390	7	6.8E-13	1.2E-12	1.2E-12	9.0E-09	1.1E-07	2.5E-07	440.3	545.9	312.8	37.5	43.6	40.1	2	21	28.3	1.8	8.6	9.4	Yes	Yes	Yes	retrotransposon protein, putative, unclassified, expressed
Os.7338.1.S1_at	LOC_Os07g28850	6	2.6E-07	1.9E-05	1.8E-03	1.3E-05	2.0E-03	1.5E-01	1038	976.2	963.1	542.9	499.5	734.2	284.5	251.7	283.9	134.7	160.7	208.6	No	No	Yes	argonaute-like protein, putative, expressed
OsAffx.28651.1.S1_x_at	LOC_Os07g28850	6	4.2E-08	2.9E-05	3.0E-04	3.8E-06	2.6E-03	5.0E-02	764.9	550.7	553.4	382.2	354.2	369.6	145.4	133.3	155.4	88.4	115.4	60.7	No	No	Yes	argonaute-like protein, putative, expressed
Os.12688.1.S1_at	LOC_Os07g29330	3	3.9E-07	1.1E-04	1.0E-04	1.7E-05	6.5E-03	2.5E-02	127.2	188.5	128.5	84	116.9	96.5	84.8	126.7	44.4	55.8	99.9	62.1	No	Yes	No	serine/threonine-protein kinase CTR1, putative, expressed
Os.10075.1.S1_at	LOC_Os07g30090	11	1.1E-08	3.3E-10	3.3E-08	1.6E-06	2.0E-06	1.5E-04	62.3	56.6	49.1	356.9	275.4	219.9	81.5	57.4	46.2	1172.3	1081.3	1127.8	No	No	No	actin-depolymerizing factor 1, putative, expressed
Os.55785.1.S1_at	LOC_Os07g31610	7	1.5E-07	1.8E-07	3.2E-07	8.6E-06	8.8E-05	6.2E-04	176.8	150.8	118.5	1030	694.7	779.8	108.5	104.7	146.9	1118.9	1130.9	1611.8	No	No	Yes	peroxidase 7 precursor, putative, expressed
Os.12165.1.S1_at	LOC_Os07g32570	8	3.8E-05	3.0E-06	6.1E-04	4.9E-04	5.6E-04	7.9E-02	5763.9	5689.4	4558	2548.6	1296.1	1924.5	2154.1	3176.9	2531.6	1472.4	1275.6	1336.9	Yes	No	Yes	OsAPRL1 - Oryza sativa adenosine 5'-phosphosulfate reductase-like, expressed
Os.19519.1.S1_s_at	LOC_Os07g32570	5	5.8E-05	1.2E-06	3.6E-04	6.8E-04	2.9E-04	5.6E-02	4563.6	5612.2	4859.9	2008.8	1824.4	1833.7	2295.2	3640	3362	2144	1741	2173.2	Yes	No	Yes	OsAPRL1 - Oryza sativa adenosine 5'-phosphosulfate reductase-like, expressed
Os.5697.1.S1_at	LOC_Os07g33270	4	1.7E-06	4.9E-06	3.4E-05	4.7E-05	7.6E-04	1.2E-02	981.8	1502.9	1118.5	370.1	725.4	479	576.2	634.9	516.5	472	461.9	340.8	No	No	Yes	expressed protein
Os.32947.2.S1_at	LOC_Os07g33420	4	4.6E-06	1.2E-04	3.7E-04	1.0E-04	6.8E-03	5.8E-02	32.2	35.1	59.7	21.3	42.3	46.4	38.9	49.7	49.8	125.5	148.9	125.5	Yes	Yes	No	taxane 10-beta-hydroxylase, putative, expressed
Os.57049.1.S1_at	LOC_Os07g33690	9	1.2E-07	7.3E-05	5.7E-05	7.8E-06	4.9E-03	1.7E-02	397.2	444	400.7	138.9	209.8	217.5	3.1	7.6	36	7.8	16.9	16.3	Yes	Yes	Yes	NBS-LRR type disease resistance protein Hom-F, putative, expressed
OsAffx.28731.1.S1_s_at	LOC_Os07g33780	11	1.0E-06	2.5E-05	3.0E-05	3.3E-05	2.4E-03	1.1E-02	2959.2	2837.5	2480.6	1465.6	1021.1	1268.6	777.8	923.2	710.8	677	663.7	664.6	No	No	Yes	PDR-like ABC transporter, putative, expressed
OsAffx.30613.3.S1_at	LOC_Os07g34120	3	5.8E-06	3.4E-04	4.0E-04	1.2E-04	1.4E-02	6.0E-02	10.8	9.5	1.8	1.2	8.5	25.1	68.7	87.7	60.2	130.4	157.2	163.3	Yes	Yes	Yes	transposon protein, putative, unclassified
OsAffx.30613.3.S1_x_at	LOC_Os07g34120	5	1.2E-07	2.4E-04	2.6E-04	7.5E-06	1.1E-02	4.6E-02	15.5	16.5	1.8	1.1	5	5.3	133.5	92.4	141.6	284.9	180.2	289.2	Yes	Yes	Yes	transposon protein, putative, unclassified
OsAffx.16557.1.S1_at	LOC_Os07g34670	3	1.2E-04	4.3E-05	1.8E-04	1.2E-03	3.4E-03	3.6E-02	41.2	8.5	20.1	337.4	143.1	368.4	7.4	4.8	10	3.7	10.3	8.1	Yes	Yes	Yes	peroxidase 1 precursor, putative, expressed
Os.57292.1.S1_at	LOC_Os07g34690	3	8.9E-10	7.7E-10	8.4E-10	3.5E-07	3.2E-06	1.5E-05	55.2	69	4.4	3.5	15.1	19.5	1.7	22.5	5.6	2.6	3.4	2.9	Yes	No	Yes	expressed protein
Os.29830.1.S1_at	LOC_Os07g34920	5	1.2E-06	7.7E-08	1.1E-06	3.7E-05	4.9E-05	1.3E-03	18.8	40.7	34.6	114.6	90.8	121.6	43.7	47.5	40.3	262.8	238	312.2	No	No	Yes	aspartic proteinase nepenthesin-1 precursor, putative, expressed
Os.19326.1.S1_at	LOC_Os07g34940	4	4.3E-05	1.6E-05	4.6E-04	5.3E-04	1.7E-03	6.5E-02	814.4	961.8	751.3	459.1	557.6	329.2	516.7	686	479.6	454.7	506.1	417.6	No	No	No	aspartic proteinase nepenthesin-1 precursor, putative, expressed
OsAffx.28754.2.S1_at	LOC_Os07g34964	5	3.0E-05	5.0E-05	6.4E-05	4.1E-04	3.8E-03	1.9E-02	139.9	155.5	125.3	63.9	46.5	46.8	29.9	7	33.4	19.2	43.7	9.7	Yes	No	No	expressed protein
OsAffx.28760.2.S1_at	LOC_Os07g35180	3	2.4E-07	1.8E-03	1.9E-03	1.2E-05	4.5E-02	1.5E-01	2.7	8.3	12.5	18.5	5.2	6.2	419.4	471.2	559.6	303.7	241.9	231.5	Yes	Yes	No	hypothetical protein
Os.26323.1.S1_at	LOC_Os07g35290	3	8.8E-08	1.6E-05	2.2E-03	6.1E-06	1.8E-03	1.7E-01	272.4	501.9	374.9	262.7	304.3	274.9	799.6	1050	842.4	443.6	583.7	679.7	No	No	Yes	receptor-like serine-threonine protein kinase, putative, expressed
Os.7086.1.S1_at	LOC_Os07g35520	3	4.8E-08	7.4E-07	1.8E-05	4.1E-06	2.1E-04	8.2E-03	1261.6	1210.8	1546.7	2117.7	2157.4	2379.8	622.8	500.1	555.7	883.8	957.3	996	Yes	Yes	Yes	glucan endo-1,3-beta-glucosidase 3 precursor, putative, expressed
Os.7086.1.S1_s_at	LOC_Os07g35520	3	9.4E-07	7.6E-05	1.5E-03	3.1E-05	5.0E-03	1.4E-01	375.2	288.3	410.8	846.5	669.1	793	162.4	121.6	131.8	208.2	215.2	290.3	Yes	Yes	Yes	glucan endo-1,3-beta-glucosidase 3 precursor, putative, expressed
Os.49803.1.S1_at	LOC_Os07g35690	3	1.8E-06	9.0E-05	9.1E-04	4.9E-05	5.6E-03	1.0E-01	345.1	351.9	351.4	188.3	151.8	155.2	85	110.7	113.6	27	50.1	45.6	Yes	No	Yes	CRK6, putative, expressed
Os.54268.1.S1_at	LOC_Os07g35740	5	1.4E-08	3.9E-08	5.7E-08	1.9E-06	3.3E-05	2.1E-04	189.3	220.6	171.6	108.8	144.1	97.1	20.6	29.8	39.9	47.7	42	53.9	Yes	Yes	Yes	protein kinase, putative, expressed
OsAffx.5542.1.S1_at	LOC_Os07g35860	6	2.9E-04	5.8E-07	1.3E-04	2.4E-03	1.8E-04	3.0E-02	156.2	164.4	56.3	751.3	476.9	667.9	10	11.4	15.2	1246.5	974.4	1470.6	Yes	No	No	uclacyanin-2 precursor, putative, expressed
OsAffx.5542.1.S1_s_at	LOC_Os07g35860	8	5.6E-13	1.1E-11	1.3E-11	8.0E-09	4.5E-07	1.5E-06	322.8	433	523.9	2844.8	2227.8	2600.5	3.8	3.9	21	152.2	130.7	166.8	Yes	No	No	uclacyanin-2 precursor, putative, expressed
OsAffx.5550.1.S1_at	LOC_Os07g36750	3	2.1E-06	8.5E-07	1.4E-05	5.5E-05	2.4E-04	7.0E-03	117.8	107.1	83.7	573.3	519.8	658.4	33.5	68.9	28.3	479.5	360.7	314.5	No	No	No	CSLF3 - cellulose synthase-like family F; beta1,3;1,4 glucan synthase, expressed
Os.52135.1.S1_at	LOC_Os07g37130	3	7.0E-08	8.1E-04	1.8E-03	5.3E-06	2.6E-02	1.5E-01	248.3	113.2	143.4	272.2	220.5	188.9	530.8	601.6	705.5	836.7	791.7	1127.2	No	No	No	nucleoside transporter, putative, expressed
Os.53714.1.S1_at	LOC_Os07g38440	3	1.6E-04	1.2E-04	6.7E-04	1.5E-03	6.9E-03	8.4E-02	165.1	137.3	144.6	217	194.4	229.3	138	169.6	131.3	398.7	332.6	359.4	No	Yes	Yes	regulatory protein, DeoR, putative, expressed
Os.54822.1.S1_at	LOC_Os07g38890	4	1.2E-07	1.3E-04	1.2E-04	7.5E-06	7.4E-03	2.8E-02	13.9	6.5	4.4	5.3	41.2	10	145.5	121.2	106	315.2	335	242.3	Yes	Yes	Yes	indole-3-acetic acid-amido synthetase GH3.9, putative, expressed
OsAffx.16631.1.S1_at	LOC_Os07g39210	3	2.8E-08	2.3E-04	1.8E-04	2.9E-06	1.1E-02	3.7E-02	53	44.8	52.3	31.1	25.3	19.6	394.9	342	350.5	561.3	516.7	680.4	Yes	No	No	expressed protein
OsAffx.28832.1.S1_at	LOC_Os07g39940	4	2.8E-05	2.8E-06	2.7E-04	3.9E-04	5.3E-04	4.7E-02	60.8	52.7	108.9	160.2	79.4	108.1	58.8	46.3	47.5	197	185.7	289.2	No	No	No	DNA binding protein, putative
Os.11798.1.S1_at	LOC_Os07g40290	5	3.9E-05	2.6E-04	6.6E-04	5.0E-04	1.2E-02	8.3E-02	908.2	952.8	853.8	460.7	677.6	446.8	473.7	452.9	393.4	530	447.7	407.1	Yes	Yes	Yes	indole-3-acetic acid-amido synthetase GH3.8, putative, expressed
Os.9527.1.S1_at	LOC_Os07g41460	11	3.6E-07	1.5E-09	5.0E-07	1.6E-05	4.5E-06	8.2E-04	3181.8	3819.5	3468	222.9	333.5	409.1	1347.6	1690.9	1291.6	253.5	264.2	215.8	No	No	No	flavonol 4-sulfotransferase, putative, expressed
OsAffx.28867.2.S1_at	LOC_Os07g42070	5	4.5E-04	3.5E-04	2.2E-04	3.3E-03	1.4E-02	4.2E-02	80.2	72.8	109.9	372.9	368.8	529.8	113.8	79.2	102.4	115.8	102.1	215.6	No	No	No	protein binding protein, putative, expressed
Os.32823.1.S1_at	LOC_Os07g42834	3	1.0E-05	4.5E-05	5.8E-05	1.8E-04	3.5E-03	1.7E-02	31.2	14.8	21.4	2.3	12.8	17.4	1.4	0.7	6.4	5.2	6.4	0.9	No	No	Yes	retrotransposon protein, putative, unclassified
Os.6901.1.S1_at	LOC_Os07g42834	9	1.1E-08	5.3E-09	1.2E-08	1.6E-06	9.8E-06	7.8E-05	451.4	491.5	437.2	26.6	55	50.6	94.6	55.3	55.3	32.2	40	32	No	No	Yes	retrotransposon protein, putative, unclassified
Os.34358.1.S1_at	LOC_Os07g42924	3	9.4E-06	9.9E-05	2.1E-04	1.7E-04	6.0E-03	4.0E-02	713.1	722.6	655.3	1053.3	487.5	714	435.9	470	473.8	827.6	761.8	881.6	No	No	No	alcohol dehydrogenase 3, putative, expressed
Os.9982.1.S1_at	LOC_Os07g43240	11	1.8E-10	5.6E-10	7.0E-10	1.3E-07	2.7E-06	1.5E-05	998.4	971.1	1031	114.8	149.4	143.1	79.7	11.9	50.5	21.6	65.8	32.8	No	Yes	Yes	SKP1-like protein 1B, putative, expressed
Os.10223.1.S1_at	LOC_Os07g43604	8	1.5E-08	5.5E-06	5.3E-06	2.0E-06	8.2E-04	3.6E-03	560.2	658.2	582.4	218.8	251.4	244.8	20.9	54.5	31.9	32.9	10.9	1	No	No	No	expressed protein
Os.46050.1.A1_x_at	LOC_Os07g43604	4	2.3E-06	4.9E-06	4.6E-06	5.9E-05	7.6E-04	3.3E-03	123.9	87.2	102.9	24.2	56.2	35.7	4.1	44.2	31.1	12.3	3	23.3	No	No	No	expressed protein
Os.34315.1.S1_at	LOC_Os07g43970	4	7.4E-07	2.4E-07	5.4E-06	2.6E-05	1.0E-04	3.6E-03	77.3	80	90.5	180.2	215	176.8	119.3	107.1	142	394.1	386.8	614.7	No	No	No	calmodulin binding protein, putative, expressed
Os.9067.1.S1_at	LOC_Os07g44140	10	1.3E-07	8.3E-10	2.4E-06	7.8E-06	3.4E-06	2.2E-03	6159.6	5734	6348.6	1624.1	1885.8	1323	3250.8	4921.3	4768.6	1599.1	998.8	762.3	No	No	Yes	cytochrome P450 72A1, putative, expressed
Os.15471.1.S1_at	LOC_Os07g44410	6	1.3E-05	5.8E-09	1.2E-05	2.2E-04	1.0E-05	6.4E-03	14579.1	15739.2	15053.8	725.3	824.2	577.2	9829.7	10689.1	10356.9	1010.7	1042.7	869	No	No	No	WD40-like Beta Propeller Repeat family protein, expressed
Os.13972.1.S1_at	LOC_Os07g44430	7	9.8E-07	3.1E-09	6.6E-07	3.2E-05	7.1E-06	9.7E-04	1415.1	1073.2	1026.4	50.2	60.3	35	262	204.6	312.4	163.6	69.8	36.6	No	Yes	Yes	peroxiredoxin, putative, expressed

Probe Set ID	Locus ID	probe #	ANOVA p-value			FDR q-value			Nipponbare MV			Nipponbare CK			93-11 MV			93-11 CK			Indel (Indica vs Japonica)			Annotation
			C	T	C×T	C	T	C×T	Repeat 1	Repeat 2	Repeat 3	Repeat 1	Repeat 2	Repeat 3	Repeat 1	Repeat 2	Repeat 3	Repeat 1	Repeat 2	Repeat 3	upstream	gene	downstream	
Os.11218.1.S1_at	LOC_Os07g44440	4	3.1E-04	1.2E-12	6.5E-05	2.4E-03	1.1E-07	1.9E-02	22408.7	25034.2	21183.4	684.6	2817.9	1026.9	20862	21694.9	21711.8	2089	2141.2	1343.9	Yes	No	No	peroxiredoxin, putative, expressed
OsAffx.27508.1.S1_at	LOC_Os07g44480	8	1.4E-07	2.6E-10	1.7E-07	8.5E-06	1.8E-06	4.1E-04	152.6	120.6	66.7	521.8	609.6	590.8	110.6	132.4	151.6	1770.1	1866	1813.1	No	No	No	peroxidase, putative, expressed
Os.29933.1.S1_at	LOC_Os07g44910	3	1.5E-07	3.0E-06	1.0E-04	8.8E-06	5.5E-04	2.5E-02	374.9	561.3	402	713.8	512.3	593.6	173.6	171.9	156.6	332.4	369.4	437.7	Yes	Yes	Yes	gibberellin receptor GID1L2, putative, expressed
Os.34356.1.S1_at	LOC_Os07g45260	3	1.3E-04	7.8E-03	1.7E-04	1.2E-03	1.2E-01	3.5E-02	401	472.5	338.3	245.8	188.4	174	146.9	107.9	91.3	228.6	219.6	144.6	No	No	No	transferase, transferring glycosyl groups, putative, expressed
OsAffx.30966.4.S1_at	LOC_Os07g46060	5	3.0E-04	2.9E-04	2.8E-04	2.4E-03	1.3E-02	4.9E-02	38.9	19.5	19	38.5	2	7.9	2.8	1	19.8	209.8	163.1	115.6	Yes	No	Yes	expressed protein
Os.26799.1.A1_at	LOC_Os07g47590	5	7.6E-04	3.5E-05	1.2E-03	4.9E-03	3.0E-03	1.2E-01	2224.2	1716.2	2241.5	892.7	771.5	962.5	1367.2	993.9	1255.6	993.4	768	961.7	No	No	No	expressed protein
Os.11556.1.S1_at	LOC_Os07g48030	3	1.2E-04	1.3E-08	2.9E-04	1.2E-03	1.6E-05	4.9E-02	869.5	1024.4	953.4	4722.7	3864.7	5628.8	1329.1	1668.5	1595.2	3187	3460	3094	No	No	No	peroxidase 2 precursor, putative, expressed
Os.49354.1.S1_at	LOC_Os07g48730	5	4.2E-06	5.3E-08	6.9E-06	9.4E-05	3.9E-05	4.4E-03	498.3	540.4	523.4	75.1	108.2	101.1	106.4	219.2	183	92.6	60	46.8	No	No	No	serine/threonine-protein kinase NAK, putative, expressed
Os.32634.1.S1_at	LOC_Os07g48870	3	1.0E-05	2.8E-07	3.3E-04	1.8E-04	1.2E-04	5.3E-02	641.9	709.1	649.1	200.2	263.4	190.4	274.1	266.6	271	149.2	113.6	163.9	No	No	No	typical P-type R2R3 Myb protein, putative, expressed
Os.34729.1.S1_at	LOC_Os07g49120	4	1.1E-04	8.7E-07	1.6E-04	1.1E-03	2.4E-04	3.3E-02	151.3	145.7	186.8	266.1	223.7	270.9	148.2	186.1	158.8	434.2	333.4	351.9	Yes	No	Yes	sex determination protein tasselseed-2, putative, expressed
Os.50798.1.S1_at	LOC_Os08g01480	11	3.9E-09	2.0E-09	1.9E-08	8.3E-07	5.5E-06	1.0E-04	1662.1	1579.2	1682.2	253.2	163.3	209	194.6	243	208.5	33.2	29.6	64.9	No	No	No	cytochrome P450 71C4, putative, expressed
Os.11284.1.S1_at	LOC_Os08g01760	3	6.1E-05	2.9E-05	1.2E-05	7.0E-04	2.6E-03	6.4E-03	6454.7	5932.9	7234	3661.6	2953.6	3672.6	4344.2	4086	4141.2	4742	3843.8	4196	No	No	No	S-glutathione dehydrogenase/class III alcohol dehydrogenase, putative, expressed
Os.19547.1.S1_at	LOC_Os08g02030	8	1.3E-11	5.8E-11	1.3E-10	3.4E-08	9.8E-07	5.5E-06	3579.1	3108.6	2849.7	794.2	543.6	702.9	127.9	265.1	329	63.6	97.5	35	No	No	No	expressed protein
Os.19547.1.S1_x_at	LOC_Os08g02030	10	1.5E-12	6.5E-12	1.6E-11	1.3E-08	3.9E-07	1.5E-06	3447.3	2734.7	3124.6	729.7	778.6	645.2	254.1	311.9	335.7	97	147.6	86.6	No	No	No	expressed protein
Os.10579.1.S1_at	LOC_Os08g02700	3	2.2E-06	3.0E-08	8.1E-05	5.8E-05	2.8E-05	2.2E-02	5519.2	7635.2	6777.3	2337	2539.2	2617.4	3434.4	4976.2	4930	1640.7	2115.5	2229.7	No	No	No	fructose-bisphosphate aldolase cytoplasmic isozyme, putative, expressed
Os.53070.1.S1_at	LOC_Os08g03170	7	4.3E-07	2.2E-05	6.5E-05	1.8E-05	2.2E-03	1.9E-02	3.4	3.1	1.4	4.9	3.5	20.5	287.1	347	391.6	133	160.1	184.2	No	No	No	protein kinase, putative, expressed
Os.51918.1.S1_at	LOC_Os08g04130	6	4.1E-07	1.1E-04	3.7E-04	1.8E-05	6.6E-03	5.8E-02	999.1	1145.4	1050	474.7	614.4	622.1	335.8	315.2	389.6	301.1	319.7	389.3	Yes	No	No	copine-6, putative, expressed
Os.13900.1.S1_at	LOC_Os08g04180	11	8.3E-08	4.1E-08	3.3E-07	5.9E-06	3.3E-05	6.4E-04	7487.3	5546.4	7497.1	2874.8	1962.7	2376.3	3216.4	2670.4	3038.8	2195.8	2180.1	2477	Yes	No	Yes	tryptophan synthase beta chain 1, putative, expressed
Os.57472.1.S1_x_at	LOC_Os08g04180	11	1.0E-06	3.1E-07	2.6E-06	3.3E-05	1.2E-04	2.3E-03	4592.9	3895.6	4475.5	1703.6	1191.2	1205.6	1731.2	1473	1872.4	1307.9	1126.6	1155.6	Yes	No	Yes	tryptophan synthase beta chain 1, putative, expressed
Os.23207.1.S1_at	LOC_Os08g04340	9	2.2E-09	6.8E-08	5.8E-08	5.9E-07	4.6E-05	2.1E-04	587.3	492.9	592.2	124.9	168.3	175	4.6	18.6	4.4	4.6	4.4	4.9	No	No	No	blue copper protein precursor, putative, expressed
Os.27876.1.S1_x_at	LOC_Os08g04540	3	1.1E-04	5.5E-07	1.2E-03	1.1E-03	1.8E-04	1.2E-01	491.7	346.7	355.9	95	90.8	47.4	134.4	236.5	189	45.3	9.8	67.3	No	No	No	aromatic-L-amino-acid decarboxylase, putative, expressed
OsAffx.5702.1.S1_s_at	LOC_Os08g04560	4	2.8E-04	5.8E-05	1.6E-03	2.3E-03	4.2E-03	1.4E-01	915.7	1005.5	1214.2	350	398.8	422.1	526.8	581	450.7	533.6	400.3	360.6	No	No	Yes	aromatic-L-amino-acid decarboxylase, putative, expressed
Os.10682.1.S1_at	LOC_Os08g05960	7	1.1E-04	1.5E-10	1.7E-04	1.1E-03	1.5E-06	3.5E-02	5370.6	7016.7	6356.8	170	383.1	187.9	3893.9	5481.5	4399.9	178	224.8	116.4	No	No	No	expressed protein
Os.36496.1.S1_at	LOC_Os08g05980	6	6.8E-05	2.0E-10	5.2E-05	7.6E-04	1.7E-06	1.6E-02	6769.5	6069.4	7496.4	189.5	202	130.9	5995.7	5168	4817.9	224.5	183.4	105.7	No	No	No	expressed protein
Os.26820.1.A1_at	LOC_Os08g07080	4	5.9E-08	7.3E-07	7.9E-07	4.7E-06	2.1E-04	1.1E-03	35.2	40.2	15.8	16.4	47.2	32	118.1	171.6	127.2	15.4	76.8	60.6	Yes	No	No	terpene synthase 10, putative, expressed
Os.26820.1.A1_s_at	LOC_Os08g07080	5	4.1E-04	1.1E-04	2.3E-05	3.0E-03	6.4E-03	9.7E-03	272	103.6	103.9	147.7	175.6	103.1	332.2	519.1	332	46.9	147.1	124.6	Yes	No	No	terpene synthase 10, putative, expressed
Os.51406.1.S1_at	LOC_Os08g07690	3	1.0E-03	7.1E-06	2.3E-03	6.1E-03	9.9E-04	1.7E-01	543.6	558.1	533.6	740.1	605.6	731.6	92.3	137.9	205.9	608.1	704.2	661	No	No	No	expressed protein
OsAffx.16888.1.S1_at	LOC_Os08g07990	5	5.6E-05	3.0E-08	6.5E-04	6.6E-04	2.8E-05	8.2E-02	1360.1	1568.9	1150.1	230.8	201	298.5	584	862.8	871.4	78.4	82.1	81.3	No	No	No	(11674.m00736)
Os.39994.1.S1_at	LOC_Os08g12660	6	6.9E-04	4.9E-06	7.9E-04	4.6E-03	7.7E-04	9.3E-02	336	434.9	432.3	52.9	14.5	43.6	126.6	203	218.6	30.2	25.9	31.9	No	No	No	retrotransposon protein, putative, Ty3-gypsy subclass
Os.49534.1.S1_at	LOC_Os08g17160	5	1.3E-12	2.8E-06	3.1E-06	1.2E-08	5.3E-04	2.6E-03	1466.9	1741.1	1594.7	1990.3	2415.1	1743.4	17.1	25.5	25.3	5	46.8	11.7	No	Yes	No	early nodulin-like protein 1 precursor, putative, expressed
Os.49534.1.S1_x_at	LOC_Os08g17160	5	3.5E-13	3.8E-07	4.0E-07	6.5E-09	1.4E-04	7.1E-04	2155.5	2181.5	1862	2822	2870	2456.7	37.1	45.1	37.3	93.2	49	37.4	No	Yes	No	early nodulin-like protein 1 precursor, putative, expressed
Os.54153.1.S1_at	LOC_Os08g24310	10	6.3E-09	6.8E-07	4.1E-07	1.1E-06	2.0E-04	7.2E-04	840.2	905.1	874.1	178.5	247.6	281.8	43.3	43.3	58	10.2	6	4.3	No	No	No	receptor-like protein kinase precursor, putative, expressed
Os.27296.1.A1_at	LOC_Os08g24630	7	1.6E-07	4.1E-05	4.2E-05	9.3E-06	3.3E-03	1.4E-02	587.9	764.1	537.9	280.5	275.3	292.4	100.5	154.7	120.5	112	146.1	132.4	No	No	No	protein kinase, putative, expressed
OsAffx.29332.1.S1_at	LOC_Os08g24670	4	8.7E-07	6.0E-05	4.1E-05	3.0E-05	4.3E-03	1.4E-02	112.2	125.1	75.9	63.6	4.8	48.9	2.9	5.6	7.8	2.9	3.9	8.4	No	No	Yes	lectin-related protein precursor, putative, expressed
Os.51079.1.S1_at	LOC_Os08g24750	5	2.5E-05	4.5E-06	1.9E-05	3.6E-04	7.2E-04	8.4E-03	435.4	428.8	389	130.6	119.4	127.5	164.8	247.3	207	165.2	84.3	90.2	No	No	No	fucosyltransferase 7, putative, expressed
Os.50824.1.S1_at	LOC_Os08g26010	9	6.9E-08	5.2E-07	6.0E-07	5.2E-06	1.7E-04	9.3E-04	803.5	1095.8	785.1	74.4	153.7	106.6	12.8	31.7	25.7	10	39.2	1.9	Yes	Yes	Yes	transposon protein, putative, CACTA, En/Spm sub-class
Os.50824.1.S1_x_at	LOC_Os08g26010	9	8.3E-10	6.9E-09	1.5E-08	3.4E-07	1.1E-05	8.7E-05	1014.8	1320	1241.5	115.7	162	298	161.5	158.2	103.4	13.9	57.4	50.9	Yes	Yes	Yes	transposon protein, putative, CACTA, En/Spm sub-class
Os.51235.1.S1_at	LOC_Os08g26120	10	5.7E-07	8.6E-06	1.6E-05	2.2E-05	1.1E-03	7.8E-03	895.6	1019.7	1024	212.9	333.2	422.1	104	143.4	112.1	14	26.6	21.5	Yes	No	No	expressed protein
Os.9543.1.S1_at	LOC_Os08g26350	5	6.0E-06	6.1E-09	5.2E-04	1.2E-04	1.0E-05	7.1E-02	1116.1	1273.6	1116.5	260	365.6	228.2	562.6	748.6	636	174.6	164.3	184.4	Yes	No	No	expressed protein
Os.15737.1.S1_x_at	LOC_Os08g26710	6	3.5E-07	1.5E-04	2.1E-04	1.6E-05	8.0E-03	4.0E-02	647.1	906.2	639.4	411	415.6	411.7	4.4	89.7	6.8	10.8	5.6	4.2	Yes	Yes	Yes	expressed protein
OsAffx.5948.1.S1_at	LOC_Os08g27170	11	3.6E-10	1.1E-10	2.1E-09	2.1E-07	1.3E-06	2.4E-05	3551.9	3665.8	3530.7	274.5	371.7	549.3	698.1	703	526.8	53.8	110.9	39	No	No	Yes	calmodulin binding protein, putative
Os.17020.1.S1_x_at	LOC_Os08g29570	4	2.7E-10	1.0E-09	8.0E-07	1.7E-07	3.6E-06	1.1E-03	535	670.8	458.1	177.2	229.5	222.9	202.6	338.1	352.1	131	162.1	136.2	No	No	No	PDR-type ABC transporter 1, putative, expressed
Os.11326.1.S1_at	LOC_Os08g30510	3	8.6E-10	3.0E-05	8.3E-05	3.4E-07	2.7E-03	2.2E-02	1040.7	1191.8	1378.6	715.4	789.5	932	48.9	107.2	110.8	81.8	82.8	67.9	Yes	No	Yes	expressed protein
Os.6288.1.S1_at	LOC_Os08g31850	11	9.2E-10	3.9E-09	8.3E-09	3.5E-07	8.2E-06	6.2E-05	4332.5	5103.1	6015	619.4	600.5	1158.6	25.4	125.5	29.8	22	39.3	14.4	Yes	Yes	Yes	expressed protein
Os.30528.1.S1_at	LOC_Os08g31860	10	2.1E-11	2.1E-11	3.0E-11	4.5E-08	5.8E-07	2.0E-06	525.5	472.1	508.5	50.9	12.2	51.2	8.3	35.2	12.3	0.9	5.2	12.3	Yes	Yes	Yes	expressed protein
OsAffx.6009.1.S1_at	LOC_Os08g32840	7	4.9E-08	1.3E-05	2.1E-05	4.2E-06	1.5E-03	9.3E-03	461.8	528.2	507.5	245.3	172.7	248.2	28.5	54.6	58.4	37.8	43.8	15.5	No	No	Yes	carbonic anhydrase precursor, putative, expressed
Os.6139.1.S1_at	LOC_Os08g34580	8	1.6E-05	5.4E-07	3.4E-05	2.5E-04	1.7E-04	1.2E-02	449.9	356.4	412.3	131.7	101.8	58.3	155.5	175.6	118.4	64	63.5	75.3	No	No	Yes	trehalose-6-phosphate synthase, putative, expressed
OsAffx.29514.1.S1_at	LOC_Os08g34720	7	1.9E-08	4.0E-05	1.0E-04	2.3E-06	3.2E-03	2.6E-02	8252.4	11083.4	9665	3830.2	5398.6	5328.3	1296.3	2058.4	1849.4	1314.9	1611.8	1479	No	No	No	D-3-phosphoglycerate dehydrogenase, chloroplast precursor, putative, expressed
Os.55850.1.S1_at	LOC_Os08g34800	5	4.9E-04	5.4E-04	3.5E-04	3.5E-03	1.9E-02	5.5E-02	738.7	856.9	853	248.2	339.6	431.2	286.1	448.6	343.1	450.7	330.2	411.1	Yes	No	Yes	expressed protein
Os.8851.1.S1_at	LOC_Os08g35630	8	2.0E-06	2.9E-07	5.6E-05	5.4E-05	1.2E-04	1.7E-02	1999.1	1719.3	1866.4	987.3	722.5	805.8	757.6	932.4	803.3	481.6	498.8	504.1	No	No	No	MTD1, putative, expressed
Os.4717.1.S1_at	LOC_Os08g36480	4	1.3E-06	1.0E-04	9.9E-04	3.9E-05	6.2E-03	1.1E-01	345.9	497.9	322.5	266.1	335.5	266.6	1547.9	1974.6	1352.2	775.2	996.1	770.3	No	No	No	nitrate reductase 1, putative, expressed
Os.10908.1.S1_a_at	LOC_Os08g36910	3	1.0E-06	1.6E-06	1.8E-06	3.4E-05	3.6E-04	1.8E-03	192.7	144.7	128.6	33.1	62.8	38.1	12.9	30	47.6	1	27.8	12.7	No	No	Yes	alpha-amylase isozyme 3D precursor, putative, expressed
Os.12452.1.S1_s_at	LOC_Os08g36910	11	4.8E-07	5.3E-07	6.0E-07	2.0E-05	1.7E-04	9.3E-04	416.3	327.2	498.9	23.3	52.1	23.8	48.8	32.3	38.2	5.4	22.5	20.6	No	No	Yes	alpha-amylase isozyme 3D precursor, putative, expressed
Os.23397.1.S1_at	LOC_Os08g37874	11	4.3E-07	5.8E-09	5.6E-07	1.8E-05	1.0E-05	8.8E-04	2680.6	3030.2	2748	585.9	617.6	559.7	1069.3	1210	1262.9	546.6	510.4	500.5	No	Yes	Yes	oxidoreductase, putative, expressed
Os.7659.1.S1_s_at	LOC_Os08g38560	3	3.9E-04	6.3E-07	4.2E-04	2.9E-03	1.9E-04	6.2E-02	393.1	626.7	579	1064.8	909	1279.4	486.1	645.2	510	1903.2	1481.5	1261.9	No	No	No	receptor-like protein kinase precursor, putative, expressed
Os.10254.1.S1_at	LOC_Os08g38600	9	3.6E-06	1.3E-07	4.3E-06	8.3E-05	6.9E-05	3.2E-03	808.6	915.5	808.3	150.1	128.2	138.9	248.4	316.9	315	144.2	161.5	179.2	No	No	No	copine-6, putative, expressed
Os.54776.1.S1_at	LOC_Os08g38920	4	1.3E-09	5.3E-05	1.7E-05	4.4E-07	3.9E-03	8.0E-03	122.2	68.9	61.3	58.4	82.4	29	469	497.2	468.9	570.4	855.7	715.6	No	No	No	caffeoyl-CoA O-methyltransferase 2, putative, expressed
Os.14951.1.S1_at	LOC_Os08g39730	10	1.3E-07	4.0E-08	1.4E-07	7.9E-06	3.3E-05	3.7E-04	1135	1288.7	1352.5	71.8	55.8	78.2	149.6	137.4	116.2	45.8	42.6	78.1	No	No	No	cytochrome P450 76C2, putative, expressed
Os.4416.1.S1_at	LOC_Os08g39840	8	6.8E-06	5.5E-06	8.6E-05	1.3E-04	8.2E-04	2.2E-02	378.7	596.3	395.6	53.2	104.4	77.4	84.6	124.5	154.5	24.4	36.6	15.5	No	Yes	No	lipoxygenase 7, chloroplast precursor, putative, expressed
Os.13008.1.S1_at	LOC_Os08g39850	11	1.3E-06	1.1E-07	3.0E-06	3.8E-05	6.3E-05	2.5E-03	1438.9	1473.1	1404.8	120.3	149.5	167.1	180.2	318	286.6	95.7	76.8	108.6	No	No	No	lipoxygenase 8, chloroplast precursor, putative, expressed
Os.4867.1.S1_at	LOC_Os08g40680	6	1.3E-05	6.7E-06	2.0E-04	2.2E-04	9.5E-04	3.9E-02	286.1	345	303.2	139.7	165.6	132.6	129.2	138.5	132.2	76.2	44	63.7	Yes	No	No	xylanase inhibitor protein 2 precursor, putative, expressed
Os.10394.2.S1_at	LOC_Os08g42030	10	1.7E-07	1.0E-09	3.5E-07	9.7E-06	3.6E-06	6.5E-04	256	256.5	212.2	837.1	694	811.5	305.1	243	235.4	1736.4	1572.5	1419.5	No	No	No	peroxidase 73 precursor, putative, expressed
Os.11632.1.S1_at	LOC_Os08g42590	9	1.4E-05	5.7E-09	1.2E-05	2.3E-04	1.0E-05	6.2E-03	10043.2	10143.6	10754.2	2393.8	2368.2	2335.1	5657	7546.5	5842.2	2380.3	2608.2	2189.1	No	Yes	No	mtN19-like protein, putative, expressed
Os.9749.1.S1_at	LOC_Os08g44340	4	3.5E-07	1.5E-09	2.4E-05	1.6E-05	4.5E-06	1.0E-02	14796.6	15673.6	15243.6	7948.4	6720.7	8881.3	13019.3	11866.5	13139.7	7071.2	5594.9	6883.8	No	No	No	monodehydroascorbate reductase, putative, expressed
Os.37533.1.S1_at	LOC_Os08g44390	9	4.2E-05	1.7E-07	8.3E-06	5.3E-04	8.3E-05	4.9E-03	4390.9	4058.2	5208	1588	1334.7	1917.8	2696.6	2458.2	2630.5	1878.3	1495.5	2149.9	No	No	No	grancalcin, putative, expressed
Os.51029.1.S1_at	LOC_Os09g02770	6	8.8E-07	1.5E-04	1.4E-04	3.0E-05	7.9E-03	3.1E-02	316.2	330.6	272.6	162.3	172.5	159.6	43.1	52.7	30.2	46.7	10.4	22.6	Yes	No	No	PE-PGRS family protein, putative, expressed
Os.52750.1.S1_x_at	LOC_Os09g03190	8	5.5E-10	6.1E-10	8.8E-10	2.6E-07	2.9E-06	1.5E-05	1211.2	1043.1	1113.4	172.8	202.1	223.1	118.2	143.9	146.4	139.5	76.8	89.7	Yes	Yes	Yes	expressed protein
Os.50903.1.S1_at	LOC_Os09g08130	8	1.7E-07	3.0E-08	4.3E-07	9.6E-06	2.8E-05	7.4E-04	1327.5	1450.1	1476.9	75.1	108.5	60.3	139.7	187.6	201	74.2	30.5	48.3	No	No	No	indole-3-glycerol phosphate synthase, chloroplast precursor, putative, expressed
Os.50903.2.S1_x_at	LOC_Os09g08130	8	9.3E-09	3.1E-09	1.4E-08	1.4E-06	7.1E-06	8.3E-05	1166.5	931.4	981.7	160.8	87.8	75.4	185.6	223.9	220.9	116.1	58.4	43.7	No	No	No	indole-3-glycerol phosphate synthase, chloroplast precursor, putative, expressed
Os.39995.1.S1_x_at	LOC_Os09g09650	3	3.8E-05	2.3E-05	2.8E-05	4.9E-04	2.2E-03	1.1E-02	81	72.3	88.3	9.4	2.2	14.6	9.2	1.6	2.5	0.6	6.9	20.3	Yes	Yes	No	retrotransposon protein, putative, Ty3-gypsy subclass, expressed
OsAffx.6250.1.S1_at	LOC_Os09g12400	8	1.4E-09	3.9E-08	3.7E-08	4.5E-07	3.2E-05	1.6E-04	8	3.8	16.4	5.3	9.4	20.6	84.2	88.9	97.7	554.6	313	500.8	No	No	Yes	hypothetical protein
OsAffx.6251.1.S1_at	LOC_Os09g12420	3	4.1E-06	5.4E-05	4.2E-05	9.1E-05	4.0E-03	1.4E-02	1.7	4.8	17	39.4	2	2.4	41	16.6	49.2	121.9	103.2	106.1	Yes	Yes	No	transposon protein, putative, Mutator sub-class, expressed
OsAffx.17663.1.S1_at	LOC_Os09g12430	3	1.0E-05	2.1E-05	3.6E-04	1.8E-04	2.1E-03	5.6E-02	5.4	2.7	10.2	5.4	37.1	5.9	16.1	8.1	41.8	71.3	11.1	107	No	No	Yes	hypothetical protein
OsAffx.29827.1.S1_at	LOC_Os09g13250	4	4.2E-05	1.1E-05	5.2E-05	5.3E-04	1.3E-03	1.6E-02	1.9	3.3	1.5	6.3	4.3	6.7	2.5	2.1	2.4	50.4	26.3	10.1	No	No	Yes	hypothetical protein
Os.56191.1.S1_at	LOC_Os09g13890	10	1.5E-05	2.4E-06	1.5E-04	2.4E-04	4.8E-04	3.2E-02	915.3	1054	1114.6	243.1	285.4	324.7	281.8	363.3	266.1	102.5	98.3	87.7	No	Yes	No	calmodulin binding protein, putative, expressed
Os.5768.1.S1_at	LOC_Os09g15320	10	3.5E-09	7.5E-08	4.2E-06	7.8E-07	4.8E-05	3.2E-03	1293	1355.6	1416.2	536.9	557.4	481.2	263.5	306.8	334.8	166.8	147.3	166.6	No	No	No	NEDD8-conjugating enzyme Ubc12-like, putative, expressed
Os.53227.1.S1_at	LOC_Os09g15790	5	1.8E-05	1.7E-07	1.1E-04	2.8E-04	8.4E-05	2.6E-02	103.5	159.3	143.2	223.8	271.8	218.3	133	113	92.8	456	465	393.7	No	No	No	ras-related protein Rab11B, putative, expressed
OsAffx.29871.3.S1_at	LOC_Os09g16290	3	1.1E-04	5.7E-04	3.6E-04	1.1E-03	2.0E-02	5.6E-02	4.1	3.2	6.9	10.7	2.4	12.7	29.3	23.5	30.5	61.8	58.3	82.4	No	No	No	ABC transporter family protein
OsAffx.29871.1.S1_x_at	LOC_Os09g16330	11	2.8E-08	1.0E-08	1.8E-07	2.9E-06	1.4E-05	4.2E-04	7438.3	6949.5	5964.6	1739.7	1353	1908	2048	2368.8	2321	1040.5	1211.4	1299.4	No	No	No	PDR5-like ABC transporter, putative, expressed
Os.57392.1.S1_at	LOC_Os09g17190	4	2.2E-06	4.1E-08	3.7E-06	5.9E-05	3.3E-05	2.9E-03	351.4	339.5	431.9	30.6	39.1	9.7	178.3	273.4	212.9	59.3	34	46.3	Yes	No	Yes	F-box domain containing protein, expressed
Os.54347.1.S1_at	LOC_Os09g17540	9	9.8E-07	1.7E-08	3.5E-07	3.2E-05	2.0E-05	6.6E-04	627.8	568.4	673.5	14.7	2.2	30.2	130	107.1	175.3	5.5	21.9	15.3	Yes	No	No	F-box domain containing protein, expressed
OsAffx.17771.1.S1_at	LOC_Os09g18390	6	3.5E-06	7.9E-07	1.9E-05	8.2E-05	2.3E-04	8.7E-03	58.7	42.9	60.1	94.8	52	63.5	50.3	92.8	51.5	281.3	335.7	300.3	No	Yes	No	flavonol synthase/flavanone 3-hydroxylase, putative, expressed
OsAffx.6325.1.S1_at	LOC_Os09g18450	3	5.9E-07	1.6E-03	3.6E-05	2.3E-05	4.1E-02	1.3E-02	465.8	375.3	534.6	984.2	741.9	899.4	484.6	420.8	393.4	358.5	255.4	483.7	Yes	No	Yes	flavonol synthase/flavanone 3-hydroxylase, putative
Os.49805.1.S1_at	LOC_Os09g19160	3	6.5E-07	2.5E-03	2.3E-03	2.4E-05	5.6E-02	1.7E-01	53	26.5	24.2	40.4	72.6	65.8	712.1	1010.3	944.4	279.8	616	497.7	Yes	Yes	Yes	protein kinase domain containing protein, expressed
Os.51599.1.S1_at	LOC_Os09g19820	10	1.7E-10	1.0E-11	2.8E-09	1.3E-07	4.5E-07	2.9E-05	11804.6	11742.5	11731.7	2395.5	2039.3	2110.7	5218.5	4767	4404.9	667.5	476.2	344.1	Yes	No	No	aminopeptidase-like protein, putative, expressed
OsAffx.6337.1.S1_x_at	LOC_Os09g19970	9	1.0E-05	6.7E-07	1.5E-05	1.8E-04	2.0E-04	7.3E-03	547.5	364.3	295.3	49	40.8	38.6	108.3	154.5	111.7	30.2	7.2	4.6	No	No	No	expressed protein
Os.4759.1.S1_at	LOC_Os09g20220	10	3.1E-07	1.3E-09	1.9E-07	1.4E-05	4.2E-06	4.3E-04	8646.2	7701.7	8794.8	176.6	219.7	118.7	3584.2	3763.1	4151.8	428.7	340.2	289.5	No	No	No	glutathione S-transferase, putative, expressed
Os.49030.1.A1_s_at	LOC_Os09g20220	6	1.5E-05	3.7E-11	1.5E-06	2.4E-04	8.5E-07	1.6E-03	20954.1	22814.3	20457.7	558.2	1094.1	626.4	15472.4	16849.1	16279.3	3062.9	2030.6	1908.6	No	No	No	glutathione S-transferase, putative, expressed

Probe Set ID	Locus ID	probe #	ANOVA p-value			FDR q-value			Nipponbare MV			Nipponbare CK			93-11 MV			93-11 CK			Indel (Indica vs Japonica)			Annotation
			C	T	C×T	C	T	C×T	Repeat 1	Repeat 2	Repeat 3	Repeat 1	Repeat 2	Repeat 3	Repeat 1	Repeat 2	Repeat 3	Repeat 1	Repeat 2	Repeat 3	upstream	gene	downstream	
Os.20221.1.S1_at	LOC_Os09g21710	9	1.7E-05	1.1E-07	5.7E-05	2.7E-04	6.2E-05	1.7E-02	2172.7	2080.9	2425.2	261.1	333.9	312.9	1164.4	1368.3	1141	362.7	268	315.9	Yes	No	No	AN1-type zinc finger protein 2B, putative, expressed
Os.52863.1.S1_at	LOC_Os09g22000	7	9.8E-10	5.7E-04	4.6E-04	3.7E-07	2.0E-02	6.6E-02	1424.6	1148.6	1188.8	1003.7	856.1	824.6	14.8	25.1	8.6	52.4	23	6	No	No	No	hydrolases of the HAD superfamily, putative, expressed
Os.56136.1.S1_at	LOC_Os09g25200	6	7.5E-06	3.8E-07	9.2E-05	1.4E-04	1.4E-04	2.3E-02	272.9	259.5	259.9	41.5	62.5	47.8	143.8	141.1	89.2	44.1	9	42.2	Yes	Yes	Yes	ubiquitin-protein ligase/ zinc ion binding protein, putative
Os.8168.1.S1_at	LOC_Os09g25320	3	9.4E-04	6.7E-05	1.1E-03	5.8E-03	4.6E-03	1.1E-01	6521.5	7319.3	7507.6	4388.1	4979.9	4692.4	5490.8	5534.2	6080.8	5214.6	5445.5	4855	No	No	No	polyubiquitin 2, putative, expressed
Os.50202.1.S1_at	LOC_Os09g25934	3	3.0E-07	3.1E-03	7.7E-04	1.4E-05	6.4E-02	9.1E-02	297.5	337	282.9	281.3	268.3	221.4	12.5	21	27.5	43.7	26.1	5.2	Yes	Yes	Yes	expressed protein
OsAffx.6404.1.S1_at	LOC_Os09g26170	3	1.7E-04	1.3E-03	7.3E-04	1.6E-03	3.6E-02	8.8E-02	363.1	329.4	341.1	602	533.2	619	217.2	361.1	320.1	225.4	332.2	482	No	No	No	MYB36, putative, expressed
Os.53944.1.A1_s_at	LOC_Os09g26554	3	1.6E-12	1.2E-06	3.3E-06	1.3E-08	3.0E-04	2.7E-03	1053.9	956.2	1140.4	1329.1	1255.7	1235.1	12.8	1.6	12	4.8	2.7	15.7	Yes	Yes	Yes	expressed protein
Os.7932.1.S1_at	LOC_Os09g26880	3	7.6E-05	1.5E-06	4.3E-04	8.4E-04	3.4E-04	6.3E-02	11493.5	11021.2	11588.1	6572.3	6095.3	7282.3	9060.7	9082	9678.8	6674.6	6705	7277.4	Yes	Yes	No	aldehyde dehydrogenase family 7 member A1, putative, expressed
Os.7860.1.S1_at	LOC_Os09g27030	3	2.6E-03	1.3E-03	2.9E-03	1.2E-02	3.5E-02	2.0E-01	12415.2	11638.4	12658.7	10384.1	8792.4	10485.7	13553.7	12083.7	12605.9	13709.9	13519.9	12167.1	No	No	No	thiol protease aleurain precursor, putative, expressed
Os.9107.1.S1_a_at	LOC_Os09g27260	10	2.4E-08	1.9E-10	4.2E-08	2.6E-06	1.7E-06	1.7E-04	4018.7	3538.6	3579.7	204.9	211	190.8	774.6	755.5	957.3	44.8	113.5	74.2	No	No	No	TMV response-related gene product, putative, expressed
Os.9107.2.S1_x_at	LOC_Os09g27260	9	2.0E-07	7.4E-10	4.2E-07	1.1E-05	3.2E-06	7.3E-04	5425.2	5058.4	5444	434.9	651.2	506	2152.6	2952	2507.6	442.8	433.6	307.2	No	No	No	TMV response-related gene product, putative, expressed
Os.56907.1.S1_at	LOC_Os09g27510	3	9.5E-05	1.7E-06	2.1E-05	9.9E-04	3.8E-04	9.1E-03	296.8	340.3	458.1	202.8	367.2	290.6	570.4	952.2	708.4	171.8	243.2	324.4	No	No	No	cytochrome P450 76C4, putative, expressed
OsAffx.30103.4.S1_s_at	LOC_Os09g27930	11	2.8E-05	1.5E-06	2.1E-05	3.9E-04	3.5E-04	9.0E-03	2329.9	3059.6	2298.2	12.1	43.5	27.4	442.7	490.6	316.2	31.3	56.6	10.2	Yes	No	No	UBiQuitin family member, putative, expressed
Os.23468.1.A1_at	LOC_Os09g27950	3	3.3E-06	2.6E-07	2.4E-05	7.7E-05	1.1E-04	9.9E-03	221.5	275.3	252.7	57	120.5	111.3	106.1	165.3	111.1	52.8	75.2	58.4	No	No	No	avr9 elicitor response protein, putative, expressed
Os.27824.1.S1_at	LOC_Os09g28000	4	8.0E-08	1.1E-05	4.9E-05	5.8E-06	1.3E-03	1.6E-02	5544.6	6363.1	6219.5	3310	3082.8	3910.1	4059	4855	3890.3	3779.1	3307.5	3195.3	No	No	No	peptidase/ serine-type peptidase, putative, expressed
Os.23183.1.S1_at	LOC_Os09g28160	4	1.6E-04	1.7E-08	1.1E-04	1.5E-03	2.0E-05	2.6E-02	1612.7	2073	1765.6	397.1	393.6	379.6	990.6	1432.6	1007.9	422.9	397.8	310.4	No	No	No	phosphate carrier protein, mitochondrial precursor, putative, expressed
OsAffx.17942.1.S1_at	LOC_Os09g28160	7	4.4E-04	2.1E-08	1.7E-05	3.2E-03	2.2E-05	7.9E-03	903.6	746.8	669.4	215.8	195.4	166.7	416.9	441.1	438.2	248.8	174.4	149.5	No	No	No	phosphate carrier protein, mitochondrial precursor, putative, expressed
Os.55695.1.S1_at	LOC_Os09g28470	3	2.2E-04	3.5E-07	6.4E-04	1.9E-03	1.3E-04	8.2E-02	53.2	48.5	64.5	309.6	156.6	305.9	36.6	46.9	30.5	425.6	384.3	319.1	Yes	No	Yes	protein binding protein, putative, expressed
Os.11197.2.S1_x_at	LOC_Os09g30438	3	2.8E-07	1.3E-04	3.6E-04	1.4E-05	7.1E-03	5.6E-02	1930.4	1570.8	1253.7	1026.6	599.3	840.7	133.4	221.5	206	102.9	199.3	124.9	Yes	Yes	Yes	expressed protein
OsAffx.30103.2.S1_s_at	LOC_Os09g31019	4	2.3E-05	1.5E-05	6.9E-06	3.4E-04	1.7E-03	4.4E-03	89.1	235.2	127	1	2.3	17	25.4	48	44.1	4.9	26.9	30.2	No	No	Yes	UBQ1, putative, expressed
Os.12239.1.S1_at	LOC_Os09g31031	11	2.1E-06	1.8E-08	7.2E-06	5.6E-05	2.0E-05	4.5E-03	6117.5	5968.9	5661.5	712.8	687	782.5	2364.4	2643.5	1773.1	410.8	381.5	306	Yes	Yes	Yes	ubiquitin, putative, expressed
Os.15092.1.S1_at	LOC_Os09g31130	7	4.9E-05	1.1E-04	3.1E-06	5.9E-04	6.4E-03	2.6E-03	1212.6	1484.2	1462.6	582.7	657.4	631.4	548.5	672.2	663.1	741.4	822.9	695.4	No	No	No	tonoplast dicarboxylate transporter, putative, expressed
OsAffx.17976.1.S1_at	LOC_Os09g31410	11	1.9E-11	7.8E-11	9.5E-11	4.2E-08	1.2E-06	4.5E-06	103.3	108.9	117.7	142.3	120.2	100.6	335.8	331.4	351.9	2186.8	2430	2186.9	No	No	No	beta-glucosidase, chloroplast precursor, putative, expressed
OsAffx.6464.1.S1_at	LOC_Os09g31990	9	1.0E-05	1.5E-07	1.2E-05	1.8E-04	7.6E-05	6.5E-03	816.2	1037.3	1131.2	34	44.2	19.2	205.7	310.5	317	27.9	7	4.5	No	No	No	expressed protein
Os.55250.1.S1_at	LOC_Os09g32000	11	1.4E-05	5.1E-08	1.9E-05	2.3E-04	3.8E-05	8.7E-03	2654.4	2489.8	3242	114.8	175.5	147	972.4	1142.6	1091	136	109.8	99.1	No	No	Yes	TMV response-related gene product, putative, expressed
Os.9105.1.S1_at	LOC_Os09g33680	10	4.1E-08	7.4E-10	1.1E-07	3.7E-06	3.2E-06	3.1E-04	12643.2	13447	13687.8	3895.6	3589.8	3613.8	7267	8673.6	8434	2918.2	4482.2	3832.1	Yes	No	No	cyanogenic beta-glucosidase precursor, putative, expressed
OsAffx.30135.1.S1_at	LOC_Os09g34160	11	2.8E-08	1.4E-09	3.7E-08	2.9E-06	4.4E-06	1.6E-04	1488.5	1774.9	1456.3	98.2	117.3	132.8	400.8	466.5	218.9	28.9	46.1	30.7	No	Yes	Yes	resistance protein, putative, expressed
Os.48216.1.S1_at	LOC_Os09g34250	3	8.7E-06	7.7E-08	1.0E-03	1.6E-04	4.9E-05	1.1E-01	6900.9	9051.9	7480.5	1533.2	2697.1	1741.7	9867.4	10638.2	11867.9	5585.8	6623.2	5098.9	No	No	No	indole-3-acetate beta-glucosyltransferase, putative, expressed
OsAffx.6480.1.S1_at	LOC_Os09g34860	7	1.3E-06	1.3E-08	2.2E-06	4.0E-05	1.6E-05	2.1E-03	3944.6	4762.6	3678.2	1762.8	2033.9	1586.5	2370.6	2829.5	2512.7	1848.3	1753.9	1485.8	Yes	Yes	No	abhydrolase domain-containing protein 5, putative, expressed
Os.52276.1.S1_at	LOC_Os09g35980	3	1.0E-09	1.1E-03	1.1E-03	3.8E-07	3.2E-02	1.1E-01	33.2	39.6	29	33.9	42.2	36.6	2279	2263.9	2745.6	1171.8	1175.6	1484.3	No	No	No	expressed protein
Os.15026.1.S1_at	LOC_Os09g37540	9	8.9E-09	4.3E-09	3.1E-07	1.4E-06	8.7E-06	6.1E-04	1632.9	1798.2	1782.5	508.8	689.1	551.5	384.9	513.6	450.8	139	140.8	151.3	No	No	No	carboxy-lyase, putative, expressed
OsAffx.30180.4.S1_at	LOC_Os09g37840	4	5.1E-05	4.1E-05	4.1E-05	6.2E-04	3.3E-03	1.4E-02	127.7	198	146.5	41.7	36.1	43.4	41.8	7.8	27.7	18.8	45.1	22.9	Yes	Yes	No	ATP binding protein, putative
OsAffx.30180.4.S1_x_at	LOC_Os09g37840	6	2.6E-08	3.4E-08	4.7E-08	2.8E-06	3.0E-05	1.8E-04	189.2	253.8	202.4	31.7	15	37.1	34.5	34	7.4	45.3	14.9	27.4	Yes	Yes	No	ATP binding protein, putative
OsAffx.30196.1.S1_at	LOC_Os09g39070	9	3.0E-07	1.7E-05	1.9E-05	1.4E-05	1.8E-03	8.5E-03	1022.1	925.1	1179.9	3337.1	2186.9	4302.3	2.6	2.5	15.2	41.6	2.7	5.2	Yes	Yes	Yes	thiol protease SEN102 precursor, putative, expressed
OsAffx.6536.1.S1_at	LOC_Os09g39810	4	1.9E-04	2.0E-05	2.0E-04	1.7E-03	2.0E-03	3.8E-02	319.7	374.4	260.1	106.4	63.9	105.5	15	76.3	45.8	28.1	58.8	12.6	No	No	No	pathogenesis-related genes transcriptional activator PTI5, putative
OsAffx.18062.1.S1_x_at	LOC_Os09g39850	6	6.4E-06	2.3E-05	4.7E-05	1.3E-04	2.2E-03	1.5E-02	664.1	600.1	618.5	263.3	227.8	198.4	169.3	191.2	179.4	144.7	40.7	155	Yes	No	No	transcription factor TSRF1, putative, expressed
Os.52791.1.S1_at	LOC_Os09g39960	5	3.8E-07	1.2E-06	1.5E-04	1.7E-05	3.0E-04	3.2E-02	1946.1	2206.9	1975.9	1168.2	1023.9	1278.7	986.7	1244.4	1138.6	669	709.9	909.5	No	No	No	dynamin-related protein 1C, putative, expressed
Os.11341.1.S1_a_at	LOC_Os10g01134	4	7.3E-06	2.0E-08	9.0E-05	1.4E-04	2.2E-05	2.3E-02	1766.6	1706.4	1472.1	591.8	462	610.1	861.4	950.6	739.4	315	228	182.9	No	No	No	serine carboxypeptidase 1 precursor, putative, expressed
Os.46839.1.S1_at	LOC_Os10g02300	6	3.0E-09	2.1E-05	2.3E-05	7.2E-07	2.1E-03	9.7E-03	1099.7	1098.8	1290.1	563.8	389.4	650.7	231	334.2	244.5	164.5	227.8	195.4	Yes	Yes	Yes	expressed protein
Os.4020.1.S1_at	LOC_Os10g02380	11	1.4E-09	8.5E-05	7.3E-05	4.5E-07	5.4E-03	2.0E-02	4911.5	6169.9	5513.1	2487.4	3532.7	2316.7	87.2	116.9	113.6	184.4	217.7	87.8	No	Yes	No	NAD(P)H-dependent oxidoreductase, putative, expressed
Os.46268.1.S1_at	LOC_Os10g02390	11	4.0E-05	2.1E-05	1.6E-04	5.1E-04	2.1E-03	3.4E-02	936.5	1256	1366.1	3192.4	1617.6	1980.5	1508.8	1764.3	1330.7	11600.5	10672.4	7476.9	Yes	No	Yes	NAD(P)H-dependent oxidoreductase, putative, expressed
OsAffx.12171.1.S1_at	LOC_Os10g03400	10	2.2E-08	7.5E-10	1.2E-08	2.5E-06	3.2E-06	7.8E-05	110.1	135.7	74	346	238.6	284.1	79.5	70.3	51	827.3	778.9	816	No	No	Yes	CRAL/TRIO, N-terminus family protein, expressed
Os.47706.2.A1_at	LOC_Os10g03570	3	8.1E-08	9.3E-05	2.4E-04	5.8E-06	5.8E-03	4.4E-02	474.6	589.9	588	287.4	307.4	424.4	77.2	74.2	43.7	56.7	61.2	32.5	Yes	Yes	Yes	RGH1A, putative
Os.10358.1.A1_at	LOC_Os10g04270	9	5.1E-10	1.4E-05	1.6E-05	2.5E-07	1.6E-03	7.7E-03	1898.1	1679.9	1898.6	3225.5	2690.7	3038.5	28.6	40	55.9	59.7	67.8	23.5	Yes	Yes	Yes	protein GOS9, putative, expressed
Os.9427.1.S1_at	LOC_Os10g05020	3	5.3E-06	1.4E-07	1.7E-03	1.1E-04	7.5E-05	1.5E-01	2292.5	2197.3	2384.6	853.3	584.2	695.8	1016.7	1225.3	1089.6	220.3	283.2	293.4	No	No	No	transposon protein, putative, unclassified, expressed
Os.18989.1.S1_at	LOC_Os10g05660	4	1.8E-10	6.8E-04	8.0E-04	1.4E-07	2.3E-02	9.4E-02	58.4	17.6	37.2	62.8	68.3	52.9	958.9	1097.3	1186.1	640.9	728.5	889.8	No	No	No	thaumatin-like protein 1a precursor, putative, expressed
Os.5945.1.S1_at	LOC_Os10g06680	5	3.9E-04	1.3E-07	1.7E-04	3.0E-03	7.0E-05	3.5E-02	115.7	141.5	139.1	269.3	295.2	284.2	54.8	133.3	107.1	404.1	577.2	428.4	No	No	No	expressed protein
OsAffx.18163.1.S1_at	LOC_Os10g07040	8	1.3E-05	3.1E-06	2.2E-05	2.2E-04	5.7E-04	9.3E-03	46.1	68.8	71.6	450.6	156.2	139.9	62.2	101.5	83.9	1586.5	1465.3	1168.4	Yes	No	Yes	chalcone synthase 8, putative
Os.46718.1.S1_at	LOC_Os10g08319	9	1.6E-09	4.0E-06	8.3E-06	4.8E-07	6.7E-04	4.9E-03	242.9	267.7	207.3	391.8	240.9	204	1165.8	1290	1271.8	2319.9	2384.6	2527.8	No	No	Yes	cytochrome P450 76B1, putative, expressed
OsAffx.20713.1.S1_at	LOC_Os10g08620	9	8.1E-09	1.0E-08	1.3E-08	1.3E-06	1.4E-05	7.8E-05	19.5	32.8	4.9	89	23.8	33.5	37.9	63	15	510.4	478.8	426.9	No	No	Yes	chalcone synthase 8, putative
OsAffx.30342.1.S1_at	LOC_Os10g08670	9	4.7E-08	9.4E-08	9.7E-08	4.1E-06	5.7E-05	2.9E-04	15.1	5	9.3	30.1	31.7	22	74.5	113.4	65.6	1144.3	1063.1	817.5	No	No	Yes	chalcone synthase A, putative
OsAffx.20709.1.S1_at	LOC_Os10g08710	3	1.1E-09	8.9E-10	1.5E-09	4.0E-07	3.4E-06	2.0E-05	39.9	27.7	21.3	63.7	33.2	30.4	2.3	46.7	31.3	96.1	64.8	72.6	Yes	Yes	Yes	chalcone synthase 8, putative
OsAffx.18220.1.S1_x_at	LOC_Os10g11370	6	4.5E-05	5.8E-06	1.7E-05	5.5E-04	8.5E-04	8.0E-03	26.7	18.5	20.1	73.3	57.6	39.5	37.3	18.2	27.3	492.6	348.4	429.8	No	No	No	protease inhibitor/seed storage/LTP family protein
Os.33792.1.S1_at	LOC_Os10g12290	7	2.6E-09	4.8E-07	3.4E-07	6.5E-07	1.6E-04	6.4E-04	342.7	319.7	314	182.1	171	144.7	32.8	31	10.9	17.5	46.2	1.4	Yes	Yes	Yes	expressed protein
Os.18064.1.S1_at	LOC_Os10g14889	3	8.5E-04	4.3E-03	2.4E-03	5.4E-03	8.0E-02	1.8E-01	91.1	63	57.8	12.1	8.8	19.9	21.8	1.5	19.8	22.3	0.5	0.7	Yes	Yes	No	retrotransposon protein, putative, Ty3-gypsy subclass
Os.9840.1.S1_at	LOC_Os10g17489	6	5.3E-10	1.5E-06	9.2E-06	2.6E-07	3.4E-04	5.3E-03	1586.4	1942.9	1896.7	949.6	1247.8	1116.2	331	380.1	296.2	271.2	250.1	313.1	Yes	Yes	No	cytokinin-O-glucosyltransferase 2, putative, expressed
Os.8510.1.S1_at	LOC_Os10g18760	9	5.1E-08	1.9E-06	8.7E-06	4.3E-06	4.1E-04	5.1E-03	830.6	806.5	1212.8	3857.1	2474.3	4088.6	82.3	107.3	63	608.9	433.7	913.1	No	No	Yes	jasmonate-induced protein, putative, expressed
Os.46698.1.S1_at	LOC_Os10g20350	7	2.5E-10	2.6E-10	3.7E-10	1.6E-07	1.8E-06	1.1E-05	155	117.8	88.4	26.3	25.6	25.5	60.3	34.8	33.7	19.1	48.7	17.7	Yes	No	No	transparent testa 12 protein, putative, expressed
Os.46698.2.S1_x_at	LOC_Os10g20350	9	1.3E-10	9.8E-12	1.3E-10	1.1E-07	4.5E-07	5.5E-06	3568.9	2873.6	3158.5	184.7	153.7	114.7	597.5	695.8	525.2	165	190.6	107.4	Yes	No	No	transparent testa 12 protein, putative, expressed
Os.46698.3.S1_x_at	LOC_Os10g20390	8	2.3E-08	5.1E-08	8.0E-08	2.5E-06	3.8E-05	2.6E-04	333.1	269.4	199.3	6.1	4.6	2.9	4	3.5	3.6	2.8	6.8	4.2	No	Yes	Yes	transparent testa 12 protein, putative, expressed
Os.7350.1.S1_at	LOC_Os10g20450	4	5.1E-06	1.6E-04	1.1E-04	1.1E-04	8.3E-03	2.7E-02	191.9	183.2	119.7	73	122	49.1	36	29.5	48.6	29.7	38	32.9	Yes	No	No	transparent testa 12 protein, putative, expressed
Os.5049.1.S1_at	LOC_Os10g20470	10	1.8E-05	2.0E-08	6.2E-06	2.8E-04	2.2E-05	4.0E-03	1474.9	1406.9	1158.2	236	248.5	151.3	538.5	732.5	841.7	248.3	274.8	230.5	No	No	No	transparent testa 12 protein, putative, expressed
OsAffx.18335.1.S1_at	LOC_Os10g21630	4	4.9E-06	4.5E-06	8.5E-05	1.0E-04	7.2E-04	2.2E-02	230.8	240	238.1	89	81.2	39.7	49.8	155	96.4	47.7	76.7	73.4	No	No	No	expressed protein
Os.46695.1.S1_at	LOC_Os10g21790	3	1.4E-06	1.4E-08	4.6E-06	4.1E-05	1.7E-05	3.3E-03	120.1	78.3	50.8	57.1	34.6	37.2	142.7	127.7	238.5	53.8	48.2	36.3	No	No	No	dehydration stress-induced protein, putative, expressed
Os.26719.1.S1_at	LOC_Os10g22570	10	6.1E-08	8.1E-07	2.5E-07	4.8E-06	2.3E-04	5.4E-04	2903.2	3216.2	2746.5	889.3	1024.2	946.6	607.6	799	645.4	818.1	832.5	746.7	Yes	Yes	No	cellulase containing protein, expressed
Os.3350.1.S2_a_at	LOC_Os10g25130	3	4.7E-04	2.4E-07	2.8E-04	3.4E-03	1.1E-04	4.8E-02	5204.8	5127.1	5799.7	2610.6	2382.7	2560.6	4360.2	4355.8	5043.1	2754.2	1899.1	2436.2	Yes	No	No	alanine aminotransferase 2, putative, expressed
OsAffx.30528.1.S1_at	LOC_Os10g25340	3	2.5E-05	4.8E-06	2.4E-05	3.5E-04	7.5E-04	9.9E-03	10.1	9.8	36.1	104.2	68.3	77.8	13.6	8.2	6.7	51.5	40.1	66.8	No	Yes	Yes	esterase precursor, putative, expressed
Os.46833.1.S1_at	LOC_Os10g25870	3	4.9E-07	2.0E-04	1.3E-03	2.0E-05	9.7E-03	1.2E-01	231.1	241.8	229.2	526.2	333.8	438	71.7	38	45.8	121.3	157.8	159	Yes	Yes	Yes	dirigent-like protein pDIR12, putative, expressed
Os.46683.1.S1_at	LOC_Os10g25900	4	5.5E-05	6.1E-04	1.0E-03	6.5E-04	2.1E-02	1.1E-01	226.1	127	231.4	472.4	349.2	410.1	15.9	74.9	101.6	73	60.8	161.6	Yes	No	No	dirigent-like protein pDIR12, putative, expressed
Os.55787.1.S1_at	LOC_Os10g28000	9	1.2E-05	2.2E-07	1.5E-05	2.1E-04	9.9E-05	7.3E-03	461.1	484.4	355	128.2	159.5	121.9	156.5	265	202.2	123.4	132.6	80.1	No	No	No	glutathione reductase, chloroplast precursor, putative, expressed
OsAffx.8085.1.S1_at	LOC_Os10g30340	3	6.1E-04	1.1E-05	7.1E-04	4.2E-03	1.3E-03	8.7E-02	89.9	112.6	13.9	306.4	91.6	112.5	68.1	93.8	150.1	557.1	299.7	542.1	No	No	No	alpha-expansin 17 precursor, putative, expressed
Os.46565.1.S1_at	LOC_Os10g31240	7	8.4E-10	2.9E-06	1.9E-06	3.4E-07	5.4E-04	1.9E-03	426.9	509.1	473	797.5	964.8	919	71.1	76.3	73.1	49.3	68.2	91.7	Yes	Yes	No	expressed protein
OsAffx.20295.1.S1_at	LOC_Os10g31250	4	1.3E-06	2.0E-05	3.7E-06	3.9E-05	2.0E-03	2.9E-03	136.7	176.6	113.8	42.2	24	56.5	10.7	3.4	4.7	5.4	3.7	20.4	Yes	Yes	No	expressed protein
Os.48647.1.A1_at	LOC_Os10g31620	3	6.3E-06	8.6E-04	2.0E-03	1.3E-04	2.7E-02	1.6E-01	4.3	4.9	16.9	24.3	23.2	12.3	118.4	113	76	510.1	339.2	289.2	Yes	No	No	glycine-rich cell wall structural protein 2 precursor, putative, expressed
Os.47592.1.A1_at	LOC_Os10g31630	5	5.4E-04	2.0E-03	5.4E-03	3.8E-03	4.8E-02	2.8E-01	7.6	19.1	17.8	1.8	19.3	27.3	54.7	74.4	47.4	287.9	156.9	201.2	No	No	Yes	expressed protein
Os.21894.1.S1_at	LOC_Os10g31720	7	5.8E-07	2.5E-06	2.9E-06	2.2E-05	5.0E-04	2.5E-03	428.6	322.3	168.2	1219.5	1366.6	1456	184.4	200.7	229.5	196.5	227.4	200.8	Yes	No	No	glycine-rich cell wall structural protein 2 precursor, putative, expressed
Os.17506.1.S1_at	LOC_Os10g32680	4	8.4E-08	5.6E-07	3.5E-05	6.0E-06	1.8E-04	1.2E-02	3228.7	3418.5	2700.7	1705.5	2190.7	1674.1	1408.6	1869	1305	894.9	1030.9	897.9	No	No	No	expressed protein
Os.32630.1.S1_at	LOC_Os10g32700	5	5.7E-06	4.9E-06	4.8E-04	1.2E-04	7.7E-04	6.8E-02	2340.4	2063.7	2156.2	982.3	912.1	1031	812	1200.3	1073.9	480.6	493.7	788.8	No	No	No	hypersensitive-induced response protein, putative, expressed
OsAffx.30647.1.S1_x_at	LOC_Os10g32940	3	2.6E-05	6.1E-05	3.3E-04	3.7E-04	4.3E-03	5.4E-02	313.6	322.1	390.4	55.9	164.3	103.5	81.2	105.3	119.1	96.9	24.9	97.2	No	No	No	expressed protein
OsAffx.8033.1.S1_at	LOC_Os10g33060	10	1.4E-07	6.5E-07	1.2E-06	8.6E-06	2.0E-04	1.4E-03	38.8	21.6	29.7	65	33.9	55.4	135.1	136.5	115.6	634	567.4	472.9	Yes	No	Yes	receptor-like protein kinase precursor, putative
Os.39228.1.S1_at	LOC_Os10g34020	3	7.9E-07	4.1E-06	6.2E-06	2.8E-05	6.8E-04	4.0E-03	196.9	176.9	179.6	79.4	57.9	78.7	33.3	55.3	35.2	52	22.1	44.9	Yes	Yes	Yes	glutathione S-transferase GSTU6, putative, expressed
OsAffx.30676.2.S1_x_at	LOC_Os10g34910	5	3.1E-08	8.5E-06	4.7E-07	3.1E-06	1.1E-03	7.9E-04	1684.2	1508.4	1455.1	1292.3	1109.8	1324.7	845.1	974.5	963.3	1322.3	1184.5	1499.4	No	No	Yes	secretory protein, putative
Os.46450.1.S1_at	LOC_Os10g34930	8	7.9E-11	3.5E-08	3.5E-08	8.8E-08	3.0E-05	1.6E-04	3841.2	4688.6	4031.4	1386.9	2175.6	1610.4	404.3	676.3	330	216.8	279.7	227.2	No	No	No	secretory protein, putative, expressed
Os.46577.1.S1_at	LOC_Os10g34940	3	1.8E-05	3.3E-05	4.5E-03	2.7E-04	2.9E-03	2.5E-01	145.8	89.4	75.2	69.6	74.1	71.3	88	128.2	172.9	56.4	97.4	134.9	Yes	No	Yes	expressed protein
Os.53171.1.S1_at	LOC_Os10g35500	3	4.6E-09	3.3E-07	2.8E-07	9.2E-07	1.3E-04	5.7E-04	52.9	84.9	52.7	121.4	79.2	115.1	4.3	34.7	4.5	2.9	7.2	18.7	Yes	Yes	Yes	epoxide hydrolase, putative, expressed
Os.17865.1.S1_at	LOC_Os10g36980	4	2.6E-09	2.7E-04	2.1E-04	6.5E-07	1.2E-02	4.0E-02	1303	1259.1	1458.2	736.7	730.2	822.8	71	49.5	72.1	87	106.8	52.2	Yes	Yes	Yes	transposon protein, putative, unclassified, expressed
Os.4666.1.S1_at	LOC_Os10g37340	5	2.1E-06	1.2E-05	2.0E-04	5.7E-05	1.4E-03	3.9E-02	11633	11231.1	9565.2	6803	5164.9	5723	6400.8	5461.7	5797.4	4411.6	5017.9	5088.8	No	No	No	O-succinylhomoserine sulfhydrylase, putative, expressed
OsAffx.20193.1.S1_at	LOC_Os10g37570	3	2.2E-06	4.0E-05	8.4E-04	5.8E-05	3.2E-03	9.6E-02	282	173.6	149.3	99.3	15.5	114.1	47.3	67.6	17.1	64.2	28.1	10.1	Yes	No	No	F-box domain containing protein, expressed

Probe Set ID	Locus ID	probe #	ANOVA p-value			FDR q-value			Nipponbare MV			Nipponbare CK			93-11 MV			93-11 CK			Indel (Indica vs Japonica)			Annotation
			C	T	C×T	C	T	C×T	Repeat 1	Repeat 2	Repeat 3	Repeat 1	Repeat 2	Repeat 3	Repeat 1	Repeat 2	Repeat 3	Repeat 1	Repeat 2	Repeat 3	upstream	gene	downstream	
Os.38110.1.S1_at	LOC_Os10g37660	6	3.3E-07	5.2E-05	5.2E-05	1.5E-05	3.8E-03	1.6E-02	460.7	353.9	373.3	212.6	199.2	139	121.3	89.8	85.1	113.1	49	74.3	No	No	No	trehalase precursor, putative, expressed
Os.51639.1.S1_x_at	LOC_Os10g38040	4	9.3E-07	1.7E-05	2.4E-04	3.1E-05	1.8E-03	4.4E-02	1107.1	1072.7	922	623.7	858.1	766.6	473.4	390.4	418.9	356.6	317.8	326.3	No	No	No	lysM domain containing protein, expressed
Os.36568.1.S1_x_at	LOC_Os10g38150	4	2.9E-07	4.3E-06	1.5E-04	1.4E-05	7.0E-04	3.3E-02	770.2	766.3	823	489.9	386.9	424.7	542.9	522.7	648	395.6	352.1	406.9	Yes	Yes	Yes	glutathione S-transferase GSTU6, putative, expressed
Os.49755.1.S1_at	LOC_Os10g38189	11	3.3E-10	5.8E-08	1.2E-07	2.0E-07	4.1E-05	3.2E-04	97.6	140.5	107.5	47.1	138.9	79	5483.3	5333.9	5819.1	2373.8	1713.6	2028.9	Yes	Yes	Yes	glutathione S-transferase GSTU6, putative, expressed
Os.9013.1.S1_at	LOC_Os10g38340	11	1.3E-10	1.2E-10	3.2E-10	1.1E-07	1.4E-06	1.0E-05	11630.5	16254.3	12899.7	604.6	772.1	805.8	36.1	100.5	157.9	35.4	130.8	19.2	Yes	No	Yes	glutathione S-transferase GSTU6, putative, expressed
Os.46635.1.S1_x_at	LOC_Os10g38350	8	1.4E-07	2.6E-09	4.8E-07	8.2E-06	6.4E-06	8.1E-04	1745.4	1610.3	1383.3	281.8	302.6	305.3	1005.4	1049.1	1222.7	267.8	144.4	101.6	Yes	Yes	Yes	glutathione S-transferase GSTU6, putative, expressed
Os.13015.1.S1_at	LOC_Os10g38360	8	4.2E-11	1.0E-09	9.2E-10	6.2E-08	3.6E-06	1.5E-05	10559.6	7229.2	9253.9	1395.7	1165.7	1019.8	128.4	94.4	19.2	173.5	243.3	42.3	Yes	Yes	Yes	glutathione S-transferase GSTU6, putative, expressed
Os.47438.1.A1_at	LOC_Os10g38470	10	1.1E-13	9.7E-11	9.3E-11	3.4E-09	1.3E-06	4.5E-06	14.4	11.7	18.5	8	25.9	20.2	914.5	1038.2	1632	292.2	368.9	387	Yes	Yes	Yes	glutathione S-transferase GSTU6, putative, expressed
Os.5441.1.S1_at	LOC_Os10g38470	8	2.3E-08	2.3E-06	2.8E-06	2.5E-06	4.6E-04	2.4E-03	537.6	703.4	734.2	161	160.2	250.6	34.3	17.3	5.7	23.6	23	25.5	Yes	Yes	Yes	glutathione S-transferase GSTU6, putative, expressed
Os.46634.1.S1_x_at	LOC_Os10g38489	3	2.1E-07	2.1E-08	2.3E-05	1.1E-05	2.2E-05	9.7E-03	226.6	301.3	175.2	211.8	252.9	146.2	497	653.4	639.2	384.3	344.4	357.1	Yes	No	Yes	glutathione S-transferase GSTU6, putative, expressed
Os.2612.1.S1_at	LOC_Os10g38495	8	6.5E-06	3.8E-09	3.2E-06	1.3E-04	8.1E-06	2.7E-03	11416	12374.7	12771.7	1001.2	723.2	999	7364.7	7441	8344.7	2211.1	1480.2	1769.3	Yes	No	No	glutathione S-transferase GSTU6, putative, expressed
Os.7794.1.S1_at	LOC_Os10g38501	10	1.3E-12	1.2E-11	3.3E-11	1.2E-08	4.5E-07	2.0E-06	13154.9	13205.8	12515.4	3377.2	3274.8	3354.1	4241.9	4702.2	4918.9	1043.9	1224.7	1191.9	No	No	Yes	glutathione S-transferase GSTU6, putative, expressed
Os.7732.1.S1_at	LOC_Os10g38630	6	1.2E-11	5.2E-05	5.8E-05	3.2E-08	3.9E-03	1.8E-02	1860.3	1613.1	1586.7	1467.6	1170.6	1143.5	2.4	22.3	3.9	3.2	6.6	2.9	Yes	Yes	No	glutathione S-transferase GSTU6, putative, expressed
Os.21842.1.S1_at	LOC_Os10g38670	10	3.4E-07	1.6E-07	2.1E-06	1.6E-05	7.9E-05	2.1E-03	719.8	671.1	831.8	4755.6	1662	2786.5	2265.1	1899.8	1792.4	18046.8	19086.4	15382	No	No	Yes	glutathione S-transferase GSTU6, putative, expressed
OsAffx.20172.1.S1_at	LOC_Os10g38730	9	6.8E-10	7.5E-07	7.3E-06	3.0E-07	2.2E-04	4.5E-03	1268.3	955.2	927.4	679.3	407.3	601.4	85.6	118.3	162.5	123.6	105.3	191	Yes	No	No	glutathione S-transferase GSTU6, putative, expressed
OsAffx.20172.1.S1_x_at	LOC_Os10g38730	7	9.2E-08	9.5E-07	3.3E-05	6.3E-06	2.5E-04	1.2E-02	1484.2	1235.9	986.9	785.7	488.3	612.8	137.7	276.2	189	174.8	201.4	199.8	Yes	No	No	glutathione S-transferase GSTU6, putative, expressed
Os.4762.1.S1_at	LOC_Os10g38740	11	6.0E-10	8.1E-11	5.3E-09	2.8E-07	1.2E-06	4.8E-05	20074.3	22348.2	20805.2	5105.1	3703.3	5862.7	7751.1	6809.6	7729	1517.3	1541.3	1368.8	Yes	No	Yes	glutathione S-transferase GSTU6, putative, expressed
Os.9101.1.S1_at	LOC_Os10g38780	10	2.2E-10	3.3E-06	3.3E-06	1.5E-07	5.9E-04	2.7E-03	6461.7	8917.1	7034.3	3602.1	4542.3	4188.4	296.8	524.6	477.1	333.6	330.7	439.5	No	No	No	glutathione S-transferase GSTU6, putative, expressed
Os.46418.1.S1_at	LOC_Os10g39100	4	3.1E-09	1.3E-08	1.6E-07	7.3E-07	1.6E-05	3.9E-04	1752	1477.9	1690.8	422.8	480.8	452.5	806.4	930.4	938	318.1	244.9	312	No	No	No	domain of unknown function DUF614 containing protein, expressed
Os.37813.1.S1_at	LOC_Os10g39130	3	1.4E-11	2.5E-05	2.3E-05	3.5E-08	2.3E-03	9.7E-03	156.5	138.7	156.1	142.3	190.8	134.6	1642.7	1848.6	1598.9	1241.4	1468.2	1342.6	No	No	No	MADS-box transcription factor 56, putative, expressed
OsAffx.20168.1.S1_at	LOC_Os10g39160	9	1.6E-05	1.9E-06	7.5E-05	2.5E-04	4.1E-04	2.1E-02	6	2.9	1.6	206.4	78.8	114.8	66.5	2.6	57.1	663.7	477.8	594.5	No	No	No	peroxidase 2 precursor, putative
Os.22058.1.S1_at	LOC_Os10g39680	5	2.1E-08	4.5E-05	4.5E-05	2.4E-06	3.5E-03	1.5E-02	95.8	62.1	62.7	51.3	88.5	60.4	1466.7	2254.9	1915.5	832.4	712.3	1070.7	No	Yes	Yes	acidic endochitinase Q precursor, putative, expressed
Os.3415.1.S1_at	LOC_Os10g39680	6	1.0E-07	5.7E-05	1.2E-04	6.7E-06	4.1E-03	2.7E-02	1405	1773.6	2019	520.8	792.1	795.7	36.5	8.4	37.5	33.2	29.8	9.7	No	Yes	Yes	acidic endochitinase Q precursor, putative, expressed
OsAffx.20155.1.S1_at	LOC_Os10g39890	4	3.4E-04	4.7E-06	7.1E-04	2.7E-03	7.5E-04	8.7E-02	3.7	5.8	8.6	508.5	179	365.2	68.7	4	8.4	1024.8	599.2	1321.9	No	No	No	pollen proteins Ole e I family protein, expressed
Os.46629.1.S1_s_at	LOC_Os10g40480	5	4.6E-05	2.1E-07	1.8E-04	5.7E-04	9.6E-05	3.7E-02	330	317.8	412.6	3805.6	2238.9	3309.5	692.8	503.8	656.5	7556.8	6134.6	5530.2	No	No	Yes	cortical cell-delineating protein precursor, putative, expressed
OsAffx.18589.1.S1_s_at	LOC_Os10g40480	3	1.2E-03	3.9E-07	7.4E-03	7.1E-03	1.4E-04	3.2E-01	497.1	686.5	814.8	6185.2	4853.8	7283	1455.1	1295.7	1174.5	13493.7	12824.5	9407.5	No	No	Yes	cortical cell-delineating protein precursor, putative, expressed
Os.6838.1.S1_at	LOC_Os10g40510	6	5.5E-06	3.1E-07	6.3E-05	1.1E-04	1.2E-04	1.8E-02	4966.4	7224.8	7971.6	14347.6	13175.4	17473.3	5487.2	5945.4	5822.6	10930.9	9582.1	10271.9	No	No	Yes	cortical cell-delineating protein precursor, putative, expressed
Os.22427.1.S1_at	LOC_Os10g40520	10	5.9E-09	7.5E-06	9.0E-06	1.1E-06	1.0E-03	5.2E-03	2428.1	3301.3	3145.2	7206.4	8615.1	10794.9	28.5	16.4	9.5	282.5	63	39.2	No	No	No	cortical cell-delineating protein precursor, putative, expressed
Os.4741.2.S1_x_at	LOC_Os10g41930	11	2.7E-10	3.8E-10	7.9E-10	1.7E-07	2.2E-06	1.5E-05	11165.6	10530	9848.1	906.9	945.3	839.3	3074.8	2825.2	3390	482.4	422.5	549.5	No	No	No	catalytic/ hydrolase, putative, expressed
Os.4152.1.S1_at	LOC_Os10g41980	9	1.1E-08	2.2E-05	5.8E-05	1.6E-06	2.2E-03	1.7E-02	4985.8	3931	3810.3	2570.1	2035.5	2737.3	348	495.6	557.3	57.3	104.5	136.6	No	Yes	Yes	expressed protein
Os.46616.1.S1_at	LOC_Os10g41980	5	8.7E-08	4.5E-05	3.5E-05	6.1E-06	3.5E-03	1.2E-02	586.4	733.4	716.9	304.1	187.7	427	29	8.2	40.8	35.4	41.8	4.6	No	Yes	Yes	expressed protein
Os.46616.1.S1_x_at	LOC_Os10g41980	8	1.3E-09	6.7E-05	1.5E-04	4.4E-07	4.6E-03	3.3E-02	2012.8	1978.7	1901	1000.4	1187.3	1219.9	74.8	203.3	63.3	52.2	103.2	46.6	No	Yes	Yes	expressed protein
Os.46393.1.S1_x_at	LOC_Os10g41999	4	8.2E-08	2.6E-04	6.5E-04	5.9E-06	1.2E-02	8.2E-02	252.5	418.2	251.4	263.1	250.3	332.8	66.4	96.3	134.5	58.8	60.8	83.2	Yes	Yes	Yes	expressed protein
Os.16615.1.S1_at	LOC_Os10g42020	11	5.8E-10	4.7E-07	4.7E-07	2.7E-07	1.6E-04	7.9E-04	63.5	111.7	45.6	39.4	175.7	69.2	5603	7017.1	6630.9	2189.1	2798.4	2552.5	No	No	No	expressed protein
OsAffx.29329.1.S1_x_at	LOC_Os10g42020	3	6.9E-06	1.7E-03	1.3E-03	1.3E-04	4.3E-02	1.2E-01	197	285.5	133	82.4	119.1	95.4	34	42.1	16	10.7	25.1	37.5	No	No	No	expressed protein
Os.46613.1.S1_s_at	LOC_Os10g42900	3	1.8E-08	4.1E-07	4.4E-05	2.2E-06	1.5E-04	1.4E-02	285	224.4	236.8	586.1	471.9	445.2	695.5	608.5	511.1	1091.1	1095.9	978.2	No	Yes	Yes	peptide transporter PTR2, putative, expressed
Os.31991.1.S1_at	LOC_Os11g02290	7	2.7E-07	2.7E-05	2.5E-06	1.3E-05	2.5E-03	2.3E-03	511.5	412.1	502.7	112	186.2	199.1	10.1	6.1	17.5	29.1	10.7	8	No	No	No	expressed protein
Os.51389.1.S1_at	LOC_Os11g02670	3	9.1E-06	4.2E-05	4.6E-05	1.7E-04	3.3E-03	1.5E-02	128.1	116.6	127.5	126.8	104.3	150.6	156.8	183	185.3	291.5	286.8	378.3	No	Yes	Yes	protein binding protein, putative, expressed
Os.11908.1.S1_s_at	LOC_Os11g03230	7	4.1E-09	1.4E-04	1.3E-04	8.6E-07	7.6E-03	3.0E-02	139.2	80.3	130.8	156.5	124.5	66.4	679.8	700.9	729.7	457.9	405.7	438.6	Yes	No	Yes	nucleoside-triphosphatase, putative, expressed
Os.15801.1.S1_at	LOC_Os11g03290	6	3.4E-08	3.9E-06	1.9E-04	3.3E-06	6.6E-04	3.7E-02	397.9	529.1	385.8	1127.3	697.6	691.1	1426.6	1404.5	1640.4	3201.7	2442.9	3319.4	Yes	No	No	nucleoside-triphosphatase, putative, expressed
Os.15801.1.S1_x_at	LOC_Os11g03290	6	2.6E-08	4.6E-06	6.7E-04	2.7E-06	7.3E-04	8.3E-02	649.8	750.7	579.7	1366.2	1026	1008.4	2067.6	2150.5	1913.4	3684.7	3562.7	3453.3	Yes	No	No	nucleoside-triphosphatase, putative, expressed
Os.18177.1.S1_at	LOC_Os11g04104	8	1.9E-05	4.5E-07	3.9E-05	2.9E-04	1.6E-04	1.3E-02	1102.5	1167.7	967.5	401.1	370.1	380.2	594.6	673.3	727	319.6	447.6	406.6	No	Yes	Yes	carbohydrate transporter/ sugar porter/ transporter, putative, expressed
Os.9752.3.S1_x_at	LOC_Os11g04104	4	2.4E-06	3.0E-07	2.7E-05	6.1E-05	1.2E-04	1.1E-02	2741.7	3222.5	2569.8	1442.7	1380.6	1219.4	2179.3	2456.7	2616.5	1871.2	1804.2	2055.2	No	Yes	Yes	carbohydrate transporter/ sugar porter/ transporter, putative, expressed
Os.54318.1.S1_at	LOC_Os11g04290	3	1.1E-03	1.3E-05	5.6E-03	6.6E-03	1.5E-03	2.8E-01	831	1164.1	900.4	54.3	94.8	65.8	569.5	784.6	656.9	54.3	67.8	19.6	Yes	No	No	cytochrome P450 94A1, putative, expressed
OsAffx.31572.1.S1_at	LOC_Os11g04320	3	3.5E-08	9.0E-05	1.1E-03	3.4E-06	5.6E-03	1.1E-01	349.3	409.4	366	387.9	391	434.3	510.6	596.1	693.8	1058.5	825.8	870.5	No	No	Yes	phosphoglycerate mutase gpmB, putative, expressed
Os.50379.1.S1_at	LOC_Os11g04830	5	2.1E-07	1.6E-07	9.1E-06	1.1E-05	7.9E-05	5.3E-03	856.9	1004.1	926.9	336.2	330.7	427.7	356.2	364.5	276.1	206.7	218.2	156.5	No	No	No	cadmium tolerance factor, putative, expressed
Os.55208.1.S1_at	LOC_Os11g04860	5	2.2E-07	3.1E-06	1.8E-04	1.2E-05	5.7E-04	3.6E-02	407.9	603.8	552.3	262.1	289.1	314.8	1794.6	2111.3	2182.9	894.1	908.5	808.5	No	No	Yes	indole-3-acetate beta-glucosyltransferase, putative, expressed
Os.6118.1.S1_at	LOC_Os11g05700	8	5.1E-08	1.7E-07	1.5E-05	4.3E-06	8.3E-05	7.3E-03	2093	2333.5	1636.3	917.5	966.1	876.1	742.9	1013.4	940.4	445.3	684.5	516	Yes	No	No	multidrug resistance-associated protein 14, putative, expressed
Os.54511.1.S1_at	LOC_Os11g08380	8	3.1E-07	3.5E-07	1.4E-05	1.4E-05	1.3E-04	7.0E-03	334.4	400.1	376.3	776	605.8	857.7	565.9	640.3	545.1	1935.3	1991.3	2191.8	No	No	No	1-aminocyclopropane-1-carboxylate oxidase, putative, expressed
Os.18467.1.S1_at	LOC_Os11g09010	7	8.6E-10	5.7E-07	2.0E-06	3.4E-07	1.8E-04	1.9E-03	980.7	850.3	775.1	434.8	372.5	357.6	227.8	329.5	322.9	162.9	119.3	132.9	No	No	Yes	phytoalexin-deficient 4-1 protein, putative, expressed
Os.18467.1.S1_s_at	LOC_Os11g09010	4	3.3E-06	4.5E-06	9.3E-04	7.7E-05	7.3E-04	1.0E-01	2316.8	2365.8	2252.7	1101.3	1063	1016.4	1015.1	1528.7	1037.8	741.1	641.2	628.3	No	No	Yes	phytoalexin-deficient 4-1 protein, putative, expressed
Os.2426.1.A1_at	LOC_Os11g10470	6	6.1E-06	5.3E-09	1.4E-05	1.2E-04	9.8E-06	7.0E-03	3964.1	4958.2	4713.2	463.1	570	596.4	2322.5	3069.1	2697.2	509.6	320.4	337.4	No	No	No	expressed protein
Os.50870.2.S1_at	LOC_Os11g10550	5	4.8E-08	4.4E-05	1.4E-04	4.2E-06	3.4E-03	3.1E-02	465.8	694.5	491.1	223.1	348.2	375.2	2.6	9.1	2.2	27.2	3.6	2.2	Yes	Yes	Yes	NBS-LRR disease resistance protein, putative, expressed
Os.9836.1.S1_at	LOC_Os11g10590	3	4.0E-08	4.1E-04	5.1E-04	3.6E-06	1.6E-02	7.0E-02	928.2	782	873.4	1554.3	1170.6	1712.8	584.3	401.9	454.7	448.4	358.8	484.7	Yes	Yes	Yes	expressed protein
Os.20557.1.S1_s_at	LOC_Os11g10760	3	4.8E-11	1.3E-07	3.4E-06	6.6E-08	7.1E-05	2.7E-03	258.1	227.1	177.7	238.1	161.9	226.7	576	579.6	522.9	430	358.6	418.7	No	No	Yes	NBS-LRR disease resistance protein, putative, expressed
Os.52223.1.S1_at	LOC_Os11g12000	5	5.7E-09	3.1E-04	3.0E-04	1.1E-06	1.3E-02	5.0E-02	918.2	1028.5	948.4	517.2	704.7	741.3	3.5	44.7	32.6	46.1	69.5	46.6	Yes	Yes	Yes	NBS-LRR disease resistance protein, putative, expressed
Os.54085.1.S1_at	LOC_Os11g12050	5	7.9E-10	1.1E-04	1.2E-04	3.3E-07	6.5E-03	2.8E-02	725.7	834.4	758.6	468.6	488.2	556.2	3	13.7	7.1	23.4	2.1	2.9	Yes	Yes	Yes	NBS-LRR type disease resistance protein, putative, expressed
OsAffx.30940.1.S1_at	LOC_Os11g12330	5	7.7E-09	3.9E-05	6.6E-05	1.3E-06	3.2E-03	1.9E-02	1730.8	2035	1909.5	998.2	976.3	1280.4	236.2	250.1	230.6	177.2	181.4	171.5	Yes	Yes	Yes	disease resistance protein RPM1, putative, expressed
Os.4192.1.S1_at	LOC_Os11g12340	5	1.3E-08	9.0E-04	8.5E-04	1.8E-06	2.8E-02	9.7E-02	3493.5	4732	4379.3	1961.7	2929	3111.2	17.3	18.3	25.2	16.8	42.8	2.4	Yes	Yes	Yes	disease resistance protein RPM1, putative, expressed
OsAffx.30947.1.S1_at	LOC_Os11g12760	5	7.5E-10	1.6E-06	9.6E-07	3.2E-07	3.6E-04	1.2E-03	102.7	95.4	11.2	115.2	63.1	81.9	321.9	323.1	355.9	655.1	581.4	690.3	No	No	No	O-methyltransferase ZRP4, putative, expressed
OsAffx.7105.1.S1_x_at	LOC_Os11g13970	3	2.8E-05	2.7E-06	8.4E-05	3.8E-04	5.2E-04	2.2E-02	365.6	159.9	215.3	216.1	120	101.2	125.3	193.5	138	69.8	86.1	96.4	No	No	Yes	transferase, putative
Os.48915.1.S1_at	LOC_Os11g14380	7	3.8E-06	7.2E-06	6.1E-05	8.7E-05	1.0E-03	1.8E-02	222	258.3	221.3	89	120.1	87.3	51.1	85.1	78.3	36.7	60.6	72.2	Yes	No	No	resistance protein LR10, putative, expressed
Os.11271.2.S1_at	LOC_Os11g14544	4	6.7E-13	9.1E-05	5.0E-05	9.0E-09	5.7E-03	1.6E-02	6147.7	5761.8	4863.4	4370.4	4745.5	4860.4	34.4	58.5	33.2	70.7	60.4	25	Yes	Yes	No	expressed protein
Os.56946.1.S1_at	LOC_Os11g14910	11	1.4E-06	6.5E-07	2.4E-06	4.1E-05	2.0E-04	2.2E-03	1641.6	1705.7	1699.2	273.2	347.8	224.8	233.4	420.6	411.1	370.3	207.3	142.4	No	No	No	NADP-dependent oxidoreductase P1, putative, expressed
OsAffx.31010.1.S1_at	LOC_Os11g16970	4	6.3E-06	1.0E-04	4.1E-04	1.3E-04	6.1E-03	6.1E-02	226.9	192.8	149.9	496.1	410.1	483.7	42.9	61.1	17.9	41.4	66.4	80.8	No	No	No	carnitine racemase like protein, putative, expressed
OsAffx.18955.1.S1_at	LOC_Os11g20040	5	4.0E-04	2.8E-06	1.5E-04	3.0E-03	5.4E-04	3.3E-02	81.5	166.3	111.7	255.2	219.8	270.8	64	118.8	97.7	380.3	484.4	524.3	Yes	No	No	O-methyltransferase ZRP4, putative
Os.8673.1.S1_at	LOC_Os11g20090	9	3.2E-06	2.5E-07	1.3E-05	7.6E-05	1.1E-04	6.8E-03	813.8	1117.1	767.4	2332.9	1361.9	1668	907.8	1032.8	844.3	3653.9	3835.6	3883.3	Yes	No	No	O-methyltransferase ZRP4, putative, expressed
OsAffx.31072.1.S1_at	LOC_Os11g20160	11	2.4E-08	3.1E-07	2.2E-06	2.6E-06	1.2E-04	2.1E-03	1996.3	2285.4	1734.6	4557.6	2335.6	3500.9	5399.4	5639.7	5507.9	14499.2	12224	13596.1	No	No	Yes	O-methyltransferase ZRP4, putative, expressed
Os.36149.1.S1_x_at	LOC_Os11g23080	6	1.0E-06	3.3E-06	4.1E-06	3.3E-05	5.9E-04	3.1E-03	630.6	774.9	343	152.2	259.3	142.3	65.6	90.2	81.8	40.5	90.3	16.8	No	No	No	expressed protein
Os.48418.1.S1_at	LOC_Os11g24389	9	4.5E-09	3.0E-07	9.1E-06	9.1E-07	1.2E-04	5.3E-03	105.7	114.7	98.8	213.7	187.8	167.1	267.9	310.7	340.8	829.3	722.7	847	Yes	No	Yes	serine carboxypeptidase 1 precursor, putative, expressed
Os.9268.2.S1_at	LOC_Os11g24610	3	1.3E-10	2.8E-04	2.5E-04	1.1E-07	1.2E-02	4.5E-02	10624.1	8674.1	11882.8	12107	11762.9	14135.1	1091	1101.7	1744.5	1732.2	1673.5	2186.2	Yes	No	Yes	60S ribosomal protein L38, putative, expressed
Os.10315.1.S1_at	LOC_Os11g25454	5	3.6E-07	3.5E-05	1.7E-04	1.6E-05	3.0E-03	3.4E-02	836.7	807.8	764.8	450.3	436.2	396.5	263.3	308.6	256.7	304.6	215.7	172	Yes	No	Yes	cytokinin-N-glucosyltransferase 1, putative, expressed
Os.52253.1.S1_at	LOC_Os11g28420	4	1.6E-09	3.8E-05	4.0E-05	4.8E-07	3.1E-03	1.3E-02	748.7	784.9	764.9	480.1	590.9	562.5	16	22.4	0.5	47.8	22.5	7.2	Yes	Yes	Yes	expressed protein
Os.27675.1.S1_at	LOC_Os11g28940	9	7.4E-07	1.0E-04	8.2E-05	2.6E-05	6.1E-03	2.2E-02	343.7	422.4	354.6	149.6	182.2	147	36.2	2.1	58.8	41.9	55.3	23.5	No	Yes	Yes	expressed protein
Os.55827.1.S1_at	LOC_Os11g29870	7	8.6E-10	2.4E-08	1.8E-08	3.4E-07	2.4E-05	1.0E-04	944.2	1098.2	1079.9	203.7	296.7	224.1	121.2	285.7	219	88.8	88.4	57.2	No	No	Yes	OsWRKY72 - Superfamily of rice TFs having WRKY and zinc finger domains, expressed
OsAffx.19095.1.S1_at	LOC_Os11g31060	4	1.4E-05	2.8E-04	7.2E-04	2.3E-04	1.2E-02	8.7E-02	207.2	281.3	203.9	90.9	68.2	107.3	37.6	42.6	12.1	14.1	65.7	52.1	No	No	Yes	BAG domain containing protein, expressed
Os.4974.1.S1_at	LOC_Os11g31190	6	1.3E-08	2.3E-08	5.4E-08	1.8E-06	2.3E-05	2.0E-04	1756.6	3638.3	2409	869.9	1438.4	875.7	676.6	565.8	482.3	537.5	559.1	457.9	Yes	No	No	mtN3-like protein, putative, expressed
Os.4974.1.S1_x_at	LOC_Os11g31190	6	5.6E-06	1.7E-04	1.8E-04	1.2E-04	8.8E-03	3.7E-02	2687.3	3832.6	2909.3	1262.7	1381.4	1071.9	702.9	845.3	1010.3	1013.8	794.7	843.1	Yes	No	No	mtN3-like protein, putative, expressed
Os.54340.1.S1_at	LOC_Os11g31250	4	9.8E-13	1.8E-05	1.7E-05	1.1E-08	1.9E-03	8.0E-03	646.8	616.1	574.8	523.9	606.4	393.9	4.2	3.8	4.2	9.2	1.7	2.4	Yes	No	Yes	hypothetical protein
Os.7793.1.S1_at	LOC_Os11g31570	11	7.3E-08	5.1E-08	2.5E-06	5.4E-06	3.8E-05	2.3E-03	183.3	175.4	170.7	408	365.9	412.7	248.9	358.4	316.4	1263.6	1158.3	1328.4	No	No	No	expressed protein
Os.40244.1.S1_at	LOC_Os11g31940	3	5.3E-10	5.4E-06	7.3E-06	2.6E-07	8.1E-04	4.5E-03	334.6	289.6	356.1	582.8	516.5	636.1	260.9	301.9	189.5	153.3	183.5	176.8	No	No	No	esterase precursor, putative, expressed
Os.9988.1.S1_at	LOC_Os11g32890	3	1.4E-07	1.3E-04	1.7E-04	8.6E-06	7.4E-03	3.4E-02	66.2	50.1	57.4	53.8	55.3	46.4	151.2	268.6	176.6	266.2	468	353.3	No	No	Yes	expressed protein
Os.11465.1.S1_at	LOC_Os11g33270	10	1.7E-09	1.8E-07	3.0E-07	5.0E-07	8.5E-05	6.0E-04	1441.6	1170.6	1416.4	3865.1	3083	3671.7	49.8	73.4	43.2	178.1	195.2	137.1	Yes	No	Yes	xyloglucan endotransglucosylase/hydrolase precursor, putative, expressed
OsAffx.19161.1.S1_at	LOC_Os11g34880	3	8.6E-09	7.1E-05	4.8E-05	1.4E-06	4.8E-03	1.5E-02	78.8	75.8	47.1	47.1	74.5	41.2	131.5	227.7	149.9	97.6	106.2	121.4	No	No	No	NB-ARC domain containing protein, expressed
OsAffx.19165.1.S1_at	LOC_Os11g35040	7	7.2E-05	1.4E-05	2.6E-07	8.0E-04	1.6E-03	5.5E-04	1384.8	1302.1	1218.9	291.1	286.1	259.9	830.9	1044.9	1034.5	1205.3	898.7	1062.9	No	No	No	tyrosine aminotransferase, putative, expressed
Os.17190.1.A1_x_at	LOC_Os11g36150	3	1.8E-07	1.6E-03	9.8E-04	1.0E-05	4.1E-02	1.1E-01	28.5	4.1	41.9	3.9	39.6	32.8	294.4	254	452	159.3	200	271.7	Yes	Yes	Yes	receptor-like protein kinase precursor, putative, expressed
Os.49819.1.S1_at	LOC_Os11g36200	4	7.2E-07	2.9E-04	8.8E-04	2.6E-05	1.3E-02	9.9E-02	210.1	175.4	184.9	137.4	143	97.2	25.1	32.9	3.9	17	33.5	13.5	Yes	No	No	receptor kinase-like protein, putative, expressed
Os.53816.1.S1_at	LOC_Os11g37040	4	2.3E-08	1.4E-04	1.3E-04	2.5E-06	7.6E-03	2.9E-02	437.2	521.3	443.5	827.5	763.4	867.4	8.5	3.8	7.2	28.1	21.9	34.4	Yes	Yes	Yes	expressed protein

Probe Set ID	Locus ID	probe #	ANOVA p-value			FDR q-value			Nipponbare MV			Nipponbare CK			93-11 MV			93-11 CK			Indel (Indica vs Japonica)			Annotation
			C	T	C×T	C	T	C×T	Repeat 1	Repeat 2	Repeat 3	Repeat 1	Repeat 2	Repeat 3	Repeat 1	Repeat 2	Repeat 3	Repeat 1	Repeat 2	Repeat 3	upstream	gene	downstream	
OsAffx.7332.1.S1_at	LOC_Os11g37940	11	3.3E-11	1.2E-09	1.8E-09	5.6E-08	4.0E-06	2.2E-05	5765.7	4833.7	5624.4	2018.8	1394.3	1980.6	386.4	358	455.8	386.7	305.8	420.5	No	No	No	win2 precursor, putative, expressed
OsAffx.31373.1.S1_at	LOC_Os11g38780	10	4.2E-07	3.3E-06	4.4E-06	1.8E-05	5.9E-04	3.2E-03	926.4	1139	1129.6	131.8	252.3	273.5	140.9	327.3	202.7	30.4	18.3	125.1	No	No	Yes	calcium ion binding protein, putative
OsAffx.7355.1.S1_at	LOC_Os11g39530	5	1.8E-08	3.5E-04	4.2E-04	2.2E-06	1.4E-02	6.2E-02	368	365.1	258.7	590.7	368.4	515.7	24.5	3.5	4.3	1.4	22.9	2.7	Yes	Yes	Yes	jacalin-like lectin domain containing protein, expressed
Os.54291.1.S1_at	LOC_Os11g42200	8	1.2E-06	2.3E-07	8.8E-07	3.8E-05	1.0E-04	1.2E-03	337.8	314.3	337.3	26.9	64.2	11.4	37.5	132.8	64.4	56.6	35.8	2.2	No	No	No	laccase LAC2-1, putative, expressed
OsAffx.28903.1.S1_x_at	LOC_Os11g44014	4	5.7E-10	2.6E-05	3.3E-05	2.7E-07	2.5E-03	1.2E-02	1.7	5.4	2.4	1.5	2	3.2	160.6	172.1	264.5	174	140.7	133.7	Yes	Yes	Yes	retrotransposon protein, putative, unclassified
OsAffx.19366.2.S1_at	LOC_Os11g44170	5	7.9E-09	3.8E-04	3.4E-04	1.3E-06	1.5E-02	5.5E-02	466.3	648.7	421.6	251.5	267.5	265.9	37	19.9	4.9	2.2	4	2.1	Yes	Yes	Yes	conserved hypothetical protein
OsAffx.31470.1.S1_at	LOC_Os11g44310	5	2.0E-05	2.1E-05	2.2E-04	3.0E-04	2.1E-03	4.1E-02	247.7	192	179	9.9	52.8	100.3	32.7	46.1	44.4	25.6	32.2	7	No	No	Yes	expressed protein
OsAffx.31467.3.S1_at	LOC_Os11g44430	5	1.1E-08	3.3E-06	1.3E-05	1.6E-06	5.9E-04	6.6E-03	306.4	308.3	200.8	146.7	171.8	111.4	5.9	5.9	25.6	6.5	46.2	45.9	Yes	Yes	Yes	protein kinase domain containing protein, expressed
OsAffx.31467.3.S1_s_at	LOC_Os11g44430	7	7.0E-10	2.2E-06	2.5E-06	3.1E-07	4.4E-04	2.3E-03	911.3	682.4	687.2	444.4	345.7	374.1	49.6	75.8	52.1	44.1	48.1	100.9	Yes	Yes	Yes	protein kinase domain containing protein, expressed
Os.27110.1.A1_at	LOC_Os11g44560	8	1.5E-08	4.6E-05	3.0E-05	1.9E-06	3.5E-03	1.2E-02	1237	1151	1337.2	409.5	451.8	663.2	20.1	19.4	46.7	12.5	30	37	Yes	Yes	Yes	protein kinase domain containing protein, expressed
OsAffx.31475.1.S1_at	LOC_Os11g44630	11	2.9E-07	7.8E-06	2.7E-05	1.4E-05	1.1E-03	1.1E-02	767.3	618	544.6	214.7	252.6	333.3	29.3	22.6	21.7	30.1	47.8	21	Yes	Yes	Yes	expressed protein
OsAffx.19384.1.S1_at	LOC_Os11g45160	4	9.9E-06	8.4E-05	1.2E-04	1.8E-04	5.4E-03	2.7E-02	22.5	29.6	25.9	52.8	44.2	53.2	0.9	7.6	0.9	4.8	1	5.3	Yes	Yes	Yes	NBS-LRR disease resistance protein, putative
OsAffx.19384.1.S1_x_at	LOC_Os11g45160	4	5.1E-05	3.8E-05	5.8E-05	6.1E-04	3.1E-03	1.8E-02	58.7	49.1	44.7	146.3	139.5	131.2	40.4	23.5	21.7	27.5	66.9	47.6	Yes	Yes	Yes	NBS-LRR disease resistance protein, putative
OsAffx.19399.1.S1_at	LOC_Os11g45970	5	5.0E-05	3.2E-05	1.9E-04	6.1E-04	2.8E-03	3.8E-02	35.8	115.9	51	237.7	215.9	237.6	31.2	36.2	23.7	71.1	85.1	81.3	No	Yes	Yes	NBS-LRR disease resistance protein, putative, expressed
Os.19895.1.S1_at	LOC_Os11g46860	11	7.9E-12	7.1E-08	9.8E-08	2.7E-08	4.7E-05	2.9E-04	210.5	229.5	252.3	375.5	620.4	578.6	11424.1	12387.3	11508.2	18493.4	18937.2	17343.3	Yes	Yes	No	protein kinase, putative, expressed
Os.25171.1.A1_at	LOC_Os11g47140	9	2.0E-08	2.0E-05	2.2E-05	2.4E-06	2.0E-03	9.4E-03	809.8	973.5	1326.6	2572.5	2286.7	3049.6	28.4	49.3	18.9	68.5	36.8	61.1	Yes	Yes	Yes	OsWAK123 - OsWAK receptor-like protein kinase, expressed
Os.25171.2.S1_at	LOC_Os11g47140	6	8.5E-08	6.1E-05	1.0E-04	6.0E-06	4.3E-03	2.5E-02	93.9	61.2	104.8	337.5	170.4	189.1	3.2	12.4	2.7	2.5	3.5	7.7	Yes	Yes	Yes	OsWAK123 - OsWAK receptor-like protein kinase, expressed
OsAffx.19414.2.S1_at	LOC_Os11g47150	5	8.6E-06	1.8E-05	7.1E-06	1.6E-04	1.9E-03	4.4E-03	64.4	76.5	25	180.3	254	144.6	15.6	37.5	29.6	7.1	17.4	28	Yes	Yes	Yes	OsWAK122 - OsWAK receptor-like protein kinase
Os.172.1.S1_a_at	LOC_Os11g47500	7	2.8E-08	3.7E-08	3.3E-08	2.9E-06	3.2E-05	1.5E-04	1826.6	1491.1	1817.5	43.2	123.6	67.2	71.4	54	38.2	3.8	7.9	11.7	Yes	Yes	Yes	xylanase inhibitor protein 1 precursor, putative, expressed
Os.25497.1.S1_at	LOC_Os11g47500	10	6.6E-08	1.9E-07	2.0E-07	5.1E-06	8.8E-05	4.5E-04	15.6	18.9	27.3	2.8	46.6	1.5	3090.2	4408.4	3508	233.3	306.1	301.1	Yes	Yes	Yes	xylanase inhibitor protein 1 precursor, putative, expressed
Os.25497.1.S1_x_at	LOC_Os11g47500	10	1.5E-07	1.8E-07	2.7E-07	8.7E-06	8.8E-05	5.7E-04	15.2	2	8.1	1	46.2	0.7	2522.3	3935	2813.5	177.9	227.2	220.2	Yes	Yes	Yes	xylanase inhibitor protein 1 precursor, putative, expressed
Os.4670.1.S1_at	LOC_Os11g47510	3	3.5E-08	6.1E-09	3.6E-08	3.3E-06	1.0E-05	1.6E-04	23.5	44.3	77	20.3	32.5	20	77.7	57.3	80.9	29.2	31.5	31.8	Yes	No	Yes	xylanase inhibitor protein 1 precursor, putative, expressed
Os.9347.1.S1_at	LOC_Os11g47580	6	8.3E-07	6.7E-08	1.5E-06	2.9E-05	4.5E-05	1.6E-03	781.4	527.6	736.2	62.7	77.9	83.1	142	154.8	312.8	32.7	39.2	24.1	No	No	Yes	xylanase inhibitor protein 1 precursor, putative, expressed
Os.55400.2.S1_at	LOC_Os11g47670	4	1.7E-08	7.5E-08	5.8E-08	2.1E-06	4.8E-05	2.1E-04	211.1	154.2	186.7	78	95.6	61.2	53	60.7	3.5	42.9	42	63	Yes	Yes	Yes	pathogenesis-related protein 5 precursor, putative, expressed
OsAffx.19428.1.S1_s_at	LOC_Os11g47680	7	1.8E-10	1.2E-08	1.2E-08	1.3E-07	1.5E-05	7.8E-05	1208.8	1126.1	1220.6	234	345.9	260.2	153.2	255.8	231.8	34.6	93.4	94.9	Yes	No	No	thaumatin-like protein 2 precursor, putative, expressed
OsAffx.19473.1.S1_at	LOC_Os12g03740	7	1.4E-10	1.1E-07	1.1E-07	1.2E-07	6.2E-05	3.2E-04	835.8	892.9	906	408.6	505.3	475.3	18.2	55.6	53.6	7.4	4.6	21.1	No	Yes	No	ATPP2-B10, putative, expressed
OsAffx.19473.1.S1_s_at	LOC_Os12g03740	5	9.3E-09	4.6E-06	4.6E-05	1.4E-06	7.3E-04	1.5E-02	1478.2	1693.7	1815.5	703.4	958.2	848.1	88.4	95.2	100.4	53.3	73.5	53.8	No	Yes	No	ATPP2-B10, putative, expressed
Os.9752.2.S1_x_at	LOC_Os12g03899	5	9.7E-05	9.6E-08	5.0E-05	1.0E-03	5.8E-05	1.6E-02	2564.1	2697.9	2602.2	1183.7	1428.8	1369.5	2162	2373.4	2309.6	1574.2	1601.2	1839.3	Yes	Yes	No	carbohydrate transporter/ sugar porter/ transporter, putative, expressed
Os.5943.1.S1_at	LOC_Os12g04150	3	1.2E-08	2.0E-06	8.9E-05	1.7E-06	4.2E-04	2.3E-02	12345.4	15449.7	13099.4	7630.2	9225.9	9640.9	4578.7	6302.6	4734.5	2259.2	2384.2	2481.3	Yes	No	No	gibberellin receptor GID1L2, putative, expressed
Os.13051.1.S1_at	LOC_Os12g04980	6	9.1E-07	3.0E-06	4.9E-06	3.1E-05	5.5E-04	3.4E-03	81.9	93.9	74.3	47.8	57.4	69	303.1	516.1	387.3	67.6	120.2	77.7	No	No	No	meiotic recombination protein DMC1, putative, expressed
Os.12064.1.S1_at	LOC_Os12g05080	7	3.9E-07	1.9E-04	6.9E-04	1.7E-05	9.3E-03	8.5E-02	815.8	1057.3	874.1	540.3	556	516.2	376.5	425.9	413.5	394.2	431.2	362.4	No	No	No	expressed protein
OsAffx.19506.1.S1_at	LOC_Os12g05510	7	3.9E-05	2.3E-07	3.8E-05	5.0E-04	1.0E-04	1.3E-02	127.2	163.6	114.5	46.1	70.7	1.4	416	492.9	419.5	19.2	94.2	73.4	Yes	No	No	conserved hypothetical protein
Os.51532.1.S1_at	LOC_Os12g05840	9	2.5E-08	6.1E-07	1.2E-06	2.7E-06	1.9E-04	1.4E-03	616.8	584	589.3	153.6	108.7	127.7	111.9	112.9	165.2	137.8	67.6	95.1	No	No	No	germin-like protein subfamily 1 member 8 precursor, putative, expressed
Os.55421.1.S1_at	LOC_Os12g06550	3	2.3E-06	4.7E-04	1.6E-04	6.0E-05	1.8E-02	3.4E-02	236.9	137.5	158.7	73.1	122.7	116.9	38.8	39.8	48.9	18.1	54.6	4.3	No	Yes	No	expressed protein
Os.47560.1.S1_at	LOC_Os12g06660	5	2.2E-06	1.6E-07	3.3E-05	5.9E-05	7.9E-05	1.2E-02	407.3	435	813	1762.3	1072	1664.7	1032.9	695.1	648	3311.2	2753.2	3123.7	No	No	Yes	actin-7, putative, expressed
Os.53000.1.S1_at	LOC_Os12g06840	3	3.2E-04	5.6E-06	4.8E-04	2.5E-03	8.3E-04	6.8E-02	23	19.6	26.7	98.2	148.7	78	36.8	9.3	38.1	281.5	348.3	160.8	No	Yes	No	protein binding protein, putative, expressed
Os.17909.1.S1_a_at	LOC_Os12g08260	4	7.0E-06	1.7E-09	2.4E-04	1.4E-04	4.8E-06	4.4E-02	2554.7	2117	2076.5	1235.9	1113.5	1182.7	1575.8	1691.3	1577.6	1183.6	1357.8	1179	No	No	No	pyruvate dehydrogenase E1 component, alpha subunit, putative, expressed
OsAffx.7530.1.S1_s_at	LOC_Os12g08930	7	6.0E-07	2.1E-10	2.8E-07	2.3E-05	1.7E-06	5.7E-04	2045.5	2229.2	2342.5	93.5	164.6	68.6	1093.2	1504.7	1201.2	70.3	101.4	116.9	No	No	No	expressed protein
Os.51603.1.S1_at	LOC_Os12g10220	7	8.9E-11	4.9E-06	6.0E-06	9.1E-08	7.6E-04	3.9E-03	3251.3	2763.5	3607.3	5975.7	4617.1	5376.4	36.4	52.5	40	83.8	79.9	50.2	Yes	Yes	Yes	expressed protein
Os.54234.1.S1_at	LOC_Os12g10330	3	3.5E-07	1.5E-03	1.5E-03	1.6E-05	3.9E-02	1.3E-01	875.7	1022.8	1112.6	1457	1775.2	2022.1	3.9	3.1	8.1	64.8	4.7	3.5	Yes	Yes	Yes	NB-ARC domain containing protein, expressed
Os.54200.1.S1_at	LOC_Os12g10340	10	6.0E-08	3.5E-07	6.8E-07	4.7E-06	1.3E-04	9.9E-04	199.6	186.4	180.4	536.6	494.7	611.6	30.9	37.5	54.3	60	55.9	75.4	Yes	Yes	No	rust resistance-like protein RP1-3, putative, expressed
Os.50877.1.S1_at	LOC_Os12g10710	4	2.0E-07	2.6E-06	4.7E-04	1.1E-05	5.0E-04	6.7E-02	1151.5	1411.3	1064.3	539.7	579.4	609.2	216.2	426.4	249.5	169.7	92.8	166	No	No	No	NB-ARC domain containing protein, expressed
OsAffx.31701.1.S1_at	LOC_Os12g11370	3	3.3E-07	9.0E-07	9.9E-06	1.5E-05	2.5E-04	5.6E-03	198.9	294.6	211.3	52.1	86.8	70.6	66	78.8	62.8	22.8	41	43.5	No	Yes	Yes	verticillium wilt disease resistance protein, putative, expressed
OsAffx.19617.1.S1_at	LOC_Os12g11840	8	3.9E-07	7.1E-06	1.3E-05	1.7E-05	9.9E-04	6.7E-03	597.8	719.2	585.5	178.2	133.3	212.1	35.2	154.1	123.4	17.2	21.8	88.1	Yes	No	No	expressed protein
Os.40001.1.A1_at	LOC_Os12g12080	8	3.6E-10	4.2E-10	4.2E-10	2.1E-07	2.4E-06	1.1E-05	1631.7	1066.2	1367.1	56.9	94.2	54.3	10.8	2.4	4.2	5.9	29.1	11.8	Yes	Yes	Yes	retrotransposon protein, putative, Ty3-gypsy subclass, expressed
OsAffx.19632.1.S1_at	LOC_Os12g12590	7	1.1E-07	1.1E-06	2.2E-06	7.2E-06	2.8E-04	2.1E-03	309.6	321	314.3	566.6	226.9	320.7	477.5	542.9	317.5	1417.4	1040.9	1261	Yes	Yes	Yes	NADP-dependent oxidoreductase P1, putative, expressed
Os.51323.1.S1_at	LOC_Os12g14540	11	7.9E-10	6.6E-09	2.4E-09	3.3E-07	1.1E-05	2.6E-05	1284.7	1376.3	1382.3	218.9	272.7	176.1	2.7	22.7	27.4	1.1	22.5	0.8	Yes	Yes	Yes	expressed protein
Os.34112.1.S1_at	LOC_Os12g15530	7	9.1E-11	9.5E-09	7.0E-09	9.3E-08	1.3E-05	5.6E-05	923.3	642.7	826.4	402.2	348.1	172	1	34.7	39.5	28.3	58.4	3.4	Yes	Yes	No	retrotransposon protein, putative, unclassified, expressed
Os.17659.1.S1_at	LOC_Os12g15680	11	1.3E-09	2.3E-10	2.2E-09	4.3E-07	1.7E-06	2.4E-05	1782.5	1465	1617.1	224.1	297.6	202	560	647.8	681.1	163.8	92.5	62.6	Yes	No	Yes	L-ascorbate oxidase precursor, putative, expressed
OsAffx.7606.1.S1_at	LOC_Os12g15680	10	2.9E-10	7.5E-10	6.5E-10	1.8E-07	3.2E-06	1.5E-05	522.6	447.5	450.9	85.4	57.7	71.9	97.6	82.4	52.2	4.5	28.3	5	Yes	No	Yes	L-ascorbate oxidase precursor, putative, expressed
Os.52760.1.S1_at	LOC_Os12g16650	10	1.0E-07	2.1E-06	7.7E-05	6.9E-06	4.3E-04	2.1E-02	613.9	581.6	620.1	1156.8	770.6	806.7	959.7	1552.6	1219.3	2781.8	3035.3	3243.6	Yes	No	No	cyclopropane fatty acid synthase, putative, expressed
Os.25621.2.S1_at	LOC_Os12g16720	6	3.2E-06	4.9E-06	2.3E-04	7.5E-05	7.6E-04	4.2E-02	2770.3	3520.7	3160.5	1502.8	1280.3	1350.3	1674.6	1655.5	1301.6	1262.8	1362.5	1457.2	No	No	Yes	cytochrome P450 71A1, putative, expressed
Os.5320.1.S1_at	LOC_Os12g22970	4	1.9E-06	6.1E-06	1.7E-06	5.2E-05	8.8E-04	1.8E-03	67.8	104.7	86.2	35.2	26.9	13.2	2	36.8	4.1	2	1.2	28	No	No	No	expressed protein
Os.4956.1.S1_at	LOC_Os12g23170	7	8.7E-07	4.7E-04	7.1E-04	3.0E-05	1.8E-02	8.7E-02	7.6	50.2	21.5	27.1	40.6	31.9	383.2	366.6	300.2	121.1	284.1	163.2	Yes	No	No	beta-glucosidase homolog precursor, putative, expressed
Os.36283.1.S1_at	LOC_Os12g25090	7	1.4E-05	2.0E-07	2.1E-05	2.3E-04	9.2E-05	9.2E-03	3922.9	2454.9	4056.6	1141	748.4	2149.4	2175.5	1571.4	2150.7	1245.7	805.5	1524.6	No	No	No	expressed protein
Os.22842.1.S1_at	LOC_Os12g25170	3	2.0E-07	8.3E-05	2.8E-04	1.1E-05	5.3E-03	4.8E-02	691.5	672.5	729.4	346.1	429.6	464.7	152.6	174.8	140.7	104.3	142.9	168.9	Yes	No	No	disease resistance protein RGA2, putative, expressed
Os.40389.1.S1_at	LOC_Os12g25370	4	3.0E-06	3.2E-06	1.7E-06	7.2E-05	5.7E-04	1.8E-03	37.8	54.2	64.1	7.1	20.8	15.3	1	2.7	0.7	8.7	4.4	12.3	Yes	Yes	Yes	retrotransposon protein, putative, unclassified
Os.9997.1.S1_at	LOC_Os12g25370	6	6.8E-08	1.0E-07	1.1E-07	5.2E-06	6.0E-05	3.2E-04	152.6	179.8	147.4	3	3.9	4.6	2.7	3.1	2.9	22.7	7.4	11.3	Yes	Yes	Yes	retrotransposon protein, putative, unclassified
Os.9997.1.S1_x_at	LOC_Os12g25370	9	3.8E-08	6.6E-08	7.5E-08	3.5E-06	4.5E-05	2.5E-04	262	270	273.3	2.3	27.8	4.9	2.1	14.9	3	2.5	5.8	2.4	Yes	Yes	Yes	retrotransposon protein, putative, unclassified
Os.8762.1.S1_at	LOC_Os12g25630	11	3.7E-10	2.5E-10	5.8E-09	2.1E-07	1.8E-06	5.1E-05	3502.4	3528.1	3940.4	744.2	678.2	901	955.1	933.6	988.7	399.1	387.6	334	Yes	No	No	sulfite oxidase, putative, expressed
Os.51010.1.S1_at	LOC_Os12g26690	3	4.8E-09	8.3E-06	2.2E-06	9.5E-07	1.1E-03	2.1E-03	372.2	332.3	335.8	611.7	461	435.4	70.5	97.3	99.1	121.8	107.5	85.1	Yes	Yes	Yes	expressed protein
Os.46345.1.A1_s_at	LOC_Os12g28550	5	1.4E-08	1.9E-05	1.9E-05	1.9E-06	2.0E-03	8.6E-03	23.7	3	17.6	4.4	47.6	26.7	634.5	756.6	486.5	477.3	371.7	428.2	Yes	Yes	Yes	ATPase 3, putative, expressed
Os.11305.1.S1_at	LOC_Os12g29400	3	6.4E-04	2.7E-06	5.5E-04	4.3E-03	5.2E-04	7.4E-02	828.2	774.6	759.3	546.6	357.9	276.4	508.4	682.7	573.2	416.2	416.5	410.9	No	No	Yes	ABA-responsive protein, putative, expressed
Os.26979.1.S1_x_at	LOC_Os12g29950	6	2.7E-05	4.8E-05	5.6E-04	3.7E-04	3.7E-03	7.5E-02	125.2	250	216.6	355.5	391	284.6	447.5	556.8	360.1	1308	1464.7	835.7	No	No	No	nitrate and chloride transporter, putative, expressed
OsAffx.31980.1.S1_at	LOC_Os12g32400	3	2.1E-06	9.8E-05	1.9E-04	5.7E-05	6.0E-03	3.7E-02	43.1	19.2	32.8	46.1	37	34	56.4	69	69.8	156	164.9	145.6	Yes	No	No	DNA binding protein, putative
Os.15688.1.S1_at	LOC_Os12g36110	6	4.4E-07	8.4E-08	1.4E-05	1.8E-05	5.3E-05	6.9E-03	2896.4	4279.5	3899.4	775.8	1321.1	1085.1	1355.8	1941.8	1279.4	545.7	506.2	774.3	Yes	No	No	calmodulin binding protein, putative, expressed
Os.15688.2.S1_x_at	LOC_Os12g36110	9	6.8E-09	2.6E-09	4.6E-07	1.2E-06	6.4E-06	7.8E-04	3677	4331.4	3987.4	1285.3	1620.6	1374.8	1604	1897.6	1474.9	784.2	705.8	661.8	Yes	No	No	calmodulin binding protein, putative, expressed
Os.48206.1.S1_at	LOC_Os12g36220	11	8.9E-11	7.0E-08	7.1E-08	9.1E-08	4.6E-05	2.4E-04	25.5	3.8	18.1	1.5	79.3	85.1	5973.9	5486.8	5297.8	13347	14984.8	12684.7	No	No	No	MPI, putative
Os.17439.1.S1_at	LOC_Os12g36270	4	1.0E-08	1.8E-05	2.0E-05	1.5E-06	1.9E-03	9.0E-03	844.6	890.4	796.4	469.1	577.9	494.7	252.4	426.9	285.1	245.3	231.7	220.1	Yes	No	Yes	expressed protein
Os.8771.1.S2_x_at	LOC_Os12g36490	3	5.1E-05	1.1E-03	6.4E-04	6.2E-04	3.1E-02	8.2E-02	9.7	40	21.3	74.7	85.6	91.6	26	4.8	1	14.3	16	13.1	No	No	Yes	expressed protein
Os.24180.1.A1_s_at	LOC_Os12g36920	5	3.1E-04	1.7E-06	2.6E-05	2.5E-03	3.7E-04	1.0E-02	1148.9	1373.9	1339	271.7	333.8	350.1	605.7	633.6	723.5	401.2	395	359.7	No	No	No	calmodulin-binding protein, putative, expressed
Os.52191.1.A1_at	LOC_Os12g38850	5	2.1E-07	2.0E-05	1.0E-03	1.1E-05	2.0E-03	1.1E-01	312.1	260.2	313.2	419.1	335	341.2	356.5	384.3	403.9	731.2	674.3	776.7	Yes	No	No	expressed protein
OsAffx.32104.1.S1_at	LOC_Os12g39850	3	1.6E-03	2.8E-04	1.1E-03	8.7E-03	1.2E-02	1.1E-01	16.7	48.6	33.5	74.6	69.9	44.7	41.3	45.9	40.4	233.1	109.8	182.3	No	No	No	DNA binding protein, putative
Os.7855.1.S1_at	LOC_Os12g40900	5	2.5E-07	1.2E-07	2.9E-05	1.3E-05	6.7E-05	1.1E-02	1589.2	1893.2	2174.6	2931.2	3077	3030.8	1918.6	2558	2132.1	4872.8	5253.7	5518.6	Yes	No	No	OsIAA31 - Auxin-responsive Aux/IAA gene family member, expressed
Os.55193.1.S1_at	LOC_Os12g42280	10	8.7E-06	1.7E-06	8.6E-06	1.6E-04	3.8E-04	5.1E-03	817.2	937.7	867.1	63	65.7	47.7	189	289.1	164	58	52.4	36.1	Yes	No	No	viviparous-14, putative, expressed
OsAffx.32169.1.S1_at	LOC_Os12g43310	4	1.3E-08	1.2E-08	2.7E-08	1.8E-06	1.5E-05	1.4E-04	25.5	19.8	7.5	87.7	18.2	62.2	20.9	50.4	68.3	232.5	344.1	341.8	No	No	Yes	hypothetical protein
OsAffx.32169.1.S1_x_at	LOC_Os12g43310	6	4.6E-07	4.0E-07	9.1E-07	1.9E-05	1.4E-04	1.2E-03	18.8	25.5	19.3	82.9	17.7	91.1	54.1	106.9	48.6	266.8	319	501.3	No	No	Yes	hypothetical protein
OsAffx.20101.1.S1_at	LOC_Os12g43320	3	1.1E-04	1.4E-04	1.2E-04	1.1E-03	7.7E-03	2.7E-02	42.6	9.7	4.2	55.2	41.7	33.4	15.6	7.9	30.6	95.1	117.9	107	Yes	No	Yes	hypothetical protein
OsAffx.20102.1.S1_at	LOC_Os12g43330	9	6.8E-10	9.4E-10	1.1E-09	3.0E-07	3.5E-06	1.7E-05	12.9	47.5	36.1	49.4	36.7	35.7	58.9	62.1	55.3	689.5	772.2	748.2	Yes	Yes	Yes	hypothetical protein
Os.39257.1.S1_at	LOC_Os12g43340	6	2.4E-07	2.0E-05	1.3E-05	1.2E-05	2.0E-03	6.6E-03	682.5	664.4	574.9	1445.2	1253.2	1400.2	487.1	614.7	830.9	680	576.8	821.3	Yes	No	Yes	actin-depolymerizing factor, putative, expressed
Os.6867.1.S1_x_at	LOC_Os12g43380	6	1.6E-07	4.3E-04	5.1E-04	9.3E-06	1.7E-02	7.0E-02	1749.3	2018.8	2135.7	1023.7	1022.3	1129.6	10.3	34.1	6.3	8.2	11.9	38.6	Yes	Yes	Yes	thaumatin-like protein precursor, putative, expressed
Os.49466.1.S1_at	LOC_Os12g43430	6	9.0E-09	4.7E-08	2.0E-05	1.4E-06	3.6E-05	8.8E-03	1444.8	1421.2	1435.5	554.5	642.4	502.8	441.1	492	425.2	188.1	287.8	216.6	No	No	Yes	thaumatin-like protein precursor, putative, expressed
Os.50620.1.S1_at	LOC_Os12g44190	7	9.9E-08	2.8E-07	3.4E-06	6.6E-06	1.2E-04	2.7E-03	2251.8	3261	3005.4	665.4	841.4	783.3	732.6	1042.6	698.4	267.6	213.4	204.6	No	No	No	ATPase 3, putative, expressed
Os.56885.1.S1_at	LOC_Os12g44210	5	9.7E-06	1.9E-05	1.8E-04	1.7E-04	2.0E-03	3.7E-02	166.8	281.1	141.4	41.2	85.8	79.6	37.4	39.9	42.4	40.2	19.4	28.2	Yes	Yes	No	cell Division Protein AAA ATPase family, putative, expressed
OsAffx.32185.1.S1_s_at	LOC_Os12g44220	10	3.2E-11	3.9E-10	1.4E-09	5.6E-08	2.2E-06	1.9E-05	1339.5	1247.7	912	403.4	279	155.5	66.4	87	117.8	7.7	44.6	1.9	No	Yes	Yes	cell Division Protein AAA ATPase family, putative, expressed
OsAffx.32185.1.S1_x_at	LOC_Os12g44220	9	2.7E-11	4.5E-10	8.1E-10	5.1E-08	2.4E-06	1.5E-05	1585.7	1117.2	1090.7	440.4	209.6	201	22.2	45.5	55.1	12.6	16.7	42.1	No	Yes	Yes	cell Division Protein AAA ATPase family, putative, expressed
Os.14650.1.S1_at	Not Matched	5	5.1E-08	1.1E-06	6.2E-07	4.3E-06	2.8E-04	9.4E-04	10.7	22.3	2.7	5.8	4.4	7.2	180.5	345.9	251.5	28.8	109.7	82.7	N/A	N/A	N/A	Transcribed sequence
Os.15838.1.A1_at	Not Matched	11	7.3E-09	2.9E-08	1.2E-07	1.2E-06	2.7E-05	3.2E-04	247.9	257.8	260.8	66.1	55.4	84.8	849.9	1799.2	967.2	158.1	251	203.4	N/A	N/A	N/A	Transcribed sequences

Probe Set ID	Locus ID	probe #	ANOVA p-value			FDR q-value			Nipponbare MV			Nipponbare CK			93-11 MV			93-11 CK			Indel (Indica vs Japonica)			Annotation
			C	T	C×T	C	T	C×T	Repeat 1	Repeat 2	Repeat 3	Repeat 1	Repeat 2	Repeat 3	Repeat 1	Repeat 2	Repeat 3	Repeat 1	Repeat 2	Repeat 3	upstream	gene	downstream	
Os.23059.1.S1_s_at	Not Matched	4	1.4E-04	2.0E-05	3.0E-03	1.3E-03	2.0E-03	2.0E-01	129.4	170.7	130.6	791.2	616.8	891.5	510.7	503.3	417	3112.7	1897.7	2183.5	N/A	N/A	N/A	Transcribed sequence
Os.23218.1.S1_x_at	Not Matched	3	3.9E-08	2.7E-05	1.8E-04	3.6E-06	2.5E-03	3.6E-02	256.5	254.1	309.5	166	229.9	284.3	953.4	1025.8	930.5	625.9	740.4	507.6	N/A	N/A	N/A	Z279a snoRNA
Os.24314.1.A1_s_at	Not Matched	10	3.4E-09	1.8E-09	5.7E-08	7.7E-07	5.2E-06	2.1E-04	248.6	225.5	391.1	74.7	89.7	61.4	1312.3	1465.9	1246	189.3	277	217.8	N/A	N/A	N/A	Transcribed sequence
Os.37644.1.S1_at	Not Matched	5	1.2E-05	9.9E-10	2.3E-05	2.0E-04	3.6E-06	9.7E-03	1723.7	2406.9	2261.7	93.9	156	139.8	1265.3	1492.4	1373.3	127.4	62.8	42.7	N/A	N/A	N/A	Oryza sativa (japonica cultivar-group) cDNA clone:002-134-B03, full insert sequence.
Os.51405.1.S1_at	Not Matched	5	1.6E-08	6.5E-04	8.3E-04	2.0E-06	2.2E-02	9.6E-02	839.5	1030.6	1082.5	1410.8	1514.3	1544.2	28.6	43.1	9.9	79.1	60.3	90.6	N/A	N/A	N/A	Oryza sativa (japonica cultivar-group) cDNA clone:001-106-D06, full insert sequence.
Os.54362.1.S1_at	Not Matched	7	2.2E-06	6.8E-06	1.1E-04	5.9E-05	9.6E-04	2.6E-02	108.6	251.9	206	17.3	67.4	71.8	475.7	430.1	443.8	107.9	144.7	221.4	N/A	N/A	N/A	Oryza sativa (japonica cultivar-group) cDNA clone:J033135I20, full insert sequence.
Os.54387.1.S1_at	Not Matched	5	8.6E-07	2.3E-05	1.9E-05	2.9E-05	2.3E-03	8.7E-03	342.7	236.7	238.6	151.9	121.2	192	76.3	50.9	51.3	89.3	51	27.8	N/A	N/A	N/A	Oryza sativa (japonica cultivar-group) cDNA clone:J033149A03, full insert sequence.
Os.55079.1.S1_at	Not Matched	4	1.8E-04	2.7E-09	1.1E-04	1.7E-03	6.5E-06	2.6E-02	3832.9	4406.4	4747.2	160.8	178.7	208.3	2758.9	3268.7	2905.3	267	155	157.6	N/A	N/A	N/A	Oryza sativa (japonica cultivar-group) cDNA clone:002-128-A08, full insert sequence.

(a) present the minimum ANOVA test p-value of the probes in the probe set
(b) present the minimum FDR q-value of the p-values in the probe set
(c) C presents Cultivar effect of the two-way ANOVA test
(d) T presents Treatment effect of the two-way ANOVA test
(e) C×T presents the Cultivar×Treatment effect of the two-way ANOVA test
(f) "Yes" means a possible InDel region in the upstream, gene region, and/or downstream of the gene
(g) "No" means the gene without InDel region
