# Supplementary material for: Gene Expression Profiles Deciphering Rice Phenotypic Variation between Nipponbare (Japonica) and 93-11 (Indica) during Oxidative Stress
Source: PLoS One. 2010 Jan 8;5(1):e8632. doi: 10.1371/journal.pone.0008632 (PMC2799674; doi:10.1371/journal.pone.0008632)
Supplement: Table S2 — The primer sequences of selected genes for real-time RT-PCR analysis. (0.05 MB DOC) [file pone.0008632.s006.doc]

Table S2. The primer sequences of selected genes for real-time RT-PCR analysis

| **Gene Name** | **Forward** | **Reverse** |
| --- | --- | --- |
| **LOC_Os10g38360** | GCTGCCACCAACTGAAGATA | CCACCCCTTCATTCAACATT |
| **LOC_Os10g38340** | TTGTCGTGTGCTTCCACATT | CGGCAATACGAACTGATGAA |
| **LOC_Os10g38470 (P1)** | CAGCGCACAAGTGATTGACT | CTGGACACGTAACGTACACGA |
| **LOC_Os10g38470 (P2)** | GGGAACAGTTTGTCCAAGGT | ACCCAGACGCCACTTGTACT |
| **LOC_Os10g38189** | CCGATAGGTTGGTCGAGTTC | AATTGGTGAAAGCAGCATCC |
| **LOC_Os07g23570** | CCTAAGGCTGTACAGCCCTG | ATCGGGATCGTCAATAGTGC |
| **LOC_Os01g43700** | GTATAGATCCGTGCATCCGG | GGAACATGGAGCTTCCAGTG |
| **LOC_Os03g55240** | GCTAGCAATCGCTCTTTGAGA | GAAATTGAAATACCTGGCGTGT |
| **LOC_Os11g10550** | GAAGACGCTTGCTCATGTCA | GCAGCCTGGAGTGAACTTCT |
| **LOC_Os07g33690** | TTGCAGATACCATCAGACGC | GCTCAGTGTGATGTGCGAAT |
| **LOC_Os03g62480** | CTTGTTGGCCTAGTGGCAAT | CATACCGACACCACGAGTTG |
| **LOC_Os06g18140** | GGAATGTGAGAGAAGCGAGG | CACCTGTGGACTGTGGTGAC |
| **LOC_Os08g39840** | TACCAGCTGATGAAGCCCTT | TGGAGCGTTTTGTCTCATCA |
| **LOC_Os01g71680** | GGACAGATGGAGGCGTACAT | TAGACCGGCGACTTGTTAGG |
| **LOC_Os02g12380** | AAGCAAGTTCCTACTGCGGA | GTTCATAGTGAGCTTCCCGG |
| **LOC_Os02g12350** | CAGCAGCTATGCACCAGAAG | TGCCAACTTAAGAGCATGCA |
| **LOC_Os05g12240** | GTACTGAGCATGCACTTGGC | GGCACCTGGATAGAGAGGTG |
| **LOC_Os02g56700** | GAGGAAGAGCTTGCATGAGG | GGTTCTACAAACATGCACGC |
| **LOC_Os10g12080** | CAAGGAGGAGCTGTTCAAGC | CCCCTTGTATTTGGGAATGA |
| **LOC_Os02g56460** | AGAGGCTCAAGGATTTGGGT | TTCTGCTGCATGCATAGGAC |
