# Supplementary material for: Gene Expression Profiles Deciphering Rice Phenotypic Variation between Nipponbare (Japonica) and 93-11 (Indica) during Oxidative Stress
Source: PLoS One. 2010 Jan 8;5(1):e8632. doi: 10.1371/journal.pone.0008632 (PMC2799674; doi:10.1371/journal.pone.0008632)
Supplement: Table S3 — The primer sequence for InDel detection. (0.03 MB DOC) [file pone.0008632.s007.doc]

Table S3. The primer sequence for InDel detection

| **Gene Name** | **Forward** | **Reverse** |
| --- | --- | --- |
| LOC_Os11g12340 | AAGGAAACGGTCCCAAGACT | CCACCTCCTCTGGTGTGTTT |
| LOC_Os02g56700 | CCCTCCATGTATGCCTCACT | CTCCCTCAAGATCAAGCAGG |
| LOC_Os11g10550 | ACATCCTGCTCCACTCCTCT | CGCAATTTCTGCAAAACAAA |
| LOC_Os07g33690 | ATCTGGCAGCCACGAAGC | ACATCTCCTGCATGGGAAAC |
| LOC_Os10g38360 | GAGCAGAGACCATCGGAGTT | AGCTCGATCAGCTTGTCCAC |
